# A Bio‐Based Chiroptical Overcrowded Alkene From Flavanone: Photoreactivity and Theoretical Investigation

**DOI:** 10.1002/chem.71180

**Published:** 2026-05-29

**Authors:** Deryl H. Limawan, Adrien Combe, Anthony Bongso, Bayu Dwiputra, Atthar L. Ivansyah, Roza R. Weber, Charlotte N. Stindt, Robby Roswanda, Ben L. Feringa

**Affiliations:** ^1^ Organic Chemistry Division, Department of Chemistry, Faculty of Mathematics and Natural Sciences Institut Teknologi Bandung Bandung Indonesia; ^2^ Stratingh Institute for Chemistry, Faculty of Science and Engineering University of Groningen Groningen the Netherlands; ^3^ Inorganic and Physical Chemistry Division, Department of Chemistry, Faculty of Mathematics and Natural Sciences Institut Teknologi Bandung Bandung Indonesia

## Abstract

The design of light‐driven overcrowded alkenes often relies on complex synthetic strategies to achieve precise control over rotational motion. In this study, we demonstrate a sustainable approach to design an overcrowded alkene and an *O*‐heterocyclic phenanthrene derivative using a bio‐based enantiopure flavanone, *(‐)*‐isolonchocarpin, as starting material. The synthesis involves a McMurry deoxygenative homocoupling reaction to generate a sterically hindered overcrowded alkene, which can further undergo photochemical *(E*/*Z)*‐isomerization and oxidative 6π‐electrocyclization. The oxidation product was shown to have emission properties, highlighting the potential of synthesizing bio‐based chromophoric compounds in minimal steps. Computational studies using spin‐flip time‐dependent density functional theory (SF‐TDDFT) reveal that the inherent chirality of the starting material induces asymmetry in the rotational pathway, biasing the directionality of motion. By integrating enantiopure natural products into functional photoresponsive materials, this work offers a more sustainable route to develop new bio‐based photoresponsive molecular machines while deepening our understanding of chirality‐driven motion in dynamic systems.

## Graphical abstract

A sustainable route to photoresponsive molecular systems is achieved using the bio‐based flavanone *(‐)*‐isolonchocarpin from *Tephrosia vogelii*. Through McMurry homocoupling and light‐driven 6‐π‐electrocyclization, an overcrowded alkene and a fluorescent *O*‐heterocyclic phenanthrene are synthesized. SF‐TDDFT (Spin‐Flip Time‐Dependent Density Functional Theory) reveals how natural chirality biases molecular motion.

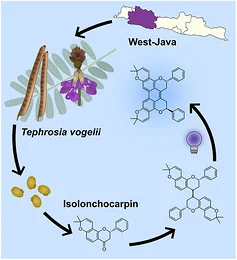

## 1 Introduction

Overcrowded alkenes represent a unique class of photoresponsive systems due to their distinctive axial chirality near the central olefin, making them highly suitable for the development of dynamic light‐driven smart materials. Their inherent chirality and stable helical structure have provided the foundation for numerous chiroptical molecular photoswitches and light‐driven molecular rotary motors [1, 2]. These systems utilize the photoinduced *(E*/*Z)*‐isomerization process to switch between different molecular geometries. Overcrowded alkene‐based molecular switches have found applications in various fields, including photochromic materials, molecular machines, and smart materials, demonstrating their versatility as photoresponsive systems [3, 4, 5]. Recent progress in this area has led to simpler synthetic pathways and provided insights into how substituents affect the photochemical behavior of these alkenes [6]. Meanwhile phenanthrene, as a key chromophoric building block, has attracted significant interest for its potential use in various materials applications, including solar cells [7, 8, 9], phototransistors [10], organic thin‐film transistors (OTFTs) [10, 11], and organic light‐emitting diodes (OLEDs) [12, 13, 14, 15]. Beyond optoelectronic applications, phenanthrene‐based molecules are also employed in the biomedical field as triplet photosensitizers [16, 17]. However, synthesizing phenanthrene‐based chromophores presents significant challenges. Most phenanthrene frameworks are prepared using phenanthrenequinone‐based compounds as starting material, which are highly reactive and may lead to a reduced scope [7, 8, 9, 11]. Recently developed synthetic methods include the use of electrochemistry [18, 19], photocatalysts [20], Lewis‐acid catalysts [21], or transition‐metal‐based catalysts [22, 23], to facilitate the synthesis of phenanthrene‐like derivatives.

**SCHEME 1:**
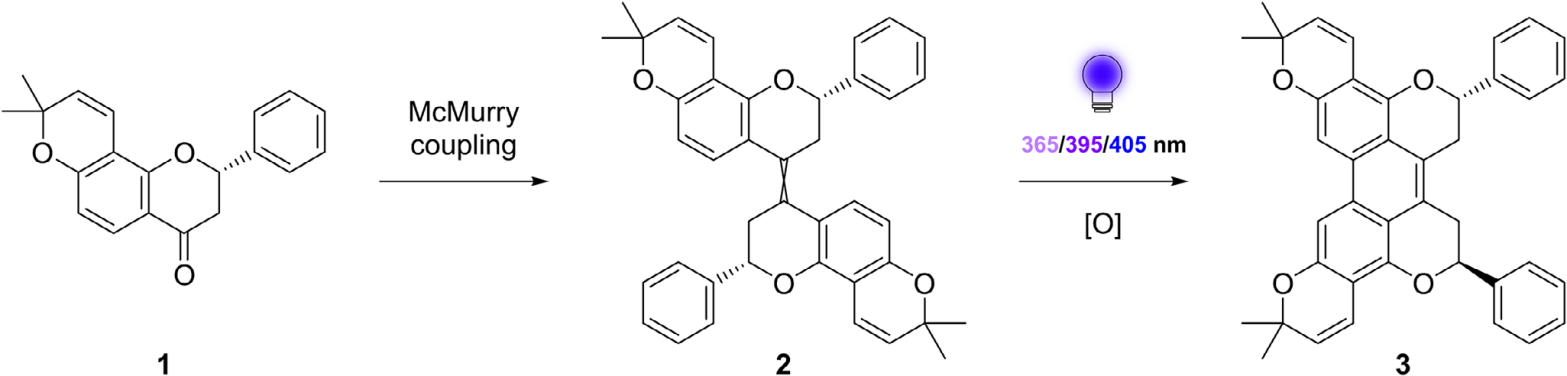
Synthetic route from *(‐)*‐isolonchocarpin (**1**) to phenanthrene‐like chromophore (**3**).

In recent years, the utilization of bio‐based secondary metabolites as starting materials has started to attract some attention [24, 25, 26, 27]. This is because bio‐based secondary metabolites often possess favorable characteristics such as enantiopurity and are readily available [28], which are beneficial for building a more complex molecular system. In this study, we demonstrate the synthesis of an overcrowded alkene‐based molecular switch and a phenanthrene‐like derivative using a natural product as starting material (Scheme 1). Specifically, we utilize *(‐)*‐isolonchocarpin **1**, a major secondary flavanone‐derived metabolite isolated from *Tephrosia vogelii* [28]. This compound features an aromatic ring conjugated to a carbonyl moiety, which allows it to undergo dimerization *via* the McMurry homocoupling reaction, forming a stilbene‐like product. The resulting overcrowded alkene **2** predominantly adopts the *E* configuration. The final step of the synthesis involves near‐UV to blue‐light irradiation, which triggers the photoisomerization from *E* to *Z* configuration, followed by oxidative photocyclization, also known as the Mallory reaction [29, 30], to yield a phenanthrene‐like chromophore **3**. This synthetic route is relatively straightforward due to the use of a bio‐based starting material. Moreover, the two olefins present on the chromene‐derived sub‐structure of **2** offer potential for further functionalization toward various applications. Achiral olefins would possess a symmetrical potential energy surface along the *(E*/*Z)*‐photoisomerization reaction coordinate where clockwise rotation (CR) with respect to the olefin bond axis exhibits an identical energy profile with its counterclockwise rotation (CCR) counterpart. A theoretical mechanistic study using spin‐flip time‐dependent density functional theory (SF‐TDDFT) revealed that the two stereogenic centers in *(E)*‐**2** play a crucial role in breaking this symmetry, thereby creating a bias between the CR and CCR pathways. This insight enhances our understanding of the reaction mechanism and the dynamic behavior of these molecular systems as well as the chiroptical switching function.

## 2 Results and Discussion

**FIGURE 1:**
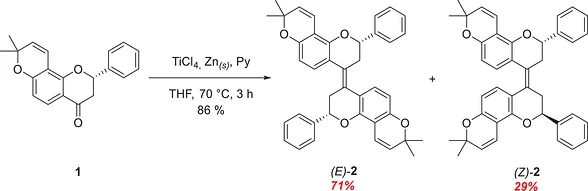
McMurry homocoupling reaction of flavanone **1**. *E*:*Z* ratio determined by ^1^H NMR spectroscopy.

*(‐)*‐Isolonchocarpin **1** was isolated as an enantiopure compound from dried *Tephrosia vogelii* pod peels through maceration in acetone, followed by purification using vacuum liquid chromatography [28], and recrystallization from *n*‐heptane. The chemical structure of flavanone **1** was confirmed by proton nuclear magnetic resonance (^1^H NMR) spectroscopy, with detailed procedures and characterization provided in the Supporting Information. Flavanone **1** was then subjected to a reductive McMurry coupling reaction using TiCl_4_, zinc powder and pyridine in anhydrous THF (Figure 1). Under these modified reaction conditions, the expected overcrowded alkene **2**, was obtained in 86% yield with a *E*:*Z* ratio of 71:29.

**FIGURE 2:**
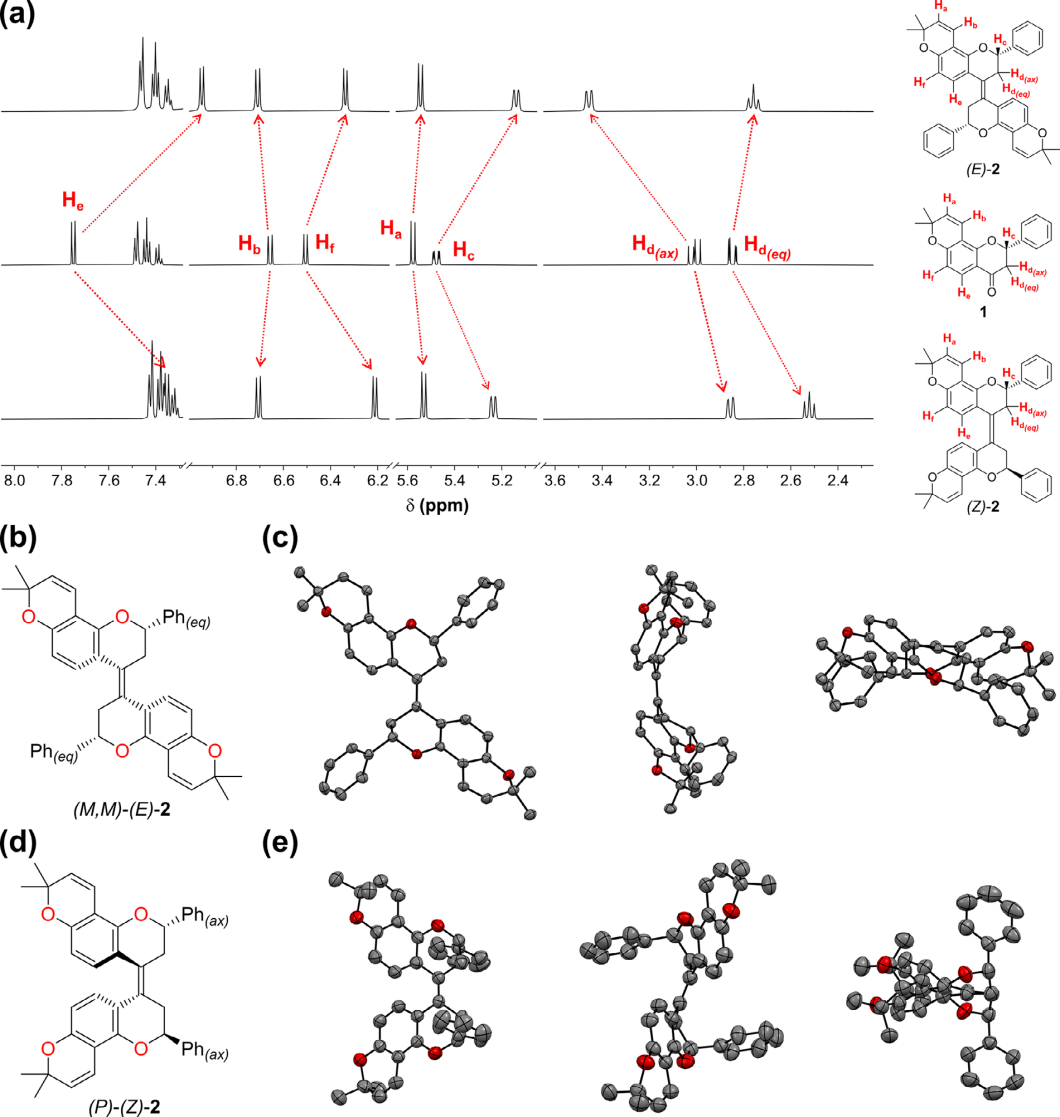
(a) ^1^H NMR spectra of isolated **1**, *(M,M)‐(E)*‐**2** and *(P)*‐*(Z)*‐**2** (600 MHz, 4.0 mM CDCl_3_, 25°C). (b) Chemical structure of *(M,M)*‐*(E)*‐**2** with phenyl groups in equatorial conformation. (c) ORTEP representation of the X‐ray crystal structure of *(M,M)*‐*(E)*‐**2** (with 50% probability) from front view (left), left view (middle), and top view (right). (d) Chemical structure of *(P)*‐*(Z)*‐**2** with phenyl groups in axial conformation. (e) ORTEP representation of the X‐ray crystal structure of *(P)*‐*(Z)*‐**2** (with ellipsoids set at 50% probability) from front view (left), left view (middle), and top view (right). Hydrogen atoms are omitted for clarity.

The pure *(E)*‐**2** isomer was successfully isolated by precipitation from methanol and found to be stable under ambient light. Separately, the *(Z)*‐**2** isomer was recrystallized from methanol. The structures of the diastereomers were determined using ^1^H NMR spectroscopy (Figure 2a) and single crystal X‐ray diffraction (XRD) (Figure 2c,e). The ^1^H NMR spectra revealed that the multiplicity pattern of *(E*/*Z)*‐**2** is quite similar to flavanone **1**, with the only significant difference being the chemical shift of protons adjacent to the central olefin, specifically H_c_, H_d_, H_e_, and H_f_ (Figure 2a). These protons displayed more shielded signals compared to those in flavanone **1**, reflecting the conversion of the ketone to an overcrowded alkene.

**FIGURE 3:**
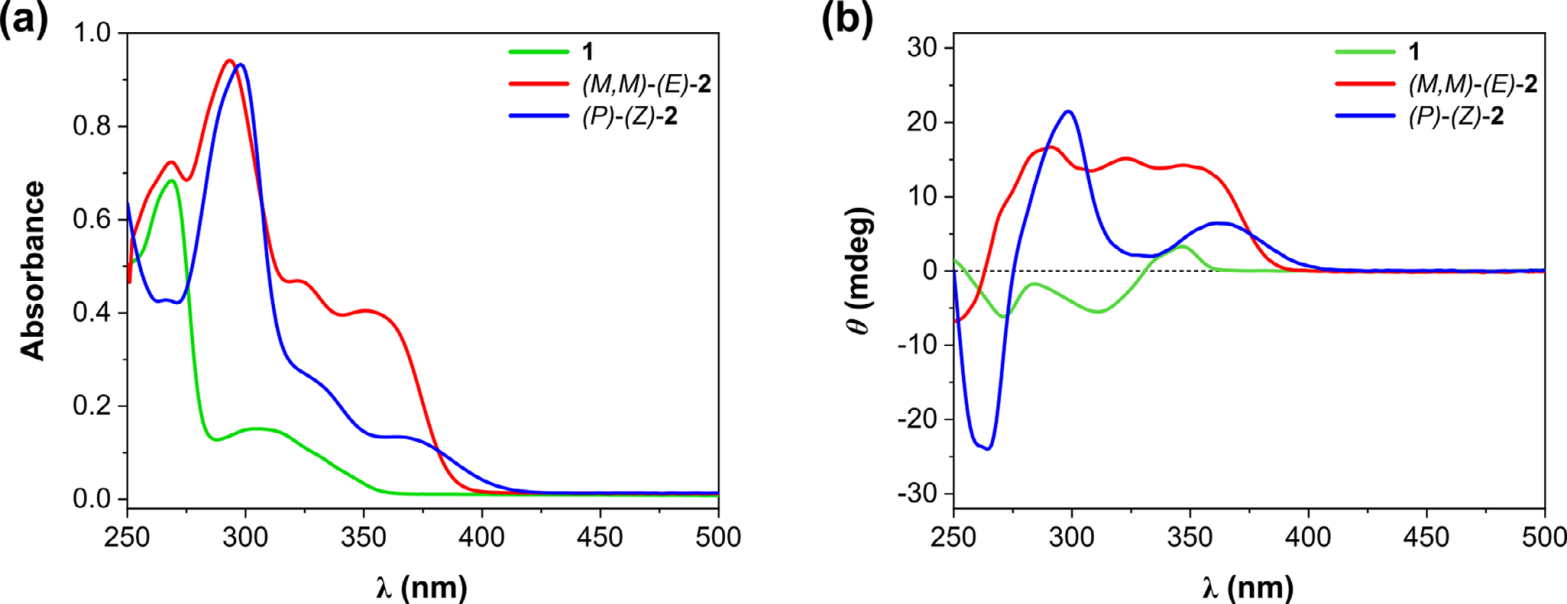
(a) UV‐Vis and (b) CD spectra of **1**, *(M,M)*‐*(E)*‐**2** and *(P)*‐*(Z)*‐**2** (20 µM in DCM, 25°C).

In addition, the chiral overcrowded alkene **2** was further analyzed using UV‐Vis absorption and circular dichroism (CD) spectroscopies (Figure 3). It is important to highlight that the McMurry homocoupling reaction is stereoselective, and generally no epimerization is observed under the reaction conditions [31]. Therefore, we expect that the enantiopurity of the flavanone **1** is preserved during the homocoupling, resulting in the formation of *(2S,2’S)*‐*(M,M)‐(E)*‐**2** and *(2S,2’S)*‐*(P)*‐*(Z)*‐**2** as products. The overcrowded alkene **2** features a more extended conjugated π‐system compared to flavanone **1**, leading to a decrease in the HOMO‐LUMO gap and resulting in a bathochromic shift in the UV‐Vis absorption spectra relative to flavanone **1**. Finally, the absolute configuration of the two stereogenic centers and helicity of the synthesized *(E)‐* **2** was confirmed to be *(2S,2’S)*‐*(M,M)‐(E)*‐**2** by comparing the experimental CD spectra with calculated spectra (see Figure S16), supported by the analysis of the obtained NOESY spectra. Furthermore, the ORTEP diagram of the obtained *(E)‐* **2** crystal shows *M,M* helicity with the phenyl moiety in equatorial position (Figure 2b,c), whereas the *(Z)*‐**2** crystal adopts *P* helicity with the phenyl group in axial position (Figure 2d,e).

**FIGURE 4:**
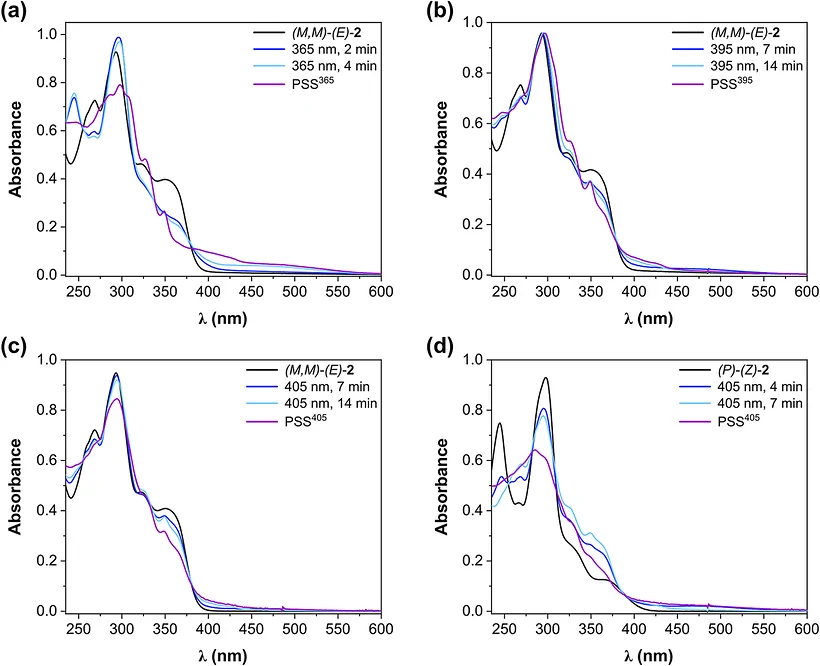
UV‐Vis absorption spectra of the photochemical reactions by irradiating a solution of *(M,M)‐(E)‐* **2** at (a) 365 nm, (b) 395 nm, and (c) 405 nm (20 µM in DCM, 1.0 A, 25°C). (d) UV‐Vis absorption spectra of the photochemical reactions by irradiating a solution of *(P)*‐*(Z)*‐**2** at 405 nm (20 µM in DCM, 1.0 A, 25°C).

The potential for overcrowded alkene **2** to undergo photocyclization was investigated using UV‐Vis, CD, and ^1^H NMR spectroscopies. Upon irradiation of *(M,M)*‐*(E)*‐**2** at 365, 395, and 405 nm in DCM, the UV‐Vis spectra showed a progressive bathochromic shift (Figure 4). Importantly, the spectra did not show one consistent set of well‐defined isosbestic points throughout the irradiation. In a simple photochemical reaction, such behavior would normally be expected for a clean conversion between two species. The absence of this pattern therefore suggests that several photochemical processes occur successively or simultaneously, rather than a single direct transformation. Overall, the data are consistent with an initial *(E/Z)*‐photoisomerization followed by additional irreversible photochemical steps.

Within the first 2 min, irradiation at 365 nm showed two isosbestic points at 255 and 379 nm together with pseudo‐isosbestic points at approximately 285 and 313 nm (Figure 4a). This is consistent with an initial photoisomerization step. Interestingly, this spectrum upon irradiation closely resembled the spectrum of pure *(P)*‐*(Z)*‐**2** (Figure 3a), suggesting formation of this isomer in the early stage of irradiation. Upon prolonged light‐exposition, the spectral changes became more complex. In fact, a new set of isosbestic and pseudo‐isosbestic points appears together with a gradual increase in absorbance in the 400–575 nm region (Figures 4a and S19). This low‐energy absorption is consistent with the formation of a more extended π‐conjugated system and therefore supports the onset of photocyclization toward phenanthrene derivative **3**. After approximately 4 min of irradiation, the lack of clearly defined isosbestic point indicated that multiple photochemical processes were occurring concurrently. The photostationary state at 365 nm was reached after around 60 min.

A similar behavior was observed upon irradiation of *(M,M)*‐*(E)*‐**2** using 395 and 405 nm. In both cases, the initial spectral evolution, again, suggested an early photoisomerization step, followed by more complex spectral changes at longer irradiation times (Figure 4b,c; Figures S20 and S21). Compared to 365 nm irradiation, the increase in absorbance within the 400–575 nm range was less pronounced, indicating that photocyclization occurs less or more slowly under these conditions. At longer irradiation times, the appearance of additional isosbestic and pseudo‐isosbestic points further supported the presence of consecutive photochemical transformations rather than a single unimolecular process. The photostationary states at 395 and 405 nm were reached after approximately 90 min.

To further probe the reactivity of the *Z* isomer, a solution of *(P)*‐*(Z)*‐**2** was irradiated at 405 nm. Its initial absorption spectrum was slightly redshifted relative to that of *(M,M)*‐*(E)*‐**2** (Figure 3a). Upon irradiation, a distinct sequence of spectral changes was observed, including a clear isosbestic point at 254 nm and several pseudo‐isosbestic points during the early stage of the process (Figures 4d and S30). These observations again indicate a multistep reaction pathway. Equal to the *E* isomer, an increase in absorbance in the 400–575 nm region was observed, which is consistent with formation of phenanthrene derivative **3** through photocyclization of *(P)*‐*(Z)*‐**2**. At longer irradiation times, the spectral evolution also became increasingly complex, pointing out multiple successive photochemical reactions.

Overall, the UV‐Vis absorption spectroscopy studies point to a multistep photochemical sequence involving initial *(E*/*Z)*‐photoisomerization and subsequent irreversible transformations, most likely photocyclization and oxidation. To gain further insight into the structural and chiroptical consequences of these processes, CD spectroscopy and ^1^H NMR spectroscopy was subsequently employed.

**FIGURE 5:**
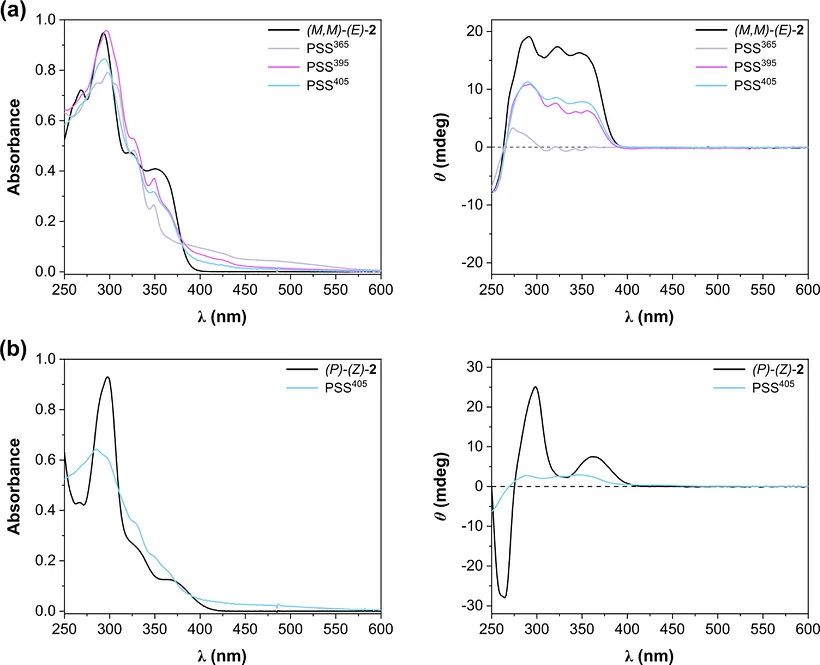
(a) UV‐Vis absorption (left) and CD (right) spectra of *(M,M)*‐*(E)*‐**2** and the PSSs at 365, 395, and 405 nm (20 µM in DCM, 1.0 A, 25°C). (b) UV‐Vis absorption (left) and CD (right) spectra of *(P)*‐*(Z)*‐**2** and the PSS at 405 nm (20 µM in DCM, 1.0 A, 25°C).

CD spectroscopy was used to gain information about any chiral or conformational changes in *(M,M)‐(E)*‐**2** and *(P)*‐*(Z)*‐**2** during irradiation. Irradiation of *(M,M)‐(E)*‐**2** at 365 nm seems to significantly decrease the CD signal, suggesting a partial loss of chiroptical response which can be due to the formation of the phenanthrene chromophore **3** (Figure 5a). Interestingly, the CD spectra at PSS^395^ and PSS^405^ after photoirradiation of *(M,M)‐(E)*‐**2** at 395 and 405 nm show a lower decrease of ellipticity, confirming a lower conversion of *(M,M)‐(E)*‐**2** (Figure 5a). In addition, these two CD spectra have a similar ellipticity intensity signal, indicating a comparable photoconversion of *(M,M)‐(E)*‐**2** upon 395 and 405 nm photoirradiation. The same measurement has been done with the PSS^405^ mixture obtained from *(P)*‐*(Z)*‐**2**, showing a similar loss of ellipticity (Figure 5b). These Cotton effect changes suggest a possible competing photocyclization (*vide infra*).

The ^1^H NMR spectrum of *(M,M)‐(E)*‐**2** in C_6_D_6_ irradiated with 395 nm light is shown in Figure S22. The results reveal a decrease in the integration of the H_f_ doublet peak, accompanied by the appearance of a new H_f_ singlet peak. This indicates the formation of a product lacking the H_e_ hydrogen atom, suggesting the subsequent formation of *(Z)*‐**2** and a photocyclization product during irradiation. The formation of the oxidative photocyclization product, phenanthrene chromophore **3**, was further confirmed by NMR spectroscopy and HRMS (Figure S23). However, the intermediate dihydrogenated phenanthrene could not be isolated or confirmed due to its instability [27, 28].

**FIGURE 6:**
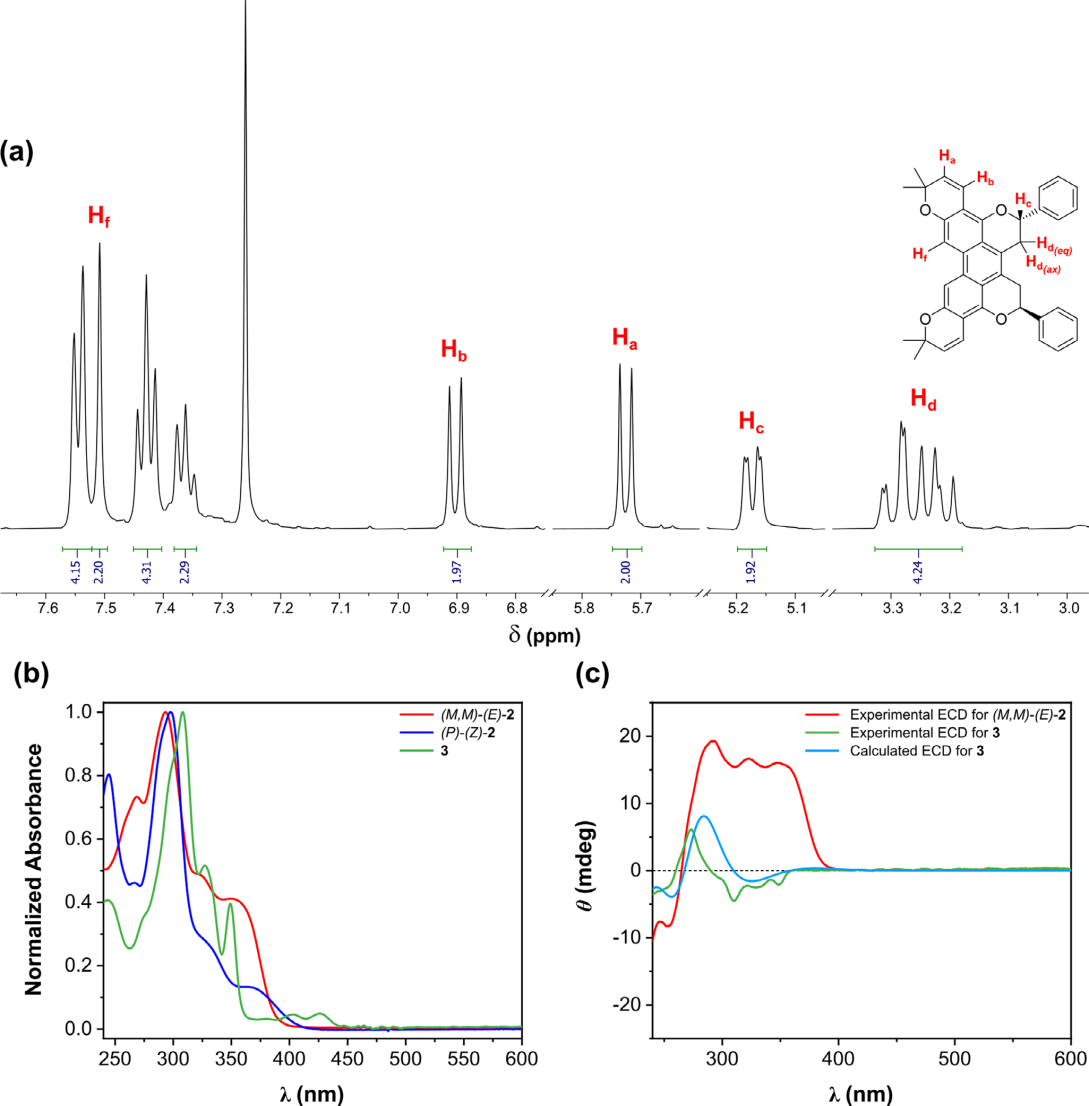
(a) ^1^H NMR spectrum of isolated phenanthrene‐derivative chromophore **3** (500 MHz, 2.0 mM in CDCl_3_, 25°C). (b) Normalized UV‐Vis absorption spectra of *(E)*‐**2**, *(P)*‐*(Z)*‐**2**, and **3**. (c) Experimental ECD spectra for *(M,M)‐(E)‐* **2** and chromophore **3** (38.5 µM in DCM, 25°C) compared to the calculated CD spectrum of chromophore **3** at the *ω*B97X‐D4/cc‐pVDZ level of theory.

Phenanthrene **3** was successfully isolated after 6 h of irradiation (395 nm, acetone) by preparative TLC in 50% yield. ^1^H NMR analysis on the isolated product shows the presence of the singlet H_f_ peak in the aromatic region, confirming previous analysis of the reaction mixture (Figure 6a). In addition, the *J*‐coupling constants of H_c_ and H_d_ suggest that the phenyl groups are also in an equatorial conformation. It was also observed that there is a bathochromic shift in the absorption spectra of **3** compared to *(M,M)‐(E)*‐**2** (Figure 6b), indicating an extension of the conjugation system. The UV‐Vis spectrum of chromophore **3** (Figure 6b) revealed three absorption maxima in the UV region (*λ*_max_ = 308, 327 and 349 nm), alongside two minor maxima in the blue region (*λ*_max_ = 403 and 426 nm). The CD spectra (Figure 6c) demonstrated a positive Cotton effect in the 260–290 nm region, and a negative Cotton effect in the 290–360 nm range and below 260 nm. These results indicate that the stereogenic centers retain their configuration without engaging in chiral‐sensitive transitions at wavelength above 360 nm. Measurement of the CD spectra also shows a decrease in ellipticity compared to *(M,M)‐(E)*‐**2** (Figure 6c), even at higher concentration, suggesting the formation of a planar chemical structure of chromophore **3**. Further analysis of compound **3** revealed two maxima of fluorescence emission at 438 and 465 nm with a quantum yield of around 5% (Figure S36). While modest compared to engineered fluorophores, this value is notable for a bio‐derived organic chromophore, with sharp emission maxima further confirming the enhanced rigidity of **3**. Beyond its role as a mechanistic photoproduct, phenanthrene **3** can also be considered as a rigid and compact π‐conjugated scaffold of potential interest for future chromophore design. Related phenanthrene‐based fused aromatic systems have recently been used as blue‐emissive building blocks in OLED‐based materials, showing that such rigid extended cores can display useful optical and electronic properties [32, 33]. Phenanthrene derivatives have also been explored as fluorescent platforms for imaging and labeling applications, highlighting the value of this motif for probe development [34, 35]. More broadly, these examples suggest that phenanthrene **3** could serve as a promising starting point for the design of functional chromophores in optical, imaging, or photoresponsive materials [36].

**FIGURE 7:**
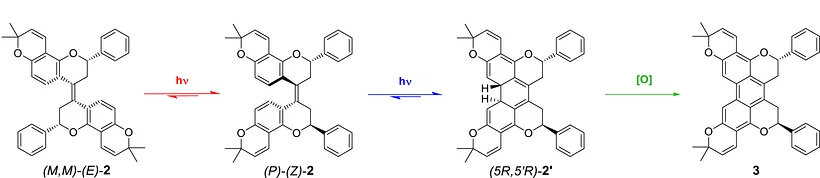
Proposed mechanism of the photoisomerization of overcrowded alkene **2**, and the subsequent cyclization and oxidation yielding chiral phenanthrene derivative chromophore **3**.

Altogether, the experimental data suggest that irradiation of *(M,M)*‐*(E)*‐**2** triggers an initial *(E*/*Z)*‐photoisomerization, which is followed by photocyclization and subsequent oxidation to afford an irreversible photo‐induced product. The proposed reaction scheme of the photocyclization of overcrowded alkene **2** is summarized in Figure 7. This pathway is in line with the photochemical behavior classically observed for *cis*‐stilbene, where photoexcitation can lead not only to isomerization but also to secondary reactions such as oxidative photocyclization, ultimately yielding phenanthrene‐type products [30].

To fully understand the mechanisms of the photochemical reactions and other properties involving electronically excited states, it is essential to investigate both the ground and excited‐state potential energy surfaces (PESs). While linear response time‐dependent density functional theory (LR‐TDDFT) has been widely used to calculate excited PESs, it often predicts an incorrect topology for conical intersections (CIs), which involve the ground state PES, a critical element in understanding excited‐state dynamics [37, 38, 39, 40, 41]. Additionally, the single‐reference nature of LR‐TDDFT can be less suitable for systems that exhibit strong correlation or multireference character. To overcome these limitations, we employed collinear spin‐flip time‐dependent density functional theory (SF‐TDDFT) to calculate the PESs for the two observed photochemical reactions.

**FIGURE 8:**
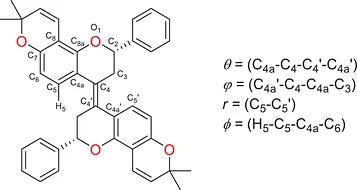
Important internal coordinates involved in the photochemical reactions of *(* *E* *)*‐**2**.

Overcrowded alkene **2**, which features a stilbene moiety, undergoes similar *(E*/*Z)*‐photoisomerization and 6π‐electrocyclization reactions as stilbene and its derivatives. These types of compounds have been the focus of numerous experimental and theoretical studies [42, 43, 44, 45, 46, 47, 48, 49, 50, 51]. Despite being relatively distant from the Fjord region [52], the two carbon stereocenters in **2** are expected to introduce an energy difference between the minimum energy paths (MEPs) for *(E*/*Z)*‐photoisomerization through clockwise rotation (CR) and counterclockwise rotation (CCR) around the central olefin. The key internal coordinates (*θ*, *ϕ*, *r*, *φ*) involved in these reactions are defined in Figure 8. The *(E*/*Z)*‐photoisomerization via the CR and CCR pathways produces *(P)*‐*(Z)*‐**2** and *(M)*‐*(Z)*‐**2**, respectively.

Single crystal XRD of *(Z)*‐**2**, isolated from the McMurry reaction mixture, revealed that the phenyl substituent on C_2_ occupies an axial position, with *P* helicity (Figure 2c). Optimization of the single crystal XRD structure at the *ω*B97X‐D4/cc‐pVDZ level in DCM resulted in *(P)*‐*(Z)*‐**2** *_(ax)_*, the most stable stereoisomer and conformer of *(Z)*‐**2**. The calculated order of stability for the helical stereoisomers and conformers of solution phase *(Z)*‐**2** is *(P)‐(Z)*‐**2** *_(ax)_* > *(M)*‐*(Z)*‐**2** *_(eq)_* > *(P)*‐*(Z)*‐**2** *_(eq)_* > *(M)*‐*(Z)*‐**2** *_(ax)_* (Table S3). This stability order may result from a helicity‐induced stereoelectronic effect. The C_8a_‐O_1_‐C_2_ bond angles for *(P)*‐*(Z)*‐**2** *_(ax)_*, *(M)*‐*(Z)*‐**2** *_(eq)_*, *(P)*‐*(Z)*‐**2** *_(eq)_*, and *(M)*‐*(Z)*‐**2** *_(ax)_* are 118.25°, 118.25°, 110.25°, and 109.25°, respectively. When the phenyl groups are axial in the *P* helicity (and equatorial in the *M* helicity), the O_1_ atom can adopt *sp^2^* hybridization, enabling electron donation to the adjacent aromatic ring. Conversely, when the phenyl groups are equatorial in the *P* helicity (and axial in the *M* helicity), the O_1_ atom adopts *sp^3^* hybridization, hindering electron donation to the aromatic ring. In contrast to *(Z)*‐**2**, the most stable conformation of *(E)*‐**2** is *(M,M)*‐*(E)*‐**2** *_(eq)_*, with the phenyl substituent in the equatorial position. Calculations of the alternative helicity show that *(P,P)‐(E)‐* **2** *_(ax)_*, although expected to be more stable, lies 8.2 kJ.mol^−1^ higher in energy than *(M,M)‐(E)‐* **2** *_(eq)_*, resulting in the order of stability of *(M,M)‐(E)*‐**2** *_(eq)_* > *(P,P)*‐*(E)*‐**2** *_(ax)_* > *(M,M)*‐*(E)*‐**2** *_(ax)_* > *(P,P)*‐*(E)*‐**2** *_(eq)_*. DFT calculations further revealed that the thermal conversion proceeds from *(P,P)*‐*(E)*‐**2** *_(ax)_* to *(M,M)*‐*(E)*‐**2** *_(eq)_* with barriers of 22.8 and 15.1 kJ.mol^−1^, respectively, at 25°C. In comparison, the conversion from *(M)*‐*(Z)*‐**2** *_(eq)_* to *(P)‐(Z)*‐**2** *_(ax)_* has a barrier of 25.5 kJ.mol^−1^. These results indicate that, unlike molecular rotary motors [52, 53], the *E* and *Z* isomers of compound **2** favor different helicities and substituent orientations in their most stable conformations. Therefore, in the following discussion, *(M,M)*‐*(E)*‐**2** and *(P)*‐*(Z)*‐**2** refer to their most stable conformers, *(M,M)*‐*(E)*‐**2** *_(eq)_* and *(P)*‐*(Z)*‐**2** *_(ax)_* respectively unless stated otherwise. Calculations also revealed that the most stable conformation of electrocyclization product **3** has the phenyl substituent at C_2_ in an axial orientation. However, the energy difference between the conformations with the phenyl substituent in the axial versus equatorial position is only approximately 3.3 kJ.mol^−1^ (Table S3).

**FIGURE 9:**
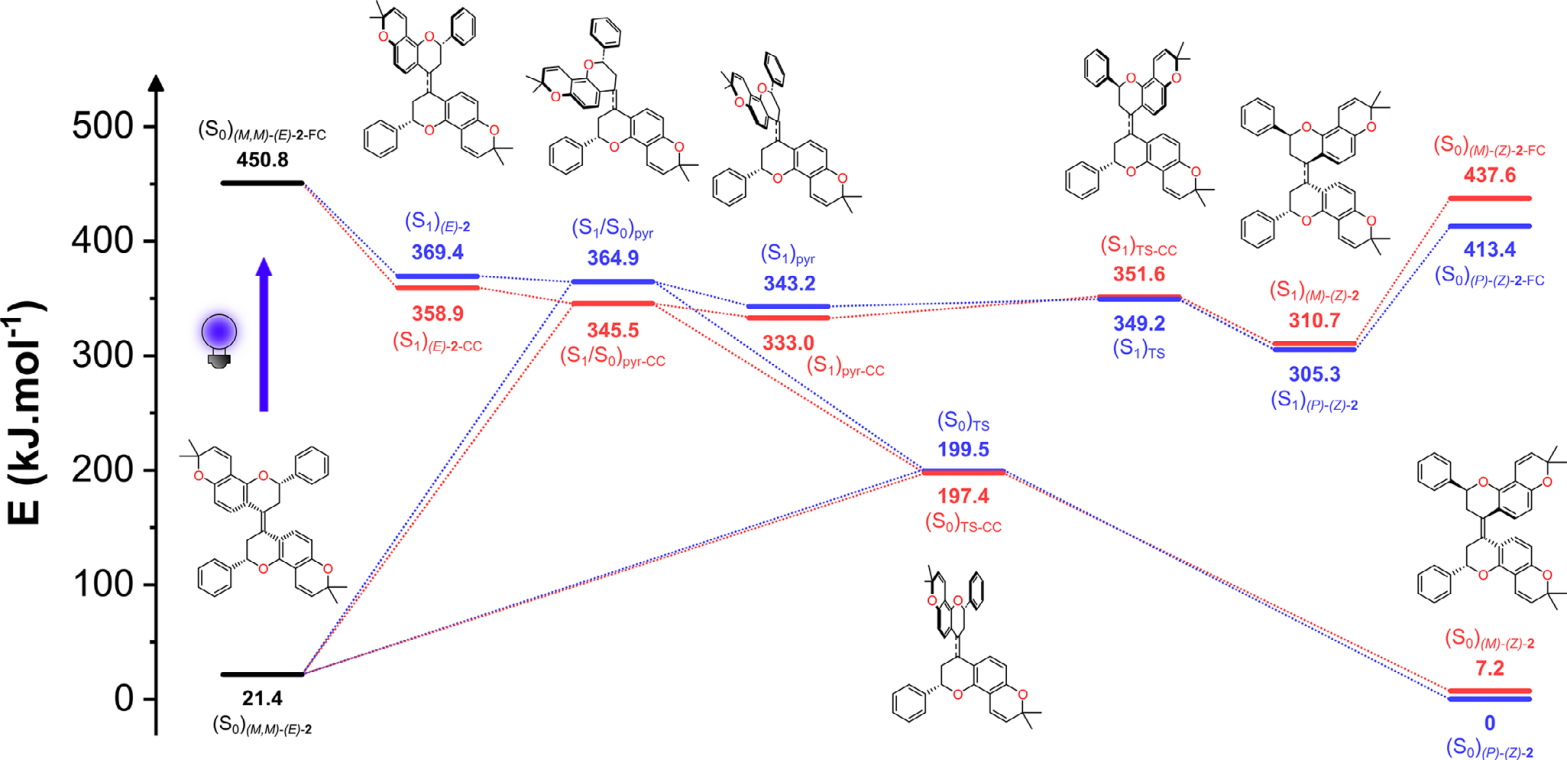
S_1_ and S_0_ electronic energies of geometries optimized at the ground state (S_0_) and first singlet excited state (S_1_) PESs along the clockwise isomerization of *(M,M)*‐*(E)*‐**2** (blue), counterclockwise isomerization of *(M,M)*‐*(E)*‐**2** (red). For clarity, only structures along the *(M,M)*‐*(E)*‐**2** CCR isomerization are shown. Calculations were done at the SF‐TD‐BHandHLYP/cc‐pVDZ level of theory.

A summary of the ground state (S_0_) and first singlet excited state (S_1_) energies for critical points along the *(E/Z)* isomerization and 6π‐electrocyclization pathways is shown in Figure 9. Geometries labelled with “(S_0_)” and “(S_1_)” were optimized at their respective PES. Upon photon absorption, the ground state *(E)*‐**2** ((S_0_)*_(E)_*_‐_ **_2_**, *θ* = 171.9°) undergoes vertical excitation to the (S_1_) ππ*‐state at the same geometry, referred to as the Franck‐Condon point ((S_0_)*_(E)_*_‐_ **_2_**_‐FC_). Vibrational relaxation may follow, leading to two possible excited state minima, either (S_1_)*_(E)_*_‐_ **_2_** (*θ* = 136.8°) or (S_1_)*_(E)_*_‐_ **_2_**_‐CC_ (*θ* = 127.4°) along the CR and the CCR pathways, respectively. As the twisting and pyramidalization continue, the system approaches CIs where nonradiative transitions (internal conversion) from the S_1_ to the ground state (S_0_) occur. For the CR pathway, this transition happens at (S_1_/S_0_)_pyr_ (*θ* = 116.5°, *ϕ*  = 116.3°), while for the CCR pathway, it occurs at (S_1_/S_0_)_pyr‐CC_ (*θ* = 114.5°, *ϕ*  = 104.8°). From these CIs, the system can relax to either ground state *(P)*‐*(Z)*‐**2** or *(M)*‐*(Z)*‐**2** for the CR and CCR pathways, respectively, or return to the ground state *(E)*‐**2**. Inversely, if excitation begins from *(P)*‐*(Z)*‐**2**, the molecule moves from the Franck‐Condon point to a minimum (S_1_)*_(P)_*_‐_ *_(Z)_*_‐_ **_2_** (*θ* = 20.3°), through a saddle point (S_1_)_TS_ (*θ* = 109.5°), and to another minimum (S_1_)_pyr_ (*θ* = 109.5°), before reaching the ground state PES via the (S_1_/S_0_)_pyr_ conical intersection. From there, the system may evolve back to the ground state *(M,M)*‐*(E)*‐**2** or return to *(P)*‐*(Z)*‐**2**. Similarly, for *(M)*‐*(Z)*‐**2**, a comparable process occurs, with transitions through the critical points (S_1_)*_(M)_*_‐_ *_(Z)_*_‐_ **_2_**, (S_1_)_TS‐CC_, (S_1_)_pyr‐CC_, and (S_1_/S_0_)_pyr‐CC_ in sequence before returning to *(M,M)*‐*(E)*‐**2** or *(M)*‐*(Z)*‐**2**.

**FIGURE 10:**
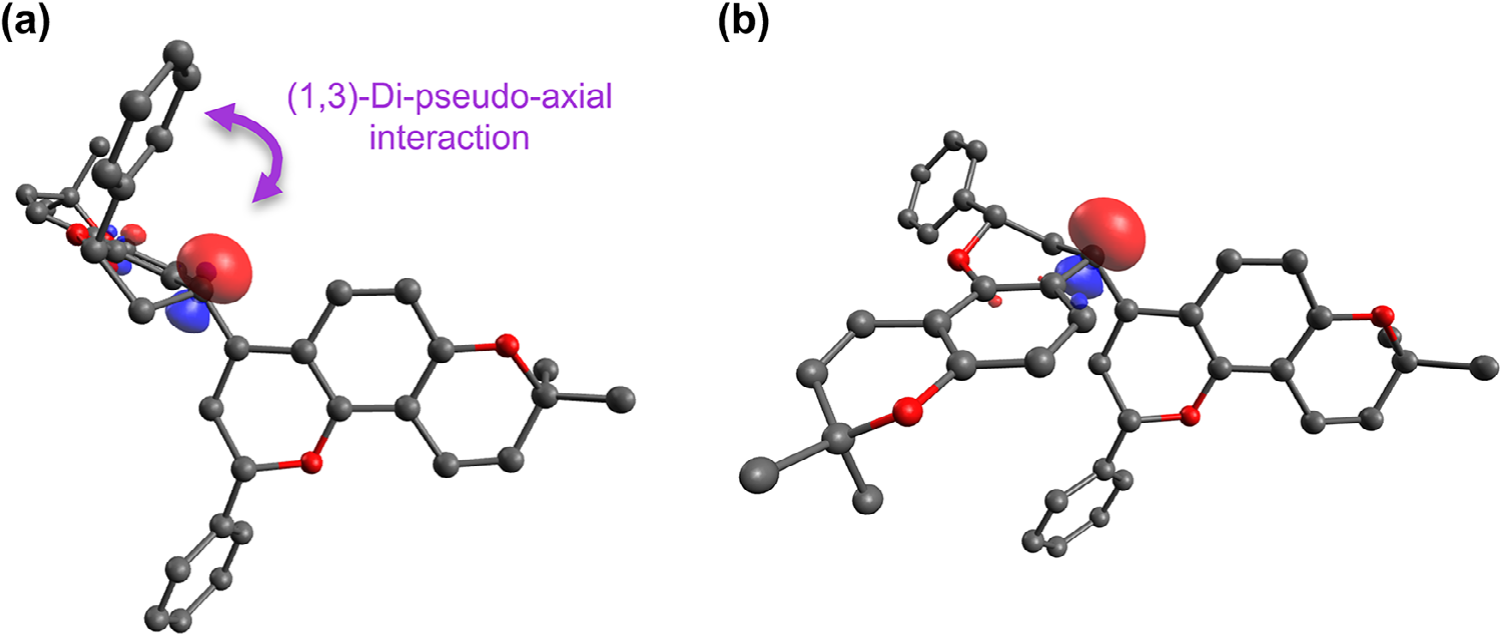
Highest occupied spatial natural orbitals of (a) (S_1_/S_0_)_pyr_ and (b) (S_1_/S_0_)_pyr‐CC_. Hydrogen atoms are omitted for clarity.

Visualization of spatial natural orbitals was performed to explain the energetic inequivalence between the (S_1_/S_0_)_pyr_ and (S_1_/S_0_)_pyr‐CC_ CIs. The pyrane‐derivative ring of *(E)*‐**2**, which includes the stereogenic centers, adopts a twisted conformation, allowing the phenyl groups at the C_2_ position to occupy a pseudo‐equatorial position in (S_1_/S_0_)_pyr‐CC_ and a pseudo‐axial position in (S_1_/S_0_)_pyr_. In the latter case, the bulky phenyl substituent experiences a 1,3‐di‐pseudo‐axial interaction with the electron density of the pyramidalized C_4_. This interaction is absent, or replaced by a weaker interaction (between H_2_ *_(ax)_* and the electron density of C_4_), in (S_1_/S_0_)_pyr‐CC_. A conformer of (S_1_/S_0_)_pyr_ with an equatorial phenyl substituent is even higher in energy by 5.4 kJ.mol^−1^ (Table S3) due to a 1,2‐di‐pseudo‐axial interaction between H_3_ *_(ax)_* and the C_4_ electron density. This aligns with the visualization of the highest occupied spatial natural orbitals in the lowest singlet state of both (S_1_/S_0_)_pyr_ and (S_1_/S_0_)_pyr‐CC_ (Figure 10). The pyramidalization of one of the carbon atoms at the conical intersection geometry is typical for alkenes where the ground state transition state is more stable in the diradical state rather than the zwitterionic state [54]. This observation remains true in our system for the (S_0_)_TS_‐(S_0_)_TS‐CC_ pair and the (S_1_/S_0_)_pyr_‐(S_1_/S_0_)_pyr‐CC_ pair, which exhibit diradical and zwitterionic character, respectively, due to the symmetrical dimeric structure.

**FIGURE 11:**
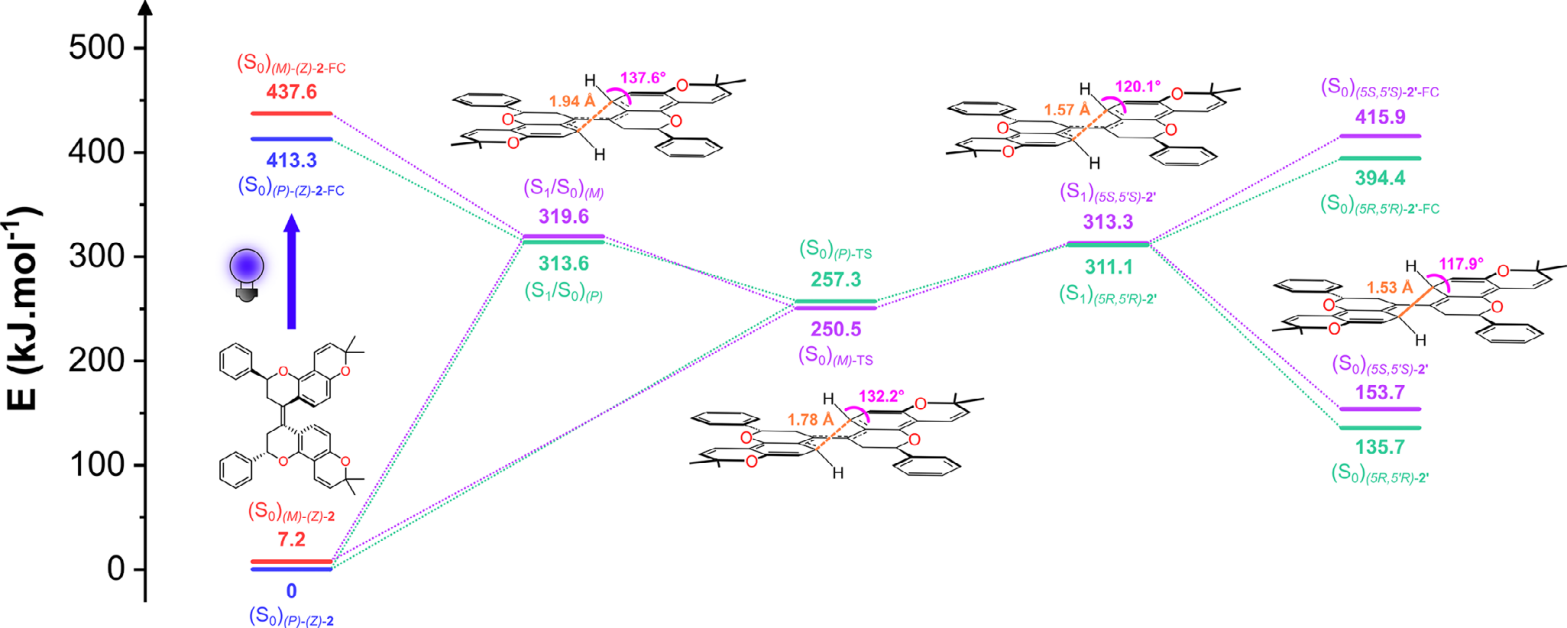
S_1_ and S_0_ electronic energies of geometries optimized at the ground state (S_0_) and first singlet excited state (S_1_) PESs along the photocyclization of *(P)*‐*(Z)*‐**2** (green) and photocyclization of *(M)*‐*(Z)*‐**2** (purple) reaction coordinates calculated at the SF‐BHandHLYP/cc‐pVDZ level of theory. For clarity, only structures along the *(M)*‐*(Z)*‐**2** cyclization pathways are shown. Calculations were done at the SF‐TD‐BHandHLYP/cc‐pVDZ level of theory.

Both *(P)*‐*(Z)*‐**2** (*r* = 3.00 Å, *ϕ* = 178.5°) and *(M)*‐*(Z)*‐**2** (*r* = 3.19 Å, *ϕ* = 178.9°) can proceed along the cyclization pathway upon excitation (Figure 11). After photoirradiation, vibrational relaxation guides the excited molecules toward their respective CIs, with (S_1_/S_0_)*_(P)_*_‐_ *_(Z)_*_‐_ **_2_** having *r* = 1.94 Å and *ϕ* = 146.4°, and (S_1_/S_0_)*_(M)_*_‐_ *_(Z)_*_‐_ **_2_** having *r* = 1.94 Å and *ϕ* = 137.6°. These CIs enable nonradiative internal conversion to the ground state PES, followed by a continued energy descent toward the final ground‐state products: *(5R,5’R)*‐**2'** (*r* = 1.53 Å and *ϕ* = 117.1°) for the *(P)*‐*(Z)*‐**2** substrate, and *(5S,5’S)*‐**2'** (*r* = 1.53 Å and *ϕ* = 117.0°) for the *(M)*‐*(Z)*‐**2** substrate. Alternatively, the excited **2'** intermediate may decay to its excited‐state minimum, either (S_1_)*_(5R,5’R)_*_‐_ **_2'_** or (S_1_)*_(5S,5’S)_*_‐_ **_2'_**, before progressing to the CIs. From these points, the system can either relax back to the ground state *(Z)*‐**2** or return to the ground state as **2'**. The structures of all critical points mentioned in this discussion are presented in the Supporting Information.

Although only small energy differences were observed between the minima or saddle points of the CR pathway and their analogues in the CCR pathway, the 19.2 kJ.mol^−1^ electronic energy difference between the two CIs due to the presence of stereocenters is intriguing. This is particularly notable because these stereocenters are located far from the Fjord region [52], a position that is typically left unsubstituted in overcrowded alkenes. However, our recent studies shows that a substituent in the β‐position relative to the olefin can influence the directionality of the *(E*/*Z)*‐photoisomerization [6]. This suggests that substituent modulation at this position in overcrowded alkenes could serve as a strategy to tune CI energetics, potentially controlling the nonradiative decay rate and even introducing unidirectionality into the PESs around the CIs. Further exploration of this molecular system could be valuable to demonstrate how β‐substituted bio‐based starting materials may be transformed into functional molecular photoswitch‐based systems. Moreover, because phenanthrene **3** exhibits notable fluorescence activity from its rigid structure, related bio‐based phenanthrene systems may provide useful platforms for the future development of multifunctional photoresponsive materials. In this context, further optimization could support the design of functional chromophores while maintaining the interest of using renewable molecular building blocks.

## 3 Conclusion

This study demonstrates a sustainable strategy to design a light‐driven overcrowded alkene with molecular photoswitch‐like behavior using an enantiopure, bio‐derived flavanone as a starting material. By utilizing *(‐)*‐isolonchocarpin **1** which was isolated from *Tephrosia vogelii*, we achieved a facile synthesis involving McMurry homocoupling, photoisomerization, and oxidative photocyclization, yielding a structurally complex photoresponsive system. The resulting overcrowded alkene **2** undergoes directional *E*/*Z*‐photoisomerization, showcasing its potential as a building block for further design of novel molecular photoswitches. Computational studies revealed that the intrinsic chirality of the natural starting material plays a crucial role in breaking symmetry and biasing rotational pathways, further enhancing control over molecular motion. This work not only highlights the viability of bio‐based compounds, such as phenanthrene **3**, in advanced photochemical applications but also provides new insights into the design of sustainable and chiroptical molecular systems for future applications in dynamic materials, molecular machines, and optoelectronics.

## Conflicts of Interest

The author declare no conflicts of interest.

## Supporting information




**Supporting File**: chem71180‐sup‐0001‐SuppMat.pdf.

## Data Availability

The data that supports the findings of this study are available in the supplementary material of this article
