# Supplementary material for: A Bio‐Based Chiroptical Overcrowded Alkene From Flavanone: Photoreactivity and Theoretical Investigation
Source: Chemistry. 2026 May 29;32(34):e71180. doi: 10.1002/chem.71180 (PMC13569967; doi:10.1002/chem.71180)
Supplement: Supplementary file 1 [file CHEM-32-e71180-s001.pdf]

Supporting Information

A Bio-Based Chiroptical Overcrowded Alkene from Flavanone: Photoreactivity and Theoretical Investigation

Deryl H. Limawan[a, b], Adrien Combe[b], Anthony Bongso[a, b], Bayu Dwiputra[a], Atthar L. Ivansyah[c], Roza R. Weber[b], Charlotte N. Stindt[b], Robby Roswanda[a]*, Ben L. Feringa[b]*

[a] Organic Chemistry Division, Department of Chemistry, Faculty of Mathematics and Natural Sciences, Institut Teknologi Bandung, Bandung 40132, Indonesia

[b] Stratingh Institute for Chemistry, Faculty of Science and Engineering, University of Groningen, 9747AG Groningen, The Netherlands

[c] Inorganic and Physical Chemistry Division, Department of Chemistry, Faculty of Mathematics and Natural Sciences, Institut Teknologi Bandung, Bandung 40132, Indonesia

*r.roswanda@itb.ac.id, *b.l.feringa@rug.nl

S0. Experimental Section……2

S1. Characterization of Isolonchocarpin (**1**)……4

S2. Characterization of (*M,M*)-(*E*)-**2**……8

S3. Characterization of (*P*)-(*Z*)-**2**……18

S4. Characterization of **3**……24

S5. Computational Study……27

S0. Experimental Section

General. All chemicals were purchased from commercial suppliers (Sigma Aldrich, Honeywell, Thermoscientific, TCI Europe N.V., and Acros). Technical grade solvents for extraction and chromatography were purchased from Honeywell and Interchem. Analytical grade solvents were purchased from Boom, Macron, J.T. Baker, and Biochimie SARL. Dry solvents were used from the solvent purification system using an MBraun SPS-800 column. All reactions were monitored by Thin Layer Chromatography (TLC) on Merck silica gel 60 M / Kieselguhr F_{254} TLC plates and visualized by exposure to UV-light (254 nm and 365 nm) and staining with potassium permanganate solution. Flash column chromatography was performed on silica gel (Biotage Sfär Silica High Capacity Duo). Manual column chromatography was performed on silica gel (Silica 60, 230-400 Mesh, particle size 0.04-0.063 mm, code 1.09385.1000) manufactured from Macherey-Nagel. Deuterated solvents were purchased from Sigma Aldrich. Nuclear magnetic resonance (NMR) spectra were recorded at 25 °C on Bruker Avance 600 (^{1}H: 600 MHz, ^{13}C: 151 MHz) and Variant Unity INOVA-500 MHz Agilent Technologies (^{1}H: 500 MHz, ^{13}C: 126 MHz). Before use, the deuterated solvent $CDCl_3$ was treated with anhydrous K_2CO_3 and $MgSO_4$. Chemical shifts (δ) are expressed relative to the resonances of the residual non-deuterated solvent for ^{1}H ($CDCl_3$: δ = 7.26 ppm) and ^{13}C ($CDCl_3$: δ = 77.16 ppm). Absolute values of the coupling constants are given in Hertz (Hz). Multiplicities are abbreviated as singlet (s), doublet (d), doublet of doublets (dd), triplet (t), doublet of triplets (dt) and multiplet (m). Infrared (IR) spectrometry was carried out with Spectrum Two FT-IR Spectrometer (PerkinElmer). Signal types are indicated as strong (s), medium (m) and weak (w). High-resolution mass spectrometry (HRMS) was performed with electrospray ionization (ESI) on a LTQ Orbitrap XL spectrometer for compounds **1** and **2**, and a Waters LCT Premier XE Mass Spectrometer for compound **3**. LC-MS analysis was performed on a Thermo Scientific Vanquish UHPLC with Acquity UPLC, and electronic ion-spray with positive mode. The eluent was MeCN at HPLC grade. UV-Vis absorption spectra were recorded on Agilent Technologies Cory 8454 UV-Vis and Agilent Technologies Cary 60 UV-Vis. CD spectra were recorded on JASCO J-810 and JASCO J-1500 spectrophotometer. UV-Vis and CD spectra were taken in 1 cm path length quartz cuvettes and were recorded at 25 °C. Irradiation was performed using Thorlabs INC OSL 1-EC fiber illuminator (365 nm, 395 nm and 405 nm) and Taffled Ultraviolet (395 nm) with the distance between LED and sample at 3 cm. The absolute fluorescence quantum yield was determined using an Edinburgh Instruments FS5 spectrofluorometer with the SC-30: Integrating Sphere module and the Edinburgh Instruments Fluoracle® software.

Plant material. The seeds of the *Tephrosia vogelii* plant were taken from Mount Burangrang, which is on the border of West Bandung and Purwakarta districts, West Java.

Extraction and isolation of isolonchocarpin (1).
The dried powdered pod peels (6 kg) of *Tephrosia vogelii* were extracted at room temperature with acetone (4 L). The solvent was evaporated under reduced pressure, resulting in a two-phases residue. The lower phase was separated and suspended in *n*-hexane followed by extraction by methanol to separate the target compounds from fatty acids. The methanol phase was extracted and subjected to vacuum liquid chromatography[55] (SiO_2, *n*-Hex:EtOAc from 10:0 to 0:10) resulting in a total of sixteen fractions. Subsequently, fractions two up to seven were purified again through the same method to obtain flavanone **1** as a white powder. The compound has been recrystallized in *n*-heptane to give white crystals (1.120 g, 3.659 mmol).; ^{1}H NMR (600 MHz, $CDCl_3$): δ = 7.75 (d, *J* = 8.7 Hz, 1H), 7.50–7.47 (m, 2H), 7.46–7.42 (m, 2H), 7.41–7.37 (m, 1H), 6.66 (dd, *J* = 0.7, 10.0 Hz, 1H), 6.51 (dd, *J* = 0.7, 8.7 Hz, 1H), 5.58 (d, *J* = 10.0 Hz, 1H), 5.48 (dd, *J* = 2.9, 13.3 Hz, 1H), 3.01 (dd, *J* = 13.3, 16.8 Hz, 1H), 2.85 (dd, *J* = 3.0, 16.8 Hz, 1H), 1.47 (s, 3H), 1.45 (s, 3H) ppm; ^{13}C NMR (151 MHz, $CDCl_3$): δ = 190.7, 159.8, 157.8, 139.2, 129.0, 128.9, 128.7, 128.1, 126.1, 116.0, 114.9, 111.3, 109.6, 79.9, 77.7, 44.5, 28.5, 28.2 ppm; IR (FT–IR): ν = 3038, 3009 (w, C–$H_{alkenes}$), 2984, 2972 (w, C–H_{alkane}), 1682 (s, C=O_{ketone}), 1635 (m, C=$C_{cis\text{-}alkene}$), 1593, 1577 (s, C=$C_{aromatics}$) cm^{-1}; HRMS (ESI pos.): calculated for $C_{20}H_{19}O_3$ ([M + H]$^+$): 307.1329, found 307.1329.

Synthesis of compound 2.
Under a nitrogen atmosphere, in a 50 mL two-neck round-bottom flask, zinc powder (173 mg, 2.64 mmol, 8.0 eq.) was suspended in anhydrous tetrahydrofuran (2.0 mL) before cooling down the flask to 0 °C. Then, titanium(IV) chloride (0.15 mL, 1.4 mmol, 4.0 eq.) was added carefully to the solution, and the resulting mixture was heated at 70 °C and stirred for 3 h. Then, the flask was cooled down at 0 °C before adding pyridine (0.13 mL, 1.6 mmol, 5.0 eq.) and the solution of flavanone **1** (0.1 M in anhydrous tetrahydrofuran, 100 mg, 0.326 mmol, 1.0 eq.). The resulting mixture was heated again at 70 °C and stirred during 3 h. After cooling at room temperature, the heterogeneous mixture was filtered throw a short silica plug and washed with ethyl acetate. The filtrate was washed with a saturated aqueous solution of sodium bicarbonate (2 x 10 mL), an aqueous solution of hydrochloric acid (1 M, 3 x 10 mL) and brine (10 mL). The organic layer was dried with anhydrous magnesium sulfate and concentrated under reduced pressure in order to obtain the crude product as a yellow oil. The compound was purified by flash chromatography (SiO_2, *n*-Pent:EtOAc 19:1 V:V) in order to obtain the diastereomeric mixture of overcrowded alkene **2** as a pale yellow solid (82 mg, 0.14 mmol, 86%, *E*:*Z* ratio of 5:2). ^{1}H NMR (600 MHz, $CDCl_3$): [*(M,M)-(E)*-**2**] δ = 7.48–7.32 (m, 10H), 6.95 (d, *J* = 8.5 Hz, 2H), 6.71 (d, *J* = 10.0 Hz, 2H), 6.34 (d, *J* = 8.5 Hz, 2H), 5.54 (d, *J* = 10.0 Hz, 2H), 5.14 (dd, *J* = 11.6, 1.5 Hz, 2H), 3.46 (dd, *J* = 13.0, 2.0 Hz, 2H), 2.76 (t, *J* = 11.9 Hz, 2H), 1.45 (s, 6H), 1.43 (s, 6H) ppm, [*(P)-(Z)*-**2**] δ = 7.43–7.28 (m, 12H), 6.69 (d, *J* = 10.0 Hz, 2H), 6.20 (d, *J* = 8.6 Hz, 2H), 5.52 (dd,

J = 1.3, 9.9 Hz, 2H), 5.23 (dd, J = 2.5, 11.4 Hz, 2H), 2.85 (dd, J = 2.6, 13.4 Hz, 2H), 2.51 (t, J = 12.3 Hz, 2H), 1.45 (s, 6H), 1.43 (s, 6H) ppm; ^{13}C NMR (151 MHz, $CDCl_3$): [*(M,M)*-*(E)*-**2**] δ = 153.5, 150.7, 141.3, 130.3, 129.0, 128.7, 128.2, 126.4, 125.3, 116.9, 114.8, 109.6, 108.6, 79.4, 76.3, 36.9, 28.2 ppm, [*(P)*-*(Z)*-**2**] δ = 153.5, 150.7, 141.4, 130.1, 128.7, 128.5, 128.2, 126.0, 122.8, 117.0, 114.8, 109.5, 108.7, 79.2, 76.3, 36.3, 28.3, 28.2 ppm; IR (FT-IR): [*(M,M)*-*(E)*-**2**] ν = 3063, 3035, 3008 (w, C–$H_{alkenes}$), 2987, 2966, 2922, 2898 (w, C–H_{alkane}), 1635 (m, C=$C_{cis\text{-}alkene}$), 1600, 1574 (m, C=$C_{aromatic}$) cm^{-1}, [*(P)*-*(Z)*-**2**] ν = 3059, 3024, 3009 (w, C–$H_{alkenes}$), 2975, 2967, 2920, 2908 (w, C–H_{alkane}), 1635 (m, C=$C_{cis\text{-}alkene}$), 1600, 1576 (m, C=$C_{aromatic}$) cm^{-1}; HRMS (ESI pos.): calculated for $C_{40}H_{37}O_4$ ([M + H]$^+$): 581.2710, found 581.2703 for *(M,M)*-*(E)*-**2** and 581.2709 for *(P)*-*(Z)*-**2**.
<u>Notes:</u> *After flash chromatography, (E)-**2** can be isolated by adding methanol to the mixture to make it precipitate as a white powder. (P)-(Z)-**2** can be recrystallized in methanol to afford off-white flowers-like crystals. (P)-(Z)-**2** is sensitive to ambient light in solution.*

Photoirradiation study of *(M,M)-(E)*-2.
For the UV-Vis absorption spectroscopy study, a stock solution of (*E*)-**2** (2.5×10^{-5} M) in dichloromethane was placed in quartz cuvette and irradiated at 20 °C with several LEDs (365 nm, 395 nm, and 405 nm) and the spectra were recorded every 30 s during 60 or 90 min. For the ^{1}H NMR study, (*E*)-**2** (2.0×10^{-3} M) was dissolved in C_6D_6. The solution was transferred to the NMR tube and the sample was irradiated with 395 nm light. The spectra were recorded at 25 °C every 5 min for 30 min.

Synthesis of compound 3.
Overcrowded alkene (*E*)-**2** (18.7 mg, 0.0340 mmol) was dissolved in acetone (3.0 mL, 0.011 M) in a quartz cuvette and the solution was irradiated at 25 °C with 395 nm LED light under agitation for 6 h. The resulting mixture was concentrated under reduced pressure, and the product was purified using preparative TLC (SiO_2, *n*-Hex:EtOAc 99:1 V:V) to obtain the phenanthrene **3** as yellow solid (9.4 mg, 0.016 mmol, 50%). ^{1}H NMR (500 MHz, $CDCl_3$): δ = 7.54 (d, J = 7.5 Hz, 2H), 7.51 (s, 1H), 7.43 (t, J = 7.5 Hz, 2H), 7.36 (t, J = 7.3 Hz, 1H), 6.90 (d, J = 9.8 Hz, 1H), 5.73 (d, J = 9.9 Hz, 1H), 5.19 (s, 1H), 3.33–3.24 (m, 2H), 1.51 (s, 3H), 1.47 (s, 3H) ppm; ^{13}C NMR (126 MHz, $CDCl_3$): δ = 151.9, 141.3, 131.0, 130.0, 128.7, 128.2, 120.3, 117.2, 114.6, 102.4, 77.9, 76.2, 34.2, 28.3, 27.9 ppm; IR (FT–IR): ν = 2958, 2919, 2851 (w, C–H_{alkane}), 1632 (m, C=C_{alkene}), 1618, 1602, 1575, 1565 (m, C=$C_{arom.}$) cm^{-1}; HRMS (ESI pos.): Calculated for $C_{40}H_{35}O_4$ ([M + H]$^+$): 579.2530, found 579.2535.

Single-crystal X-ray crystallography

Crystals of *(M,M)*-*(E)*-**2** and *(P)*-*(Z)*-**2** were obtained by slow recrystallisation from methanol, with *(M,M)*-*(E)*-**2** dissolved in methanol containing a minimum amount of dichloromethane, and *(P)*-*(Z)*-**2** in pure methanol. Both solutions were stored at -20 °C until crystals formed. A suitable crystal of *(P)*-*(Z)*-**2** was mounted on a cryoloop and placed in the cold nitrogen stream (100 K) of a Bruker-AXS D8 Venture diffractometer with a Cu Kα (λ = 1.54178 Å) source. Due to technical limitations, the crystal of *(M,M)*-*(E)*-**2** was measured under ambient conditions. Data collection and processing was done using the Bruker APEX4 software suite.[56] The structure was solved using SHELXT[57] and refinement performed using SHELXL[58] in the OLEX2 software package.[59] A multi-scan absorption correction was applied, based on the intensities of symmetry-related reflections measured at different angular settings (SADABS-2016/2).[60] For *(M,M)*-*(E)*-**2**, contributions from disordered solvent were removed using the PLATON/SQUEEZE routine.[61] These data can be obtained free of charge from the Cambridge Crystallographic Data Centre via www.ccdc.cam.ac.uk/data_request/cif, under accession numbers 2535142 and 2535143.

S1. Characterization of Isolonchocarpin (1)

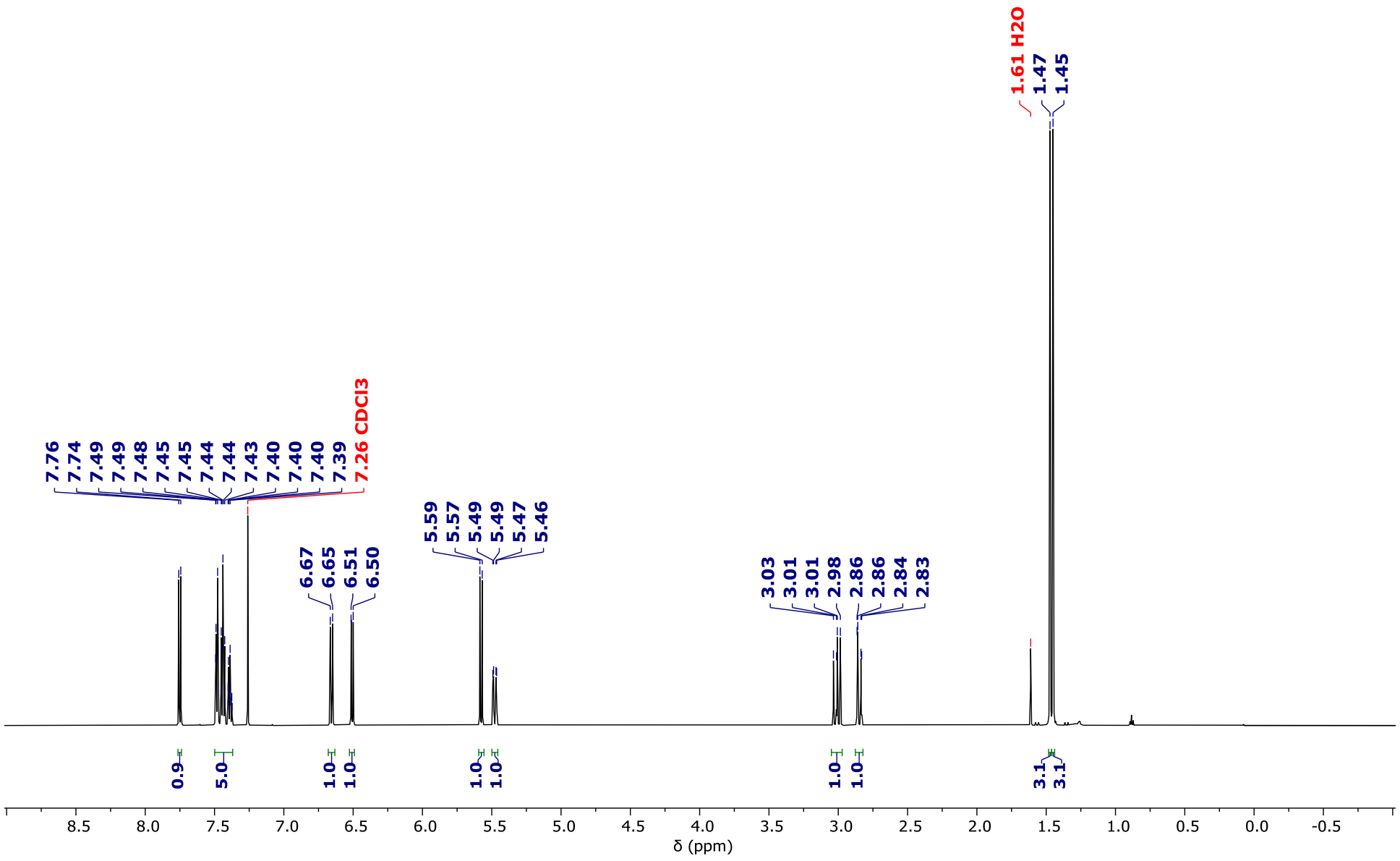

Figure S1. ^{1}H NMR spectrum of isolonchocarpin (**1**) (600 MHz, $CDCl_3$, 25 °C)

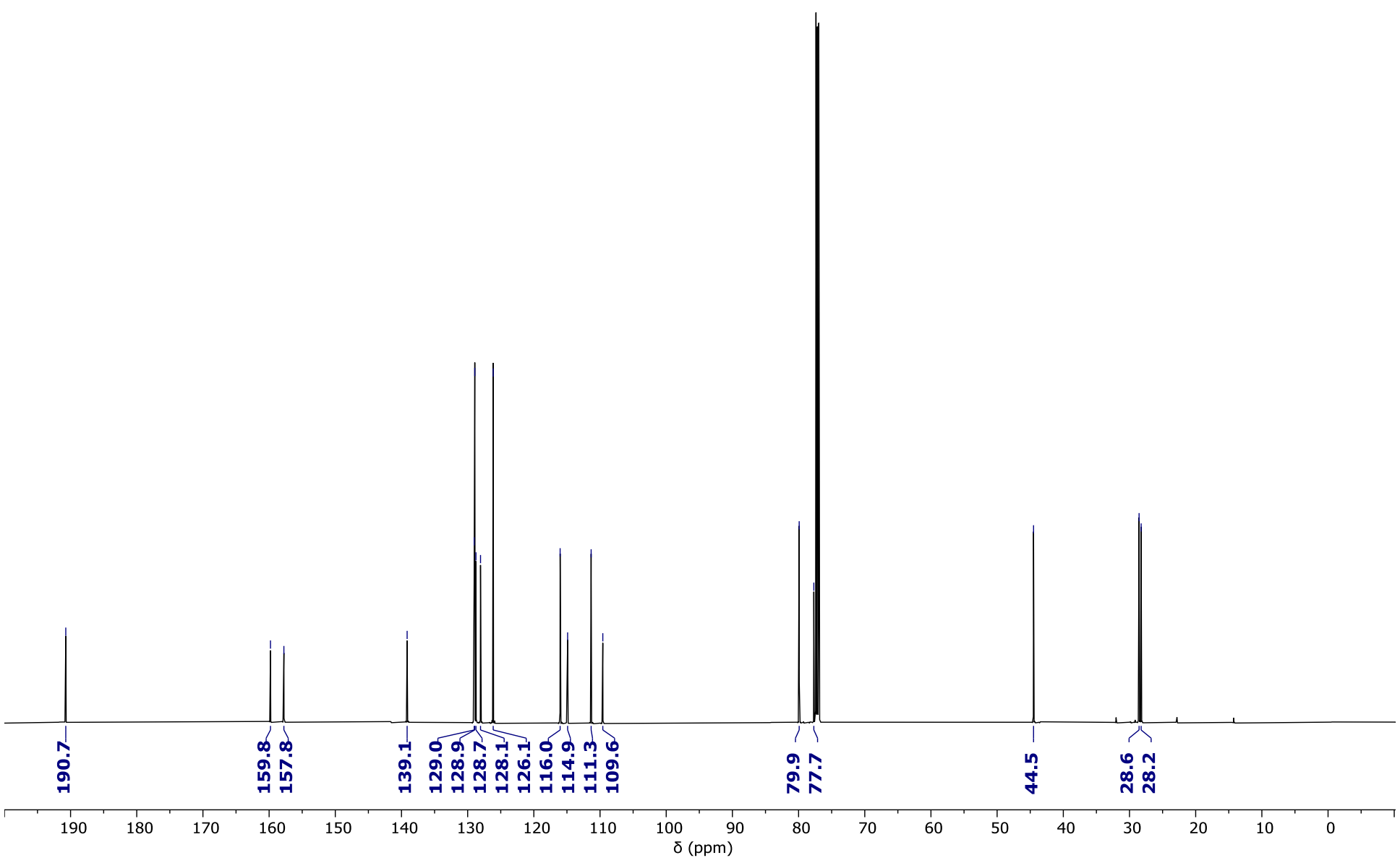

Figure S2. ^{13}C NMR spectrum of isolonchocarpin (**1**) (151 MHz, $CDCl_3$, 25 °C)

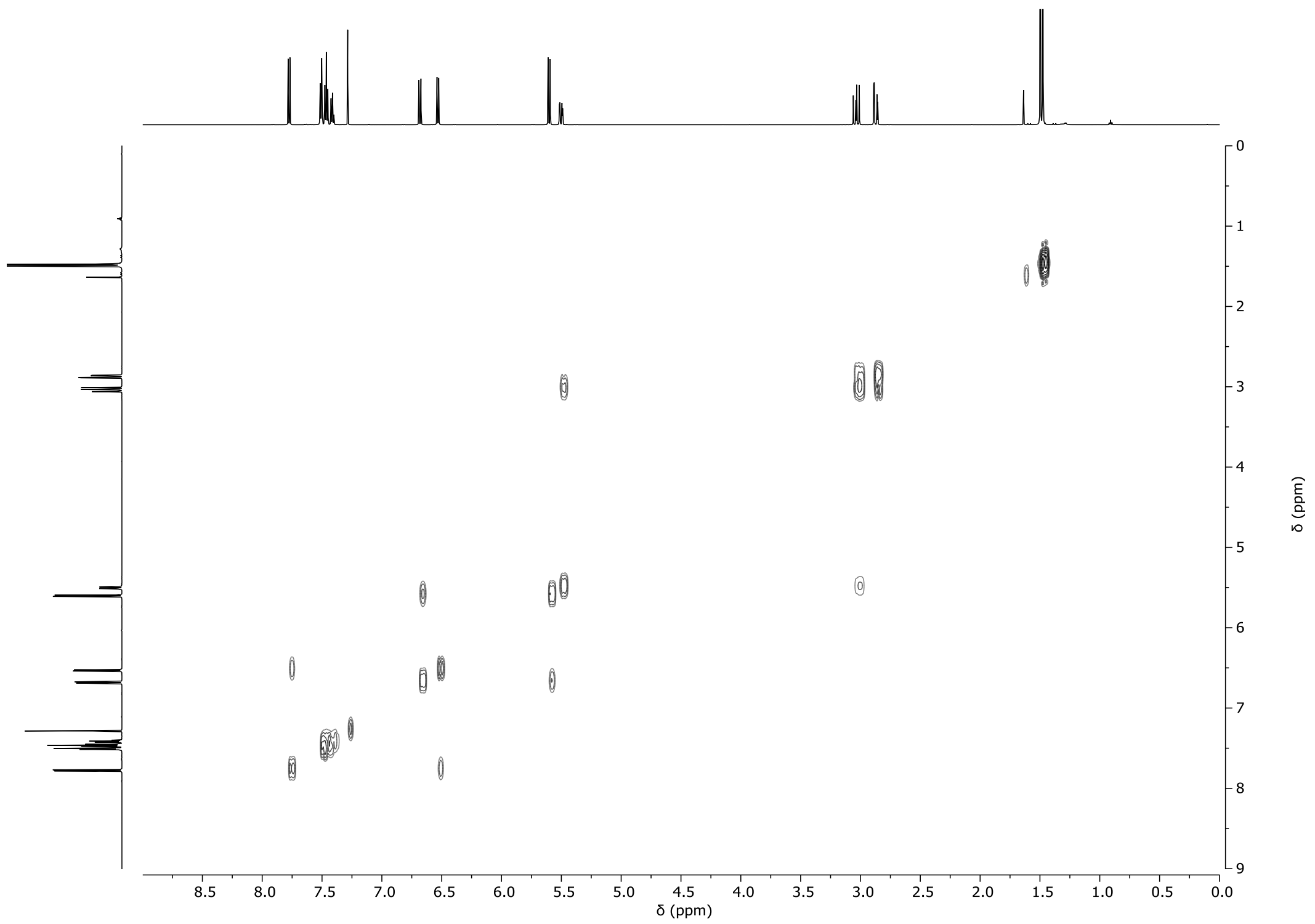

Figure S3. ^{1}H-^{1}H COSY NMR spectrum of isolonchocarpin (**1**)

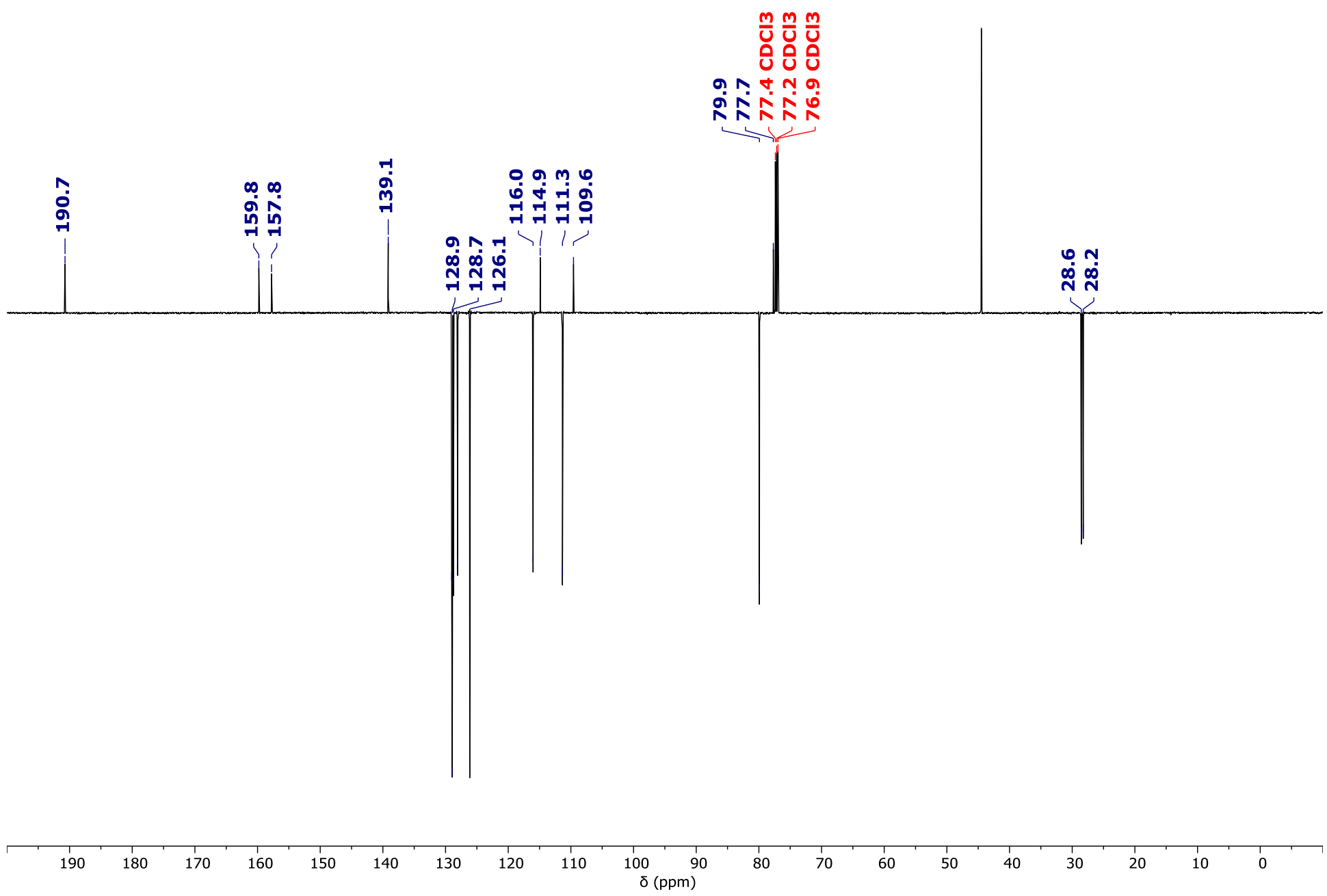

Figure S4. ^{13}C APT NMR spectrum of isolonchocarpin (**1**)

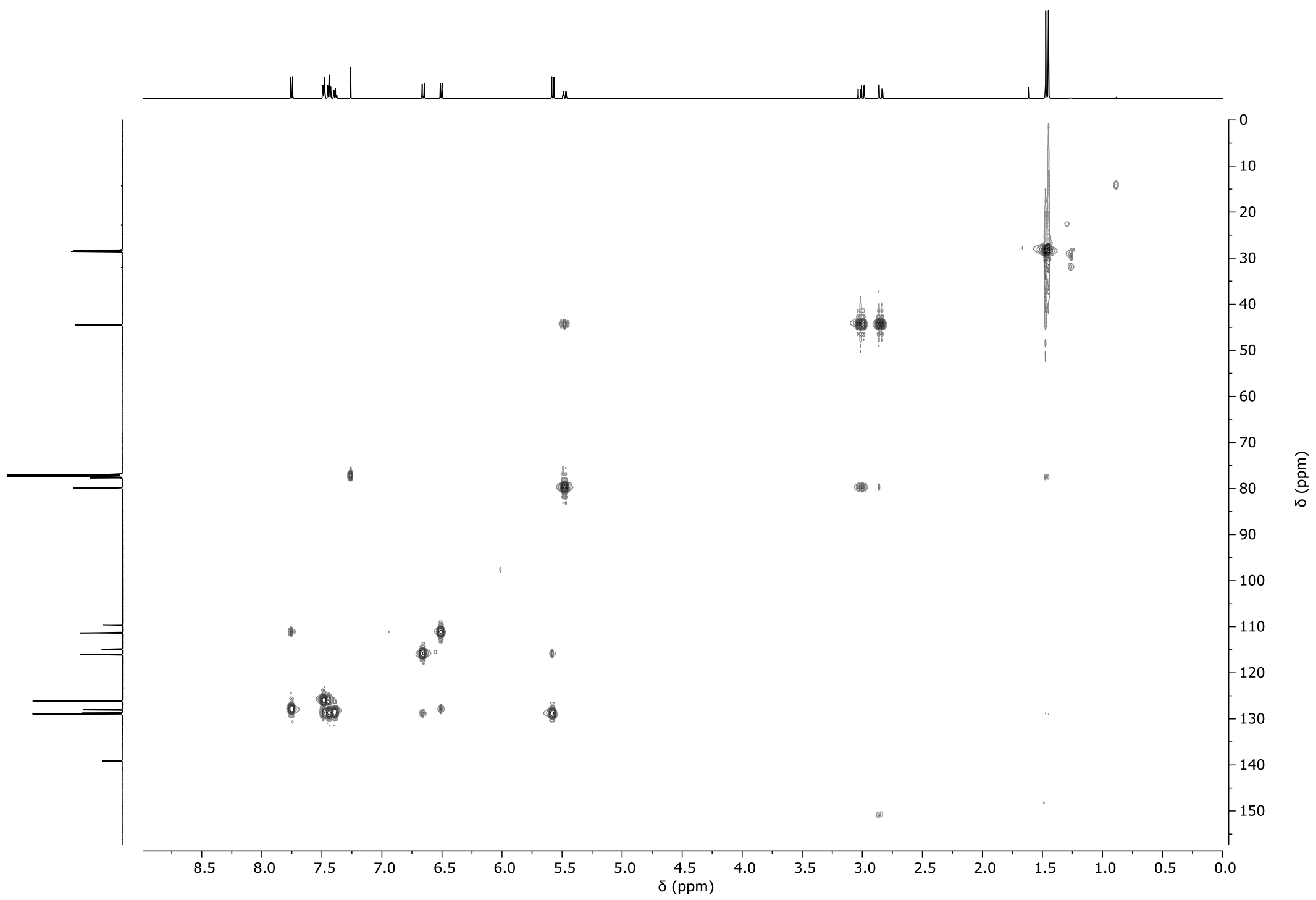

Figure S5. ^{1}H-^{13}C HSQC spectrum of isolonchocarpin (**1**)

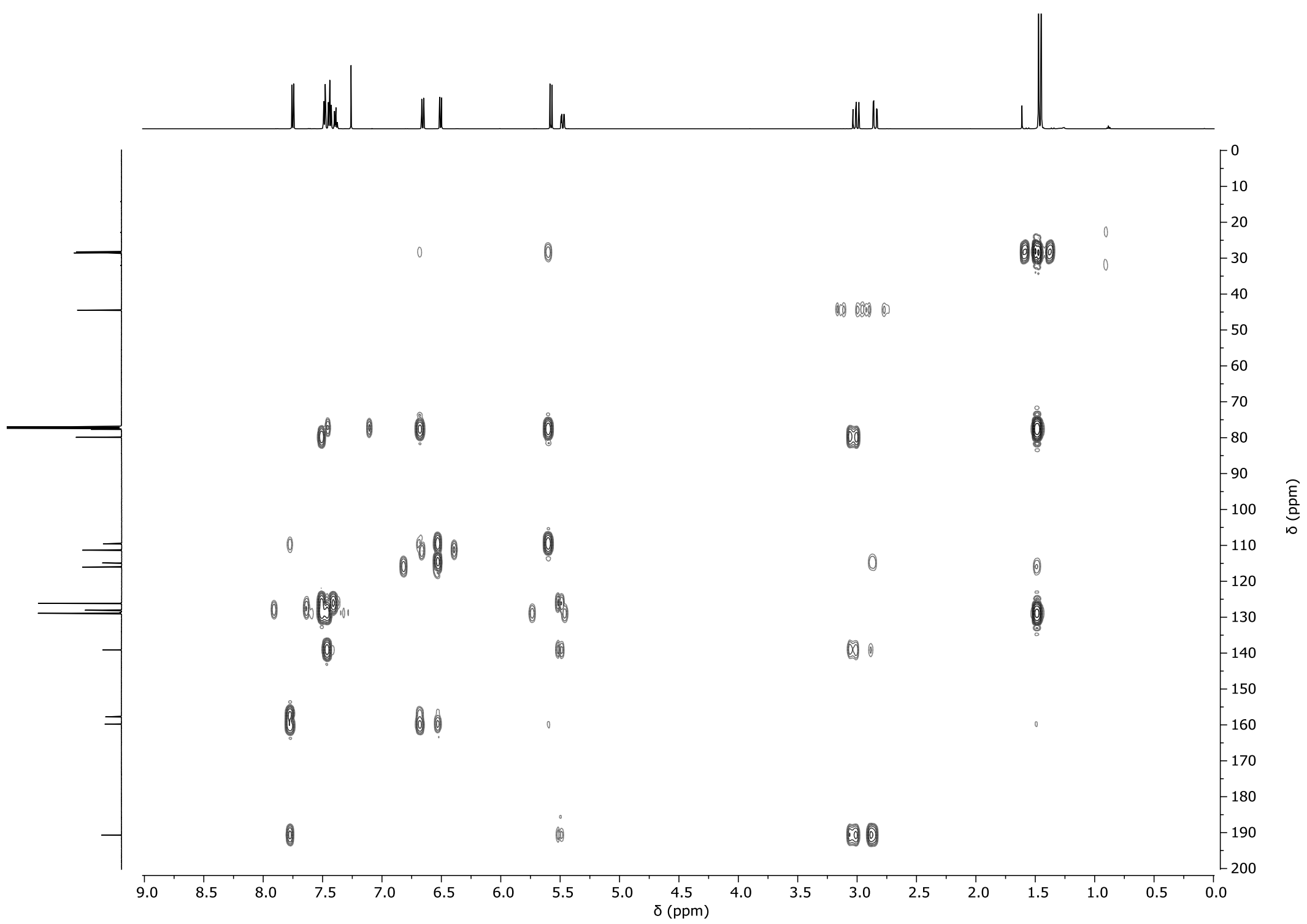

Figure S6. ^{1}H-^{13}C HMBC NMR spectrum of isolonchocarpin (**1**)

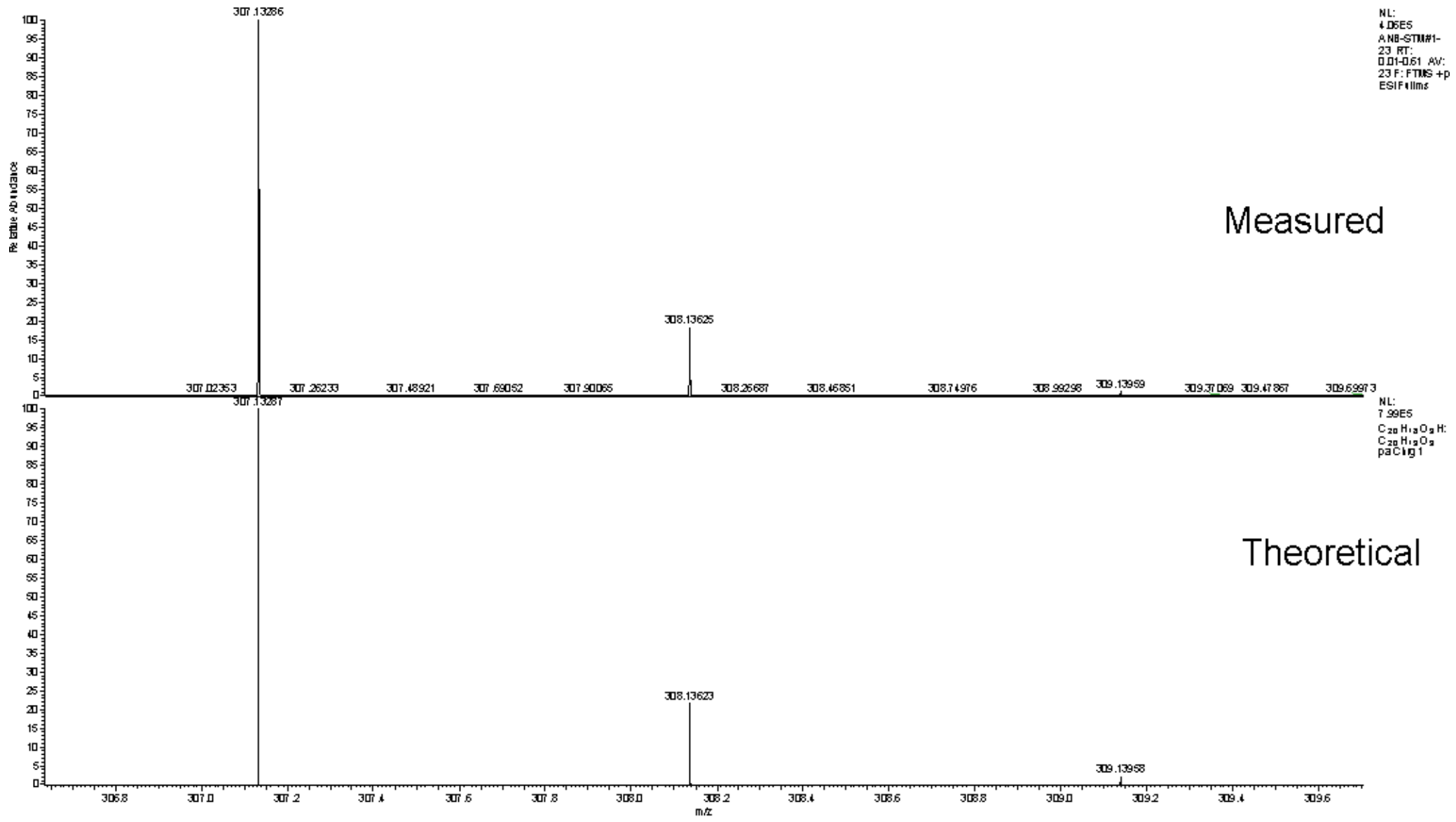

Figure S7. HRMS spectrum of isolonchocarpin (**1**)

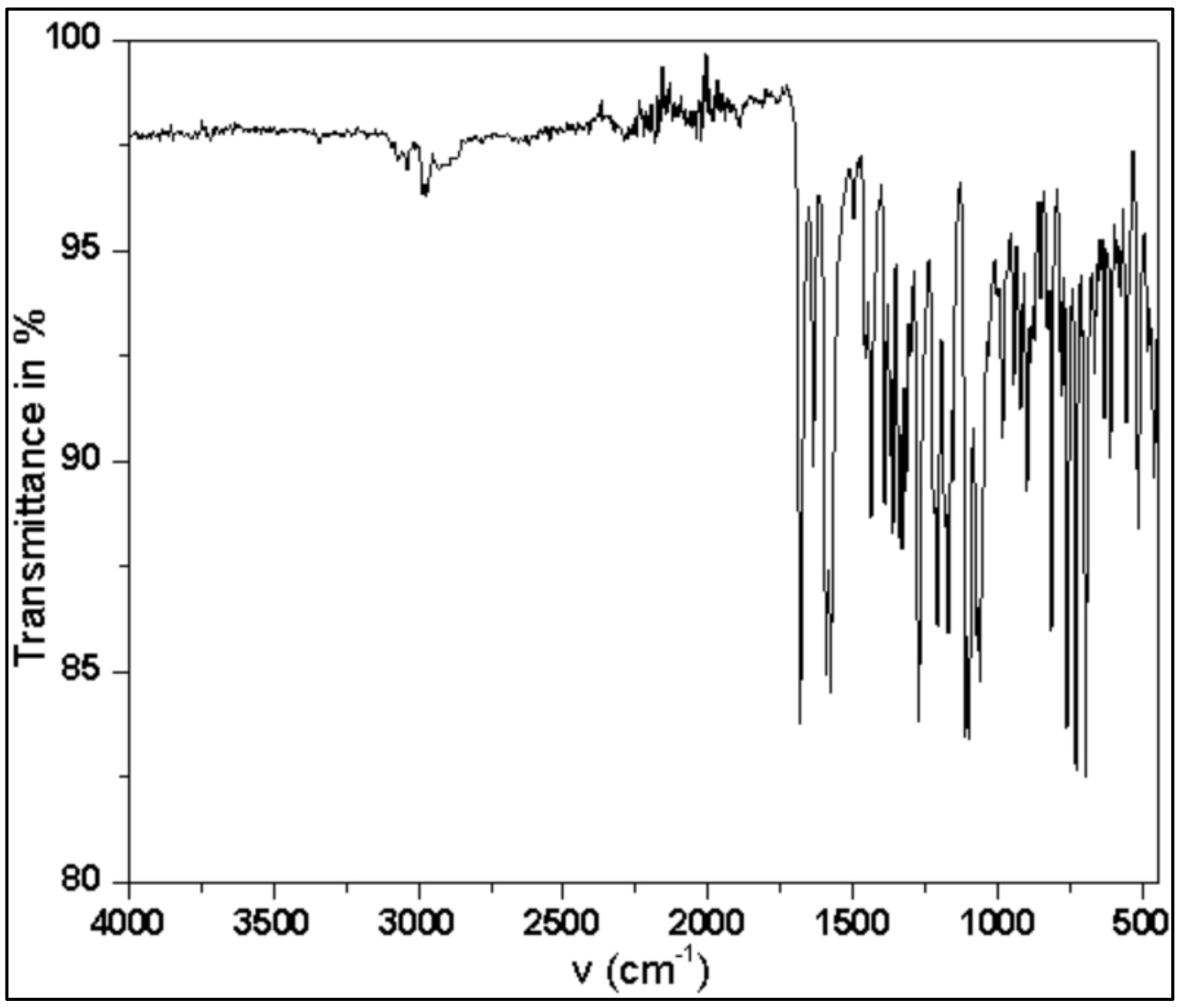

Figure S8. IR spectrum of isolonchocarpin (**1**)

S2. Characterization of *(M,M)-(E)*-2

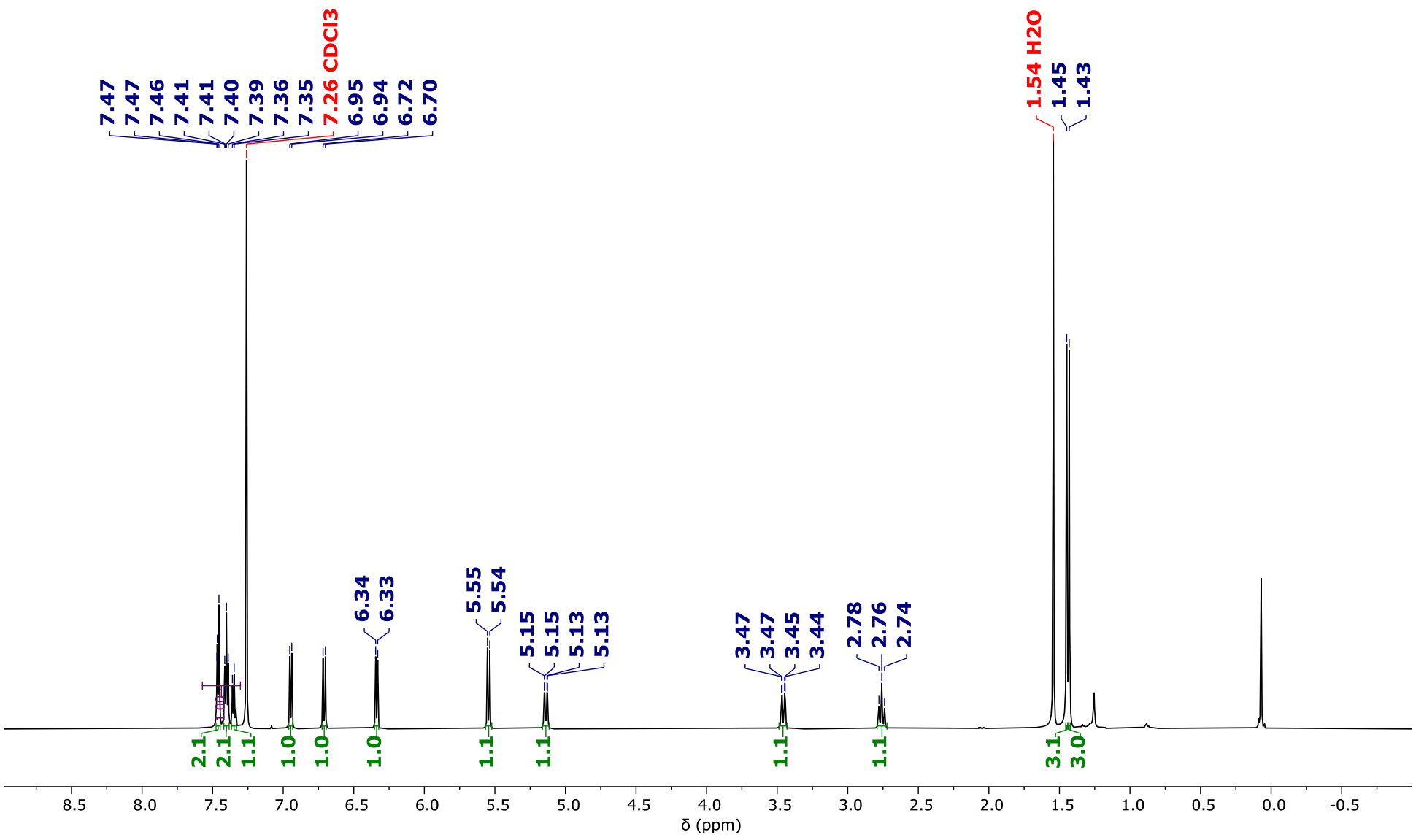

Figure S9. ^{1}H NMR spectrum of *(M,M)-(E)*-**2** (600 MHz, $CDCl_3$, 25 °C)

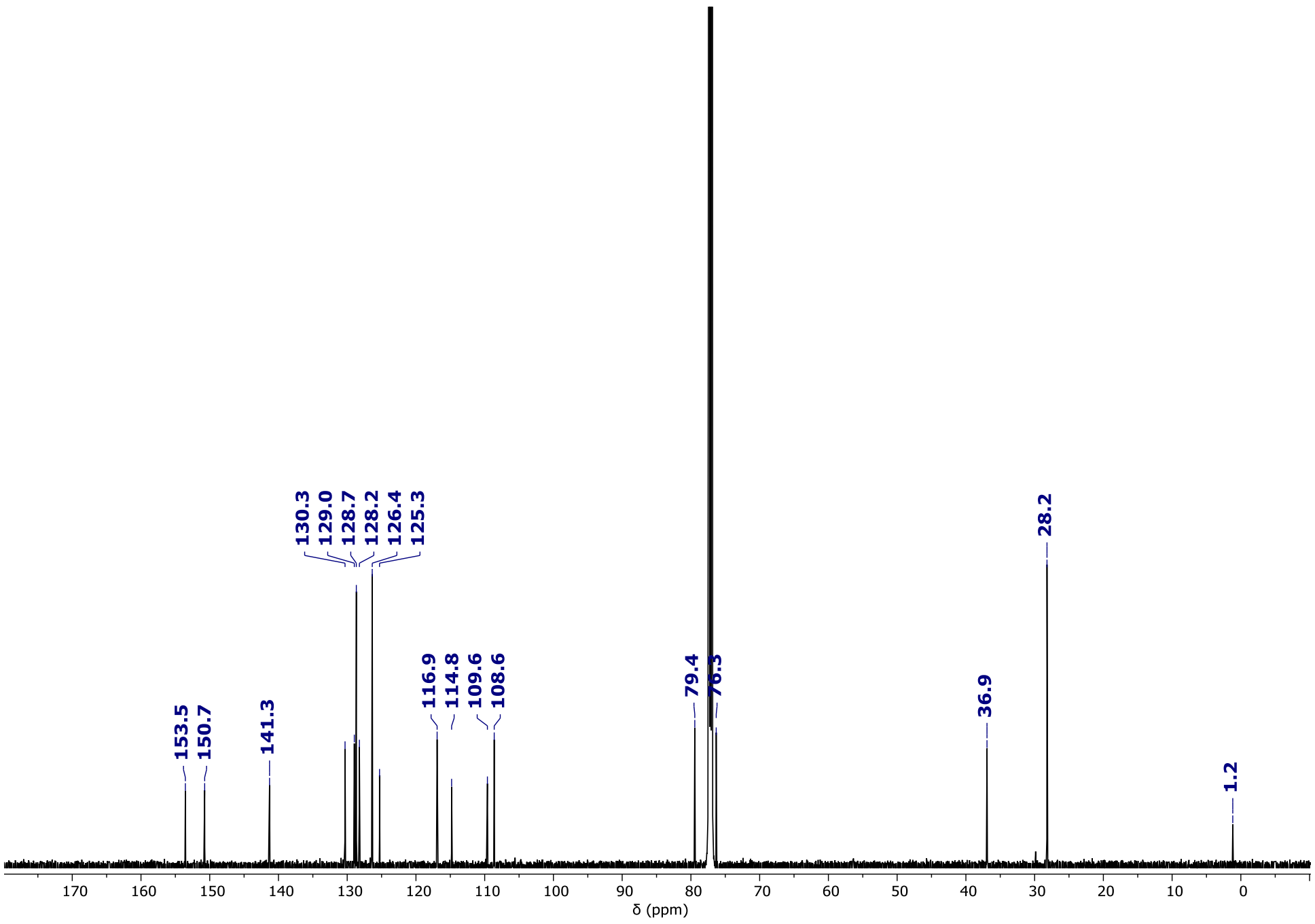

Figure S10. ^{13}C NMR spectrum of *(M,M)-(E)*-**2** (150 MHz, $CDCl_3$, 25 °C)

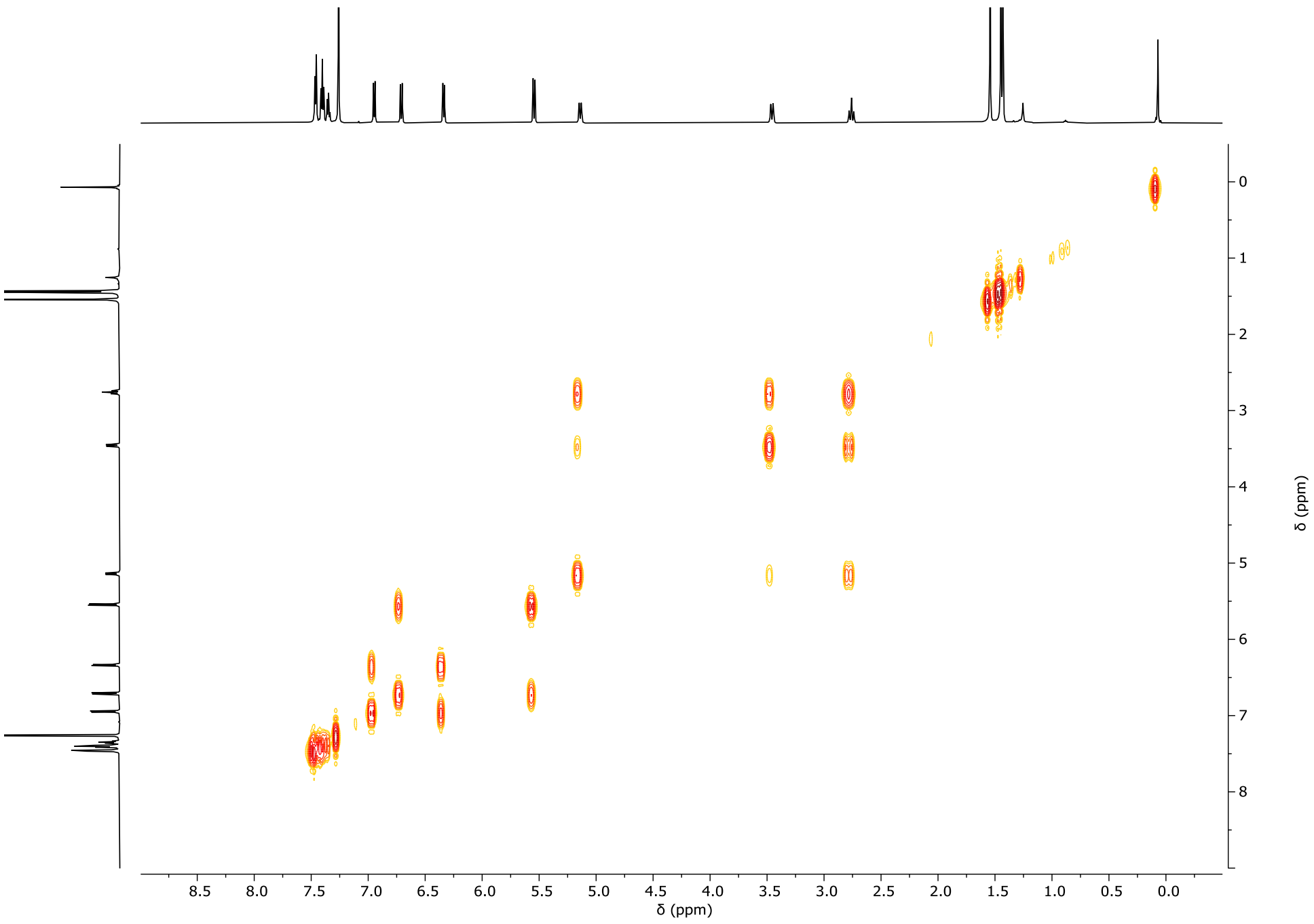

Figure S11. ^{1}H-^{1}H COSY NMR spectrum of *(M,M)-(E)*-**2**

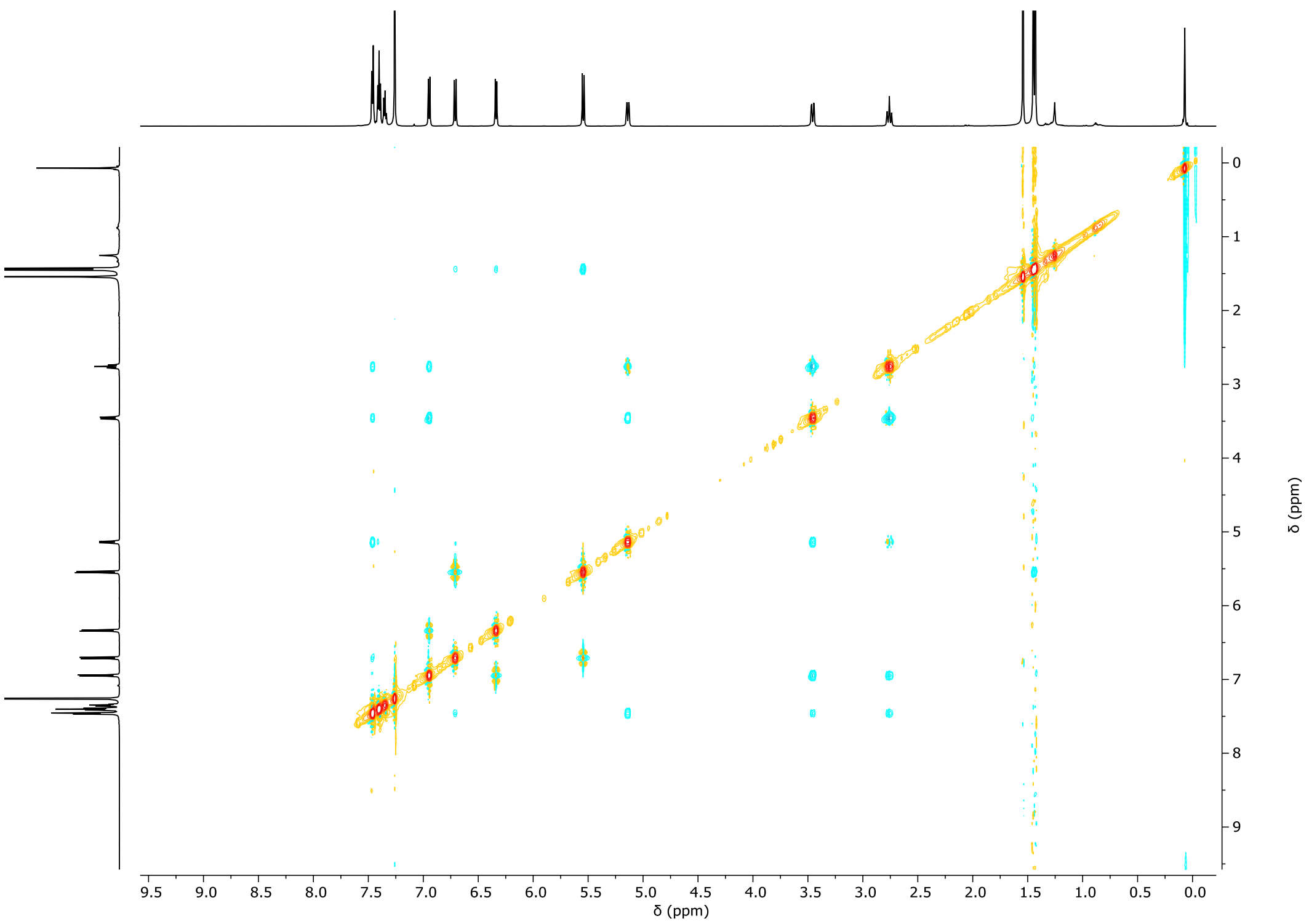

Figure S12. ^{1}H-^{1}H NOESY NMR spectrum of *(M,M)-(E)*-**2**

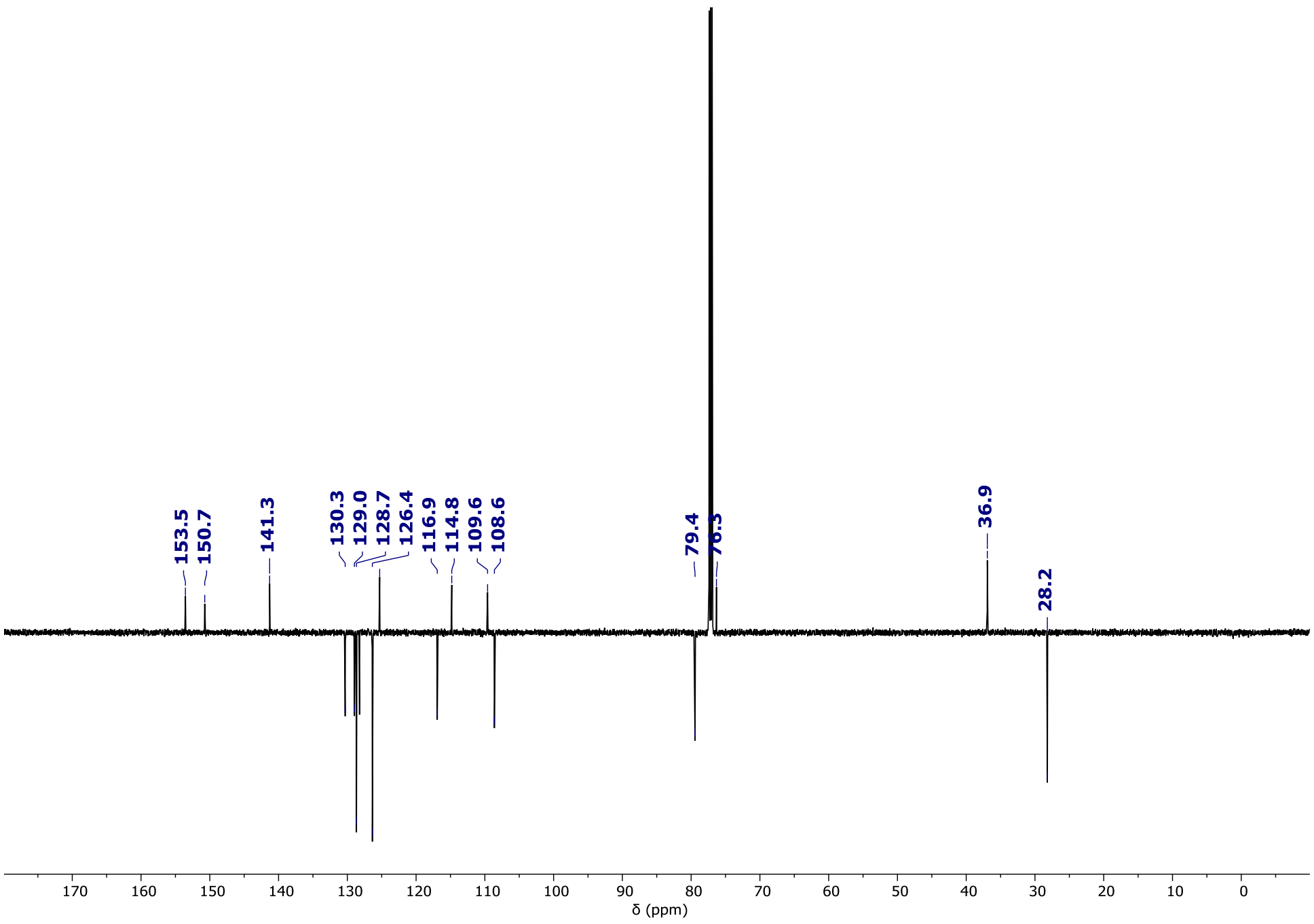

Figure S13. ^{13}C APT NMR spectrum of *(M,M)-(E)*-**2**

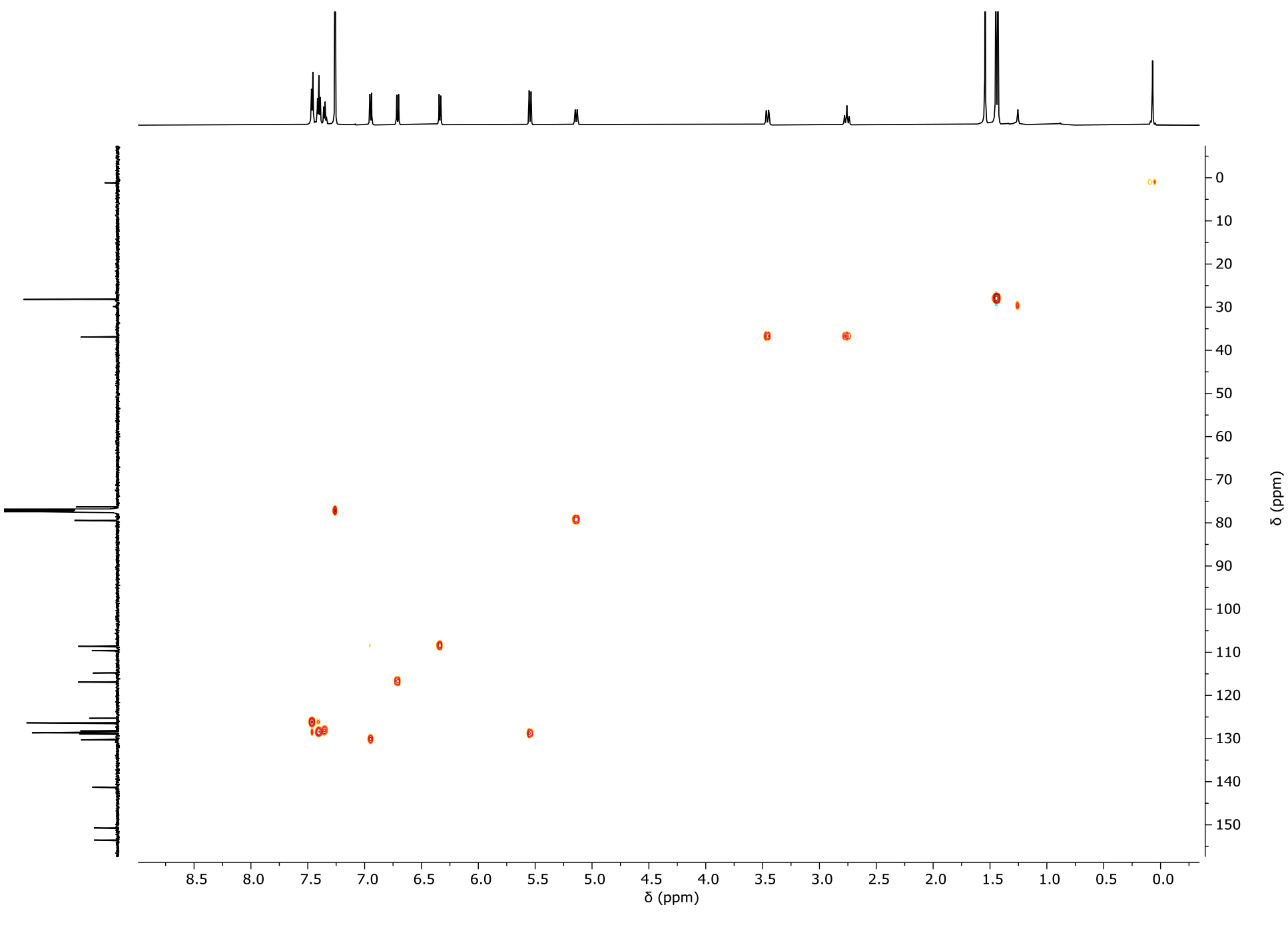

Figure S14. ^{1}H-^{13}C HSQC NMR spectrum of *(M,M)-(E)*-**2**

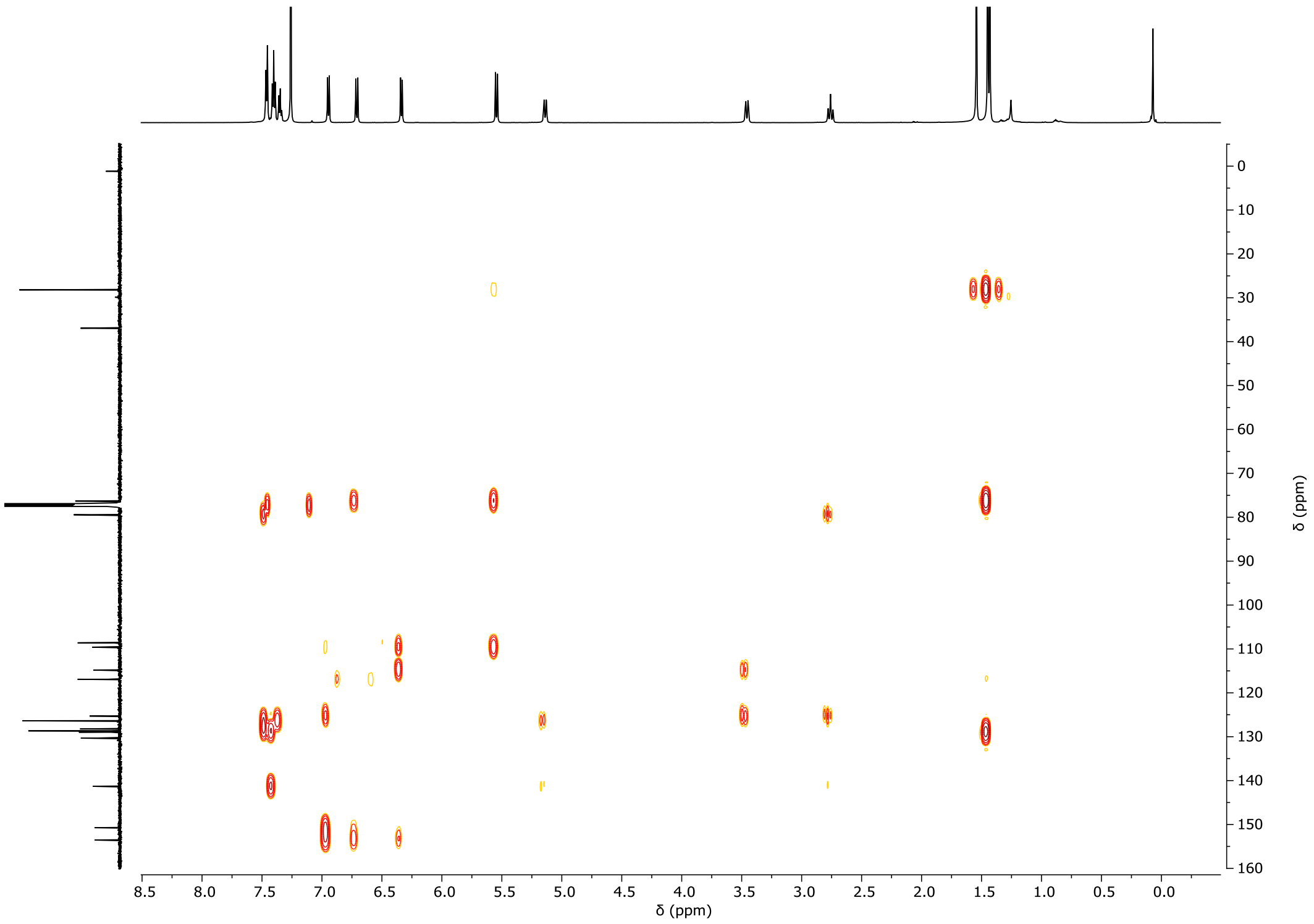

Figure S15. ^{1}H-^{13}C HMBC NMR spectrum of *(M,M)-(E)*-**2**

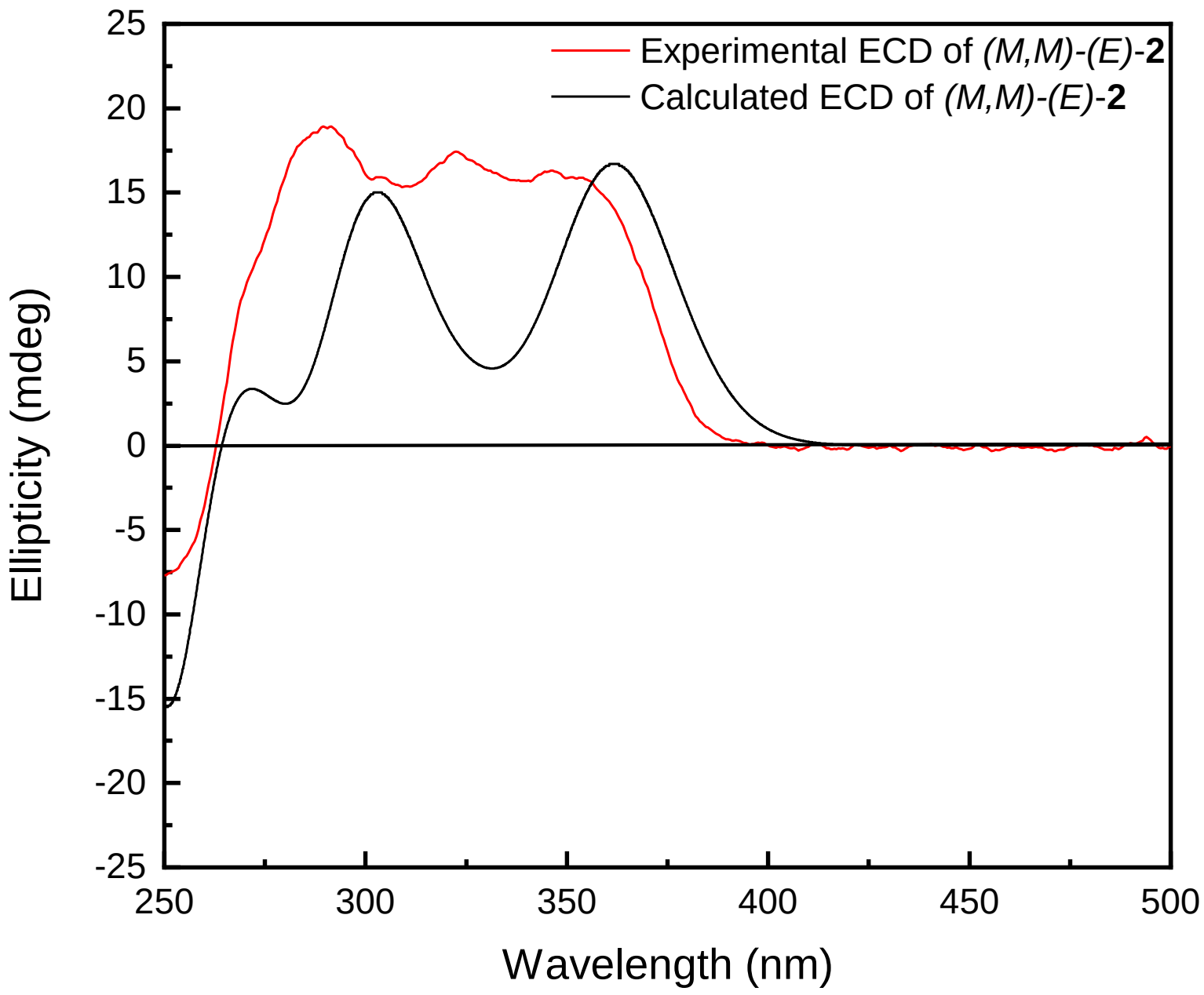

Figure S16. CD spectra of *(M,M)-(E)*-**2** (DCM, 25 μM, 20 °C). Calculated ECD spectra were done at ωB97X-D4/cc-pVDZ level of theory.

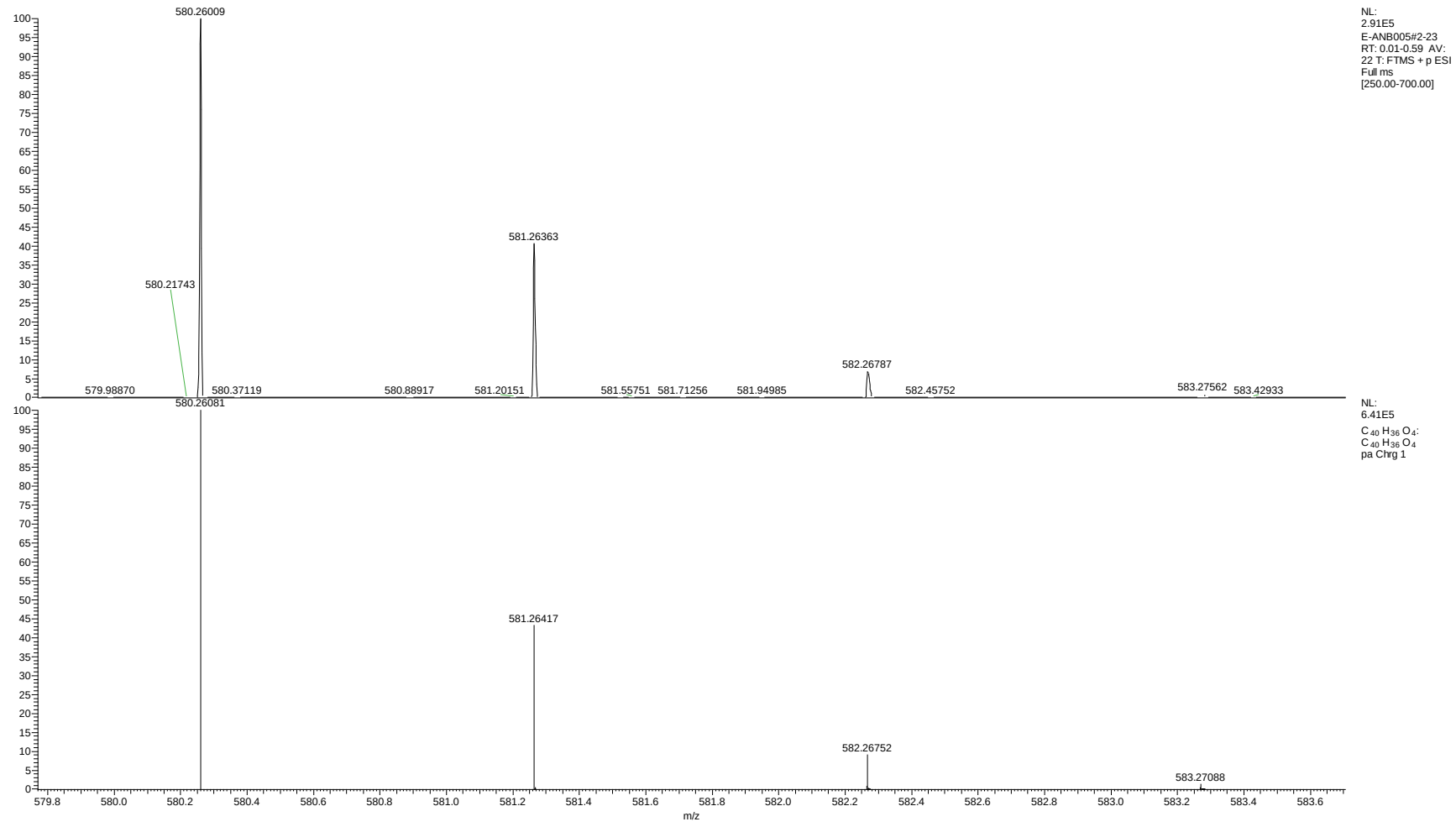

Figure S17. HRMS spectrum of *(M,M)-(E)*-**2**

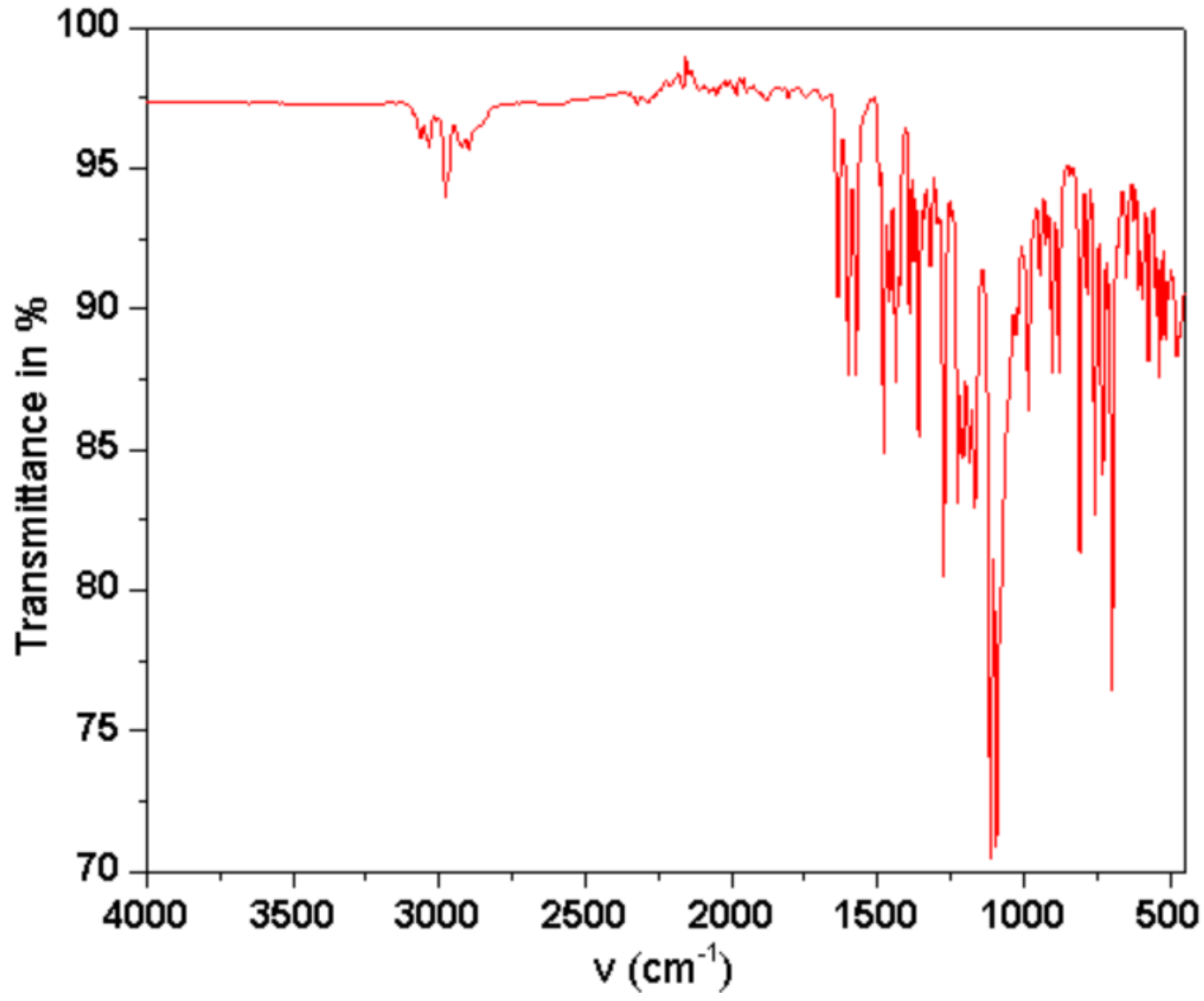

Figure S18. IR spectrum of *(M,M)-(E)*-**2**

Table S1. Crystallographic data of *(M,M)-(E)*-**2** by single crystal X-ray diffraction.

Empirical formula	$C_{40}H_{36}O_4$
Formula weight	580.69
Temperature (K)	293
Crystal system	orthorhombic
Space group	$P2_12_12_1$
a (Å)	11.4696(13)
b (Å)	11.7914(14)
c (Å)	23.397(3)
α/°	90
β/°	90
γ/°	90
Volume (Å^3)	3164.3(6)
Z	4
ρ_{calc} (g.cm^{-3})	1.219
μ (mm^{-1})	0.611
F(000)	1232.0
Crystal size (mm^3)	0.255 × 0.215 × 0.109
Radiation	CuKα (λ = 1.54178)
2θ range for data collection (°)	7.556 to 136.596
Index ranges	-13 ≤ h ≤ 12, -14 ≤ k ≤ 14, -28 ≤ l ≤ 28
Reflections collected	103232
Independent reflections	5787 [R_{int} = 0.1080, R_{sigma} = 0.0484]
Data/restraints/parameters	5787/0/401
Goodness-of-fit on F^2	1.068
Final R indexes [I>=2σ (I)]	R_1 = 0.0571, wR_2 = 0.1368
Final R indexes [all data]	R_1 = 0.0621, wR_2 = 0.1441
Largest diff. peak/hole (e.Å^{-3})	0.16/-0.28
Flack parameter	0.18(9)

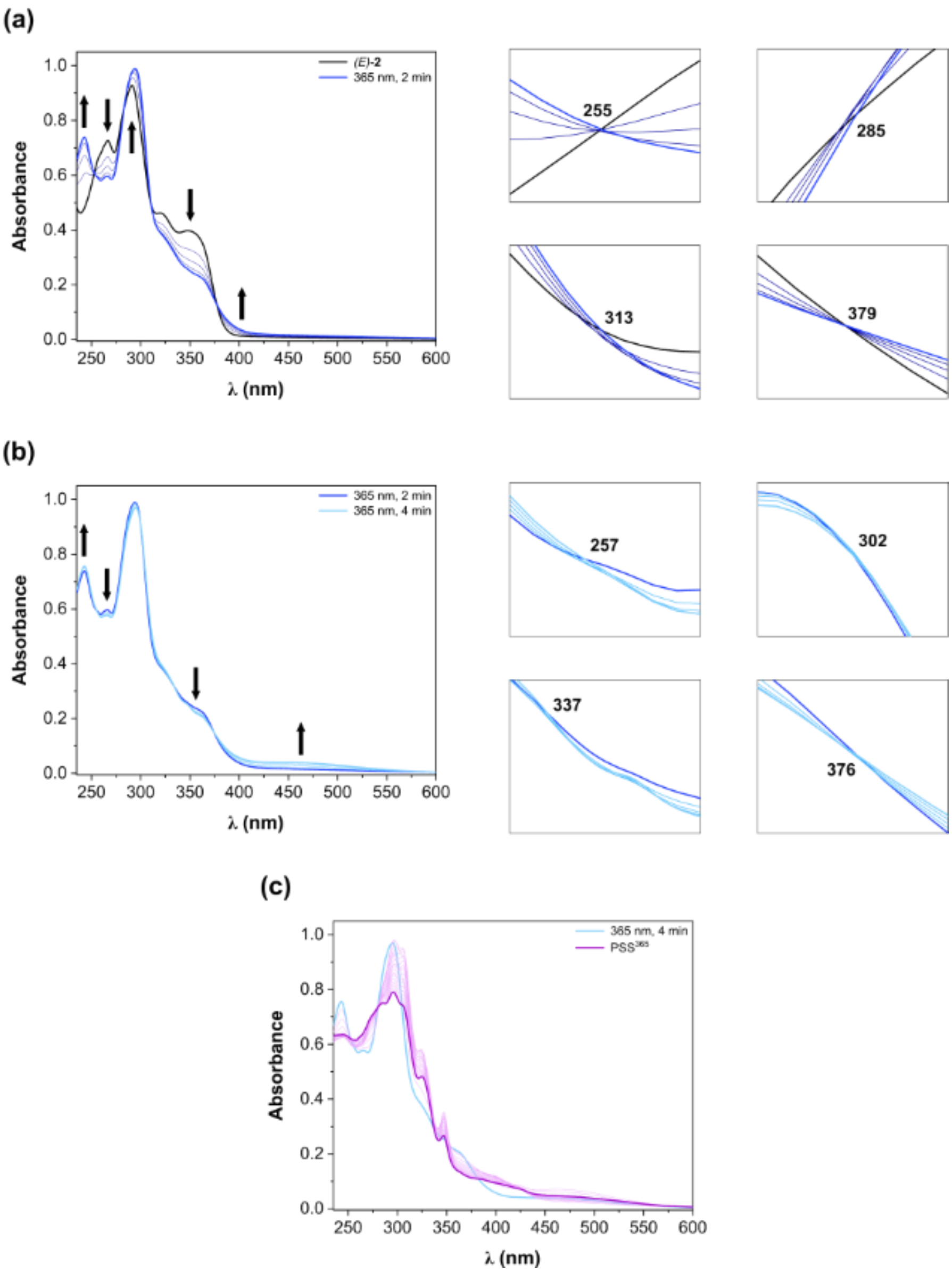

Figure S19. Photoirradiation analysis of *(M,M)-(E)*-**2** at 365 nm during **(a)** 7 min, **(b)** 14 min and **(c)** 90 min (PSS^{405}) with their respective pseudo-isosbestic points (20 μM in DCM, 1.0 A, 25°C).

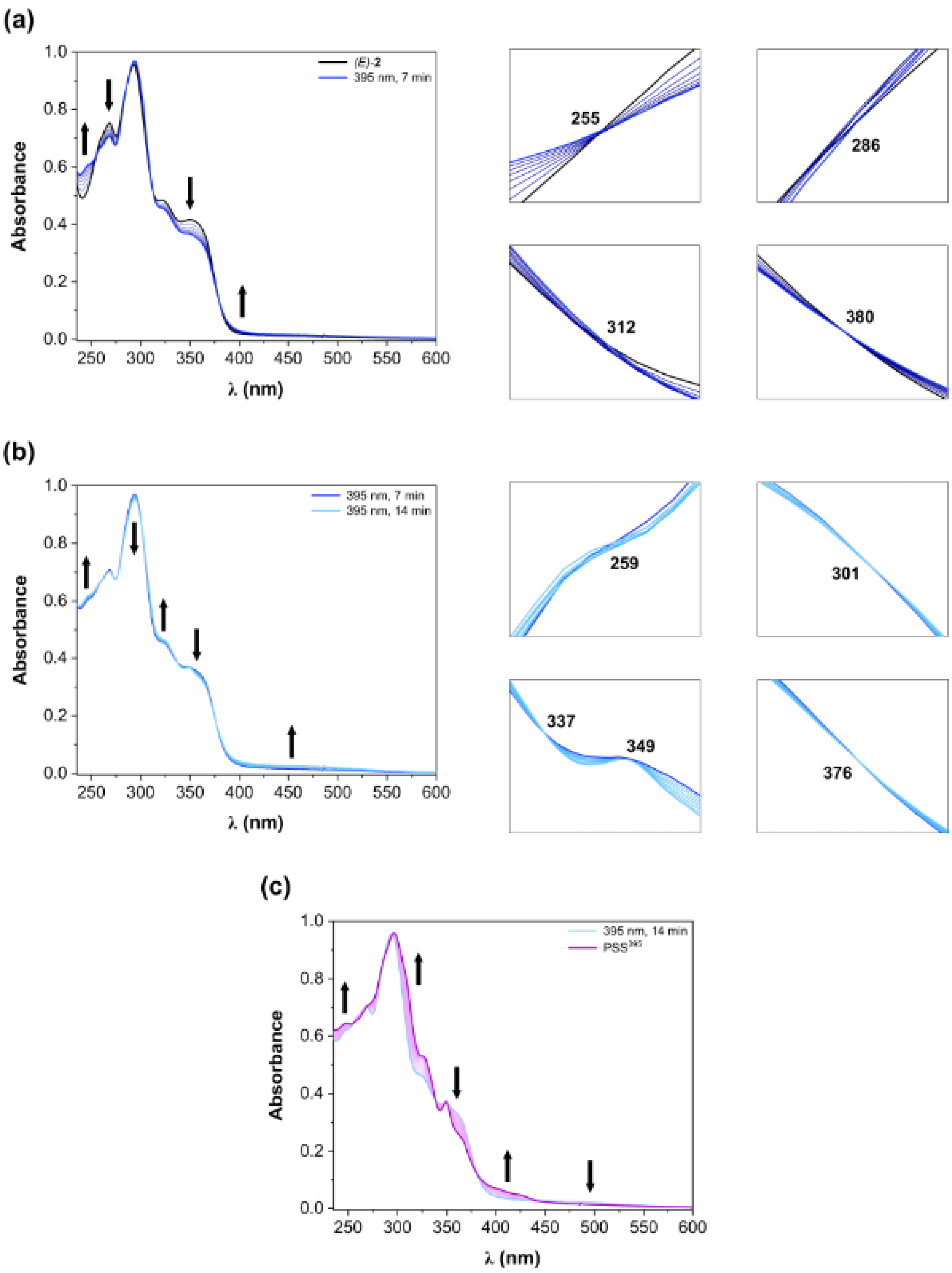

Figure S20. Photoirradiation analysis of *(M,M)-(E)*-**2** at 395 nm during (a) 7 min, (b) 14 min and (c) 90 min (PSS405) with their respective pseudo-isosbestic points (20 μM in DCM, 1.0 A, 25 °C).

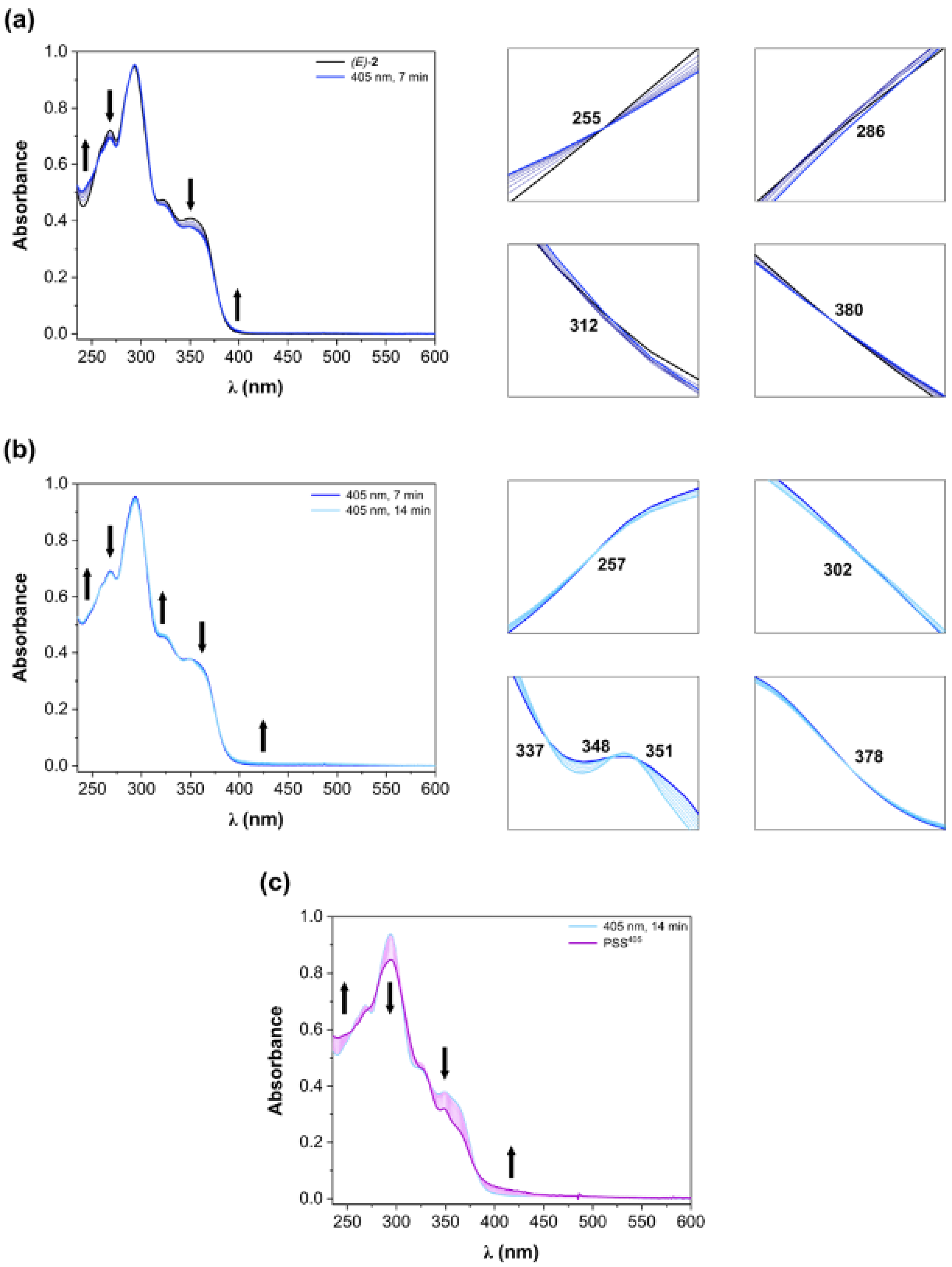

Figure S21. Photoirradiation analysis of *(M,M)-(E)*-**2** at 405 nm during (a) 7 min, (b) 14 min and (c) 90 min (PSS405) with their respective pseudo-isosbestic points (20 μM in DCM, 1.0 A, 25 °C).

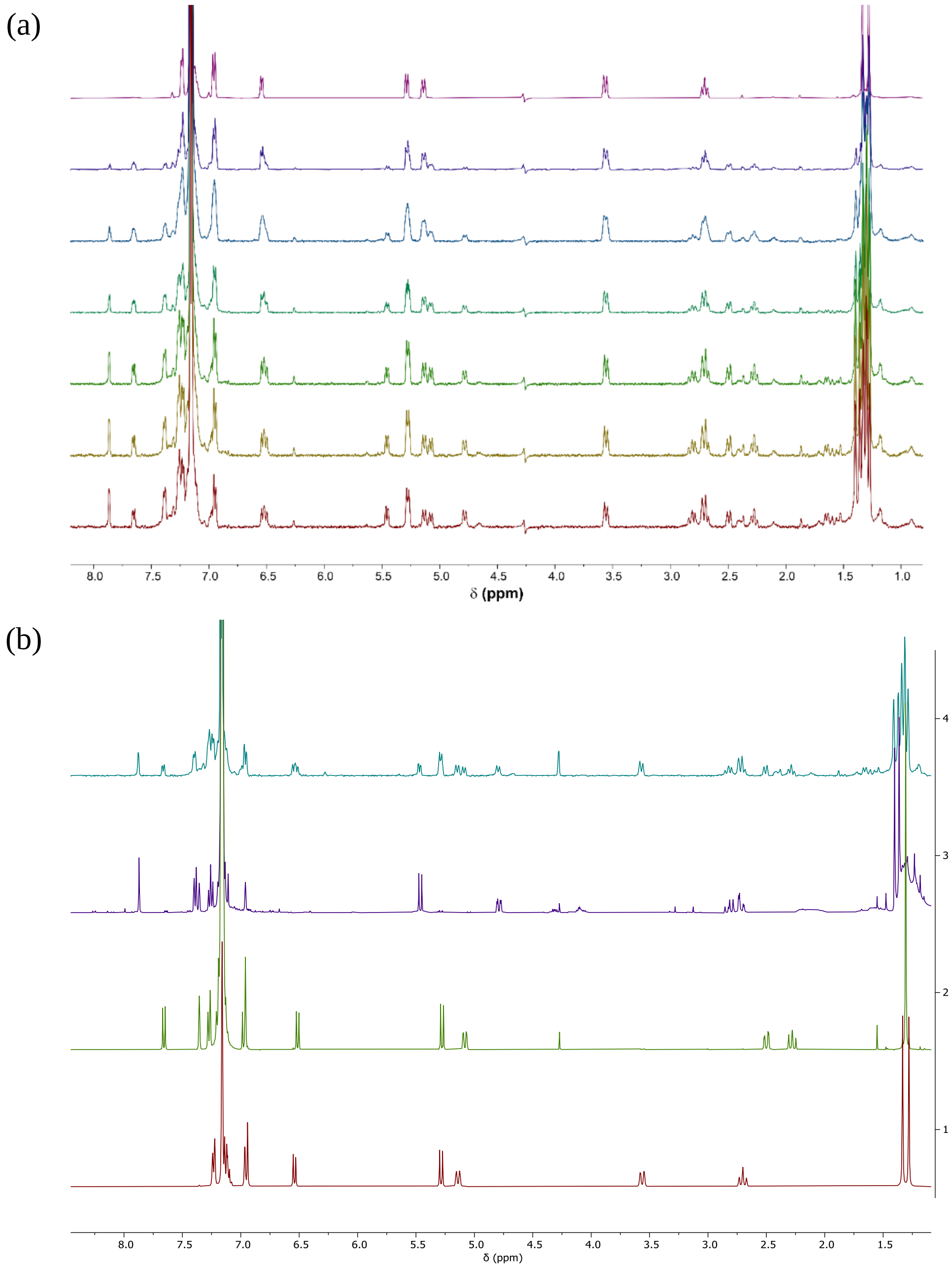

Figure S22. Stacked ^{1}H NMR spectra of the photochemical oxidation of *(M,M)-(E)*-**2** to **3**. (a) Subsequent *(E/Z)*-photoisomerization, photocyclization and oxidation of *(E)*-**2** during 30 min of irradiation at 395 nm (from top to bottom, from 0 to 30 min every 5 min of irradiation recorded) (500 MHz, 2.0 mM in C_6D_6, 25 °C) and (b) Comparison of (from top to bottom) irradiation result, phenanthrene **3,** *(P)-(Z)*-**2**, and *(M,M)-(E)*-**2** (500 MHz, 2.0 mM in C_6D_6, 25 °C).

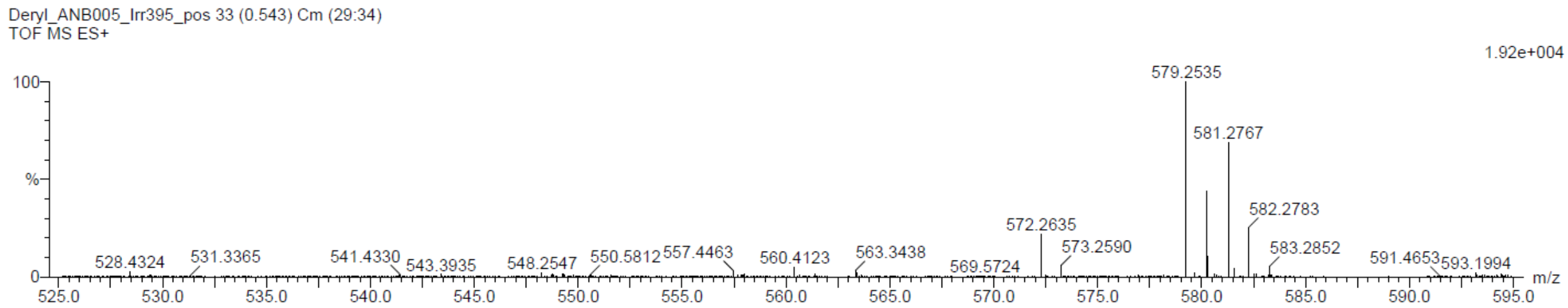

Figure S23. HRMS spectrum of *(M,M)-(E)*-**2** upon irradiation with 395 nm for 60 minutes

S3. Characterization of (P)-(Z)-2

Table S2. Crystallographic data of *(P)*-*(Z)*-**2** by single crystal X-ray diffraction.

Empirical formula	$C_{40}H_{36}O_4$
Formula weight	580.69
Temperature (K)	100
Crystal system	monoclinic
Space group	C2
a (Å)	22.5887(10)
b (Å)	10.7031(5)
c (Å)	20.0485(9)
α (°)	90
β (°)	101.394(3)
γ (°)	90
Volume (Å^3)	4751.6(4)
Z	6
ρ_{calc} (g.cm^{-3})	1.218
μ (mm^{-1})	0.611
F(000)	1848.0
Crystal size (mm^3)	0.229 × 0.137 × 0.075
Radiation	CuKα (λ = 1.54178)
2θ range for data collection (°)	4.496 to 136.482
Index ranges	$-27 \le h \le 27$, $-12 \le k \le 12$, $-24 \le l \le 24$
Reflections collected	94702
Independent reflections	8670 [R_{int} = 0.1114, R_{sigma} = 0.0491]
Data/restraints/parameters	8670/1/601
Goodness-of-fit on F^2	1.067
Final R indexes [I>=2σ (I)]	R_1 = 0.0470, wR_2 = 0.1238
Final R indexes [all data]	R_1 = 0.0510, wR_2 = 0.1269
Largest diff. peak/hole (e.Å^{-3})	0.56/-0.25
Flack parameter	1.38(11)

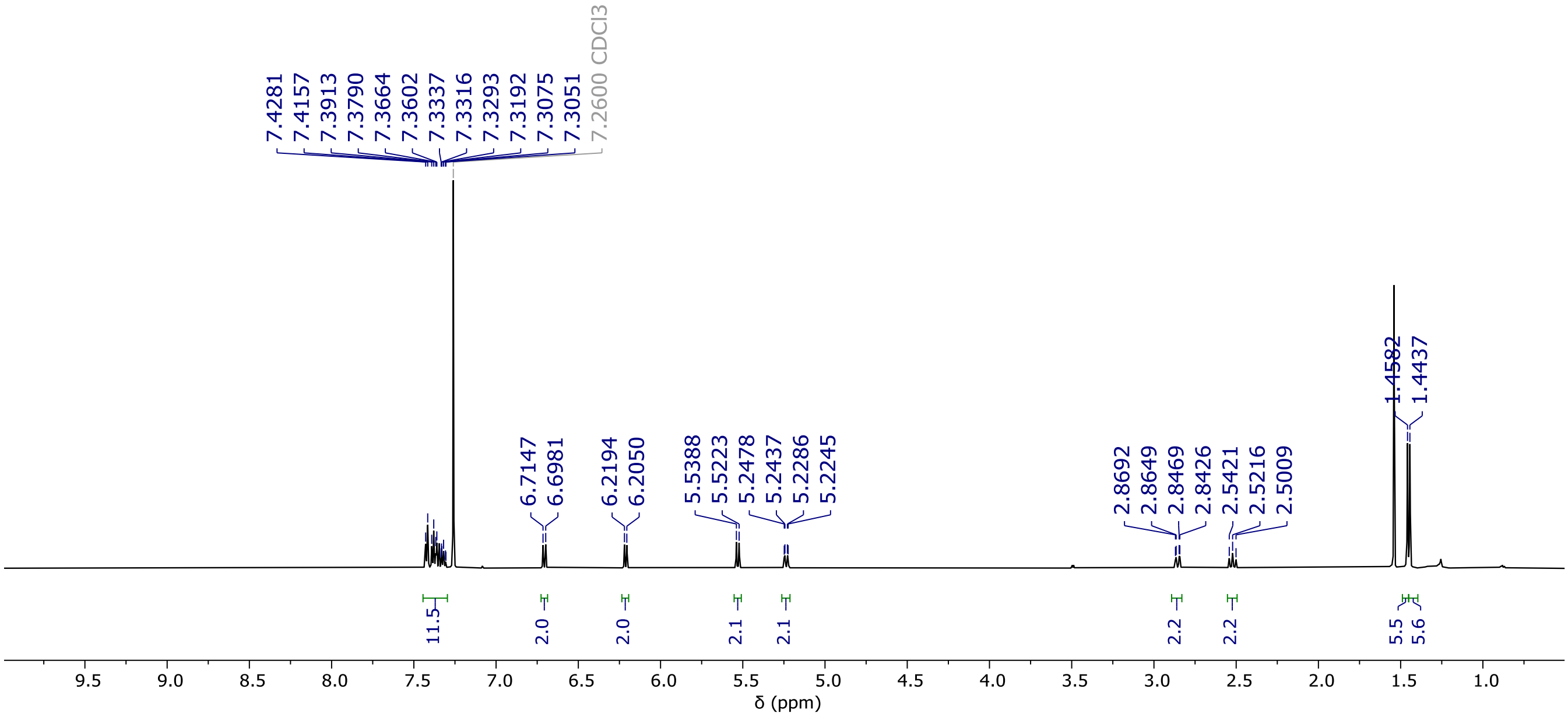

Figure S24. ^{1}H NMR spectrum of *(P)-(Z)*-**2** (600 MHz, $CDCl_3$, 25 °C)

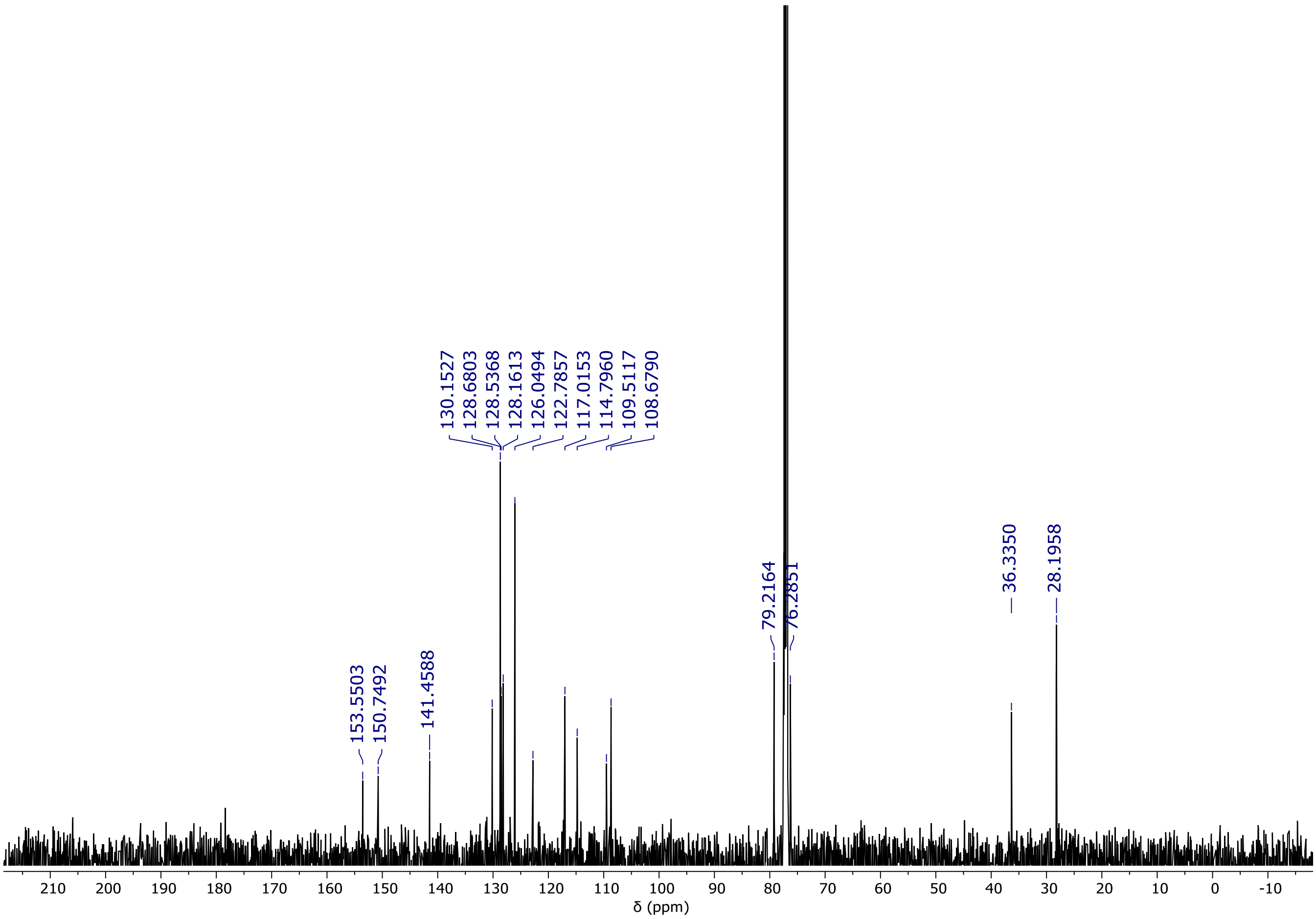

Figure S25. ^{13}C NMR spectrum of *(P)-(Z)*-**2** (150 MHz, $CDCl_3$, 25 °C)

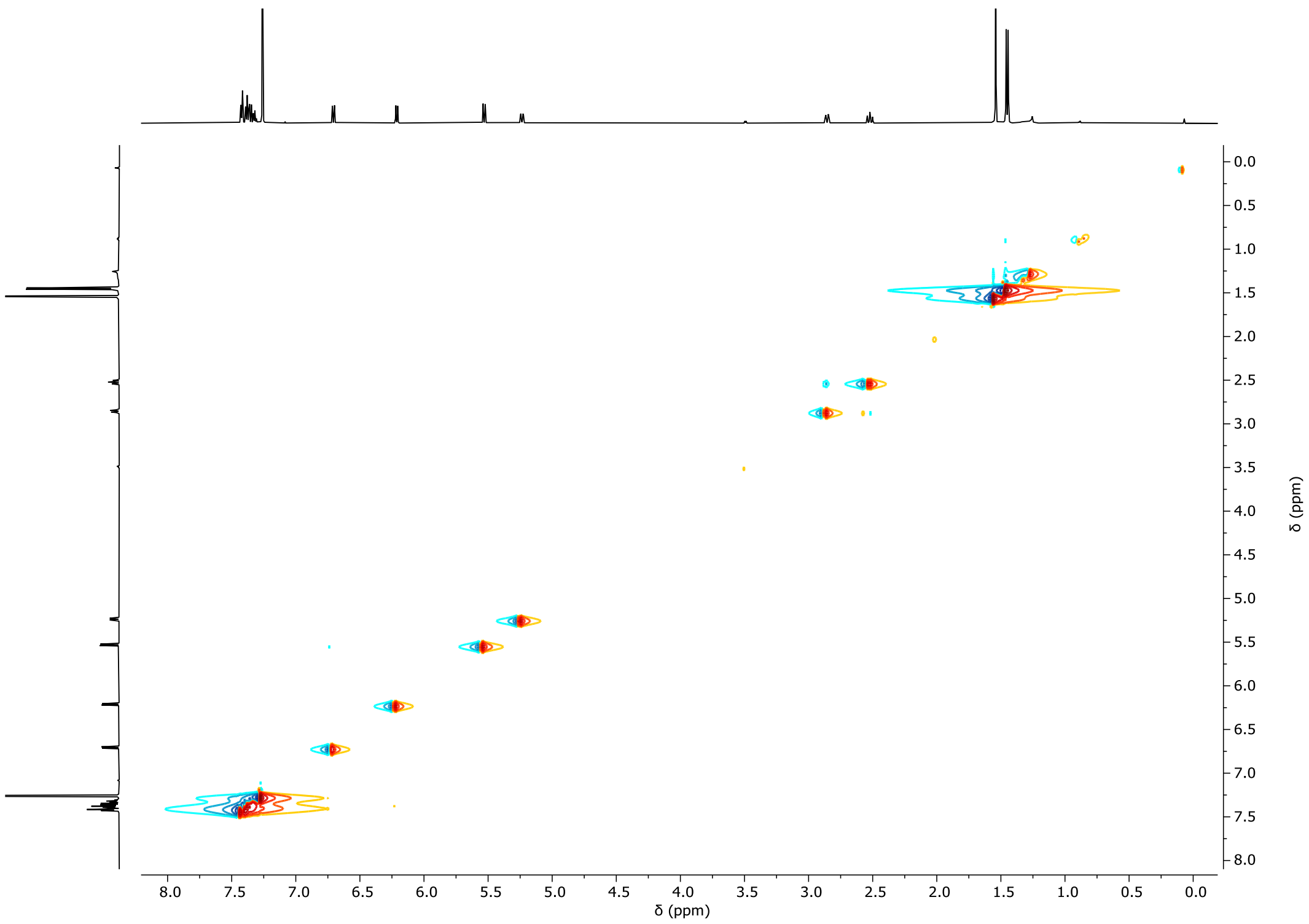

Figure S26. ^{1}H-^{1}H NOESY NMR spectra of *(P)-(Z)*-**2**

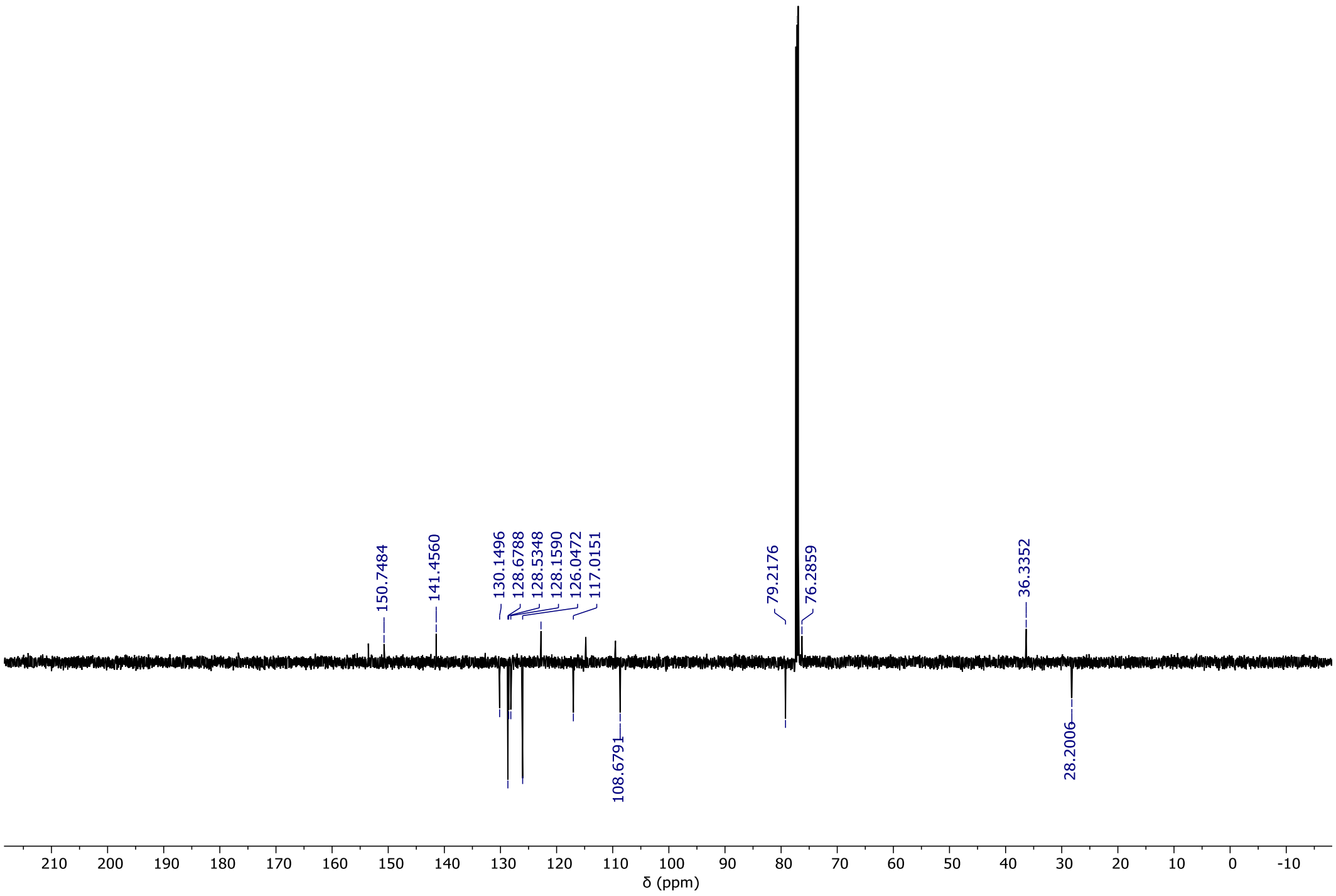

Figure S27. ^{13}C NMR spectrum APT spectra of *(P)-(Z)*-**2**

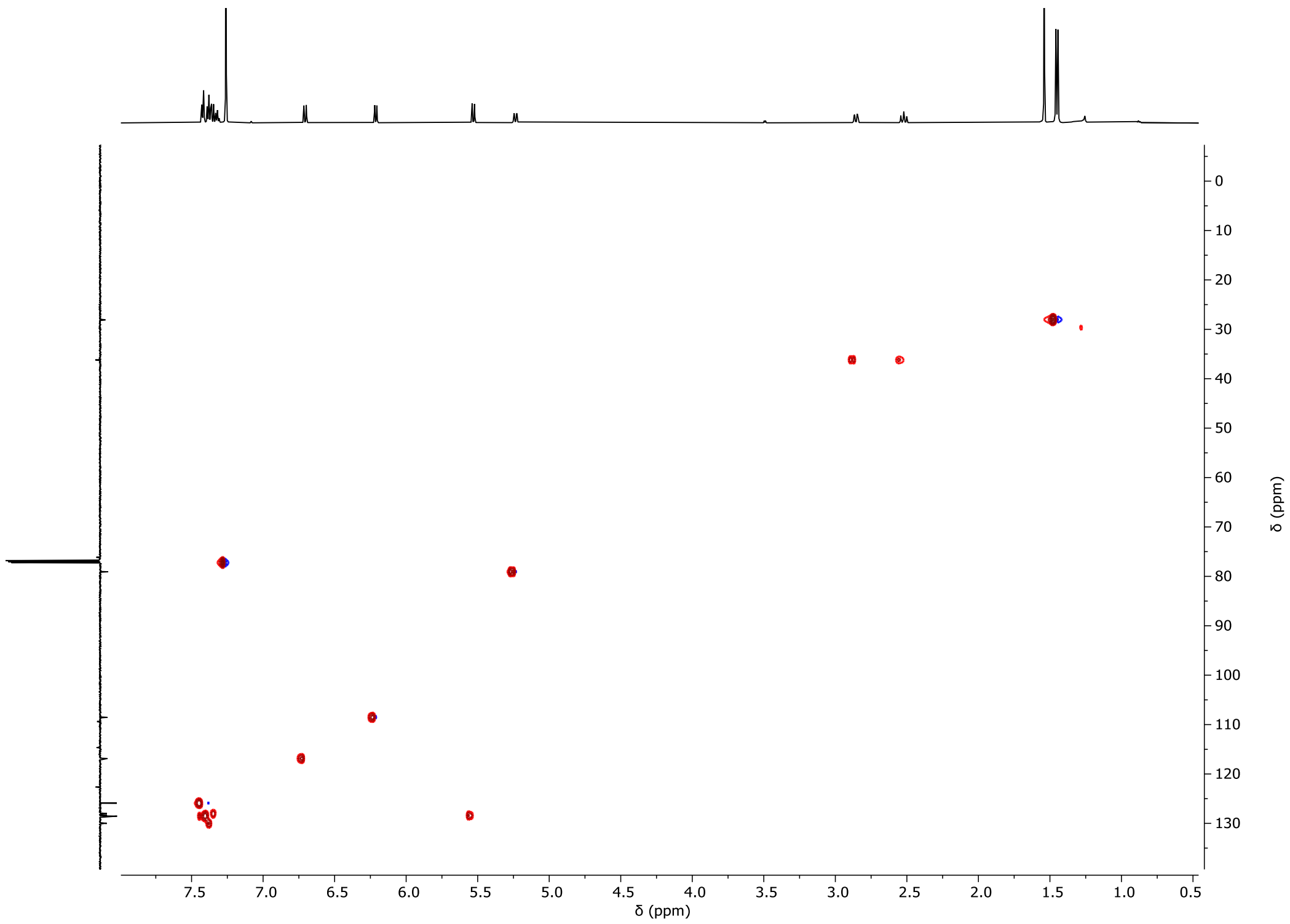

Figure S28. ^{1}H-^{13}C HSQC NMR spectrum of *(P)-(Z)*-**2**

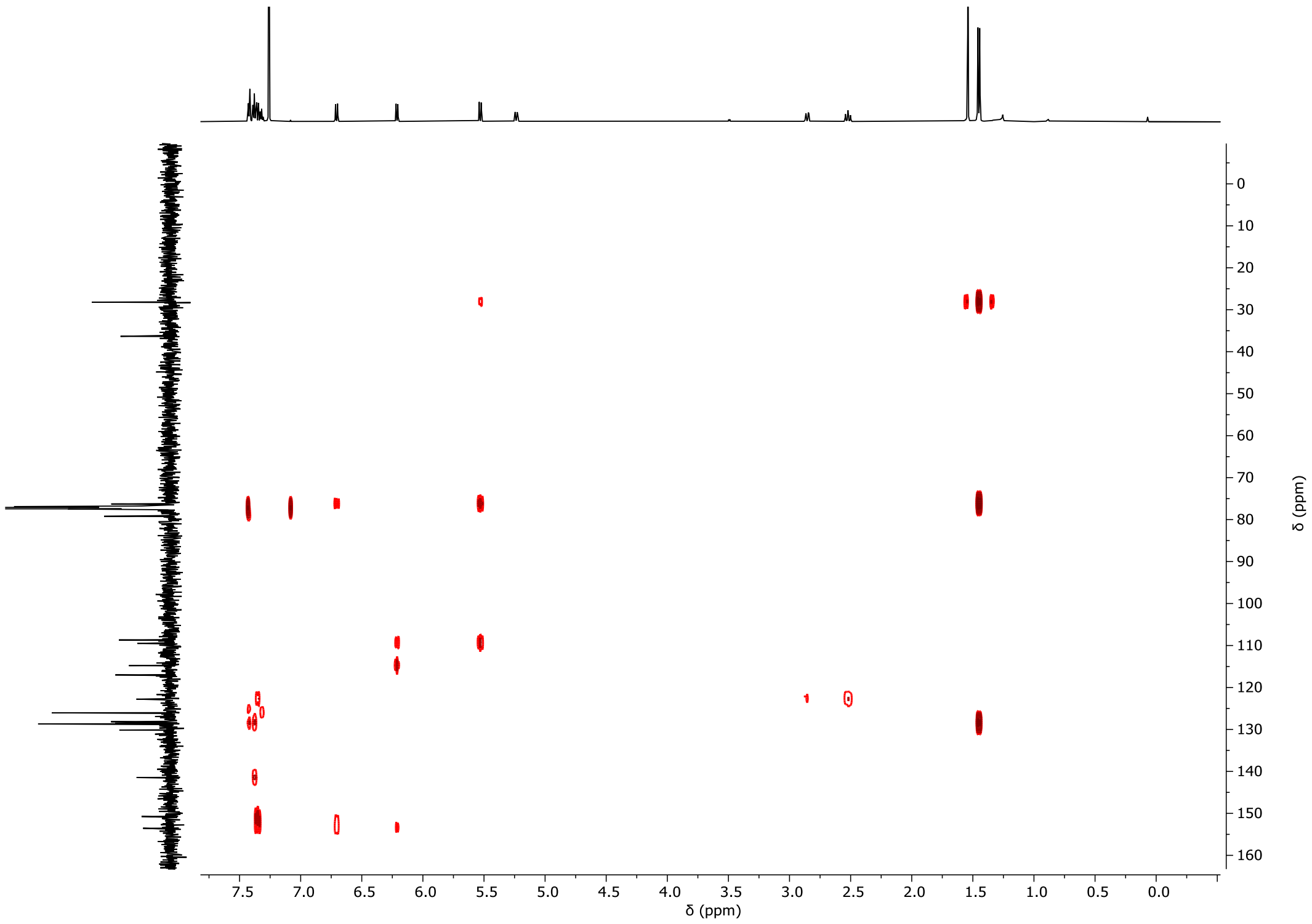

Figure S29. ^{1}H-^{13}C HMBC NMR spectrum of *(P)-(Z)*-**2**

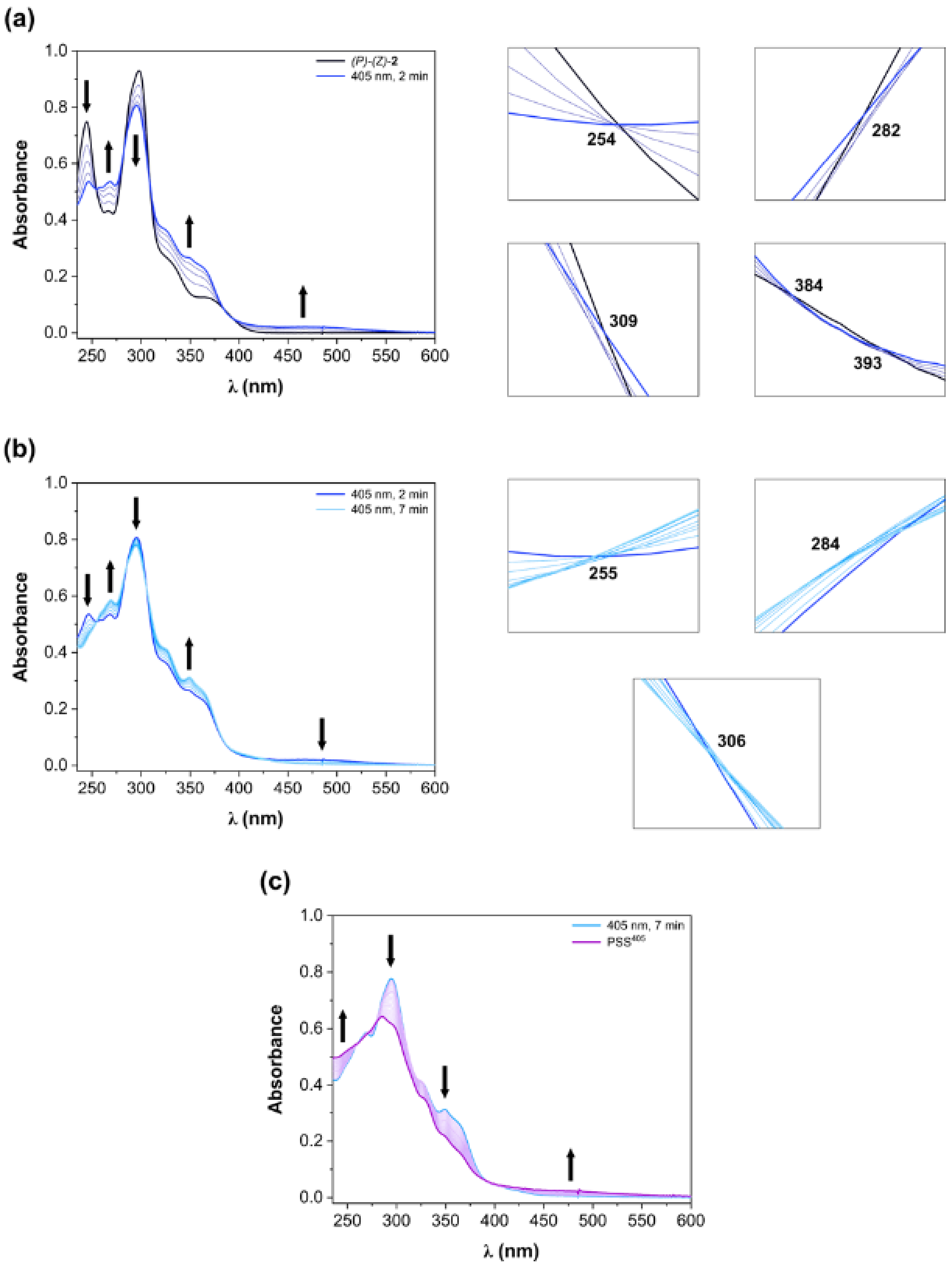

Figure S30. Photoirradiation analysis of (*P*)-(*Z*)-**2** at 405 nm during (a) 2 min, (b) 7 min and (c) 90 min (PSS^{405}) with their respective pseudo-isosbestic points (20 µM in DCM, 1.0 A, 25 °C).

S4. Characterization of 3

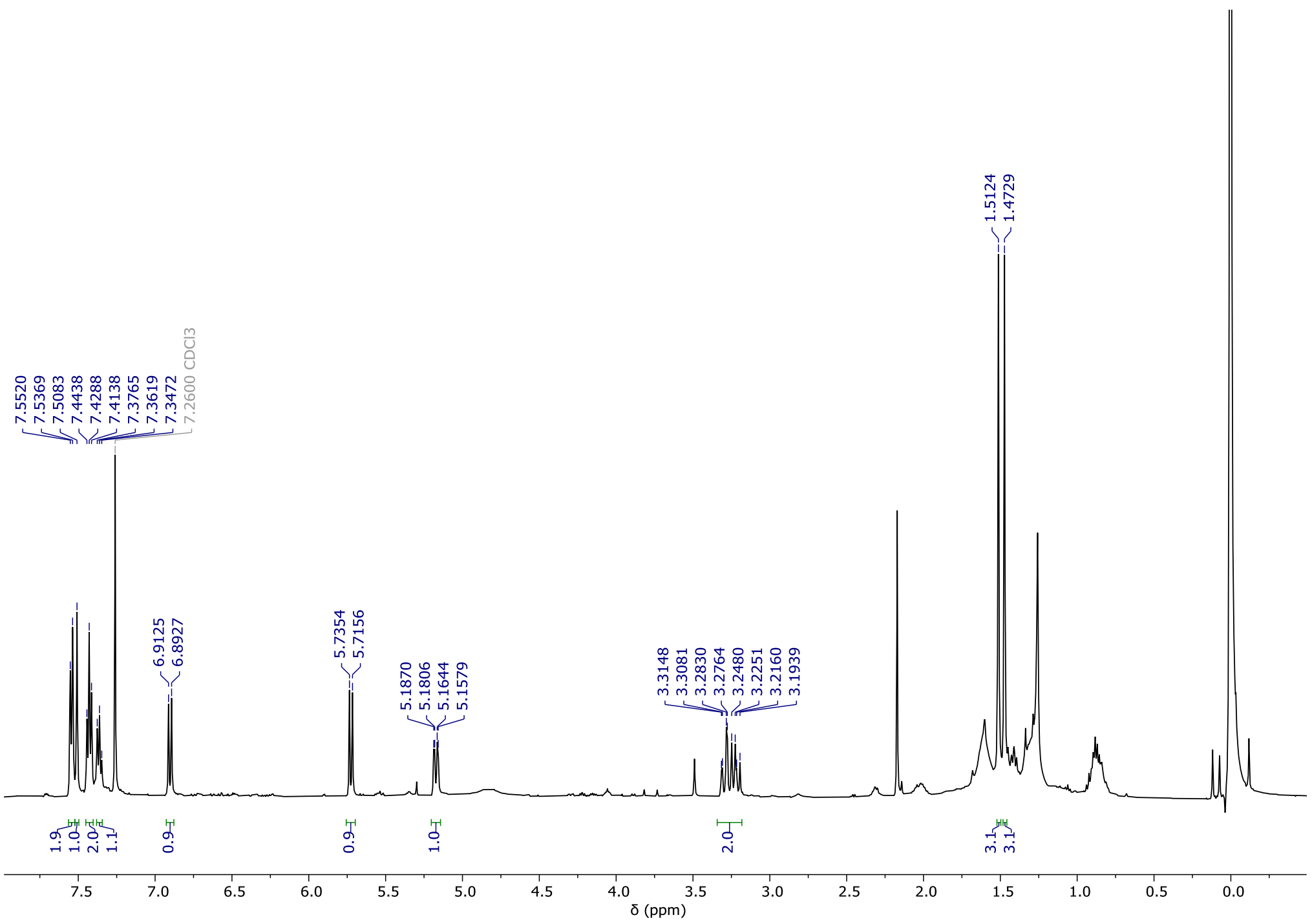

Figure S31. ^{1}H NMR spectrum of **3** (500 MHz, $CDCl_3$, 25 °C)

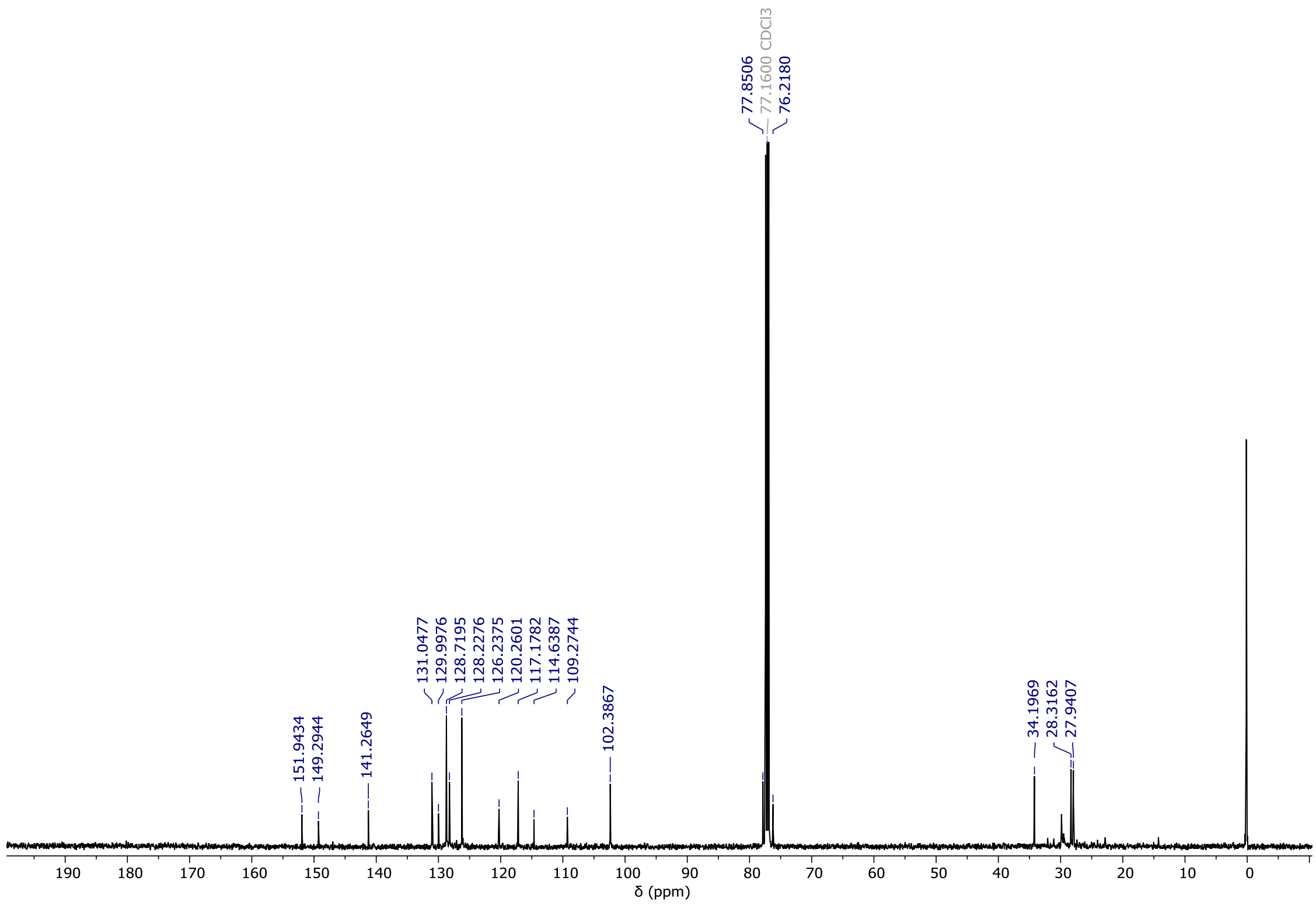

Figure S32. ^{13}C NMR spectrum of **3** (126 MHz, $CDCl_3$, 25 °C)

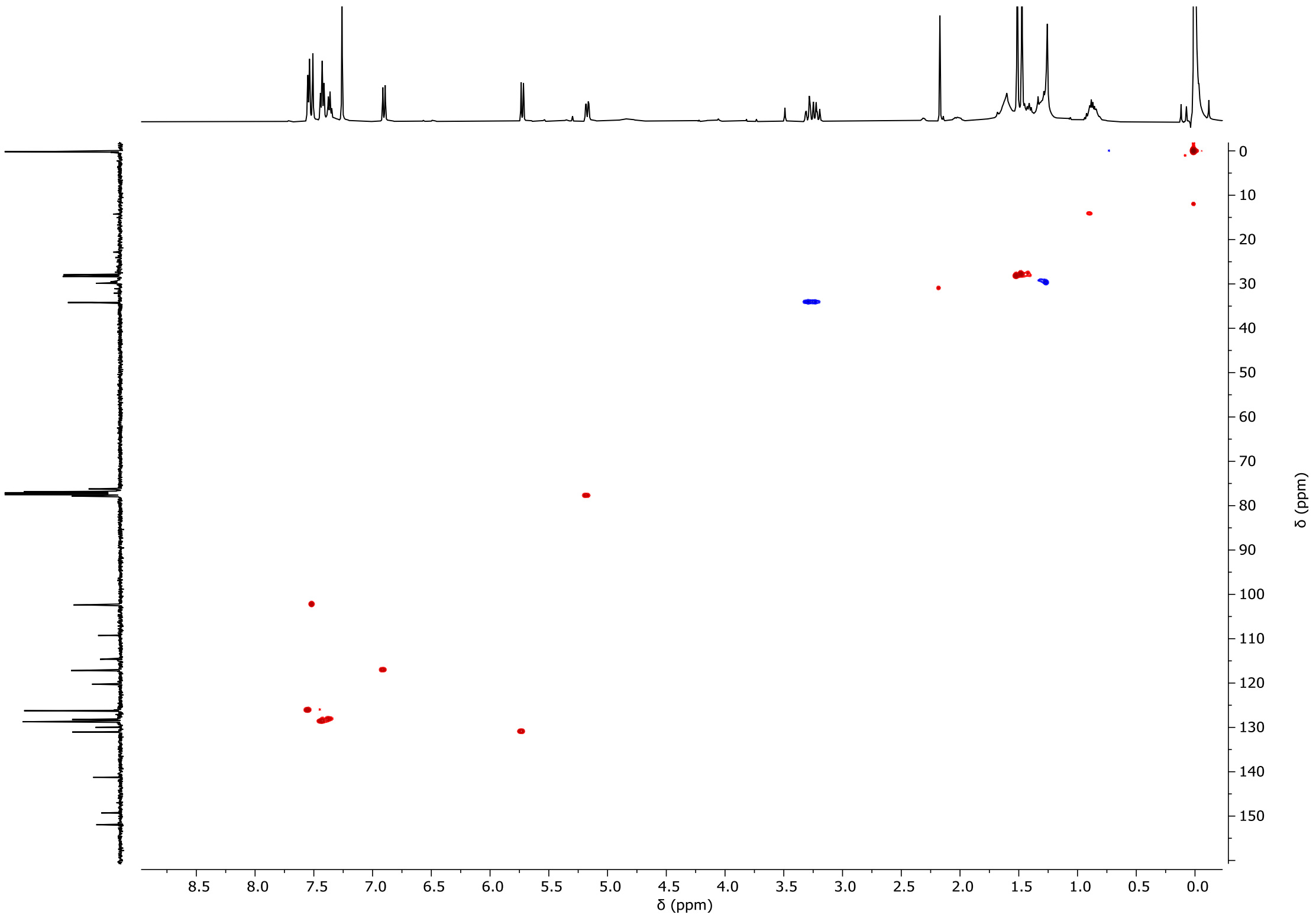

Figure S33. ^{1}H-^{13}C HSQC NMR spectrum of **3**

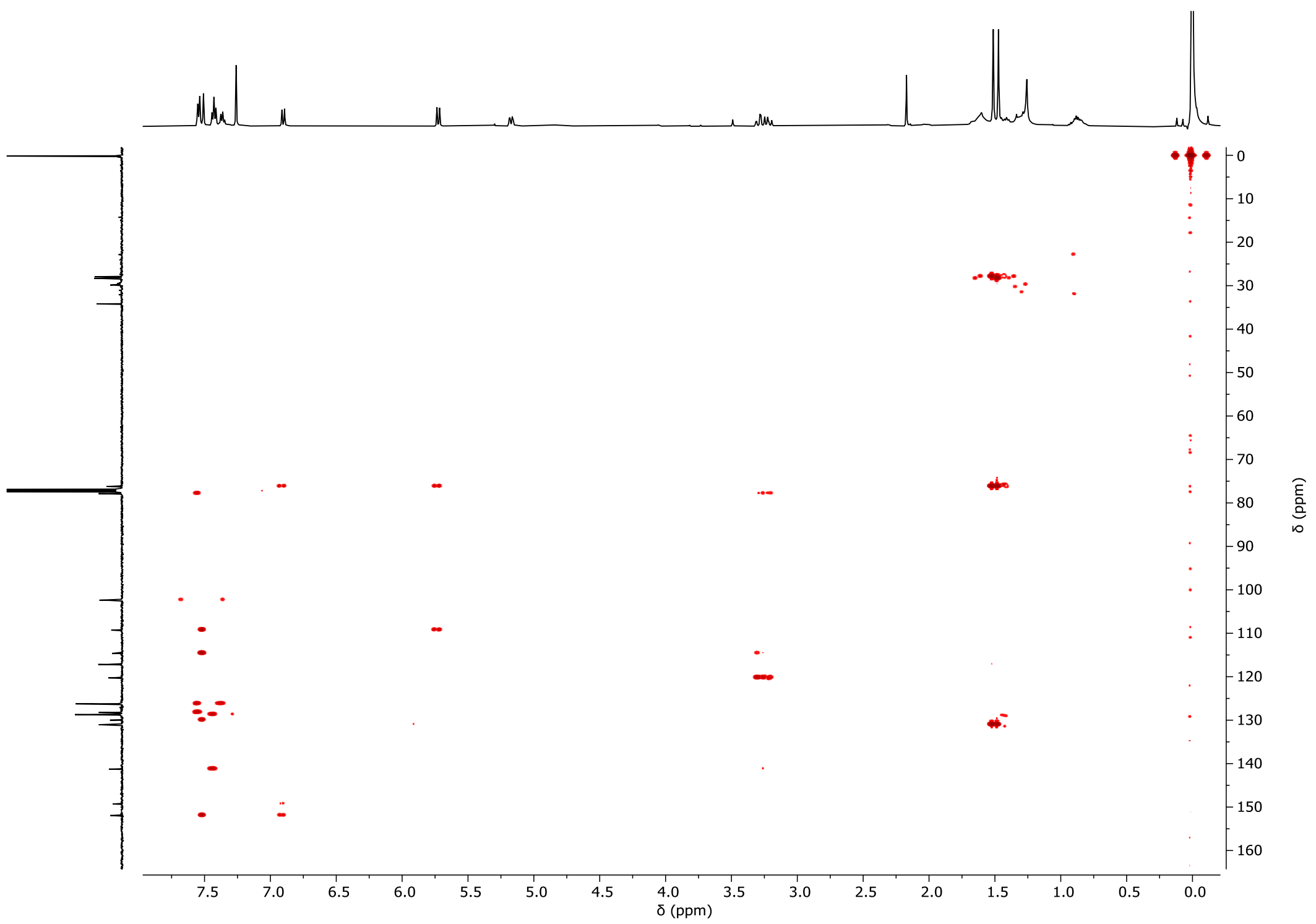

Figure S34. ^{1}H-^{13}C HMBC NMR spectrum of **3**

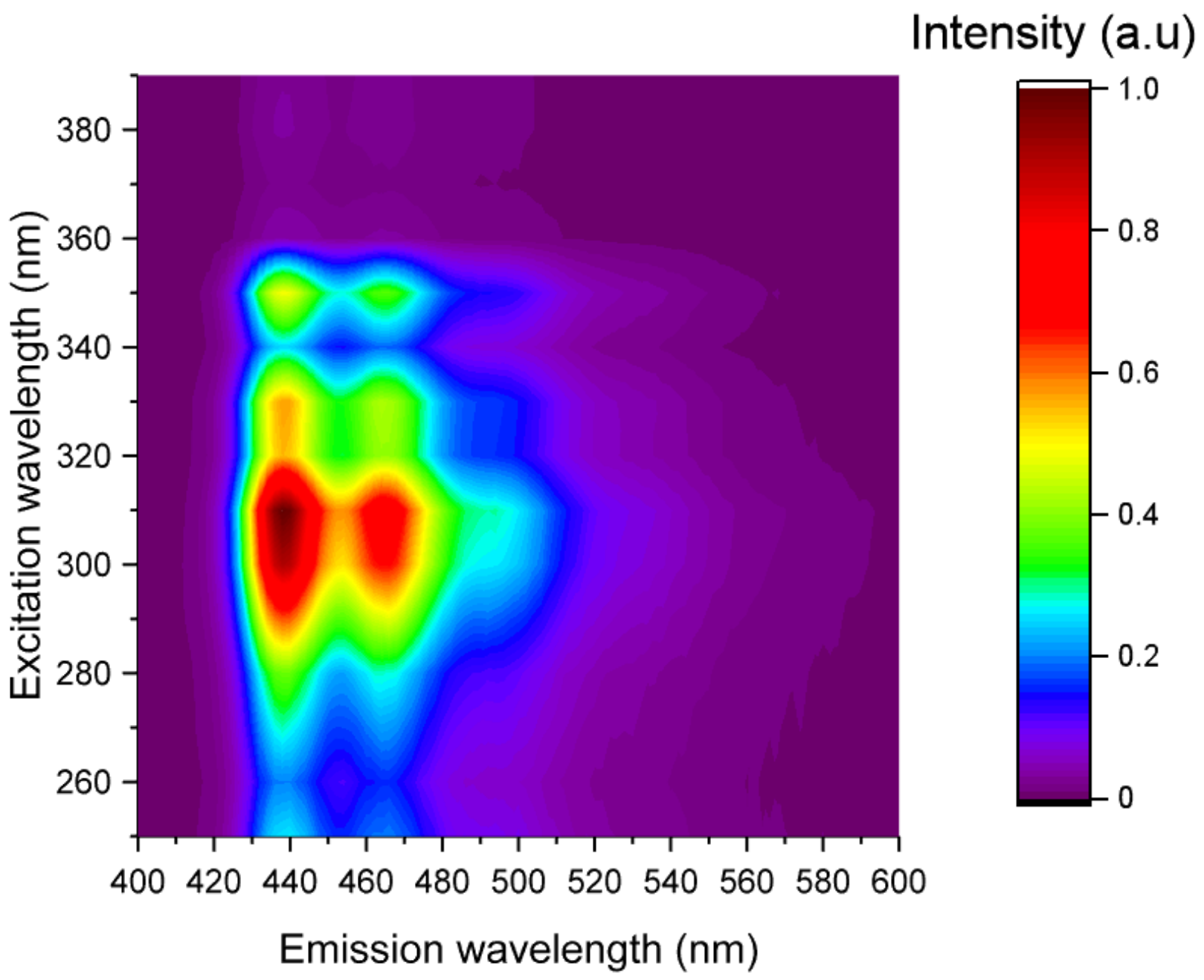

Figure S35. Fluorescence emission/excitation map of phenanthrene **3** (17.3 μM in DCM).

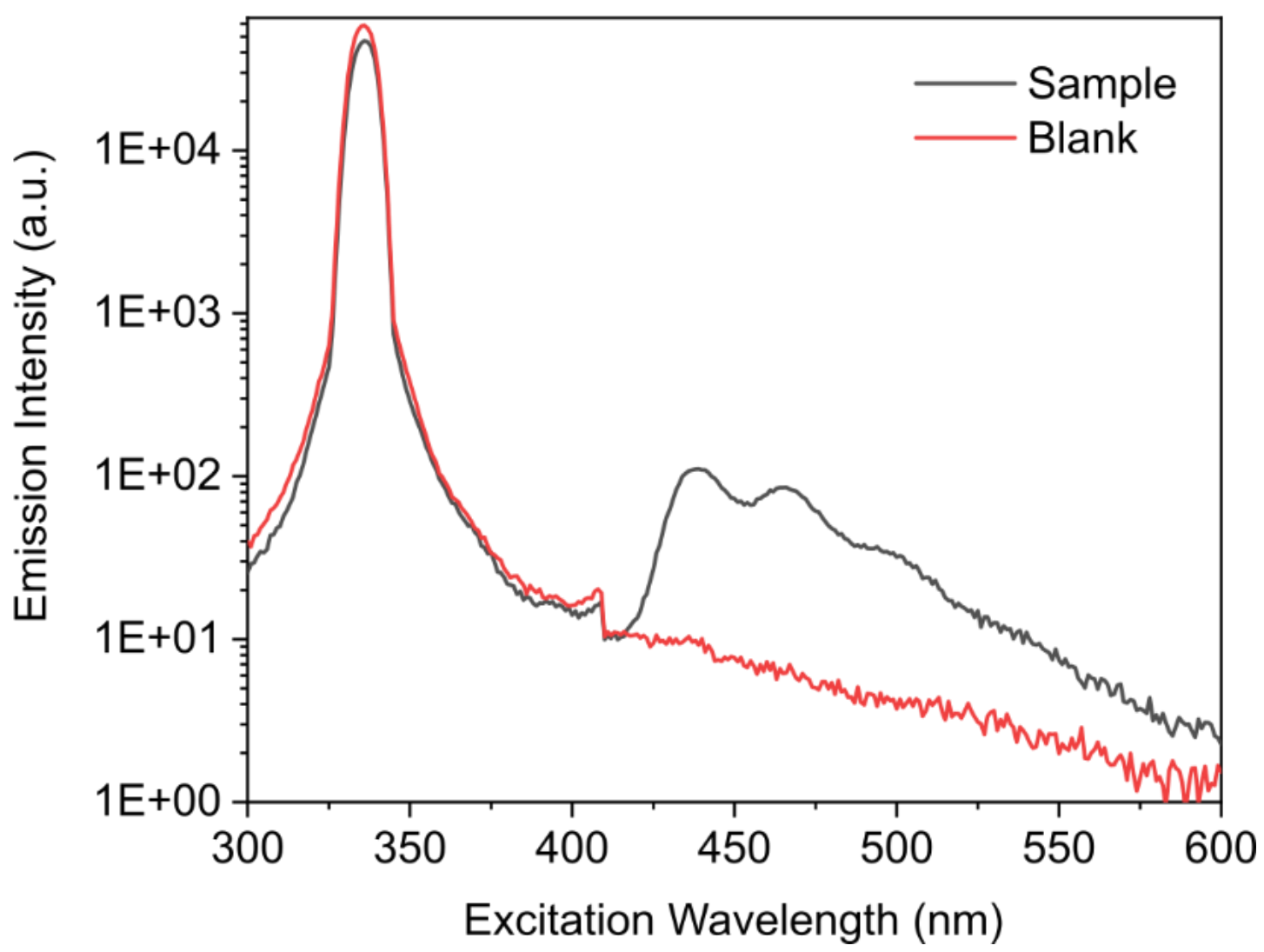

Figure S36. Plot of the fluorescence emission curve of **3** (17.3 μM in DCM). Scatter range used in the calculation 325–347 nm, emission range 411–600 nm, QY = 4.97%.

S5. Computational Study

Computational details. Initial guess of geometries, conformers of 1-3 and their stereoisomers were generated using Merck Molecular Force Field (MMFF94) as implemented in the Avogadro software.[62] Further optimizations of ground state minima were done at ωB97X-D4/cc-pVDZ level of theory while deploying the conductor-like polarizable continuum model (C-PCM) to represent the dichloromethane solvent (ε = 9.08).[63-66] Minimum energy paths (MEPs) of gaseous ***E*-(2)** isomerization and ***Z*-(2)** cyclization at the ground state (S_0) potential energy surfaces (PESs) were estimated using climbing image nudged elastic band (CI-NEB) method with its original convergence criteria at SF-TD-BHandHLYP/cc-pVDZ level of theory.[67,68] All ground state minima were reoptimized at the SF-TD-BHandHLYP/cc-pVDZ level during NEB calculation. NEB images at local maxima (at the S_1 and S_0 PESs) along the S_0 MEPs were optimized towards saddle points using an eigenvector following algorithm at their corresponding PESus.[69] NEB images with the lowest energy differences between ground state and excited state were used as inputs for minimum energy conical intersections (CIs) optimization with the updated branching plane (UBP) method,[70] Local minima at the S_1 PESs found along the two-dimensional S_0 MEPs were optimized at the same level of theory. All geometry optimizations other than the ground state minima were performed at the SF-TD-BHandHLYP/cc-pVDZ level. Grimme's D4 dispersion correction were employed in all DFT calculations, which were done within the ORCA 5.0.3 ab initio, DFT, and semiempirical SCF-MO package environment.[71–73] RIJCOSX approximation was used to accelerate the calculation of Hartree-Fock exchange, which is the default for hybrid DFT in ORCA.[74] Spatial natural orbitals were generated at SF-TD-BHandHLYP/cc-pVDZ level of theory using ORCA and Multiwfn.[75,76] Absorption and electronic circular dichroism (ECD) spectra were calculated at the LR-sTD-PBE0/def2-TZVP level of theory and C-PCM to represent dichloromethane solvent.[77,78] The calculated line spectra were broadened with a Gaussian function with a full width at half-maximum (FWHM) of 0.32 eV to match the peaks of the experimental spectra. All quantum chemical calculations were performed using the ORCA software package. The electronic structures and potential energy surfaces (PES) associated with the stereoisomerization processes (*M,M*)−(*E*)-$\mathbf{2}_{eq}$→(*P,P*)−(*E*)-$\mathbf{2}_{ax}$ and (*M*)−(*Z*)-$\mathbf{2}_{eq}$→(*P*)−(*Z*)-$\mathbf{2}_{ax}$ were investigated at the r²SCAN-3c level of theory. This composite method combines the r²SCAN meta-GGA functional with the D4 dispersion correction and geometrical counterpoise (gCP) treatment of basis-set superposition errors, providing a reliable balance between computational efficiency and accuracy for large π-conjugated systems. To elucidate the detailed reaction mechanism, the minimum energy reaction pathway connecting each pair of enantiomeric states was first explored using the Nudged Elastic Band (NEB) method. The NEB calculations were carried out to locate the Climbing Image (CI) along the reaction coordinate, corresponding to the highest-energy structure on the minimum energy path. The obtained CI structure was subsequently used as the initial guess for transition-state optimization (Opt TS) within ORCA, employing tight convergence criteria and the quasi-Newton method. The resulting transition-state (TS) geometries were confirmed by vibrational frequency analysis, ensuring the presence of one and only one imaginary frequency corresponding to the reaction coordinate.

i) 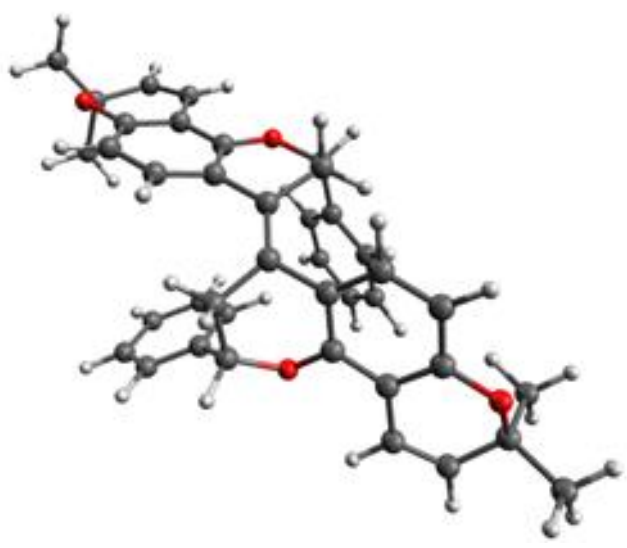

ii) 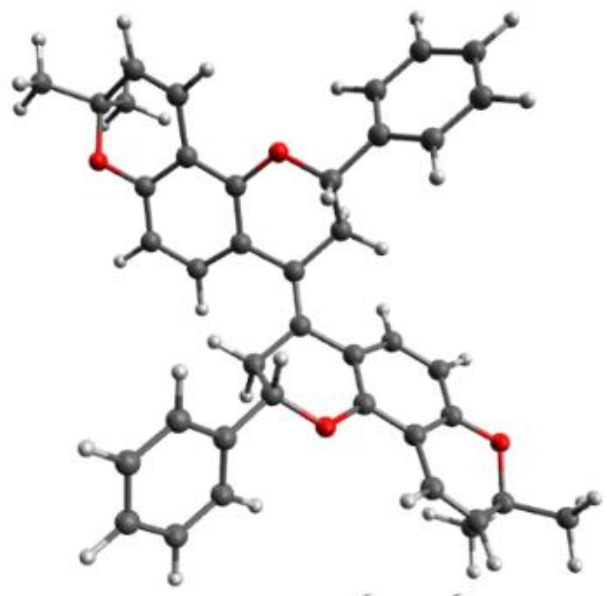

iii) 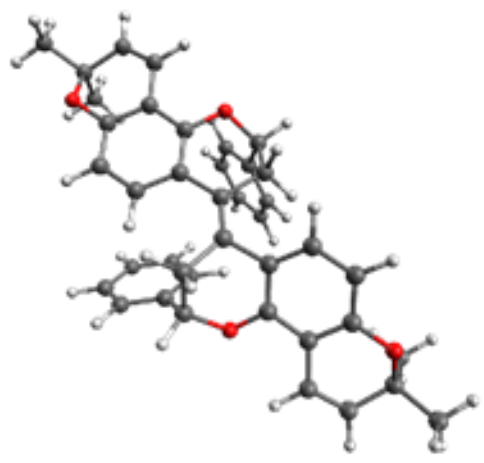

iv) 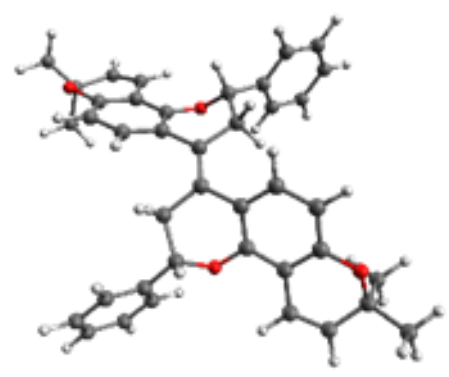

v) 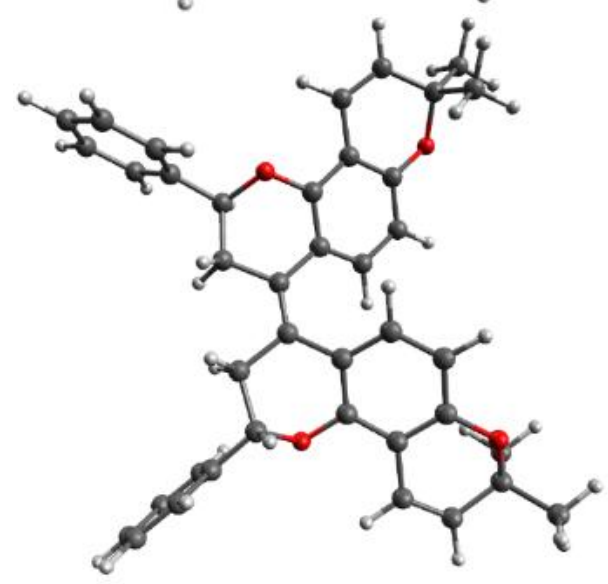

vi) 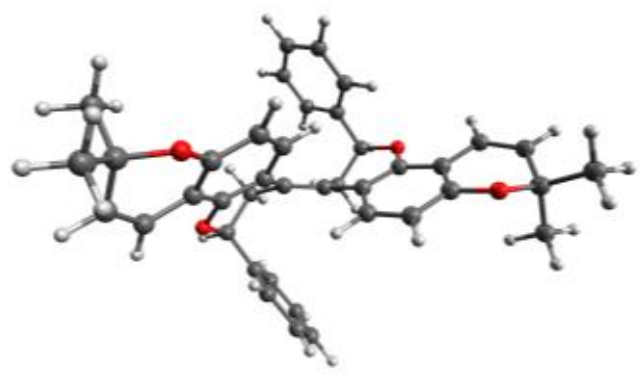

vii)

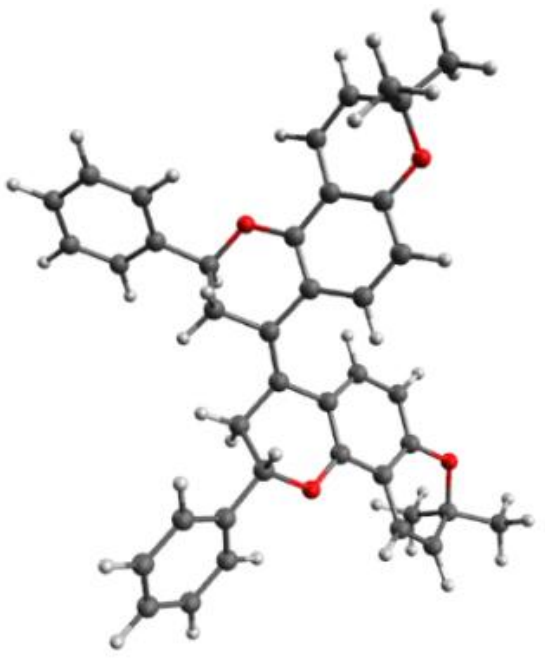

viii)

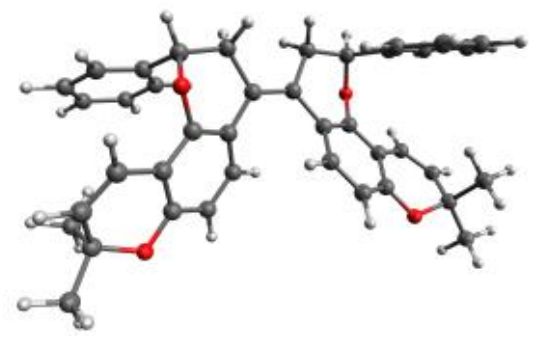

ix)

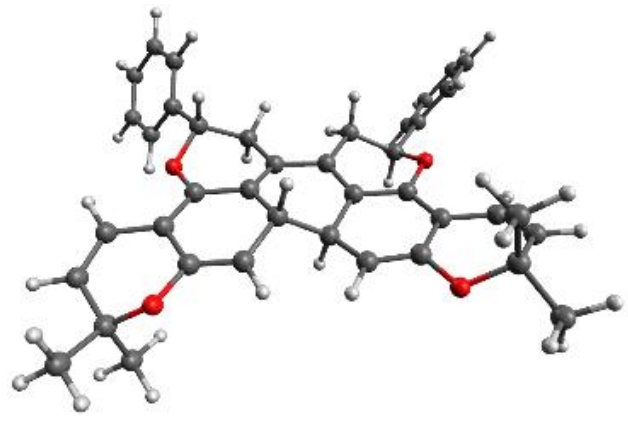

x)

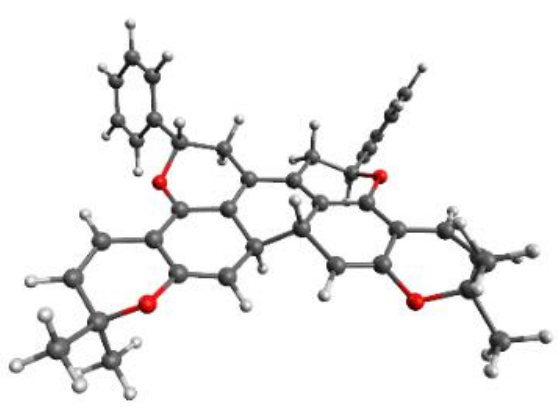

xi)

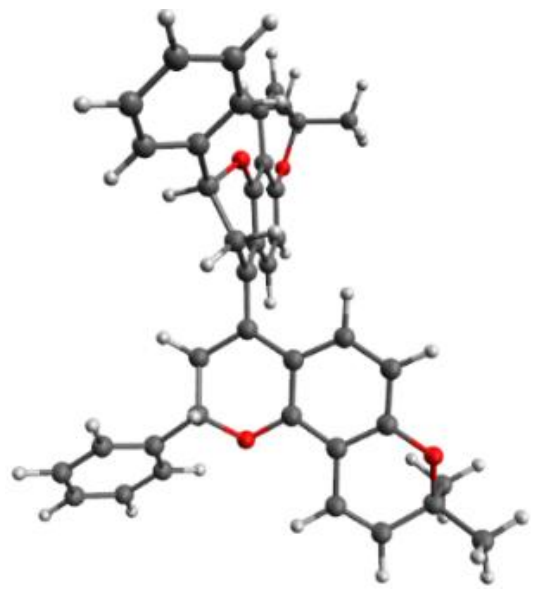

xii)

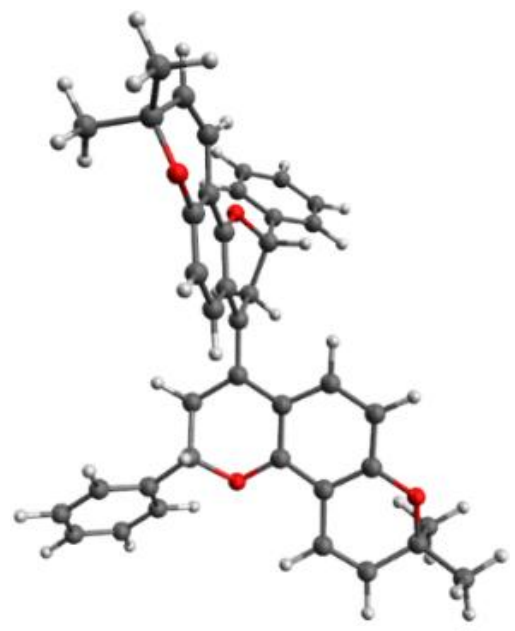

xiii)

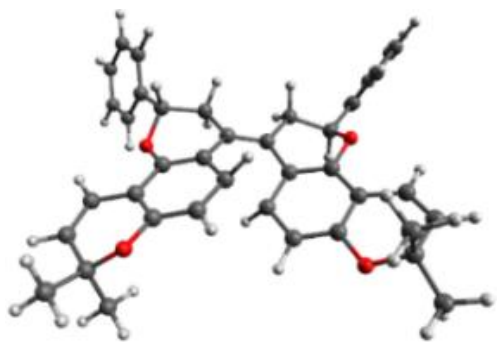

xiv)

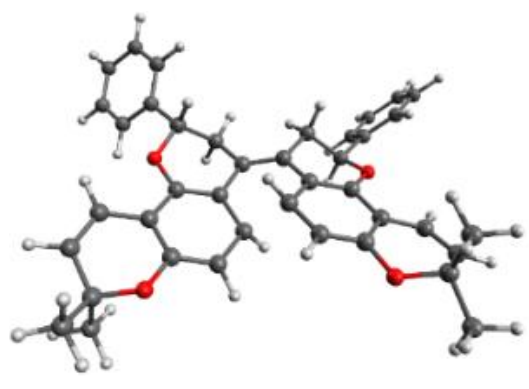

xv)

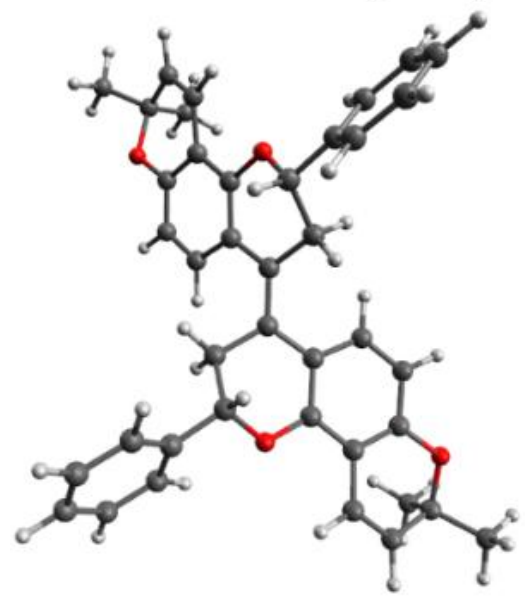

xvi)

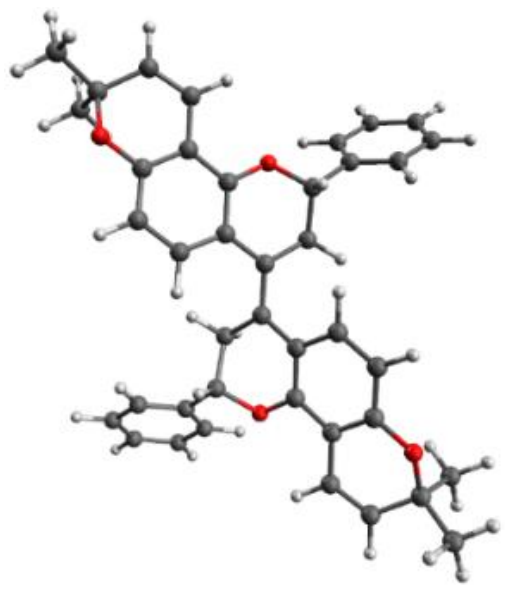

xvii)

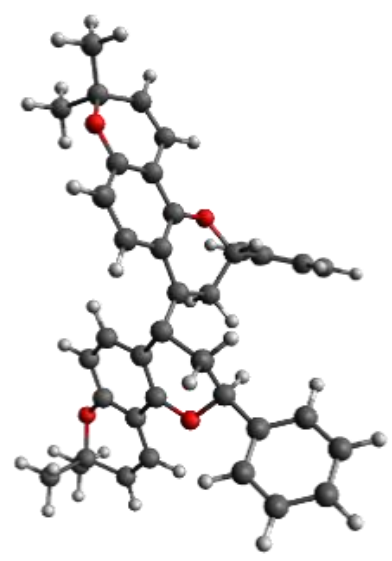

xviii)

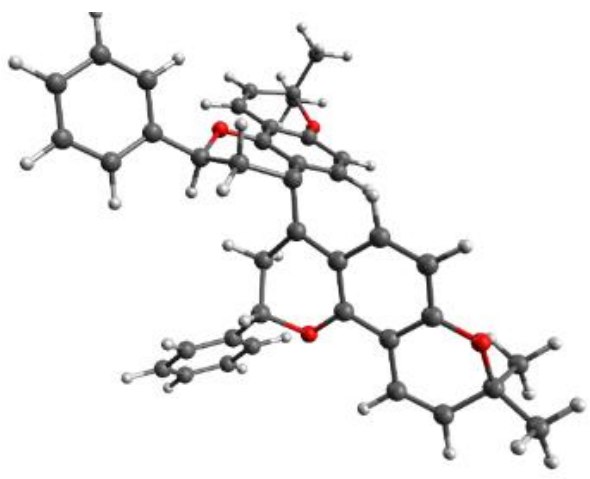

xix)

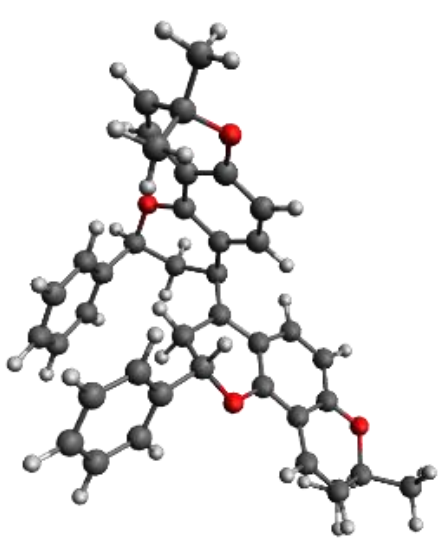

xx)

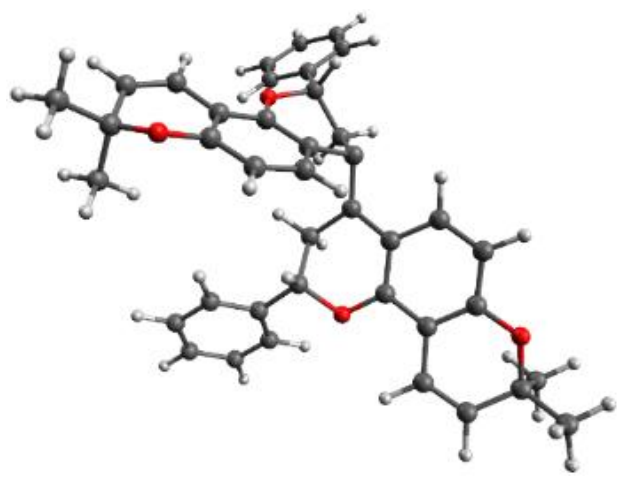

xxi)

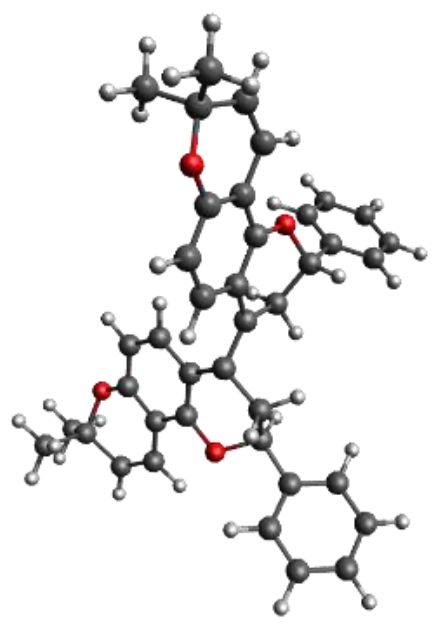

xxii)

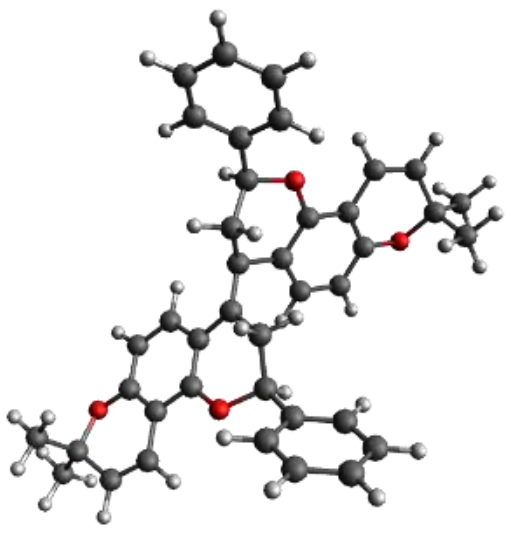

xxiii)

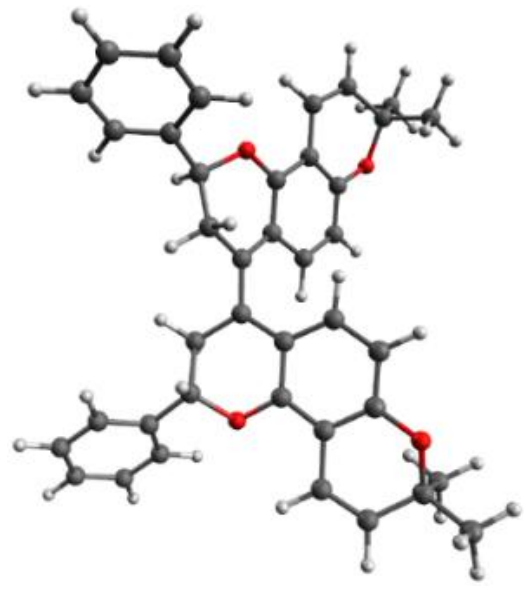

xxiv)

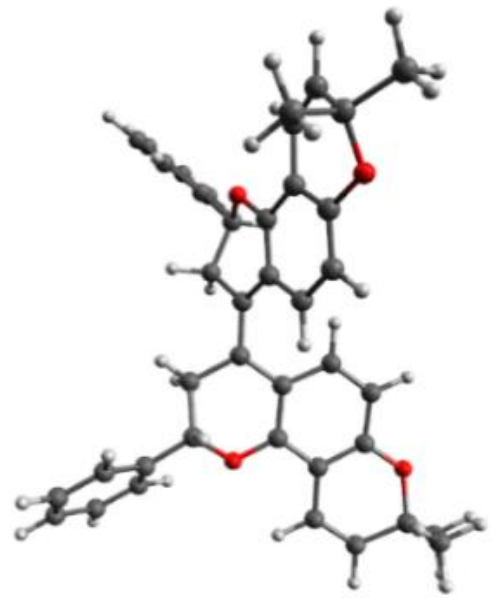

xxv) 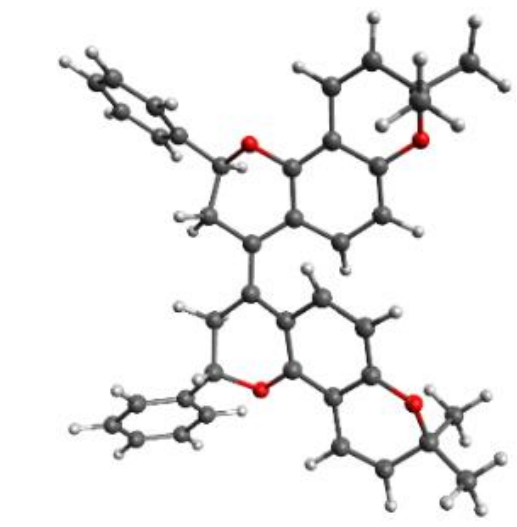 xxvi) 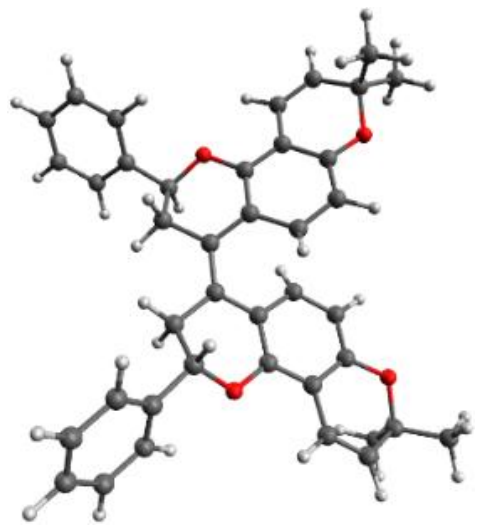 xxvii) 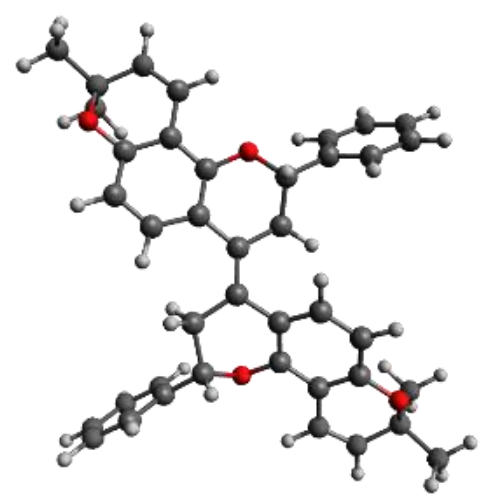

xxviii) 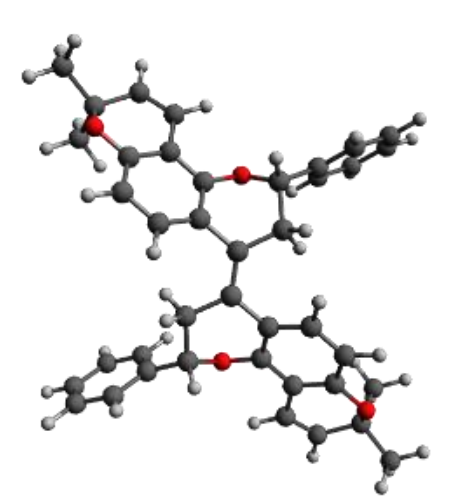 xxix) 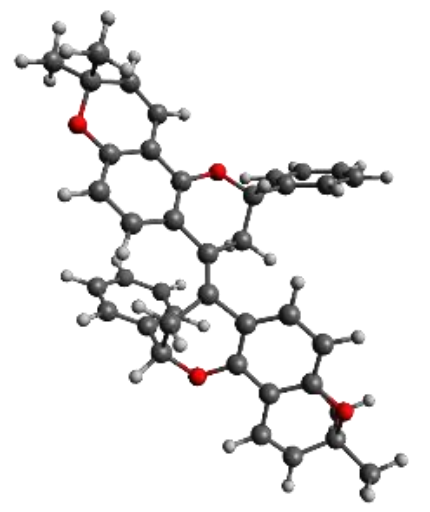 xxx) 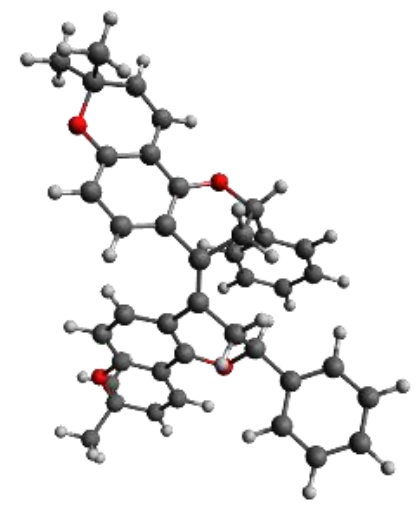

xxxi) 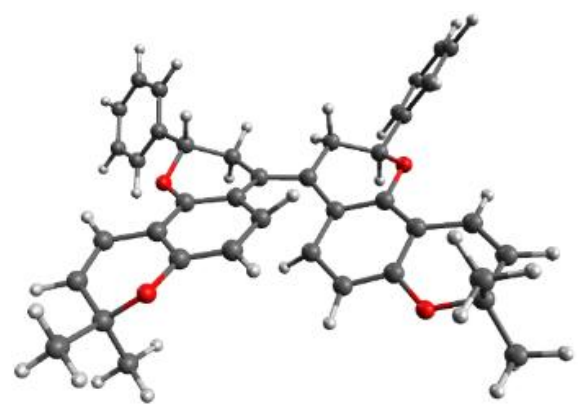 xxxii) 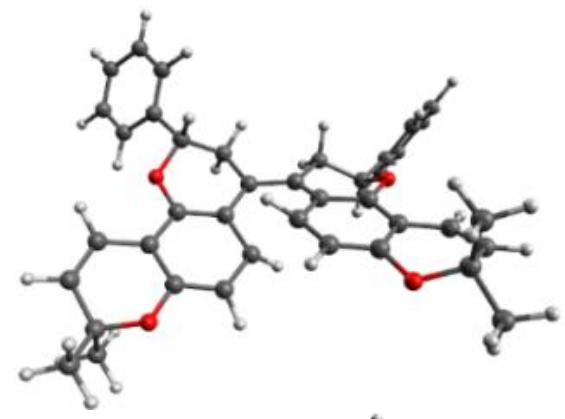 xxxiii) 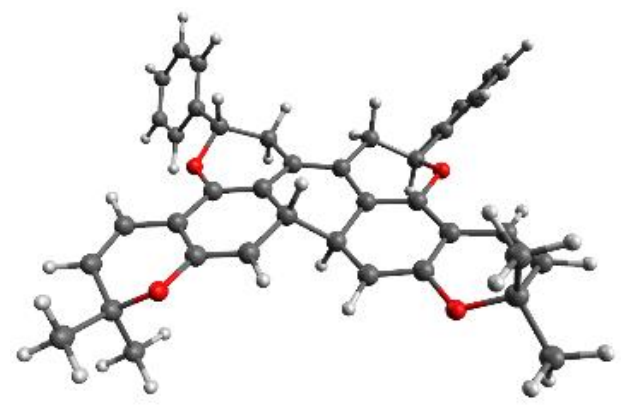

xxxiv) 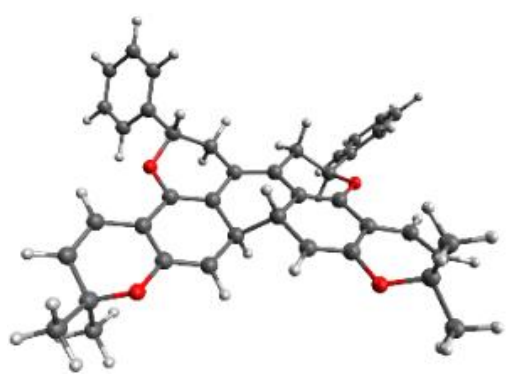 xxxv) 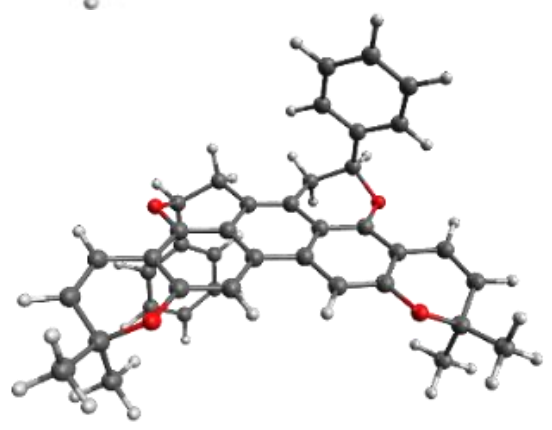 xxxvi) 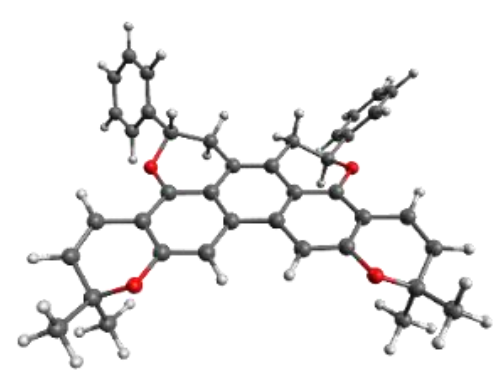

Figure S37. Graphical representation of i) (S$_0$)$_{(P,P)\text{-}(E)\text{-}\mathbf{2}\text{-ax}}$; ii) (S$_0$)$_{(M,M)\text{-}(E)\text{-}\mathbf{2}\text{-eq}}$; iii)(S$_0$)$_{(M)\text{-}(E)\text{-}\mathbf{2}\text{-ax}}$; iv) (S$_0$)$_{(P,P)\text{-}(E)\text{-}\mathbf{2}\text{-eq}}$; v) (S$_0$)$_{(P)\text{-}(Z)\text{-}\mathbf{2}\text{-eq}}$; vi) (S$_0$)$_{(P)\text{-}(Z)\text{-}\mathbf{2}\text{-ax}}$; vii) (S$_0$)$_{(M)\text{-}(Z)\text{-}\mathbf{2}\text{-eq}}$; viii) (S$_0$)$_{(M)\text{-}(Z)\text{-}\mathbf{2}\text{-ax}}$; ix) (S$_0$)$_{R,R\text{-}\mathbf{2'}\text{-eq}}$; x) (S$_0$)$_{S,S\text{-}\mathbf{2'}\text{-eq}}$; xi) (S$_0$)$_{TS}$; xii) (S$_0$)$_{TS\text{-}CC}$; xiii) (S$_0$)$_{(P)\text{-}TS}$; xiv) (S$_0$)$_{(M)\text{-}TS}$; xv) (S$_1$)$_{(E)\text{-}\mathbf{2}}$; xvi) (S$_1$)$_{(E)\text{-}\mathbf{2}\text{-}CC}$; xvii) (S$_1$/S$_0$)$_{pyr\text{-}equatorial\text{-}top}$; xviii) (S$_1$/S$_0$)p$_{yr}$; xix) (S$_1$/S$_0$)p$_{yr\text{-}axial\text{-}top\text{-}CC}$; xx) (S$_1$/S$_0$)pyr$_{\text{-}CC}$; xxi) (S$_1$)$_{pyr}$; xxii) (S$_1$)$_{pyr\text{-}CC}$; xxiii) (S$_1$)$_{TS}$; xxiv) (S$_1$)$_{TS\text{-}CC}$; xxv) (S$_1$)$_{(P)\text{-}(Z)\text{-}\mathbf{2}}$; xxvi) (S$_1$)$_{(M)\text{-}(Z)\text{-}\mathbf{2}}$; xxvii) (S$_0$)$_{(M)\text{-}(E)\rightarrow(P)\text{-}(E)\text{-}TS1}$; xxviii) (S$_0$)$_{(M)\text{-}(E)\rightarrow(P)\text{-}(E)\text{-}I}$; xxix) (S$_0$)$_{(M)\text{-}(E)\rightarrow(P)\text{-}(E)\text{ -}TS2}$; xxx) (S$_0$)$_{(M)\text{-}(Z)\rightarrow(P)\text{-}(Z)\text{-}TS}$; xxxi) (S$_1$/S$_0$)$_{(P)}$; xxxii) (S$_1$/S$_0$)$_{(M)}$; xxxiii) (S$_1$)$_{R,R\text{-}\mathbf{2'}}$; xxxiv) (S$_1$)$_{S,S\text{-}\mathbf{2'}}$; xxxv) (S$_0$)$_{\mathbf{3}\text{-ax}}$; xxxvi) (S$_0$)$_{\mathbf{3}\text{-eq}}$

Table S3. Calculated Gibbs Energies

Geometry	S$_0$ energy at ωB97X-D4/cc-pVDZ + C-PCM CH_2Cl_2 level (Eh) at 25°C	Relative Gibbs Energies (kJ/mol) at 25°C
(S$_0$)$_{(P,P)\text{-}(E)\text{-}\mathbf{2}\text{-ax}}$	-1847.245307	13.3900500
*(S$_0$)$_{(M,M)\text{-}(E)\text{-}\mathbf{2}\text{-eq}}$	-1847.248442	5.1591075
*(S$_0$)$_{(M,M)\text{-}(E)\text{-}\mathbf{2}\text{-eq-II}}$	-1847.246669	9.8141190
*(S$_0$)$_{(M,M)\text{-}(E)\text{-}\mathbf{2}\text{-eq-III}}$	-1847.246394	10.5361315
(S$_0$)$_{(M,M)\text{-}(E)\text{-}\mathbf{2}\text{-ax}}$	-1847.238722	30.6789675
(S$_0$)$_{(P,P)\text{-}(E)\text{-}\mathbf{2}\text{-eq}}$	-1847.237274	34.4806915
(S$_0$)$_{(P)\text{-}(Z)\text{-}\mathbf{2}\text{-eq}}$	-1847.239944	27.4706065
(S$_0$)$_{(P)\text{-}(Z)\text{-}\mathbf{2}\text{-ax}}$	-1847.250407	0.0000000
(S$_0$)$_{(M)\text{-}(Z)\text{-}\mathbf{2}\text{-eq}}$	-1847.247207	8.4016000

$(S_0)_{(M)\text{-}(Z)\text{-}\mathbf{2}\text{-ax}}$	-1847.238315	31.7475460
$(S_0)_{R,R\text{-}\mathbf{2}'\text{-eq}}$	-1847.199499	133.6589540
$(S_0)_{S,S\text{-}\mathbf{2}'\text{-eq}}$	-1847.197245	139.5768310
$(S_0)_{TS}$	-1847.237012	35.1685725
$(S_0)_{TS\text{-}CC}$	-1847.238922	30.1538675
$(S_0)_{(P)\text{-}TS}$	-1847.237688	33.3937345
$(S_0)_{(M)\text{-}TS}$	-1847.242160	21.6524985
$(S_1)_{(E)\text{-}\mathbf{2}}$	-1847.248598	4.7495295
$(S_1)_{(E)\text{-}\mathbf{2}\text{-}CC}$	-1847.238432	31.4403625
$(S_1/S_0)_{pyr\text{-equatorial-top}}$	-1847.240260	26.6409485
$(S_1/S_0)p_{yr}$	-1847.242326	21.2166655
$(S_1/S_0)p_{yr\text{-axial-top-CC}}$	-1847.234992	40.4720825
$(S_1/S_0)pyr_{\text{-}CC}$	-1847.248283	5.5765620
$(S_1)_{pyr}$	-1847.241207	24.1546000
$(S_1)_{pyr\text{-}CC}$	-1847.247786	6.8814355
$(S_1)_{TS}$	-1847.228424	57.7163665
$(S_1)_{TS\text{-}CC}$	-1847.244922	14.4008675
$(S_1)_{(P)\text{-}(Z)\text{-}\mathbf{2}}$	-1847.240476	26.0738405
$(S_1)_{(M)\text{-}(Z)\text{-}\mathbf{2}}$	-1847.247830	6.7659135
$(S_0)_{(M)\text{-}(E)\rightarrow(P)\text{-}(E)\text{-}TS1}$	-1847.234065	42.9059210
$(S_0)_{(M)\text{-}(E)\rightarrow(P)\text{-}(E)\text{-}I}$	-1847.239818	27.8014195
$(S_0)_{(M)\text{-}(E)\rightarrow(P)\text{-}(E)\ \text{-}TS2}$	-1847.236612	36.2187725
$(S_0)_{(M)\text{-}(Z)\rightarrow(P)\text{-}(Z)\text{-}TS}$	-1847.237505	33.8742010
$(S_1/S_0)_{(P)}$	-1847.198966	135.0583455
$(S_1/S_0)_{(M)}$	-1847.198890	135.2578835
$(S_1)_{R,R\text{-}\mathbf{2}'}$	-1847.201068	129.5395445
$(S_1)_{S,S\text{-}\mathbf{2}'}$	-1847.197925	137.7914910
$(S_0)_{\mathbf{3}\text{-ax}}$	-1846.687198	1478.7052295
$(S_0)_{\mathbf{3}\text{-eq}}$	-1846.685957	1481.9634750

Table S4. Calculated electronic energies

Geometry	S_0 energy at SF-TD-BHandHLYP/cc-pVDZ level (Eh)	S_1 energy at SF-TD-BHandHLYP/cc-pVDZ level (Eh)
(S_0)(M,M)-(E)*-**2**-eq	-1845.966378	-1845.802844
(S_0)(M,M)-(E)*-**2**-eq-II	-1845.965997	-1845.800882
(S_0)(M,M)-(E)*-**2**-eq-III	-1845.965607	-1845.799427
(S_0)*(P)-(Z)*-**2**-ax	-1845.974540	-1845.817106
(S_0)*(M)-(Z)*-**2**-eq	-1845.971790	-1845.807865
(S_0)*R*,*R*-**2'**-eq	-1845.922861	-1845.824336
(S_0)S,S-**2'**-eq	-1845.916001	-1845.816116
(S_0)TS	-1845.898566	-1845.832822
(S_0)TS-CC	-1845.899336	-1845.833849
(S_0)*(P)*-TS	-1845.876539	-1845.843134
(S_0)*(M)*-TS	-1845.879141	-1845.837026
(S_1)*(E)*-**2**	-1845.938519	-1845.833857
(S_1)*(E)*-**2**-CC	-1845.924888	-1845.837829
(S_1/S_0)*pyr*-equatorial-top	-1845.834375	-1845.834611
(S_1/S_0)pyr	-1845.835574	-1845.835574
(S_1/S_0)pyr-axial-top-CC	-1845.841657	-1845.841657
(S_1/S_0)pyr-CC	-1845.842951	-1845.842970
(S_1)*pyr*	-1845.878659	-1845.843821
(S_1)pyr-CC	-1845.874705	-1845.847694
(S_1)TS	-1845.905874	-1845.841525
(S_1)TS-CC	-1845.914908	-1845.840637
(S_1)*(P)-(Z)*-**2**	-1845.888278	-1845.858254
(S_1)*(M)-(Z)*-**2**	-1845.894494	-1845.856202
(S_1/S_0)*(P)*	-1845.855090	-1845.855446
(S_1/S_0)*(M)*	-1845.852808	-1845.853571
(S_1)*R*,*R*-**2'**	-1845.904530	-1845.856058
(S_1)S,S-**2'**	-1845.900781	-1845.855212

Table S5. Relative electronic energies

Geometry	θ (°) $(\mathrm{C_{4a}} - \mathrm{C_4} - \mathrm{C_{4'}} - \mathrm{C_{4a'}})$	S_0 energy at SF-TD-BHandHLYP/cc-pVDZ level (kJ/mol)	S_1 energy at SF-TD-BHandHLYP/cc-pVDZ level (kJ/mol)
(S_0)(M,M)-(E)*-**2**-eq	171.9	21.42933	450.7878
(S_0)(M,M)-(E)*-**2**-eq-II	172.9	22.42965	455.9391
(S_0)(M,M)-(E)*-**2**-eq-III	173.1	23.45359	459.7592
(S_0)*(P)-(Z)*-**2**-ax	7.0	0	413.343
(S_0)*(M)-(Z)*-**2**-eq	-10.9	7.220125	437.6052
(S_0)*R,R*-**2'**-eq	9.2	135.6832	394.3606
(S_0)S,S-**2'**-eq	-9.6	153.6941	415.9422
$(S_0)_{TS}$	93.2	199.4697	372.0806
$(S_0)_{TS\text{-}CC}$	-90.8	197.4481	369.3842
(S_0)*(P)*-TS	5.8	257.3016	345.0065
(S_0)*(M)*-TS	-1.3	250.4701	361.043
(S_1)*(E)*-**2**	136.8	94.57314	369.3632
(S_1)*(E)*-**2**-CC	-127.4	130.3613	358.9347
(S_1/S_0)*pyr*-equatorial-top	116.5	368.0032	367.3836
(S_1/S_0)pyr	124.2	364.8552	364.8552
(S_1/S_0)pyr-axial-top-CC	-111.2	348.8843	348.8843
(S_1/S_0)pyr-CC	-114.5	345.4869	345.437
(S_1)*pyr*	110.3	251.7356	343.2027
(S_1)pyr-CC	-111.1	262.1168	333.0342
$(S_1)_{TS}$	70.5	180.2826	349.2309
$(S_1)_{TS\text{-}CC}$	-64.2	156.5638	351.5623
(S_1)*(P)-(Z)*-**2**	20.2	226.4809	305.3089
(S_1)*(M)-(Z)*-**2**	-20.3	210.1608	310.6964
(S_1/S_0)*(P)*	26.2	313.616	312.6813
(S_1/S_0)*(M)*	-25.3	319.6074	317.6041
(S_1)*R,R*-**2'**	9.2	183.8113	311.0745
(S_1)S,S-**2'**	-9.6	193.6543	313.2957

*Three conformations were used to calculate the Boltzmann-averaged ECD spectrum of *(E)*-**2,** while only conformation I with the lowest energy was used as input in calculations at the SF-TD-BHandHLYP/cc-pVDZ level.

Cartesian nuclear coordinates of (S_0)$_{(P,P)\text{-}(E)}$-**2**-ax (**ω**B97X-D4/cc-pVDZ + C-PCM CH_2Cl_2)

C	-5.76443325205649	-0.09786347494786	0.27321155049039
C	-4.52443327160747	0.15955729417934	-0.33235825008681
C	-3.49808700923321	-0.78631821661914	-0.17974546923374
C	-5.96229467976033	-1.22882821592410	1.06054573879272
C	-4.93088516017649	-2.14768364022899	1.18415904496329
C	-3.70288232800892	-1.98187416596872	0.53033825265879
H	-6.91056706077245	-1.36607938138966	1.56871764012737
H	-5.06876845750213	-3.02124675023120	1.81479322757857
O	-2.32621752205377	-0.48095385398868	-0.79268404847638
C	-1.11735895957803	-1.23139165946296	-0.49597666937222
C	-2.59561496234242	-2.93923882802977	0.57611437223697
C	-1.28380771712546	-2.20507105093090	0.67372318842087
H	-0.41517965971350	-2.86080895101506	0.69623768078471
H	-1.27202125890848	-1.62102324856048	1.60359617736428
C	-2.74080966282585	-4.27764393894087	0.43107051051308
O	-6.77531593974869	0.80898805322719	0.19224967656794
C	-4.35174522999505	1.42703457554125	-1.01381332259059
C	-6.80809058019209	1.66973776596566	-0.98889418951651
C	-5.42216457097928	2.17753140199657	-1.29635636042107
H	-5.33218781384223	3.14765001228153	-1.77790799936823
H	-3.34636412055375	1.75322064357068	-1.25856617868551
C	-0.57570582753019	-1.81081849651368	-1.79316163478672
H	-0.40011666939144	-0.45827018505207	-0.18022546473121
C	0.02740608392031	-3.06537965743224	-1.87441962065840
C	-0.56286556758298	-0.98804114134069	-2.92504701151932
C	0.06076181152901	-1.39693991832015	-4.09587287096920
C	0.65323886031255	-3.47469087308739	-3.05034472615000
C	0.68377121957360	-2.64078524298641	-4.15983118288719
H	1.11574161787934	-4.45705681797925	-3.09172586103905
H	1.18181346164780	-2.95845106320396	-5.07177320809140
H	-1.04281191866472	-0.01485577444903	-2.88000997643881
H	0.06579669537874	-0.73921428649368	-4.96074140674071
H	0.01874330988918	-3.74814180685945	-1.03093508669782
C	-4.03382821218361	-4.94252062611809	0.03651252192681
C	-1.64574982642594	-5.25107578159294	0.43399276120412
C	-3.83872173145085	-5.57373071296457	-1.34278717059891
H	-4.86995144573424	-4.24830497005956	-0.01358092256150
H	-4.29609107288137	-5.73493797705359	0.75035585466940
O	-2.75217796892457	-6.52228463921809	-1.33021994151314
C	-1.70736547894324	-6.32178373598458	-0.48013889496468
C	-0.57076845839587	-5.23438888971278	1.33501517565270
C	-0.67906742408009	-7.27386501317276	-0.56579761635106
C	0.41326022131854	-7.15875189580273	0.30790858032323
C	0.45670528124841	-6.16311359417304	1.27989696931345
H	-0.56120434713970	-4.47959205871941	2.11535055657779
H	1.28198042151899	-6.14007175574185	1.98321064657077
O	1.41107280254652	-8.08203010924998	0.29604451182297
C	-0.72279671956376	-8.42186527312835	-1.45079343760644
C	0.37359610039126	-9.16903470123743	-1.61863831383675
C	1.67289723860948	-8.77300135583569	-0.96555467113752
C	2.46201020412277	-7.82449021798225	-1.87150817068629
C	2.50161690477331	-9.98761711006462	-0.57480757672570
H	-1.65889631516188	-8.66630487931861	-1.94143647703242
H	0.37329354278628	-10.05300367217867	-2.25091744448870
H	1.92784682306928	-10.64464347676001	0.08482223757327
H	3.41159346621048	-9.67291722403003	-0.05573727440677
H	2.78554717434369	-10.54796320889701	-1.47092302036384
C	-7.75326581918525	2.80202281882488	-0.61562572949388
C	-7.35292649032371	0.84842244434707	-2.16043157463635
H	-7.35463103330825	3.36815237064773	0.23090011335028
H	-8.73386012488556	2.40145879536844	-0.34274457741124
H	-7.87531255753503	3.47876154461452	-1.46693877798409
H	-6.69263098377567	-0.00128062645863	-2.36004552288218
H	-7.40094297871905	1.46573716198010	-3.06334023380799
H	-8.35648411029032	0.47509230400695	-1.93000977863268
H	1.87037400103930	-6.92802923446845	-2.08231606394039
H	2.69032338771146	-8.31615657027461	-2.82275789020936
H	3.39979438785850	-7.52934999073865	-1.38884128422066
C	-3.64867310476754	-4.55120734002370	-2.45098866778306

H	-4.71690575782896	-6.19059165673229	-1.58117012739667
C	-4.47608284832298	-3.42586538485225	-2.52002329047050
C	-2.68722454844030	-4.73916064968154	-3.44201572799958
C	-4.33205998267945	-2.50397500035160	-3.54970709970966
C	-3.37003862361478	-2.69905457759629	-4.53515712950871
C	-2.55385977746911	-3.82150032292562	-4.47922476231272
H	-1.79116286282934	-3.97730908121428	-5.23575387674821
H	-2.03359417430139	-5.60396392645685	-3.39627969266888
H	-5.23421163402269	-3.24538041842552	-1.76330294692757
H	-4.96826915731613	-1.62365218082921	-3.57338818533194
H	-3.25104824703258	-1.97479970656922	-5.33575457867270

Cartesian nuclear coordinates of (S_0)*(M,M)-(E)*-**2**-eq (***ω***B97X-D4/cc-pVDZ + C-PCM CH_2Cl_2)

C	-5.52158974320374	-0.54571675126001	3.01078787134833
C	-4.32705326947317	-0.51050900996974	3.74545133656116
C	-3.33136677060271	-1.45247877390202	3.44674657258250
C	-5.72449405800602	-1.49712135031145	2.01410445533622
C	-4.72372304786191	-2.41589056270564	1.74843507982036
C	-3.51567387314226	-2.44719989249064	2.46690055283999
H	-6.64659033169756	-1.48225168110715	1.44326377720598
H	-4.85155834016058	-3.10796719066964	0.92214222631590
O	-2.18378931451358	-1.32728046338215	4.16913125230679
C	-1.16848978658264	-2.32957017239962	3.99294134725612
C	-2.41369622255478	-3.35793610259873	2.14820212169582
C	-1.07619255675328	-2.77403056962976	2.52687014839552
H	-0.80112297739493	-1.89977721629731	1.91910214732177
H	-0.27177911345095	-3.50878508097282	2.45501156920996
C	-2.57311607746012	-4.63170537946255	1.69870578318467
O	-6.53710192388206	0.31069446753072	3.29478967193926
C	-4.22322066439588	0.44448102263904	4.83141019796645
C	-6.18491382664018	1.60507836113537	3.87930048659951
C	-5.12474793910326	1.42702156773248	4.93540487680390
H	-5.10143867843482	2.14097402869109	5.75453411384910
H	-3.42777293177544	0.32300744597626	5.55935062755993
C	0.13502245561012	-1.76172252166362	4.48804469069398
H	-1.44541942925673	-3.20493427456793	4.60310663339732
C	1.09075303546669	-2.62013147138161	5.03175483424987
C	0.43306518400689	-0.40617674011142	4.34992685584239
C	1.67257081979293	0.08085795079505	4.75274535890304
C	2.33178165157730	-2.13346300952944	5.42758052599717
C	2.62526527528015	-0.77972364434905	5.29018682998102
H	3.06739929057766	-2.81088804612803	5.85146431395438
H	3.59154548695198	-0.39655045354637	5.60505341266489
H	-0.31377055050974	0.26541429713408	3.93793420194933
H	1.89424273183575	1.13918938605365	4.64783902408319
H	0.86112753116775	-3.67758403449120	5.14609748950756
C	-3.86776327356748	-5.39424004585370	1.82323173206920
C	-1.49174231802135	-5.47378986043282	1.18231106533575
C	-3.57154241304552	-6.66866416842462	2.62432340493636
H	-4.28908915858860	-5.68895979851348	0.85111823736925
H	-4.62587420085023	-4.82838509205918	2.36827168290774
O	-2.61464878669116	-7.49626518870783	1.94267188467182
C	-1.58494793806656	-6.87429431490756	1.30350437607530
C	-0.41199314960806	-4.97121350330731	0.43500956093187
C	-0.61627985398813	-7.73303607548899	0.76297077641797
C	0.45165809473690	-7.16961021898662	0.04916501780429
C	0.55884589402125	-5.79071415914363	-0.11446475916136
H	-0.36974406025042	-3.90417951467166	0.24127902070230
H	1.37973332540392	-5.38567539357754	-0.69620425647636
O	1.44217763123412	-7.95247070096613	-0.45194911231156
C	-0.61401136053107	-9.16799781091624	0.97182112115870
C	0.22707383590815	-9.93591823809261	0.27075930255748
C	1.10170829339789	-9.33295641580966	-0.79838450634247
C	0.37297141510872	-9.31801782799661	-2.14412100383011
C	2.44252962684324	-10.04529211043373	-0.89828028225489
H	-1.27946287136069	-9.58807213189402	1.71886506002329
H	0.28266532149447	-11.01064706363949	0.42298585723604
H	2.95271971998286	-10.03439960575399	0.06902661000923
H	3.07811875779410	-9.55112548165192	-1.63882136505580
H	2.28981080484200	-11.08459984403742	-1.20524476648648

C	-7.48662743847182	2.12784712294259	4.46855193785314
C	-5.67572375527127	2.51085582703806	2.75559873047927
H	-7.84139585877102	1.46332252877376	5.26149120094865
H	-8.25394034378568	2.19228076702540	3.69161357615390
H	-7.32929922394211	3.12583332058843	4.88919345647307
H	-4.77803063025457	2.08085188438339	2.30035020324209
H	-5.41681962728208	3.49578675557589	3.15777787171838
H	-6.44504116148048	2.63242015662640	1.98536888677364
H	-0.55450237329814	-8.74220604397793	-2.06555457379784
H	0.11699296258005	-10.33980344329728	-2.44290374832855
H	1.00830913520678	-8.87030860920173	-2.91594402247342
C	-4.81437900536592	-7.48544521656458	2.85923689654152
H	-3.13610125294151	-6.37473600041583	3.59360514339825
C	-5.21564845070493	-8.47476952252023	1.96193213247414
C	-5.61652441692366	-7.19745259213376	3.96377349347317
C	-6.40526077422555	-9.16596313454998	2.17125787807741
C	-7.20501626331936	-8.87236900157683	3.27159467598884
C	-6.80820903987806	-7.88393995883788	4.16799464295771
H	-7.42372230714202	-7.65280816380717	5.03253662118105
H	-5.30524629285467	-6.42971095443587	4.66929124316556
H	-4.58729381277047	-8.70780378796850	1.10797147443378
H	-6.70747757530881	-9.93950556107221	1.47090707428247
H	-8.13211386540383	-9.41468794208707	3.43285215937279

Cartesian nuclear coordinates of (S_0)*(M,M)-(E)*-**2**-eq-II (***ω***B97X-D4/cc-pVDZ + C-PCM CH_2Cl_2)

H	1.49284821862063	-6.12746418302557	1.11934006927194
H	-2.70080524621838	0.70914129345019	2.15922761983373
H	0.00245804346472	-5.42403312321307	1.82674554644632
H	-4.15787881180002	4.58861293319521	1.96276947569389
C	0.44593479151661	-6.33370806779570	1.39260779259465
H	0.42754721784618	-7.12644516778727	2.15678323460938
O	-3.06207768863582	2.48467426941243	1.17414668294230
H	-1.35113220117141	-0.30387094556112	0.35365873665728
C	-2.21640355055431	1.34673723750390	1.39912961552929
H	-5.52739121382749	6.61098369076003	1.47076085655742
H	0.24777105387092	-3.36728860770531	-1.79119979483947
H	-0.60575853372784	0.15110945739114	3.22269854615053
C	-4.84784587178317	4.62603209076861	1.11812880954515
H	1.32602871058012	-7.73900337789029	-0.82406762736246
H	-1.26406189794161	-1.39492541393733	-1.64209857726302
C	-5.58643700356204	5.70993658483409	0.85398469604441
C	-2.03247960609608	0.52216494469702	0.12060223783543
C	-0.69887161900770	-3.42468828828456	-1.25116361237803
C	-0.18625086425923	1.09601424139476	2.86433311437641
H	-8.29333088177709	6.13665902984919	0.79285303086656
O	-0.27270739981135	-5.71502673130980	-0.83550830167614
C	-0.90206986717483	1.86211549414299	1.93911219831945
C	-4.12908142126818	2.33162840812951	0.34132693832262
C	-0.34522722533715	-6.77703010446702	0.15966571721754
C	-1.54637297220493	-2.32807599012550	-1.15050790017104
C	-4.99242091022970	3.43769643797260	0.27060300231344
H	-7.49900905926795	7.60004846402480	0.13820436712894
C	-1.08521785429429	-4.63645543298953	-0.67055875837861
C	1.05316263359955	1.53279838382238	3.33595335880165
C	0.29718699877149	-7.98079870508539	-0.51769178141183
H	1.60260482790105	0.92728576013866	4.06151589136247
H	-1.56067142819173	1.15634982211676	-0.64881170095034
C	-7.76076946473241	6.55279233994417	-0.07547274400588
H	0.32691869554847	-8.83212955485753	0.17901877762915
C	-0.36602319629218	3.07358744619127	1.49025011104468
C	-3.40065497697578	0.03802228477594	-0.29842521460178
C	-2.77045783729479	-2.38667782959923	-0.46092654714613
C	-4.36152619151579	1.16238486559937	-0.40390185372125
H	-0.92651574984280	3.67761426819723	0.77373659455570
C	-2.31580244246196	-4.75771700049672	-0.01312246125699
C	-6.49818185814782	5.74703443429757	-0.35484352212840
C	-3.73995715867055	-1.26600716604873	-0.41540856783765
C	1.58388149522798	2.74499531644093	2.88875174697445
H	-5.89418898779860	-0.99426008102295	-0.21980656902450
C	0.87023305139677	3.51419705397632	1.96642106505458

C	-1.78701189958826	-7.05605137625646	0.52838035152621
C	-3.14861285831108	-3.62988926828534	0.07571912015213
H	-0.28173826230067	-8.27139207847198	-1.40729423091162
C	-6.08500336926719	3.38508856271266	-0.60352721475755
H	-8.42831027054106	6.52730134202481	-0.94997836624345
O	-6.98003923048484	4.40746822848432	-0.66678476534665
H	2.55062285633627	3.09244284172752	3.26242665789718
C	-2.70458930760668	-6.08388145543950	0.47829271876332
H	-2.03551621236667	-8.06429074998100	0.87089170696782
C	-5.15707831448488	-1.78747818632047	-0.38273229143330
H	-5.38881027627486	7.30396584313938	-1.38183355553339
H	1.27916582145524	4.46540075435859	1.61524529009975
C	-5.74476605194352	6.28011986094121	-1.57548834787859
C	-5.44800982989230	1.17583142650099	-1.29675986462621
O	-4.34087208692464	-3.84351091173614	0.69755375984813
H	-5.42041491560408	-2.32228579868355	-1.31101388084566
C	-6.31118145813778	2.25924285018643	-1.40016476499647
H	-4.87133973284196	5.64653361126296	-1.79443408574128
H	-3.74085616334775	-6.25317667425428	0.77508062467898
C	-5.26963267417501	-2.75450755759840	0.79938549927595
H	-6.40693162531424	6.29315395846897	-2.45558811524386
H	-5.59813834348408	0.31833962646257	-1.95511772202480
H	-7.15036984702438	2.25589919973880	-2.09767261400069
H	-5.03750353634236	-2.20044490123440	1.72576077542077
C	-6.66010080919027	-3.34276751677857	0.90126398817092
H	-7.41754657912227	-1.67215950005780	2.04296271280926
H	-6.19728884426037	-5.12676929605592	-0.21828573930865
C	-7.66236761570182	-2.62463409840760	1.56265187803353
C	-6.97776674257289	-4.56161615656912	0.29448669215260
C	-8.96808116324192	-3.11517509503176	1.61287263076785
C	-8.28297281728512	-5.05561390444630	0.34927740701673
C	-9.28200112460890	-4.33413453382724	1.00604809756229
H	-9.74305424332995	-2.54624657715159	2.13317973372989
H	-8.52033165368400	-6.01132883593112	-0.12579053458231
H	-10.30334556500461	-4.72144441531497	1.04705214907521

Cartesian nuclear coordinates of (S_0)*(M,M)-(E)*-**2**-eq-II (**ω**B97X-D4/cc-pVDZ + C-PCM CH_2Cl_2)

H	1.41044296017474	-6.14940033198047	1.22720558111327
H	-2.65597391441041	0.73792140366982	2.12682069432649
H	-0.07881107893906	-5.41169042002673	1.90144772906954
H	-3.76325010707519	4.88184426415666	1.46212043878810
C	0.35581488034571	-6.33414220053795	1.48571979619492
H	0.31135232076925	-7.11681869231958	2.25914936676929
O	-3.03117566336014	2.51204641811496	1.14650680264586
H	-1.31536139179991	-0.27839662232149	0.32741599702142
C	-2.17743638513428	1.37714241079467	1.36473195012330
H	-5.58235106930684	6.58384519058657	1.53243860735432
H	0.25751176351491	-3.40734622120147	-1.74276654072647
H	-0.63480502016470	0.29186077023020	3.31860830530596
C	-4.71615019960493	4.72819693428453	0.95307974017034
H	1.24806389665931	-7.78203191288380	-0.69790124995754
H	-1.22413522739205	-1.40775851130040	-1.64508046700877
C	-5.69338053904489	5.64152896914704	0.98882757806140
C	-1.99026038481712	0.55105289652603	0.08761780333081
C	-0.69799039036566	-3.44101218144785	-1.21653711494372
C	-0.18807219793984	1.19832378534671	2.89933353354450
H	-7.53707447675871	3.69787222977094	1.60720118636561
O	-0.31603626353755	-5.73150020255110	-0.76156262215359
C	-0.86561068825706	1.90243890913599	1.90004935482004
C	-4.07778514988054	2.37444561496912	0.28626022121175
C	-0.42272128708618	-6.77951583541455	0.24551080129880
C	-1.52848567299734	-2.32913050803437	-1.14442538121377
C	-4.92476787519132	3.49123147658841	0.19265115874888
H	-8.16208902574122	5.27708682045056	2.15656346540416
C	-1.11307429666919	-4.63762832529438	-0.62435886837519
C	1.04884604299329	1.64833260782219	3.36664543697832
C	0.21033777994561	-8.00250439661503	-0.40576687179755
H	1.56885355891378	1.09166294519106	4.15066166722785
H	-1.50967418229946	1.18021997763215	-0.68045381376612
C	-7.97477514815071	4.65062374472416	1.27064868292768

H	0.21418040919945	-8.84492874083814	0.30234706828670
C	-0.29488344279760	3.06574692587442	1.37211671884039
C	-3.35784924211608	0.07729405278926	-0.34311342689102
C	-2.76261409790450	-2.35805990310785	-0.47154859732274
C	-4.30120676941041	1.21337345297004	-0.47380720431633
H	-0.82648585958147	3.62213781754472	0.59678643187678
C	-2.35516707791014	-4.72942787800370	0.01628304699145
C	-7.02116024698860	5.36950195254821	0.31264748210705
C	-3.71445555483086	-1.22198554160849	-0.45424459941861
C	1.61408552056110	2.81140412783099	2.83992823487447
H	-5.86724608623671	-0.91195958769785	-0.30438096678202
C	0.93830674369864	3.51926684400898	1.84211464453987
C	-1.87483099054374	-7.02960308086242	0.59403704457548
C	-3.16916578962962	-3.58624546746218	0.07833874900383
H	-0.35857790352859	-8.29513008903558	-1.30113082377948
C	-5.99638828177345	3.45044569889693	-0.70818287091150
H	-8.93473379624332	4.44651876599775	0.77051496840013
O	-6.82091610267809	4.52313671479963	-0.85598533545074
H	2.57860990462285	3.16931036759861	3.20944745039025
C	-2.77439280735414	-6.04243465194283	0.51803551323852
H	-2.14649493939136	-8.02924135694946	0.94411040651413
C	-5.14025242770716	-1.71993653956955	-0.43912841740877
H	-7.85644216544760	7.34515082369073	0.58279180330018
H	1.37477273011933	4.43216494144294	1.42811665182827
C	-7.63887955895558	6.64731114994895	-0.23975656196669
C	-5.34889464248934	1.24796289925446	-1.41048199666578
O	-4.37238874593867	-3.76884933302023	0.68891277590499
H	-5.39455285487536	-2.26749056640644	-1.36241838507089
C	-6.19934434044878	2.33984567943581	-1.53159977192410
H	-6.94608589018558	7.13364644132370	-0.94272989712760
H	-3.81801543944854	-6.19040483476876	0.80013809104885
C	-5.28660927111442	-2.66449647342077	0.75772675212224
H	-8.57763559351068	6.41793118219074	-0.76600749878595
H	-5.48744281981458	0.39291357169849	-2.07494091479255
H	-7.01315980220989	2.35258418107280	-2.25852997217212
H	-5.06009790592077	-2.09780773153769	1.67782956427022
C	-6.68638094450185	-3.23190790896514	0.85004247065569
H	-7.43398312298300	-1.53797194739983	1.96333915065874
H	-6.23498849548885	-5.03587314915861	-0.24202843167778
C	-7.68610381318651	-2.49306033610895	1.49210675842005
C	-7.01341852699806	-4.45432920625136	0.25534169817767
C	-8.99829739122191	-2.96678850918984	1.53543797600740
C	-8.32506067079847	-4.93152804357345	0.30338564406647
C	-9.32135310314323	-4.18960230502835	0.94137791688981
H	-9.77120800107364	-2.38181024977103	2.04079485648876
H	-8.56961846247365	-5.89035372600636	-0.16167079050206
H	-10.34768789673859	-4.56399144044587	0.97774362462843

Cartesian nuclear coordinates of $(S_0)_{(M,M)\text{-}(E)\text{-}\mathbf{2}\text{-ax}}$ (ωB97X-D4/cc-pVDZ + C-PCM CH_2Cl_2)
Cartesian nuclear coordinates of $(S_0)_{(P,P)\text{-}(E)\text{-}\mathbf{2}\text{-eq}}$ (ωB97X-D4/cc-pVDZ + C-PCM CH_2Cl_2)
Cartesian nuclear coordinates of $(S_0)_{(P)\text{-}(Z)\text{-}\mathbf{2}\text{-eq}}$ (ωB97X-D4/cc-pVDZ + C-PCM CH_2Cl_2)
Cartesian nuclear coordinates of $(S_0)_{(P)\text{-}(Z)\text{-}\mathbf{2}\text{-ax}}$ (ωB97X-D4/cc-pVDZ + C-PCM CH_2Cl_2)

H	1.71039277913376	-8.28961119899800	-0.69357510429847
H	1.55681387631513	-7.97461578793132	1.06346580780447
H	4.28239011253612	-7.90504585285325	-0.39576356629288
H	4.02929386756063	-7.52077790353513	1.33278452092505
C	1.56883740910999	-7.50578926305008	0.06675954401069
C	4.05026018595818	-7.09904034879134	0.31660075152735
H	0.59641195197989	-7.02153938234089	-0.11329870789174
C	2.70050810923340	-6.47913692785276	-0.02189773502334
H	4.84370886858017	-6.33850453852103	0.26179988639632
H	3.29876254596377	-6.31926011381864	-2.17583163853985
C	2.71677998595929	-5.82973719781729	-1.39007809717078
O	2.48836473263453	-5.47460585348542	1.01223007409687
C	1.54168683023576	-4.52480841093994	0.77864219332353
H	-6.46946190698470	5.08394559507179	-1.66345742915222
C	1.99873945762147	-4.72644233651480	-1.62877660328751
H	1.08671632379538	-4.34218622051402	2.87670968274223
C	0.88165952978302	-3.96292965287444	1.87431532258029
C	1.25603211480244	-4.10503198997418	-0.52728554336266

C	-5.60795207882611	4.44839905883082	-1.44247106892175
H	-5.17817980462593	5.41462426530553	0.44357130704763
H	1.96505486078742	-4.26159261254199	-2.61542681842145
C	-4.88354432006934	4.63317708829816	-0.26208836420727
H	-5.78130104298685	3.30265596544230	-3.26910571620162
C	-0.03985933478841	-2.94422232890457	1.65530938182772
C	-5.22292514257967	3.45036726963223	-2.34090726318054
C	0.30348205424491	-3.09074020099031	-0.71372992699317
H	-0.60474649404185	-2.54784694165865	2.50131807668354
C	-0.30703166542971	-2.44119642941919	0.37313142211521
O	0.02141753020346	-2.77881400024158	-2.00839247673503
H	-2.52706960014687	-2.96133364840563	-0.94813430984972
C	-3.78210698401350	3.82309616318997	0.02148873391879
C	-4.11778559784834	2.64502913098543	-2.05944511117302
H	-1.62779126969520	-2.50020128842893	-3.14988477938101
H	-3.21336600403527	3.97003160303177	0.94225167546389
C	-2.14056748313141	-1.94489636253280	-1.12470524460643
C	-1.14774031491184	-1.98729309743302	-2.30334935799185
C	-1.35666787387269	-1.43894137308990	0.06764217633117
C	-3.39289832429969	2.82391614624858	-0.87653649442925
H	-3.81121997373543	1.86906780380804	-2.76648220719234
H	-2.97849411331695	-1.30882076138019	-1.42964675107818
C	-1.48388572247853	-0.18583495063496	0.56480656111613
H	0.46535225694945	-0.90327324008436	2.51889794365989
C	-0.71685457074336	-0.60355266680845	-2.74679205608890
C	0.47578555810460	0.14230520328491	2.23331185730713
C	-0.46586956650382	0.61441816927209	1.30338448833001
H	0.96813690685346	-0.47056850643896	-1.39568420996871
C	-2.24158299818397	1.89461082867228	-0.56690410940997
C	1.42512261492507	0.96149494078715	2.83213187310687
H	-3.13671546277825	1.02990183197708	1.17909404403429
C	-2.68152688614482	0.67856237191729	0.23650882935794
H	-2.30416177355435	-0.43669529964545	-4.20161440240440
H	2.13765606831638	0.56306712937297	3.55642411026382
C	-0.41405666447963	1.99889763706266	1.01514417771475
C	0.37983950003392	0.03918760843203	-2.16093199884740
O	-1.25566659625169	2.64246983809114	0.15739949387328
C	-1.45162446243159	0.06369471550589	-3.73201779920500
C	1.46162181957342	2.31976207030657	2.50951333285990
C	0.54210248890103	2.84859826243975	1.59974862171809
H	-1.78717845252296	1.55920534824411	-1.50883057100121
O	2.40950258115015	3.07336144808439	3.11888833119712
H	1.36943017050185	5.01360603236542	4.60661150687266
C	0.62418904619335	4.27995629550427	1.29114192298430
H	-0.08296875369787	4.70353574701732	0.57749432523087
H	-3.46059747976498	0.14804280488133	-0.32026930194923
C	0.72306044500160	1.33703101180510	-2.54280716708044
C	-1.10718609068540	1.36129108611185	-4.11880973192342
C	1.55579589672041	5.03725376978271	1.87677179289628
C	2.38677367476541	5.18668293140333	4.22392583030347
C	2.56168253515619	4.50325628210451	2.86702282519953
H	3.11396181634184	4.78392609658822	4.94604766648965
H	1.57360803101569	1.83331430000916	-2.06817156357147
C	-0.01992321551226	2.00293157875972	-3.52109236758743
H	1.63554812274901	6.10613137053273	1.65876939272611
H	-1.69033347679682	1.87177808623329	-4.88973719294333
H	2.54832332146175	6.27170915754485	4.12816491726324
H	0.24895537598280	3.01987566646423	-3.81823098199822
C	3.98248366162537	4.68384556620976	2.33024609641165
H	4.09596501346130	4.15294330463462	1.37281237029925
H	4.71187180832297	4.28203004287304	3.05065220359864
H	4.19675965732436	5.75189806733129	2.16922869775159

Cartesian nuclear coordinates of $(S_0)_{(M)\text{-}(Z)\text{-}\mathbf{2}\text{-eq}}$ (**ω**B97X-D4/cc-pVDZ + C-PCM CH_2Cl_2)

H	2.49590000577861	-10.89259347234773	-0.21861092571498
H	3.40358283374851	-9.54389741927741	0.50143799267696
H	2.18950330497070	-9.52827720774422	-2.42189381284071
H	3.18217432816773	-8.25901123668385	-1.65981242369092
C	2.40357386156683	-9.95826697910081	0.34367824815162
C	2.16145378837920	-8.63463352779462	-1.78978893934050

H	1.95335217995543	-10.17535310165641	1.31644377882510
C	1.54207763975111	-8.97215387334603	-0.43142450367762
H	1.56190219428944	-7.87368866549632	-2.29903941273778
H	-0.00907196887558	-10.54574219415424	-0.74109657004354
C	0.13240692520056	-9.47699064505374	-0.60329446411866
O	1.54758796291848	-7.75653752003694	0.38279630192278
C	0.58513662562318	-6.83077652202665	0.13353586443759
H	-5.17287306512221	3.77574287598390	-2.79225760218714
C	-0.89560874526563	-8.62160752634311	-0.62556998335068
H	1.82745937281998	-5.23621217151890	0.85453185865537
C	0.84889620159191	-5.50372626697666	0.47088137689999
C	-0.65865327400240	-7.21121446384068	-0.38768393259533
C	-4.63342907966181	2.95832434367934	-2.32272733672812
H	-5.39743448417430	3.37342321980015	-0.35445909978609
H	-1.91375499580511	-8.96009996196569	-0.78727952337630
C	-4.75910771889831	2.73148364356481	-0.95491820851439
H	-3.69660143331499	2.31387485763993	-4.15065484214133
C	-0.13578623341081	-4.55288102758114	0.28069041614152
C	-3.80710064924460	2.13772601584467	-3.08456315282283
C	-1.63361893667370	-6.21779891870338	-0.57671057181704
H	0.07892518944405	-3.51440621840254	0.50956260457299
C	-1.40886204978533	-4.87639361717643	-0.21970720031733
O	-2.79940214430693	-6.65738174583626	-1.13142089087903
H	-4.15379818996057	-3.66554130043740	-1.85437246740394
C	-4.06774010170673	1.68726631884954	-0.34978381125780
C	-3.11000427105931	1.09754748278412	-2.47898556315053
H	-4.40512738463388	-5.61806466382960	-0.35176406019888
H	-4.15654293215182	1.51514418733575	0.71861187183163
C	-3.30241127469237	-4.32413307631854	-1.67374912946486
C	-3.86247501456875	-5.70535833158650	-1.30683387737265
C	-2.42934566075129	-3.86746030915402	-0.53332022537052
C	-3.23846854365129	0.86248645922494	-1.11023994701663
H	-2.45596168450806	0.46383481072416	-3.07439587782598
H	-2.73827083249580	-4.42470945956022	-2.61268646667460
C	-2.63109154690744	-2.69466629806780	0.12110110176208
H	-2.03058253753848	-4.28789166576576	2.26692488452573
C	-4.78744467807435	-6.23721917909251	-2.36875985069877
C	-1.84457282016413	-3.23705970758412	2.46208911807733
C	-2.07114030376689	-2.31578776122008	1.42500840268684
H	-3.22481243585186	-7.11768340142647	-3.55074454134859
C	-2.54103688643011	-0.32006456008489	-0.49279919887140
C	-1.41436430332561	-2.84895246817027	3.71683138768119
H	-4.32528095173703	-1.33080935225128	0.14202703690345
C	-3.43811251874959	-1.56481031976565	-0.46493017318250
H	-6.55192997102471	-5.47571441426577	-1.40170336315487
H	-1.24753558952671	-3.57294478573218	4.50698258305267
C	-1.89341248673090	-0.95434309940722	1.72913726639149
C	-4.28896040212668	-6.91256073250931	-3.48322155809623
O	-2.13956132316263	0.04800749769860	0.83743317769680
C	-6.15711176187386	-5.99228815774492	-2.27415690067124
C	-1.21853950320898	-1.49316912875177	3.97793795045670
C	-1.44730120594901	-0.52593172332595	2.99026281405563
H	-1.63336823656966	-0.55806744549575	-1.07085888834157
O	-0.73896863529864	-1.14097874098365	5.19901524809503
H	-3.17913905075631	-0.04374766689271	5.40082039209696
C	-1.13028092858672	0.85429289471195	3.30100413598621
H	-1.06310432892688	1.57020207994400	2.48842234761431
H	-3.76492619444529	-1.75409509604065	-1.48902038977375
C	-5.15271474246254	-7.33434385720630	-4.48846716698323
C	-7.01948328821754	-6.40843797027122	-3.28323592851654
C	-0.89045481061520	1.20767537373575	4.56849405942763
C	-2.48298234290700	0.20397672417138	6.20819746293010
C	-1.04525914770305	0.20525806767135	5.68318798543012
H	-2.59346377372984	-0.52919521748947	7.01447079353660
H	-4.75750327814836	-7.86560046639812	-5.34971635198733
C	-6.51857229792262	-7.08195938207976	-4.39324309649553
H	-0.61448822207808	2.22399076795690	4.83711031047171
H	-8.08498387994715	-6.21584437678375	-3.19721991952571
H	-2.74243608682106	1.19614026899746	6.59186476896856
H	-7.19118498998673	-7.41435085330167	-5.17856958827230

C	-0.04381958624392	0.44787109131213	6.80279059242174
H	0.97718989274439	0.43309041884466	6.41114734138333
H	-0.13941239199298	-0.32651689403988	7.56948924019773
H	-0.23026519871991	1.42255673162374	7.26434002537075

Cartesian nuclear coordinates of $(S_0)_{(M)\text{-}(Z)}$-**2**-ax (**ω**B97X-D4/cc-pVDZ + C-PCM CH_2Cl_2)

C	-1.19091213741730	-1.03184157758949	3.53983697679105
C	-1.46297501779635	-0.11406340692908	2.51687362203611
C	-1.99360463054494	-0.59954160052409	1.31757173372601
C	-1.45838885328527	-2.39302815532324	3.36912613996630
C	-1.99198363989265	-2.84113243822545	2.16612762931924
C	-2.24824056527051	-1.96032343418352	1.10280582844768
C	-3.72151037204638	0.05283063043748	-0.06590428310549
C	-2.89546517748245	-2.35088924211853	-0.17342455979945
C	-3.76540524419726	-1.27584238891254	-0.81290308061668
C	-2.77111543667242	-3.54251677710982	-0.80277914550989
C	-1.18742526597572	1.30400821465902	2.76836370783476
C	-1.02786650148873	0.71959255650076	5.17167066108494
C	-0.94559328070554	1.70369921967254	4.02200448045918
C	-4.63071302725374	0.17949622762975	1.14671369176928
C	-4.72472577791704	1.43468856404122	1.76268712990744
C	-5.30374383775796	-0.90460780758747	1.71567597614583
C	-6.06031122770225	-0.73676478174921	2.87969045253048
C	-5.47528736715892	1.60578981301226	2.92405771114901
C	-6.14602650974117	0.51566122004888	3.48797324448674
C	-3.71109335968562	-3.86348168541780	-1.95846412376021
C	-1.80403833135322	-4.61426681691542	-0.46523383933654
C	-3.56813850301027	-5.29607765943125	-2.46260561642365
C	-2.17589340125646	-5.93638939719699	-0.73822452693326
C	-0.49691606503125	-4.39006750059053	-0.00453748010422
C	-1.31749017646689	-7.01839487698491	-0.52094133273091
C	-0.03214468143476	-6.75346260700361	-0.02996838026551
C	0.38176849749399	-5.44278526157896	0.22404754072557
C	-1.68288791771679	-8.41352920840008	-0.78504409530541
C	-0.71865085642015	-9.34063734589369	-0.81241381765679
C	0.73403356504037	-8.94065303953799	-0.64810829878873
C	1.34393414096223	-8.59075844829927	-2.00835512360384
C	1.53347772494787	-10.02019860024007	0.06882014287354
C	-0.01944669253083	1.05082214893411	6.26394287651188
C	-2.45780484336269	0.66350902648525	5.71624242332564
C	-2.44142470319893	-5.56141947440687	-3.44934737504114
C	-1.43548019415920	-4.63403699674474	-3.73190785107602
C	-2.37288224197236	-6.83451846269020	-4.03109651235633
C	-0.37743090258586	-4.97425244644904	-4.58082410612847
C	-0.31542307818574	-6.24490726600882	-5.15336163638615
C	-1.32049681832381	-7.17758233147523	-4.87728404246719
H	-1.26611658177569	-3.08028075377015	4.19519803225778
H	-2.23443037152276	-3.89868763763627	2.05451372460414
H	-4.81017807718295	-1.61075637457064	-0.91608804056598
H	-3.40238076641357	-1.09882660995552	-1.83771822134018
H	-0.73235592596450	2.74880359428816	4.26322889035995
H	-1.17891674969745	1.99802717209375	1.92641595256406
H	-3.94385844745517	0.87264599969342	-0.76187773602996
H	-5.53799219811614	2.59134832985827	3.39289916156634
H	-6.73462591027797	0.64459403983024	4.40001929970788
H	-5.23534938865117	-1.89614078891394	1.26332456315083
H	-6.58033200299931	-1.59420260009944	3.31506973606016
H	-4.19165378524953	2.28480770020205	1.32745355941626
H	-3.58124705874643	-3.15726976612924	-2.79450197437286
H	-4.74756685742701	-3.73260785017365	-1.60913406032211
H	-0.15723828072772	-3.36689192456871	0.16241629811974
H	1.39937896311133	-5.26624790974588	0.57767508971869
H	-2.73326871555348	-8.66461015365540	-0.94144767142884
H	-0.93780124920315	-10.39569713832016	-0.99870376613788
H	1.08679003417592	-10.23587735564394	1.05105776901234
H	2.57249995346538	-9.68853727875561	0.21480726132564
H	1.53796668619831	-10.94408173725246	-0.52894015700272
H	0.99936286773259	1.07188349362671	5.84857836598774
H	-0.06405253365609	0.29425222659231	7.06176289728961
H	-0.24504844033996	2.03652865908732	6.69796124087503

H	-3.16077388848817	0.36143462131700	4.92426374053984
H	-2.76014340650466	1.65705852096085	6.08252871116168
H	-2.52191582494216	-0.05655296725392	6.54752827628938
H	0.78104189834951	-7.77350118828924	-2.48577219311627
H	1.30901038798969	-9.46745121886906	-2.67385149342471
H	2.39346919987879	-8.27928863910561	-1.88561590697756
H	-4.51556176999028	-5.62400099227240	-2.91085540296301
H	-1.28080022248375	-8.17553057315306	-5.32169090761958
H	-3.15188742152693	-7.56774726964426	-3.80403794347744
H	-1.45881771105339	-3.63915239640642	-3.28218064597591
H	0.40596512061151	-4.24083280487306	-4.78924080758432
H	0.51352481074145	-6.51048755292158	-5.81468997964008
O	-2.35404495451635	0.31211979479958	0.35455734287653
O	-0.65465847861015	-0.61425237044591	4.72082793630680
O	-3.39618436491123	-6.18155092986701	-1.32094862284969
O	0.83794417029003	-7.77261595595982	0.21643493994378

Cartesian nuclear coordinates of $(S_0)_{R,R\text{-}2'\text{-eq}}$ (ωB97X-D4/cc-pVDZ + C-PCM CH_2Cl_2)

C	1.07285569115582	-0.26203557725787	0.21493777333888
C	-0.24928393369002	0.32978155164170	0.46322422320386
C	-1.35638322926352	-0.42553234216505	0.24032308649812
C	1.23003696667293	-1.48768743376838	-0.30676453553434
C	0.05546874336598	-2.31089863639450	-0.77037480161844
C	-1.27383347143279	-1.79891464541682	-0.24319054915135
H	2.24312027028291	-1.84918323897392	-0.48585368900668
H	-0.00004856207298	-2.24039052716947	-1.88109422186625
O	-2.56634081215086	0.09203758460662	0.59415590821554
C	-3.70531229662408	-0.48878629967873	-0.06237860005936
C	-2.37850804955010	-2.58854521546721	-0.24729517637298
C	-3.71204299997241	-1.99849215276346	0.13951531679290
H	-3.95384625307877	-2.23406482011122	1.19057028504575
H	-4.52203797133318	-2.41634621928181	-0.47586074615331
C	-2.26999076060944	-3.99186720647335	-0.64303483795013
O	2.17439711469273	0.51430097227656	0.45652636284059
C	-0.30006829155190	1.69008622500801	0.99221476062768
C	2.10712667353692	1.47267759356061	1.54568681341922
C	0.81048570756852	2.24854565539526	1.49000827166592
H	0.80751147454522	3.26056191315587	1.90441891404422
H	-1.25495281822076	2.21881144842803	0.97898567380227
C	-4.94003849131196	0.16797326878802	0.50716886814986
H	-3.63005814987061	-0.26966578074511	-1.14173898988288
C	-5.01058102521263	0.49330694007537	1.86641658969955
C	-6.04730000871978	0.40278177245006	-0.31281434958113
C	-7.21649677486641	0.95205337690995	0.21830329681326
C	-6.17604796084656	1.04786948495458	2.39640107824712
C	-7.28307147121131	1.27701591861124	1.57470006936273
H	-6.22077926441663	1.30208246451126	3.45881050408248
H	-8.19557835227619	1.71162413002375	1.99104814925996
H	-5.99348717095671	0.15594584425499	-1.37729927120988
H	-8.07643961967156	1.13225177041729	-0.43207134356887
H	-4.14390360721511	0.31811755686500	2.50755655132732
C	-3.50259054468165	-4.84159968472115	-0.83345543119503
C	-1.04573396212038	-4.56230423495172	-0.78125732653211
C	-3.17269369616714	-6.30298892489575	-0.55849170226755
H	-3.90210965305991	-4.73593810583225	-1.85698917421833
H	-4.30216218336710	-4.53794263328262	-0.14320421315823
O	-2.05095182981027	-6.70782629672160	-1.35993265283237
C	-0.92646072934291	-5.95269880144311	-1.20599892127552
C	0.22044399068838	-3.79950684865095	-0.43611018802474
C	0.26650405495144	-6.50094206761380	-1.55515346207912
C	1.46912546428022	-5.65560737107199	-1.52619152063596
C	1.45447142765769	-4.39616690134374	-1.06472857878770
H	0.32869806414040	-3.86760877490909	0.67131295702162
H	2.38986133867628	-3.83577632218690	-1.05458774399747
O	2.65483849833837	-6.21867373118840	-1.91440623230800
C	0.41318740640082	-7.88113468356721	-2.01050763932567
C	1.53008971743175	-8.24881901831292	-2.65142985563227
C	2.61533218452480	-7.23826647334528	-2.94818322020924
C	2.35469085046585	-6.55775327032474	-4.29486673795327
C	3.99628817261631	-7.88108434768360	-2.90117756058297

H	-0.40233254887279	-8.58205661286186	-1.82198439579553
H	1.67471903291259	-9.27078290348461	-3.01270193553671
H	4.16560316814911	-8.34665051298414	-1.91837172836551
H	4.77348656690468	-7.12159519507136	-3.07409178813840
H	4.07960675530981	-8.65564172930220	-3.67857760796868
C	3.30439861338816	2.38811252502711	1.31996059687258
C	2.21830442362274	0.71468003737675	2.87166231978511
H	3.21012209313299	2.90401152716437	0.35246446348344
H	4.23568982436712	1.80209166771999	1.32154222081033
H	3.35935517080223	3.14219167869395	2.11959784918001
H	1.37972097203790	0.00925012452375	2.98070552373009
H	2.18897944932444	1.41847807800149	3.71794239771426
H	3.16532584955272	0.15336643710684	2.90969681752638
H	1.38526534306457	-6.03586168530757	-4.27838807963323
H	2.32957523082144	-7.30518305981231	-5.10291818916281
H	3.14990290792957	-5.82687864298971	-4.51085837004329
C	-4.32586469397532	-7.22563303592882	-0.87272581862848
H	-2.89371707505860	-6.41139475673886	0.50449899302356
C	-5.27960800863321	-7.49313310061302	0.11472218180683
C	-4.48457240655075	-7.77676912804328	-2.14815725290880
C	-6.38264300116032	-8.30053114210350	-0.16788245414323
C	-6.53587628013736	-8.85385101229239	-1.44143421801216
C	-5.58384630622559	-8.59057586369860	-2.42918595749818
H	-5.69733016316412	-9.02218318507600	-3.42722588767090
H	-3.73865278378495	-7.57312915696883	-2.91889373826026
H	-5.15510102602638	-7.06851690241918	1.11542421945583
H	-7.12118642107373	-8.50461935879841	0.61180697366980
H	-7.39489455397450	-9.49258197533966	-1.66291931577967

Cartesian nuclear coordinates of $(S_0)_{S,S\text{-}\mathbf{2'}\text{-eq}}$ ($\boldsymbol{\omega}$B97X-D4/cc-pVDZ + C-PCM CH_2Cl_2)

C	0.78810869437845	-0.10318333946783	-0.44105128136067
C	-0.39252239797769	0.33312009833578	0.32324507192712
C	-1.43093641809306	-0.52946701742607	0.46307340870487
C	0.93511091975398	-1.35388486304864	-0.90022157307620
C	-0.07556417887712	-2.42919333590518	-0.59901160578620
C	-1.39606978402744	-1.87726266253066	-0.09157908997771
H	1.84124753440861	-1.60263006179244	-1.45368831847528
H	0.33461069889213	-3.06184563281692	0.22223040530030
O	-2.54174222927707	-0.12540191166095	1.14873134505127
C	-3.78053456694085	-0.63636659415734	0.61949634054063
C	-2.49421671032168	-2.67141720212880	-0.03153288766970
C	-3.75977222443702	-2.16099806796012	0.60809904469463
H	-3.84184852604281	-2.54010029706395	1.64174446139044
H	-4.64916772026039	-2.51766067086826	0.06656842398019
C	-2.41790885697106	-4.04130959194938	-0.53137202396629
O	1.71196607243761	0.85504573112873	-0.76888618283968
C	-0.40060881346261	1.69727195219193	0.84368291566811
C	1.99949124488803	1.87339675052502	0.22468043330692
C	0.71725143393345	2.43366293330237	0.79783616653751
H	0.74766716577529	3.45136688599288	1.19709021373186
H	-1.32730341863806	2.08207388631976	1.27301396423941
C	-4.90266639132773	-0.08885013150327	1.46724185583099
H	-3.88537073814106	-0.26844692302122	-0.41600277196051
C	-4.75836635084055	0.02036378431385	2.85483234061049
C	-6.11960964030460	0.25786273716981	0.87401579752934
C	-7.18711865730125	0.70092011718398	1.65840152075221
C	-5.82160482222791	0.47044402498801	3.63833964622828
C	-7.04005821547210	0.80836191007949	3.04273326951059
H	-5.69932424148674	0.55670720360195	4.72132273861507
H	-7.87323525602397	1.15902717053864	3.65731656839961
H	-6.23202022380691	0.18203557118849	-0.21147479720518
H	-8.13489216147743	0.97025619597137	1.18500231931362
H	-3.80512028301769	-0.24232783593806	3.31864633024256
C	-3.50763207645716	-5.03244177209892	-0.21153659971791
C	-1.38359208616324	-4.40438484803648	-1.33058825209410
C	-3.00631106817458	-6.45938145131579	-0.40444757405997
H	-4.38297216614702	-4.85862259322317	-0.86132495619276
H	-3.85296041511966	-4.92017307363633	0.82749947348569
O	-2.37920991610508	-6.59374235682846	-1.69321415463973
C	-1.32100526953015	-5.75071243897331	-1.88654153991853

C	-0.35455934254075	-3.37782252937198	-1.77092662039785
C	-0.24684963090464	-6.18956238822985	-2.59028368887069
C	0.90053212128047	-5.28271910817857	-2.76161353437888
C	0.88012270182969	-4.00222321234265	-2.36657171103714
H	-0.84223467370927	-2.76170595722075	-2.56167688228933
H	1.76755388079248	-3.39138747996585	-2.53595874043270
O	2.05959812306837	-5.82325191836996	-3.25540189612729
C	-0.13801480611234	-7.53648666052658	-3.14338479097045
C	0.88899575425034	-7.84593451887243	-3.94544456028587
C	1.94035663025778	-6.81971505212960	-4.30353834921941
C	1.57253509545147	-6.11318663385261	-5.61150050725149
C	3.32292993971110	-7.45739342369464	-4.37916196915921
H	-0.91441515231930	-8.26239212851244	-2.89581455885182
H	0.98539697288439	-8.83983339196982	-4.39142614882936
H	3.56849347000316	-7.94523954631217	-3.42373316032990
H	4.08139252703856	-6.68891553889035	-4.59144485717547
H	3.34946592029167	-8.21179559550716	-5.18000396501471
C	2.76832635009595	2.94304075429414	-0.54237286163647
C	2.85364704146629	1.24982676682564	1.33249609144486
H	2.13869444835850	3.36281230592609	-1.34145400379244
H	3.67226332586218	2.50644787919841	-0.99319754000906
H	3.06831119391228	3.75540731900718	0.13655835288151
H	2.29910992138364	0.44379459100347	1.83775277270511
H	3.11899995830773	2.00871623890007	2.08466386930497
H	3.77943003754263	0.83263716413112	0.90541489685582
H	0.60309991839645	-5.60085338283937	-5.51053387376992
H	1.49309966973175	-6.84236116915395	-6.43248373258512
H	2.34366741683979	-5.37037528368209	-5.86971339522903
C	-4.12260439751901	-7.47198167199731	-0.32232319788255
H	-2.24856920895312	-6.68272769545584	0.36685341871109
C	-4.46184392892475	-8.02479301886102	0.91634900188948
C	-4.86147100669344	-7.82264215502099	-1.45696248509897
C	-5.52981542163128	-8.91748810572283	1.02337948716792
C	-6.26383306325760	-9.26865466468431	-0.11192899505858
C	-5.92505555202609	-8.72064939120692	-1.35161413270475
H	-6.49427564334073	-8.99320497663822	-2.24433530071828
H	-4.59516117747016	-7.39583064746695	-2.42617100783440
H	-3.88215609388393	-7.75639444169288	1.80457972981501
H	-5.78517400872315	-9.34500597115984	1.99649903334773
H	-7.09696725076288	-9.97167763923641	-0.03141063383359

Cartesian nuclear coordinates of $(S_0)_{TS}$ (**ω**B97X-D4/cc-pVDZ + C-PCM CH_2Cl_2)

C	-1.92818454410223	3.45697643432912	-1.81423930940675
C	-1.07970721208388	3.91317015911349	-0.79648151440119
C	-0.48620929630492	2.98486884434904	0.04391077747074
C	-2.18805785957483	2.09902292145702	-1.96973893885394
C	-1.59186041405257	1.18902338119877	-1.12543021131631
C	-0.71529321048101	1.59351437446530	-0.09230393920285
H	-2.85370807462385	1.78406300975245	-2.76291285661446
H	-1.76796282888177	0.12952527960196	-1.26245555613579
O	0.34874535763279	3.46421422891774	0.99662376184196
C	0.49021393262344	2.66217733454906	2.15881841315638
C	-0.03564724367215	0.68413547028513	0.75028732326868
C	0.90880693841187	1.25213244082025	1.77004030145224
H	1.93928544728792	1.27778027553622	1.38860955837446
H	0.93060204194039	0.62556664625884	2.66651008760996
C	-0.19858766044911	-0.76512693581408	0.64076285276705
O	-2.56350583159743	4.32920004252851	-2.62270815683412
C	-0.90424928687376	5.34840935793340	-0.65026792933296
C	-1.95706847337322	5.61020297664982	-2.86840163712503
C	-1.34346697112224	6.16030192622478	-1.60745722380571
H	-1.23884464551888	7.23719261285994	-1.53096226068040
H	-0.42704968007952	5.72131339924618	0.24652603995475
C	1.47575541160457	3.33161105126395	3.07152829211522
H	-0.48655070962205	2.61135629846947	2.65593613739470
C	2.49556541763215	4.13142951822456	2.56826652723743
C	1.39786784137765	3.11473155501373	4.44263518202415
C	2.32863927283216	3.68009405467397	5.30089970995739
C	3.42369455029349	4.70214314395795	3.42706963214832
C	3.34518257761669	4.47741057104843	4.79411122191352

H	4.21238646930665	5.32848617023184	3.02560561923617
H	4.07056789611854	4.92593363155151	5.46315234571523
H	0.59702738584316	2.50192274130470	4.84400830763437
H	2.25483197541145	3.50490393239895	6.36815609891484
H	2.54932638284358	4.31224172859634	1.50260921188500
C	-1.31081164718122	-1.45019802563059	1.36301494221261
C	0.66395773355893	-1.56187406490026	-0.14112008920622
C	-0.91296185951600	-2.87593063436761	1.71558858446487
H	-2.23233813427814	-1.48563931501199	0.76083846974323
H	-1.57242926126459	-0.91471887731961	2.28093813720434
O	-0.48798237652571	-3.56207911481834	0.54395931623433
C	0.47458358252991	-2.96133466338206	-0.18746020655493
C	1.69988525915521	-1.03609946013187	-0.93887069202002
C	1.25257762152153	-3.78230139935255	-0.98611426358673
C	2.25015824730683	-3.20534768648939	-1.77172627834639
C	2.48037954979976	-1.83410512952134	-1.73818239735258
H	1.85692362253810	0.03522046945283	-0.93541072587964
H	3.26930101658402	-1.42344007064315	-2.35444759346693
O	3.05577785783615	-3.96324766570757	-2.53121467065970
C	1.13122069625183	-5.23402249734815	-0.99815686855846
C	1.79051272306228	-5.93729591362468	-1.91061671344900
C	2.59876552961676	-5.25373229377623	-2.98115667554588
C	1.75673707609099	-5.04153704658040	-4.23325577038527
C	3.87008865515516	-6.02042329218032	-3.28955889548183
H	0.51767959615233	-5.70502298621959	-0.24154110348409
H	1.73999011014777	-7.02024302035195	-1.93911458610939
H	4.46642752120590	-6.14298315085118	-2.38306268675236
H	4.46514061935358	-5.47844770299145	-4.02781417386927
H	3.63064645019754	-7.00882985524492	-3.68995922944586
C	-3.09718782745428	6.48431262941622	-3.35294874667930
C	-0.89204405600955	5.43489000982859	-3.94499021311048
H	-3.85399584420388	6.58335547353257	-2.57200348538571
H	-3.56223240123621	6.03845991804017	-4.23502757129467
H	-2.72967667535121	7.47954651558315	-3.61610898419157
H	-0.11812408975555	4.74322608271855	-3.60371450096212
H	-0.41645902585284	6.39300564502273	-4.17193310403812
H	-1.34027161581832	5.03756237785978	-4.85985292278663
H	0.88520054075659	-4.42384133651991	-4.00356905898269
H	1.40232557679317	-6.00061329789675	-4.62083914726893
H	2.34725575379728	-4.54393879252801	-5.00743102562411
C	-2.03317044895031	-3.65916918986134	2.33275854133357
H	-0.06578288519401	-2.84110297276980	2.41099459189011
C	-2.22131408852951	-3.62040694130964	3.70946639680281
C	-2.92260330956892	-4.38279580207110	1.54616736627388
C	-3.28771624217736	-4.28595903463381	4.29493562556651
C	-4.17347938718047	-5.00588986107499	3.50594995222592
C	-3.98592347104333	-5.05377526910976	2.13167358281783
H	-4.67166983720630	-5.61907768762146	1.51067427135396
H	-2.76893538385334	-4.42567397032060	0.47556025110516
H	-1.52406088175761	-3.06738655443491	4.33065441672263
H	-3.42214728136260	-4.24925581948125	5.36996909470975
H	-5.00476226450337	-5.53176730237446	3.96135097145435

Cartesian nuclear coordinates of $(S_0)_{TS\text{-}CC}$ (ωB97X-D4/cc-pVDZ + C-PCM CH_2Cl_2)

C	-2.45901195228732	3.16796103647584	1.99011692143888
C	-1.45159019829394	3.75992786582905	1.21573786250275
C	-0.62024155874234	2.94340578093333	0.46649747660818
C	-2.60510933717242	1.78504509525733	2.03313004655948
C	-1.76980550713705	0.98674196942978	1.28368156405836
C	-0.75955627400413	1.53345877256966	0.45892534830495
H	-3.37927149765440	1.36293272446056	2.66089871038791
H	-1.88188665295427	-0.08995215168328	1.30832532460964
O	0.33293951003015	3.55127219878106	-0.27966740502863
C	1.49607581581596	2.77976903702165	-0.54036525613762
C	0.07429576328811	0.75031024187361	-0.37149222596134
C	1.10770355453676	1.46288022473019	-1.19534745366347
H	0.73365023370385	1.67109113132980	-2.20784720976889
H	1.99932380453927	0.84042560231851	-1.31388261793413
C	-0.07922178084988	-0.69801209964050	-0.49851098557623
O	-3.25878102956962	3.92194921123756	2.77030309604892

C	-1.30457773522419	5.20433188745509	1.28161305598928
C	-3.50927400886785	5.28650567349350	2.39124306820236
C	-2.25485883936892	5.93119364672033	1.86226755456789
H	-2.17664077498957	7.01027579558409	1.93898371247667
H	-0.41262732026271	5.65155644363406	0.86276285447166
C	2.42038560421639	3.60319882925067	-1.38802298718487
H	1.98153409190734	2.56406465567953	0.41973681296880
C	1.92660269300563	4.55524476366872	-2.27270517200003
C	3.79164153775950	3.38316593774252	-1.32309022000778
C	4.65950899648302	4.09843090407659	-2.13423155273359
C	2.79494701531297	5.27453565730713	-3.08070250124443
C	4.16248783681120	5.04758710400829	-3.01617717622562
H	2.40056545044755	6.01895919226074	-3.76320839228316
H	4.83948862299262	5.61207212555717	-3.64708449153038
H	4.18501605676399	2.64923764550692	-0.62705948745249
H	5.72684171580630	3.91963962376293	-2.07157578508459
H	0.86047433740438	4.73647105852293	-2.31359272842184
C	-0.88722691978787	-1.26584402306243	-1.61727965703417
C	0.61326405974944	-1.59686080395792	0.33920629746778
C	-1.36931246585773	-2.66572116619476	-1.26869149499477
H	-0.30762286184340	-1.32251195038865	-2.55252164861837
H	-1.75317945249738	-0.63218261227443	-1.83366148810162
O	-0.26637408799156	-3.47668186885336	-0.87787732104382
C	0.52141078430449	-2.98685982080818	0.10199406823478
C	1.44084072303779	-1.18838092450877	1.40425759582117
C	1.23885693915779	-3.90761479327385	0.84693866988435
C	2.07446567981829	-3.44417204335967	1.86345715154427
C	2.16122109670001	-2.08676973606413	2.15235979279819
H	1.50982868008479	-0.13009688667336	1.62236065901651
H	2.79989467310867	-1.76772729347896	2.96556248456776
O	2.76250176320428	-4.29982599526414	2.63525410539442
C	1.11818262670845	-5.34873304137473	0.67000382447262
C	1.96766909137993	-6.15619887406695	1.29332324215513
C	3.10006569701101	-5.60125199823927	2.11629867428623
C	4.35666989370043	-5.45607526217892	1.26636850895874
C	3.35696117194378	-6.44663361082839	3.34829006476453
H	0.31807719474477	-5.72503140900421	0.04622498160138
H	1.89859677647393	-7.23457938439145	1.20149865307380
H	2.44905534857686	-6.52319643490899	3.95010644561881
H	4.14190565741424	-5.99314533999410	3.95736474413309
H	3.67432890464870	-7.45206096303870	3.06011592666119
C	-3.97739345034099	5.95779866308564	3.66793940637130
C	-4.60027802883736	5.29221728811925	1.32662978713747
H	-3.18678058304333	5.92877140853729	4.42066279782261
H	-4.85466751576886	5.44135966539191	4.06398369278939
H	-4.24267573079042	7.00053336457682	3.47503266254465
H	-4.27140430931699	4.73942704444218	0.44340817205056
H	-4.83090233856786	6.31652234252333	1.02110973026611
H	-5.51024062327309	4.82636545729526	1.71509009554346
H	4.17078239659716	-4.78222100232984	0.42659797160247
H	4.65783132222429	-6.42697272853710	0.86363733708963
H	5.17650905070850	-5.05312299202482	1.86731626953109
C	-2.06034858199477	-3.34550264632937	-2.41346128127880
H	-2.05560532997768	-2.59906466577686	-0.41600471679566
C	-3.42895604648093	-3.17798838712217	-2.58866446868330
C	-1.34451796736258	-4.09917922859479	-3.33735728631431
C	-4.07564834259410	-3.74787945350590	-3.67520258567004
C	-3.35741092594319	-4.49858681211352	-4.59487213757928
C	-1.99167328919910	-4.67402810560778	-4.42090890179228
H	-1.42607540807685	-5.26401723918735	-5.13334883268795
H	-0.28107237450216	-4.24204419143093	-3.19460218294576
H	-3.99526383277579	-2.59915465228723	-1.86615263907677
H	-5.14382552142132	-3.61137101351961	-3.79940082576707
H	-3.86149171649863	-4.94938346457146	-5.44204610577620

Cartesian nuclear coordinates of $(S_0)_{(P)\text{-TS}}$ (ωB97X-D4/cc-pVDZ + C-PCM CH_2Cl_2)

C	2.68780586262412	2.48465434157102	0.81016718365685
C	1.49601856944968	3.21436134158215	1.04702820450069
C	0.28489590764420	2.64332783400516	0.61975148311444
C	2.67772679852509	1.28991331674356	0.15559794714838

C	1.44416194072450	0.65227441773638	-0.23713660070240
C	0.21764569658705	1.41065367680160	0.02422730495369
H	3.62399768859383	0.81637768007198	-0.07228140362072
H	1.48177254548549	0.20320054430463	-1.22901822073569
O	-0.84505143368951	3.36394464030597	0.84106386500609
C	-1.85264288517072	3.16449893063201	-0.14066039302794
C	-1.07503369500853	0.78972024075320	-0.17527704191909
C	-2.27054993732513	1.70036221491589	-0.18291331218762
H	-2.90396786453314	1.48872437453704	0.68635209874920
H	-2.89661591112337	1.54019651682139	-1.06697716155519
C	-1.19251437754603	-0.55852577884638	-0.25902564470590
O	3.87901337467046	3.04074989105041	1.12612058643664
C	1.59274517116938	4.50958380125609	1.65683782631319
C	3.94254050516832	3.98420925499950	2.20759028213599
C	2.75358425052191	4.90497103670730	2.18669782330987
H	2.86251345582027	5.87672555597995	2.65484265322278
H	0.71271169558454	5.13963156828998	1.67267077229388
C	-3.00684939438615	4.07072134099392	0.17485728074828
H	-1.42563467213380	3.43844317340661	-1.11302162684507
C	-3.28204522076332	4.45090920611428	1.48373262349337
C	-3.84907125824395	4.49535755349831	-0.84627793681728
C	-4.95680239527181	5.28136414397886	-0.56606433346075
C	-4.38666137469468	5.24190288699656	1.76362787851865
C	-5.22894765124363	5.65686928689353	0.74182788546122
H	-4.58989241022304	5.53685769703886	2.78701726237817
H	-6.09131440106491	6.27517690950197	0.96305049781052
H	-3.63462441103196	4.21280675141575	-1.87197191803202
H	-5.60454824881465	5.60713057286532	-1.37189464968734
H	-2.62008485928023	4.13073747641846	2.27773954556791
C	-2.46976652111011	-1.22860753799135	-0.68129284998042
C	-0.05067418179701	-1.41758459854260	-0.02727264939437
C	-2.35028451537136	-2.74503422073148	-0.60640239428813
H	-2.71689274511168	-0.94404444370667	-1.71077987770033
H	-3.31697139446762	-0.91375142684196	-0.06309196934775
O	-1.12978210468316	-3.17576074090982	-1.19403929219313
C	-0.02398849184516	-2.66723802108164	-0.59009608063189
C	1.13419302852677	-0.89233770810944	0.65722838335816
C	1.12726282770783	-3.47348284261019	-0.59030108440134
C	2.28462591717488	-2.96894675285078	0.05459671846149
C	2.28303270755135	-1.76483590376832	0.69128641347225
H	0.91616342663003	-0.42229519047173	1.61568813702356
H	3.16825627857797	-1.46724467547495	1.23846393167949
O	3.38170882757871	-3.75320599098636	0.15282754035859
C	1.17575763699955	-4.79070173806043	-1.15717252344942
C	2.35053838589326	-5.41651549299128	-1.27226324764876
C	3.62764144834137	-4.72926094194144	-0.87247562436877
C	4.24702167847824	-4.01767182208967	-2.07078041794482
C	4.60334227153357	-5.70469225604600	-0.24257125093426
H	0.24967845844712	-5.24702436182300	-1.48239171424867
H	2.42504779696731	-6.41174332610528	-1.69569857269037
H	4.14678687053098	-6.18409806858544	0.62585264588837
H	5.50326162305479	-5.17819527442364	0.08261576450271
H	4.88944525493585	-6.47637629752137	-0.96192446065966
C	5.23132958508563	4.74750326486635	1.96966911393910
C	3.99084201413351	3.20593419246853	3.51853453696729
H	5.18162226894001	5.28140740792583	1.01840197016013
H	6.07626591580027	4.05606366909826	1.93756304945946
H	5.40030952970158	5.47066348938798	2.77173996979775
H	3.08348416057053	2.60893578355163	3.63886080800492
H	4.06390711374867	3.88926859892888	4.36888569002666
H	4.85551186184988	2.53640818330820	3.52779837407644
H	3.55496999449168	-3.27007399365105	-2.46587084871137
H	4.47019884984045	-4.73056158779549	-2.86917504068148
H	5.17321732433436	-3.51737537843066	-1.77458806611880
C	-3.48318513613640	-3.43486216999287	-1.30812854430795
H	-2.33364129884898	-3.04590385549026	0.44809371537710
C	-4.66341921296805	-3.69562390134219	-0.62229777849934
C	-3.39244883985811	-3.76878906508322	-2.65509708387259
C	-5.74388032738506	-4.27353039371692	-1.27232540836834
C	-5.64987846529340	-4.60293866458434	-2.61679574639785

C	-4.47042541239323	-4.35207415981474	-3.30401674127085
H	-4.38844901076766	-4.61304235983726	-4.35318008366541
H	-2.46876424497499	-3.57832970980846	-3.18637692816266
H	-4.73741910565402	-3.44875822153853	0.43185013830521
H	-6.65843755270660	-4.47319894115714	-0.72563034200507
H	-6.49139555707389	-5.05830495296894	-3.12620104043765

Cartesian nuclear coordinates of $(S_0)_{(M)\text{-TS}}$ (ωB97X-D4/cc-pVDZ + C-PCM CH_2Cl_2)

C	1.34077326959046	1.70787273943763	3.07407367997215
C	0.72302013392500	2.76387522037173	2.36385653125525
C	-0.05917418493408	2.43351088821166	1.23270359495887
C	1.16667238414635	0.40587219799726	2.71764006012526
C	0.40959483694731	0.03929471190557	1.54202512957511
C	-0.22592898917506	1.14443167668396	0.81278771537954
H	1.62316999350157	-0.36489075087659	3.32512705797598
H	-0.26716494476873	-0.79582942581832	1.72477573482782
O	-0.61578527561350	3.49453442438123	0.59399222699111
C	-1.05055021562430	3.30320583263921	-0.74226974998276
C	-0.90951211915423	0.87411696347562	-0.44579606990040
C	-1.80949222759376	1.98493300171306	-0.88505801820492
H	-2.70563267791797	2.03885200253342	-0.25662602079130
H	-2.13288214055851	1.88117426195329	-1.92031069857674
C	-0.63731240756200	-0.24513186821032	-1.14845446680169
O	2.15825707704562	1.99135589460249	4.11196731599979
C	0.97472741289588	4.11101728828247	2.78419158601352
C	1.93877022993397	3.19519604368452	4.86523448057702
C	1.58470555817930	4.33628625888571	3.95263345365605
H	1.79557195633214	5.34218015597881	4.29761468308808
H	0.66112850433251	4.92148298285835	2.13921158091403
C	-1.88986892729386	4.48529687419603	-1.13238292470910
H	-0.16245618595330	3.26005842692702	-1.38447368723192
C	-2.67784489155672	5.14645737303012	-0.19687466265188
C	-1.92930971079299	4.89199395157757	-2.46114509022045
C	-2.75134689884428	5.93707042026698	-2.85528329791582
C	-3.49683307993107	6.19483874265714	-0.58956280794123
C	-3.53841499945641	6.59194229851630	-1.91864760735398
H	-4.10499258970295	6.70640432839194	0.14800331725533
H	-4.17813507438024	7.41241073657504	-2.22293203147569
H	-1.30770881897438	4.38875798733010	-3.19492770188162
H	-2.77127058785414	6.24554476281325	-3.89430486809942
H	-2.63585531302554	4.84146342111762	0.84076334082204
C	-1.35923629357409	-0.70674805155518	-2.37470437481572
C	0.36490925945968	-1.19158417462229	-0.67600404770047
C	-1.78447623051721	-2.15908357875430	-2.16364109089977
H	-0.70633820882798	-0.66774813072091	-3.25395942654139
H	-2.24694064617987	-0.11285145324407	-2.59016715309053
O	-0.66176001419466	-2.97995009658547	-1.88767077077378
C	0.26559371762224	-2.51087863536402	-1.01435910513131
C	1.38324231644666	-0.74724383159704	0.28380006897005
C	1.11775133779580	-3.49728449543231	-0.46560473881863
C	2.10574348645120	-3.08110106312660	0.45728810366567
C	2.26901640681781	-1.77134046560777	0.79033216118266
H	1.91846887122767	0.14386361765457	-0.04503172181788
H	3.07189917502608	-1.50257641525499	1.46453907230906
O	2.88774148400401	-4.00537819974528	1.05800757077517
C	0.98832153313008	-4.89704800410317	-0.74606427041011
C	1.93175593496290	-5.74565921563538	-0.32480810537154
C	3.16396630780905	-5.23712826087057	0.37064534854635
C	4.27854283340507	-4.98125204148548	-0.63847531116082
C	3.61403643782820	-6.18769213366684	1.46309051242480
H	0.12147623489557	-5.23620161713473	-1.29814867643892
H	1.86228530108509	-6.81093532342545	-0.51372547879216
H	2.80863425458789	-6.33880361805639	2.18476116796909
H	4.47863403741770	-5.77405260853185	1.98686111474684
H	3.89192170760798	-7.15520485910619	1.03709845262275
C	3.25563552652890	3.44254261468149	5.57566304482001
C	0.81077471028771	2.93647130973395	5.85861938431341
H	4.05194027056898	3.60375676800116	4.84613622009132
H	3.51687938052073	2.57893186931644	6.19128690526602
H	3.18317616626108	4.32320460456242	6.21898440708914

H	-0.11308989139962	2.69321426337518	5.32840802179343
H	0.62855456084714	3.82256879051482	6.47269955295682
H	1.07066756374026	2.09989218572160	6.51331904277242
H	3.95885195093875	-4.24239543032103	-1.37727853323979
H	4.53654384515751	-5.90270575977226	-1.16746453816190
H	5.16968778556421	-4.60490863777206	-0.12840195722531
C	-2.49014790772355	-2.72776105727956	-3.36104221892887
H	-2.45448861691995	-2.19849006157320	-1.29610156363567
C	-3.86351480226044	-2.55766688869821	-3.49477200584092
C	-1.78616045314169	-3.37800724273357	-4.36836467932127
C	-4.52592285301967	-3.02131293579098	-4.62145216340529
C	-3.81876617931596	-3.66752204957357	-5.62541182155294
C	-2.44888690422439	-3.84609232984407	-5.49359067189059
H	-1.89166798936782	-4.35588777217845	-6.27154813142656
H	-0.71970305819979	-3.52613323865770	-4.25827785507032
H	-4.42217256044161	-2.06268091015622	-2.70675501035902
H	-5.59753686725985	-2.88487712937158	-4.71157737279004
H	-4.33476201758990	-4.03583813030276	-6.50468914335147

Cartesian nuclear coordinates of $(S_1)_{(E)}$-**2** (**ω**B97X-D4/cc-pVDZ + C-PCM CH_2Cl_2)

C	-5.29669088218440	-0.53862425496796	2.66022426672981
C	-4.13034913060569	-0.34467675437866	3.41495815736428
C	-3.19182934824485	-1.35822768446656	3.46478158731958
C	-5.52349649725496	-1.73849163578227	1.99345177938795
C	-4.58792962469457	-2.74292003977921	2.05176814368126
C	-3.36975003945586	-2.59445337359853	2.77559371997650
H	-6.43977578973886	-1.85151025164906	1.42856136182416
H	-4.74876986208151	-3.65038453305768	1.48978118121039
O	-2.09133709724895	-1.11510189988018	4.19918628434832
C	-1.31924668255446	-2.23477706346633	4.61541716471745
C	-2.34894764477329	-3.55739335271491	2.79284432370285
C	-1.05063213923062	-3.13652804199579	3.42208180165421
H	-0.41351736826453	-2.57337851091007	2.72388103117088
H	-0.47192285351444	-4.00349826906738	3.74373853555254
C	-2.52752180079241	-4.89148162625007	2.32528767850347
O	-6.25080498861002	0.40372639372993	2.61858763997710
C	-3.99954266048343	0.89971215883966	4.15520814771153
C	-5.88308623237830	1.78169814581775	2.82813922529711
C	-4.84479683806155	1.89659586404045	3.91201422462013
H	-4.79010170107767	2.83439413967689	4.45375687540569
H	-3.22161152721163	0.97566942105012	4.90340391463828
C	-0.06009567979384	-1.71222822360380	5.24234345763116
H	-1.89781081734766	-2.79047412782087	5.36301314966687
C	0.55953680081952	-2.43802282146982	6.25303877500021
C	0.53290073439402	-0.53911049898713	4.78776187022661
C	1.72911230744802	-0.10249552402010	5.33709683440135
C	1.75847783420810	-2.00471742344452	6.79900988754384
C	2.34677017542602	-0.83392716400034	6.34181860815846
H	2.23047437344436	-2.57882968302506	7.58812754042635
H	3.28126290123059	-0.49055075919606	6.77050027802507
H	0.04448693937516	0.03520086929454	4.01108191271739
H	2.18050447538630	0.81612188153376	4.97946117075007
H	0.09841289625966	-3.34978750849996	6.61899142401422
C	-3.73141081175326	-5.68838589730128	2.74226313885083
C	-1.58413839478761	-5.56207557515189	1.53198353849614
C	-3.33594580955913	-7.13042928028860	3.01100593769648
H	-4.50986548781545	-5.70145940709763	1.96479801953542
H	-4.19032337971575	-5.26367470450491	3.63630871049097
O	-2.71492156989738	-7.67594674677802	1.85298569110593
C	-1.72998017987887	-6.95912567464060	1.28180519203315
C	-0.48805543865361	-4.91651130537502	0.89012383913722
C	-0.87774236788564	-7.64986062711243	0.44047479473172
C	0.16760693792071	-6.95329351053523	-0.18438174484643
C	0.36204577461728	-5.59588951193641	0.05153557854089
H	-0.36083267904739	-3.85347181347241	1.02809652611467
H	1.18456736169102	-5.09721534722121	-0.44471565956026
O	1.04537218910976	-7.59393480778230	-0.97146233265703
C	-0.97223624465229	-9.08196289849261	0.20936185024581
C	-0.23648725240538	-9.64025716376246	-0.74701608216973
C	0.63550528983482	-8.80174457712925	-1.64282080565131

C	-0.12192252098023	-8.40078125450647	-2.90345851183244
C	1.93348202746077	-9.51081944716942	-1.97601541894197
H	-1.63342658035119	-9.66353778007764	0.83806300562662
H	-0.26922959784221	-10.70693788943435	-0.93990861378919
H	2.46760063854415	-9.77151458047377	-1.06002043182521
H	2.57167796130271	-8.86176514901053	-2.57969272716796
H	1.73268557990277	-10.42579813826474	-2.53920961336688
C	-7.17718506784008	2.46512622911576	3.22355607451050
C	-5.34257499646210	2.32651782082878	1.51121266261923
H	-7.55490639307657	2.04654875203904	4.15878521807681
H	-7.93102192863843	2.32036300854163	2.44652853855381
H	-7.01446155631873	3.53757821381399	3.35788106366442
H	-4.45551533076785	1.76640422928318	1.20615864530377
H	-5.06132178034624	3.37745334667788	1.62058465456711
H	-6.09936564377606	2.24313775635116	0.72616726755101
H	-1.01126700677455	-7.82237857073108	-2.64210729751245
H	-0.44260437978248	-9.28906728894355	-3.45443413058709
H	0.51505447997279	-7.79327550085435	-3.55237535131023
C	-4.51117846057361	-7.99962975278798	3.34863311989098
H	-2.61068213548328	-7.15738565907847	3.83286015903965
C	-5.27342546734985	-8.59025119611427	2.34588115427492
C	-4.88528849673476	-8.17220383198465	4.67589010390272
C	-6.39408987663313	-9.33971062787413	2.66980931986724
C	-6.76705202051175	-9.50361908092759	3.99679580725305
C	-6.00926375778889	-8.91736549031238	5.00040821857436
H	-6.28943537366774	-9.04625797222803	6.03961533487994
H	-4.29023819773116	-7.72173229184265	5.46370526304469
H	-4.97481628501584	-8.47000272902218	1.31226131842774
H	-6.97876076140821	-9.80043239440900	1.88154245941315
H	-7.64300724083778	-10.09046172997176	4.24828456184168

Cartesian nuclear coordinates of $(S_1)_{(E)}$-**2**-CC (***ω***B97X-D4/cc-pVDZ + C-PCM CH_2Cl_2)

C	-5.37759338150374	-0.09019495531214	0.45434220996164
C	-4.18313700506921	0.42398427557903	-0.07079091286173
C	-3.19925243407004	-0.45779579125326	-0.47690345575104
C	-5.55620819727048	-1.45899054498983	0.60315817952739
C	-4.56785019597458	-2.32865442243321	0.19574681407653
C	-3.35988286837310	-1.86693566243824	-0.38821875585145
H	-6.47726924541747	-1.81666348290017	1.04523065361709
H	-4.69687604355740	-3.39263277425398	0.34359396919889
O	-2.06618451688068	0.07660703472626	-0.97905640560315
C	-0.89081191099065	-0.70895876497899	-0.82334386944201
C	-2.35406969842819	-2.71744562251518	-0.88830413234387
C	-1.11022928618933	-2.08297343005875	-1.43083970630293
H	-1.16061959080301	-1.97979230370627	-2.52574686715744
H	-0.23278793897559	-2.71116787479149	-1.23464870530319
C	-2.54138732991103	-4.12392357304350	-1.02458165156754
O	-6.34236491963789	0.73375601780058	0.89963500254685
C	-4.02187658443126	1.86905501599889	-0.10168441839196
C	-6.44123868536382	2.05933922652447	0.34640751441267
C	-5.07343264772826	2.64911956861699	0.12668260684061
H	-4.99054657256532	3.73048095014166	0.12405229038979
H	-3.03758144264304	2.27362574768393	-0.29763879968193
C	0.24905052466456	0.03083719484578	-1.45736797035649
H	-0.70098613174656	-0.82174920442172	0.25114946276489
C	0.04560331225940	0.84400190856187	-2.56704580638836
C	1.53825847542980	-0.13469005006213	-0.96453590900546
C	2.61260707672442	0.49538540695379	-1.57457614827454
C	1.11881635262117	1.47884988271973	-3.17393407480206
C	2.40505968335006	1.30499801329437	-2.68214752032978
H	0.94928332820273	2.11522151612983	-4.03520776081478
H	3.24238583499926	1.80328275526214	-3.15718235302627
H	1.70340260423204	-0.75920207430176	-0.09255413283741
H	3.61272913259740	0.35995750466630	-1.17893962904234
H	-0.95981248477809	0.98495309472920	-2.94241729249721
C	-3.64601713607861	-4.63625837857381	-1.89527934980430
C	-1.65503656674114	-5.06961425816520	-0.48872077265031
C	-3.98092015865714	-6.07983883699803	-1.57292157789157
H	-3.35780963421671	-4.56132121846873	-2.95444472781268
H	-4.54167472974629	-4.01552601635074	-1.78646643308038

O	-2.79590274815006	-6.86721129334652	-1.61518882680836
C	-1.77991973201144	-6.44185850711131	-0.84354229847116
C	-0.57568433150341	-4.72338844290418	0.37262143471798
C	-0.87084049335404	-7.39427766836387	-0.42238169149321
C	0.19758937627052	-6.98811647273999	0.39553821461357
C	0.32794775515625	-5.66441921728698	0.80470355358673
H	-0.49732708051515	-3.69863398762961	0.70808860339210
H	1.14869412814774	-5.40553331951548	1.46086449250922
O	1.07655716857092	-7.88088524200675	0.86566268194456
C	-1.00145699178678	-8.81055156753889	-0.71547590177012
C	-0.00511422309273	-9.63823463245516	-0.41422361410561
C	1.28569878169919	-9.12379217026748	0.16195430983697
C	2.30300286602698	-8.86287898119439	-0.94244072193792
C	1.83652035868152	-10.06591622310560	1.21463531872181
H	-1.92264350226659	-9.16135290194561	-1.16184765744719
H	-0.07317404652710	-10.70319594347071	-0.60653322330351
H	1.09959834273916	-10.22131592835359	2.00528466470773
H	2.74188512094528	-9.64557816265211	1.65822831708560
H	2.08210049956021	-11.03268785003789	0.76789534582755
C	-7.22291579456583	2.84537779743103	1.38078430577561
C	-7.19982543722632	1.96363368093127	-0.97235122925694
H	-6.67171596122856	2.88141199287189	2.32273539898631
H	-8.19029377813506	2.37103705796865	1.56032618433214
H	-7.39349614476839	3.86750229548275	1.03303898043804
H	-6.65505108670319	1.33087196203891	-1.67696750097110
H	-7.31256811132649	2.95450425335060	-1.42062369848693
H	-8.19247594222485	1.53512665879255	-0.80820740195231
H	1.91544331413833	-8.12469513486934	-1.64864424229161
H	2.51182031062030	-9.78415461824091	-1.49288883018662
H	3.23710413056570	-8.48662240592369	-0.51637349425834
C	-4.97574282475696	-6.67241336929464	-2.52584458408831
H	-4.37824472131178	-6.13220231544540	-0.55169105883352
C	-6.33237889298278	-6.44344624060410	-2.32313849602204
C	-4.56832399118698	-7.40318455876432	-3.63585989325928
C	-7.27058978951791	-6.92967955010172	-3.22020805026320
C	-6.85995513820359	-7.65912968753524	-4.32742112654940
C	-5.50796649087617	-7.89541642786432	-4.53008629606230
H	-5.18086007890294	-8.46859894665155	-5.39022155948579
H	-3.51370926806133	-7.59121063100162	-3.78927911667125
H	-6.65823324879827	-5.88131542764194	-1.45389940120862
H	-8.32514113834574	-6.74506537695828	-3.05030709760437
H	-7.59204015212313	-8.04537837026092	-5.02716435815064

Cartesian nuclear coordinates of $(S_1/S_0)_{pyr\text{-}equatorial\text{-}top}$ (ωB97X-D4/cc-pVDZ + C-PCM CH_2Cl_2)

C	1.92638360982875	1.92367682385969	-3.34908685252322
C	3.22371105324565	2.39931476488391	-3.16098155287049
C	3.55391889837017	3.03424636690550	-1.96459065600624
C	0.98369583988356	2.06858022211787	-2.34806467903133
C	1.35074149071377	2.66036052285785	-1.15081140299290
C	2.64046924734069	3.16388043195371	-0.89921893036982
H	-0.01700595989978	1.68640295064713	-2.50664840685514
H	0.62163186297682	2.70291120229726	-0.35005890101477
O	4.84056755984002	3.46533649652779	-1.87724247983001
C	5.10572303773334	4.51530774929299	-0.96848460225953
C	3.01359187561032	3.74124328508916	0.43592077215749
C	4.48438484290521	4.19255746913408	0.39553334178903
H	5.09513268667711	3.38241916550356	0.80811097837725
H	4.66162888543542	5.04392776538938	1.06379314390390
C	2.11661359026626	4.86029866984657	0.43053070318723
O	1.55637252172124	1.36740993160971	-4.53380319955447
C	4.16282520055361	2.24883984121223	-4.26195806029404
C	2.56162145150936	0.67541007378346	-5.28353954474883
C	3.84983214888172	1.45911430867545	-5.28560229485689
H	4.52192324539901	1.32415635062013	-6.12653891060835
H	5.09730000465222	2.79291647484545	-4.22051481965846
C	6.59411029041666	4.67986654666024	-0.88685478643868
H	4.69352233732676	5.43873336465077	-1.39182126526305
C	7.44247577011689	3.58234102963782	-0.99199108860218
C	7.14369237092678	5.93280769570661	-0.63773889691430
C	8.51347210737955	6.09076700565622	-0.48919811699582

C	8.81305798635163	3.73883594333981	-0.84836767026544
C	9.35416207642254	4.99163206089375	-0.59457254059705
H	9.46393924803489	2.87578298737949	-0.93640669739869
H	10.42614136762521	5.11176533616902	-0.48374706441150
H	6.49046342406645	6.79686474048798	-0.56361724378802
H	8.92666624508128	7.07506776532584	-0.29800628848362
H	7.01720058783785	2.60877202475168	-1.19838886301298
C	2.07481738663356	5.98382945502838	-0.58858195053510
C	1.07815578555970	4.97496664107548	1.39038191036510
C	1.52585881040690	7.27473418771973	-0.03652646702490
H	1.45460309330142	5.63865387967579	-1.42049906016618
H	3.06508793672965	6.15381454155452	-1.00157603227270
O	0.22118537273896	7.04586454443670	0.51590792348862
C	0.10761434337268	6.01132508523783	1.32936446624620
C	0.91269944733099	3.95853194850597	2.36201644898853
C	-1.01198680686060	5.99121434894661	2.14478438519082
C	-1.13374128442845	4.94780774978249	3.06815677876615
C	-0.15238876514500	3.94788509137209	3.20549708487258
H	1.67936365509846	3.19708749359105	2.33025061891250
H	-0.30472362016259	3.17624001073279	3.94697958711749
O	-2.15954372273011	4.87852852962913	3.87959956533465
C	-2.00562473609088	7.05411095715542	2.17465134953145
C	-3.09986140388909	6.91040536810916	2.90987227328031
C	-3.38583402006626	5.64002634883177	3.65601704480774
C	-4.31346351599707	4.73656524569006	2.85847154651753
C	-3.91920444656359	5.91308843796072	5.04783907181412
H	-1.80865099391364	7.95574501731998	1.61068502446943
H	-3.84756145788671	7.69257983229026	2.97436000643422
H	-3.22040657370164	6.53677704423846	5.60867461007974
H	-4.06518949119326	4.97472224499448	5.58679153659004
H	-4.87847413320221	6.43179090138606	4.98389351333665
C	1.98531392939314	0.56068340560894	-6.68167582632022
C	2.77776251016566	-0.69984917075459	-4.65956071728186
H	1.82209330387868	1.55503866719411	-7.10287746529333
H	1.02840381386064	0.03439135337730	-6.65131615708449
H	2.66762962689007	0.00919980061589	-7.33393494967992
H	3.12169095990330	-0.59652080761614	-3.62778593875349
H	3.53375198813470	-1.25981204139639	-5.21714913806884
H	1.84313471157788	-1.26823421604556	-4.66278684885950
H	-3.87466506701377	4.50052609785690	1.88638931138270
H	-5.26839822967814	5.24007641970106	2.68847125077468
H	-4.49768803924533	3.80558461382862	3.40068420108533
C	1.41790508065304	8.35972897456058	-1.06328221756508
H	2.15713710492661	7.61814301412818	0.79125155657154
C	2.40731600082682	9.33098685643336	-1.14132313397407
C	0.37016584218637	8.37507567312281	-1.97830318976081
C	2.35964798040981	10.30432122638154	-2.12908518226540
C	1.31324701170482	10.31737391336926	-3.03839083202886
C	0.31729871189277	9.35278480993070	-2.95846292637058
H	-0.50407589335993	9.36133836347245	-3.66558080361228
H	-0.40890161010651	7.62498902252609	-1.91656064819808
H	3.22235308094783	9.32798908664255	-0.42528630658246
H	3.13822357847746	11.05620188078482	-2.18301865702054
H	1.27132683900011	11.07935078329607	-3.80812974100787

Cartesian nuclear coordinates of $(S_1/S_0)_{pyr}$ (ωB97X-D4/cc-pVDZ + C-PCM CH_2Cl_2)

C	2.17234596930898	1.97350199797122	-4.17951157409103
C	3.46306655838175	2.07911509445611	-3.65530483993550
C	3.65398607328191	2.66900299765972	-2.41061846351425
C	1.10408441880298	2.44617809191203	-3.45628683148896
C	1.32545100462646	3.04208675110301	-2.21486151647330
C	2.59401200386531	3.18159554415420	-1.64396797019470
H	0.10400495628564	2.35049630722199	-3.86114482985537
H	0.45428711222633	3.36341909961795	-1.65140573872563
O	4.94520690334338	2.66210295529633	-1.96237786547121
C	5.27799898018418	3.29159178828645	-0.74286569047921
C	2.87881334786428	3.71671682197070	-0.28519822518169
C	4.27137869342054	4.38262314294495	-0.39775901383910
H	4.58772380636725	4.81957335628978	0.55608972207719
H	4.41793560495954	5.16545160163112	-1.17322921034373

C	2.01796230828997	4.82367772226984	-0.03241659591527
O	1.97252408564527	1.46012379205880	-5.43172696097651
C	4.56083849807908	1.56940614642584	-4.46339183348513
C	2.87675297056813	0.44775736608139	-5.87498155908929
C	4.29761915476282	0.82000345009203	-5.53039590189862
H	5.09050394443807	0.42290466323120	-6.15589801171747
H	5.57369155065907	1.81595837612119	-4.17309595015591
C	5.49196922195363	2.28272296808542	0.35459284864230
H	6.24188611543143	3.77590601114051	-0.93643295274463
C	4.73610065431817	1.11842157304825	0.43754464930189
C	6.46300987114635	2.52311336893614	1.31775404760195
C	6.67037497731042	1.63106361809114	2.36220010286998
C	4.94903493275199	0.21953231747428	1.47024135502408
C	5.91238807293910	0.47336184315834	2.43978750522702
H	4.35646173526642	-0.68757563014824	1.52187232114311
H	6.07557664747937	-0.23329783982242	3.24618517942632
H	7.07009494966550	3.42119642686023	1.25027271372886
H	7.43277696581159	1.83582597736097	3.10596860227939
H	3.97825631176015	0.92543752869102	-0.30838803329927
C	1.87768607550841	6.03444655769871	-0.92165508436224
C	1.17123148525598	4.86766516909711	1.08605922094534
C	1.53013854579365	7.29522910082975	-0.17652527698068
H	1.07308684844970	5.76321136591326	-1.61579650334559
H	2.76318295895459	6.17346548918675	-1.53396527820876
O	0.33891284429823	7.07176883289453	0.60532663884850
C	0.27473563875081	5.95571518809306	1.29540333815075
C	1.08383537008000	3.73339390223559	1.93538219458699
C	-0.72086541433656	5.87587825287503	2.25374383221790
C	-0.78010396148203	4.71958652765678	3.04096736387376
C	0.13946106018529	3.65588568035766	2.90429502061274
H	1.80692235499927	2.96200510355527	1.70976465994367
H	0.03752823337932	2.80395029313731	3.56128807952518
O	-1.67767663302006	4.58610066938446	3.97576397957791
C	-1.63451750629368	6.96607173542453	2.56042189631070
C	-2.60464384990848	6.78551358092324	3.44573791459164
C	-2.84772767364003	5.45925219273839	4.10159499472727
C	-3.99559169016848	4.72006848275947	3.43334524433817
C	-3.05550418884821	5.59969984341637	5.59625240413034
H	-1.47812061129924	7.91697161975474	2.06981938291259
H	-3.28508851131425	7.58610120190165	3.71100849693282
H	-2.20310138571672	6.10398501431493	6.05538922310963
H	-3.17334279589473	4.61621504402195	6.05543556926971
H	-3.95608118829516	6.18624068623171	5.79189915245309
C	2.67664342112258	0.38683367533243	-7.37771334357797
C	2.50262893603939	-0.87676685774333	-5.21510711402798
H	2.93611720284337	1.34725115042821	-7.82860860530242
H	1.63146123272219	0.16644018732223	-7.60804132616135
H	3.30496495683649	-0.39152560963440	-7.81938237057433
H	2.58100941561601	-0.79077197205757	-4.12895693093840
H	3.17407059948206	-1.67500808979262	-5.54435669147279
H	1.47481015436034	-1.15035496222214	-5.47189048434474
H	-3.78821322564339	4.57088661568524	2.37137005196098
H	-4.91484563459563	5.30400721332048	3.52299637798440
H	-4.14925823034923	3.74720458005952	3.90629073081533
C	1.28468126971662	8.48160289837411	-1.05683009802067
H	2.32607749147037	7.53094197747668	0.53886812061303
C	2.28636650210169	9.42834424860111	-1.22572887512795
C	0.08401572190679	8.62496836027663	-1.74470631886755
C	2.09691146073550	10.50532276380143	-2.07984376522738
C	0.89785825165609	10.64699008405597	-2.76111486588067
C	-0.10905656495364	9.70588858421493	-2.58945767436810
H	-1.04880252029407	9.81397403630543	-3.11824050263594
H	-0.70475876906322	7.89461227839629	-1.61032628574308
H	3.22192887118478	9.32517446370021	-0.68646977641312
H	2.88568057359810	11.23741149783421	-2.20670415722373
H	0.74579447687358	11.49034611218976	-3.42443203807306

Cartesian nuclear coordinates of $(S_1/S_0)p_{yr\text{-}axial\text{-}top\text{-}CC}$ ($\boldsymbol{\omega}$B97X-D4/cc-pVDZ + C-PCM CH_2Cl_2)

C	0.08770178306538	0.11981825573899	-0.01011406549466
C	1.48075396393547	0.08872844867594	0.09875801771811

C	2.16998557289817	1.29332990680154	0.08368589432959
C	-0.62170985928788	1.32321962999044	-0.14980914607294
C	0.06884202582940	2.49742123622223	-0.13440031564889
C	1.47488183810699	2.52797993488349	0.00097266462254
H	-1.69770743178535	1.28028837566101	-0.24683124240403
H	-0.39383768493993	3.47235091653700	-0.19763037945788
O	3.49014876536842	1.20817344745521	0.15779242774031
C	4.22927488257565	2.39018934616892	-0.17832666107954
C	2.13979532238438	3.77165385375260	0.19553726098977
C	3.60360416260168	3.59264047925462	0.48247878582652
H	3.68104671570590	3.49631518682646	1.57372167445533
H	4.16410196411689	4.47862470010419	0.21653771720940
C	1.42627953263238	5.01579073209844	0.10609251217102
O	-0.61851077964789	-0.99199600040756	-0.03450415601410
C	2.13854197756025	-1.20895404460006	0.11382567769226
C	-0.07434887053019	-2.26151349454483	0.42536895651038
C	1.41327428331862	-2.31379943266778	0.22896227615671
H	1.87114149874819	-3.29624318426142	0.21837394172324
H	3.21381470899320	-1.23895414240245	0.00088199998260
C	5.65493224343862	2.20088787618276	0.24126088192435
H	4.17236052044942	2.50673786047987	-1.26541159801973
C	5.97464537634615	1.54960625118259	1.42705471364238
C	6.66799713186504	2.77054680831728	-0.51904296414050
C	7.98716816212590	2.69811992450517	-0.09745522861033
C	7.29366811336077	1.47176761332748	1.84569990979009
C	8.30313602394074	2.04807730037353	1.08647653508865
H	7.53545240289861	0.95856466411879	2.76945591536490
H	9.33413224342508	1.98618422973116	1.41537315402489
H	6.42223313662412	3.28187641974246	-1.44361622382502
H	8.76990907556114	3.14671365930097	-0.69806137664072
H	5.18792230314280	1.09905449385489	2.01959699048416
C	1.62552800199247	6.03000122991083	1.23191147537875
C	2.07917262915335	5.47978685303632	-1.14851702636063
C	2.80774244591190	7.00729465991766	1.14345822162602
H	1.64379244729302	5.52933246367581	2.20418525943786
H	0.73825178707448	6.67122600616489	1.25889157217179
O	2.87673601416298	7.55781967584919	-0.15989818545153
C	2.77347234080264	6.71075720966724	-1.22040996562366
C	2.04526236468801	4.73963127139427	-2.34663305016357
C	3.33939425598542	7.17573133987631	-2.40159412308561
C	3.30081216129452	6.37669351828223	-3.54828956874239
C	2.65795127680550	5.15643700357167	-3.51840669470902
H	1.49133816487958	3.81018263938770	-2.36086009997406
H	2.61043846390901	4.56133463354778	-4.42180762728267
O	3.83168937482587	6.81756098447791	-4.71770577721239
C	3.98245982106592	8.47743765231518	-2.51246662037249
C	4.72544577780515	8.75192656442441	-3.58091510011020
C	4.94843054886812	7.71038750360772	-4.64775799392541
C	6.20375705272711	6.89837642572466	-4.34154312884239
C	5.02408825805824	8.34021826511977	-6.02514037685565
H	3.83156376265285	9.19757990443932	-1.71911698741214
H	5.21687533844310	9.71160587103263	-3.70145328155909
H	4.11099963120729	8.90278183122250	-6.23072121644109
H	5.13356573872703	7.56463424285144	-6.78654997945945
H	5.87874230788757	9.01886829660448	-6.09010548451786
C	-0.78598621737269	-3.30474384589411	-0.41294152751411
C	-0.43148744504497	-2.38079667604812	1.89981804447598
H	-0.53222072968053	-3.18249846521999	-1.46780644369717
H	-1.86758101797963	-3.20748941062581	-0.29809215133162
H	-0.48939305707462	-4.30703901959823	-0.09454376478633
H	0.03809533302538	-1.57713196825724	2.47183737055152
H	-0.07388914409996	-3.33469645721625	2.29595655505178
H	-1.51471422448126	-2.32669025662663	2.03517288350915
H	6.09951514156368	6.39280502873922	-3.37806861196451
H	7.08046751608285	7.55026961698025	-4.29168287239138
H	6.36667153449672	6.14509997430560	-5.11782929140799
C	4.15669608776562	6.47871093665874	1.57466501039507
H	2.58473245878715	7.87261746147746	1.77551177545968
C	4.30053141876652	5.86096425672150	2.81518569972252
C	5.27787042768793	6.58856893270014	0.75726328545608

C	5.51242903578852	5.30850560987236	3.20055191409015
C	6.61045734106708	5.37151413291789	2.35317550795693
C	6.49025556390536	6.02933489025587	1.13913234633730
H	7.34531818114179	6.10257047106034	0.47664342729792
H	5.19460316634855	7.09225606850045	-0.19553429877473
H	3.44891256195359	5.79187695111030	3.48313920442524
H	5.59811562810193	4.81721280029685	4.16356813954970
H	7.55065534503767	4.91548092902327	2.63883708331742

Cartesian nuclear coordinates of (S_1/S_0)pyr$_{\text{-CC}}$ (ωB97X-D4/cc-pVDZ + C-PCM CH_2Cl_2)

C	-0.05152400929859	-0.10504242316516	0.08335574933728
C	1.31404926078602	0.11670415422008	-0.12658427535232
C	1.77805010606494	1.42282430847321	-0.09568849566826
C	-0.95683261076911	0.94927814514007	0.28735214807500
C	-0.48131447332924	2.22609029376614	0.32489077764783
C	0.89520881308435	2.50154214059885	0.16715252504848
H	-2.00323773854200	0.71187124762166	0.42015896828299
H	-1.10156012537642	3.09431349555803	0.49985069639401
O	3.07138689626061	1.59917550499797	-0.33539374796278
C	3.49113712600384	2.93931885611734	-0.63305128449280
C	1.38261238956937	3.81378950399078	0.37922909564651
C	2.89059926494037	3.88089968412368	0.38541493410133
H	3.22487027113979	3.58006964427041	1.38763804238077
H	3.23672472970131	4.89666439563372	0.21615411921342
C	0.52766063624602	4.95522859777241	0.56600922179458
O	-0.55585886431707	-1.32213144361453	0.04359203158838
C	2.14775621525631	-1.02340092834359	-0.47561247479662
C	0.28307587231571	-2.50403027285418	0.17642161787503
C	1.65703672184797	-2.25203029190324	-0.37485915017019
H	2.24131573403670	-3.12141941353811	-0.65347593948301
H	3.14729739939148	-0.83356197098444	-0.84303731460226
C	4.98764905494447	2.97690504847039	-0.66721236278777
H	3.09836366559275	3.18943451887125	-1.62319329645783
C	5.75814894787280	2.14420089860072	0.13491931927517
C	5.61272252817645	3.91331017709774	-1.48399221409896
C	6.99452896592595	4.02218683424100	-1.49237875077213
C	7.14139677714037	2.24871445219603	0.11918870394732
C	7.76233255502429	3.18871429407937	-0.69094230051588
H	7.73696747115078	1.59199566728218	0.74299649720142
H	8.84314664054205	3.26917166323046	-0.70091750058823
H	5.01351348773251	4.55936440691398	-2.11767525578042
H	7.47253040921299	4.75511694429607	-2.13177317709219
H	5.27331873010602	1.40789240769761	0.76343597543645
C	0.98740807014420	5.77069766457239	1.78104370721011
C	0.92404004857474	5.66495399945553	-0.69002451818081
C	0.72679053119330	7.24165889523497	1.52590777810958
H	2.05967940922353	5.71488196865368	2.04891163797086
H	0.43950327312017	5.46349221536649	2.67785605609069
O	1.56588385021321	7.68115472870912	0.46615252802070
C	1.47772486845884	6.96274691148915	-0.68159031767035
C	0.81756351496286	5.06379121241537	-1.95518372342974
C	1.93483363225220	7.58716975660075	-1.83324175359273
C	1.88461586346650	6.89905275403853	-3.05258258154284
C	1.30694140212976	5.64861153910953	-3.11708777786117
H	0.33490280663886	4.09731804996554	-2.02681669885370
H	1.22289430822386	5.15406133429290	-4.07666972391533
O	2.31592519654272	7.48857738955962	-4.19496028379662
C	2.44862456409283	8.94783783596968	-1.84795115306240
C	3.09416464141277	9.39031377020027	-2.92307226142129
C	3.35190854229497	8.47293960967717	-4.09134244033910
C	4.68758559165000	7.75491703100534	-3.91869349786354
C	3.29783205205202	9.22588287861472	-5.40644595351634
H	2.27736719221035	9.57235118876251	-0.98075428612701
H	3.48165275350027	10.40196048369889	-2.97938105304985
H	2.32795557937373	9.71508984383920	-5.51808385629662
H	3.43691551402945	8.53513335854671	-6.24120590760749
H	4.08284608785281	9.98568215978403	-5.44533233701123
C	-0.44719626946202	-3.57655472822166	-0.60669590123915
C	0.34711590419950	-2.83235156933719	1.66101541313325
H	-0.50302448981753	-3.30526338551969	-1.66272217453774

H	-1.46137030770517	-3.69998792954042	-0.22151791370359
H	0.07914648995999	-4.52976484777344	-0.51675116621092
H	0.81493972546550	-2.01356463116898	2.21263275346730
H	0.94345657251110	-3.73447464799585	1.81990607209097
H	-0.65742452901121	-2.99966896916067	2.05769937785287
H	4.68540130104694	7.17156189253591	-2.99430618948575
H	5.50670859796566	8.47704463863451	-3.86115785949920
H	4.86761341022282	7.08194108888146	-4.76213452503895
C	1.01005439546213	8.09935817461299	2.72466076728474
H	-0.31675106308638	7.36988927813845	1.21826025374034
C	0.04583778808717	8.21693834430363	3.72084737625819
C	2.23040572684931	8.74405070116689	2.88924697785468
C	0.29665755211010	8.95757430602960	4.86517722626024
C	1.51817445012124	9.59761715468660	5.02531621597801
C	2.48131110451545	9.48954823129243	4.03290915939562
H	3.43608959453807	9.99079811738473	4.14801927565194
H	2.97763188457007	8.66282981882433	2.11090065946983
H	-0.91295311726614	7.72451596137059	3.59520116889675
H	-0.46620146120397	9.04175421148957	5.63123541718586
H	1.71534475336228	10.18166695364962	5.91714629699798

Cartesian nuclear coordinates of $(S_1)_{pyr}$ ($\boldsymbol{\omega}$B97X-D4/cc-pVDZ + C-PCM CH_2Cl_2)

C	0.79023306473753	-0.96956227207760	-3.61894918189826
C	-0.04502908045875	-0.24823643524983	-2.76246611671289
C	-0.73327734242457	-0.91699761748266	-1.76331948441491
C	0.90498174119135	-2.33590730087048	-3.47388377436322
C	0.21171909060424	-2.98695535769833	-2.46321174683638
C	-0.62552884831189	-2.30811487058327	-1.55756629318318
H	1.54831954888048	-2.88352521301770	-4.15193452144948
H	0.35718486209151	-4.05530459506341	-2.34486537246877
O	-1.55065097420076	-0.16613677547502	-0.96647463412104
C	-2.71638453857045	-0.83397235882821	-0.52450057985491
C	-1.32740366630145	-2.93547686611726	-0.46812232451583
C	-2.31971605958512	-2.06871131618148	0.28468081680428
H	-1.92298468190712	-1.71109387164017	1.24509595087846
H	-3.23807152846602	-2.61598462954012	0.53923231912022
C	-1.79483338063321	-4.26573698963561	-0.73626146208724
O	1.42926684417617	-0.34637047474742	-4.65034452325818
C	-0.17923548647608	1.18213903388888	-2.99734008664623
C	1.79868574972905	1.02203947372075	-4.46333487828148
C	0.67093330452710	1.79011379472591	-3.81916258629884
H	0.60381494043719	2.85405822796337	-4.02193374461411
H	-0.97578907514913	1.71560822046013	-2.49483969356818
C	-3.52981242427822	0.13474946251628	0.27986510456320
H	-3.29273571939470	-1.14942825647081	-1.40485424294538
C	-2.91274749404841	1.11438247178927	1.05176226670839
C	-4.91544778482630	0.02447836989509	0.31406497074597
C	-5.67502661603783	0.87022706659247	1.10858086827748
C	-3.67113151368608	1.96365399729441	1.84373090029355
C	-5.05358232326819	1.84403539205108	1.87779356087899
H	-3.17903005209253	2.72609261099874	2.43754706973491
H	-5.64438642833669	2.50964170719834	2.49691641334019
H	-5.40636126636461	-0.72948374661387	-0.29334840012855
H	-6.75507874094506	0.77407824798758	1.12140553515113
H	-1.83533704848554	1.21004021317005	1.01338510601380
C	-2.86895101268647	-4.51225067262728	-1.75549414437452
C	-1.26416135607166	-5.41459773331329	-0.13705001789416
C	-3.60406974750958	-5.82289363175239	-1.57486990088465
H	-2.37830376144828	-4.46367351336174	-2.73649214115615
H	-3.58200579329521	-3.68598313018710	-1.74151442448300
O	-2.66961768275733	-6.89921732753674	-1.41020429762994
C	-1.68016394691068	-6.71338624272065	-0.54576121275034
C	-0.20587051858487	-5.31543030185073	0.80215521435295
C	-1.04674096332838	-7.84841908180784	-0.07009013185203
C	-0.00589425935914	-7.68965773644378	0.85253252276869
C	0.40352498850906	-6.42248554334072	1.30547514269689
H	0.08932447332846	-4.30847566836899	1.06465653732668
H	1.21270903126259	-6.36875182319622	2.02049778231734
O	0.61392660084901	-8.72937745419325	1.36886649124854
C	-1.46277045542392	-9.20105338723434	-0.40821871158178

C	-0.74792890800008	-10.23557328436434	0.01427565104097
C	0.53193133857209	-10.05841822449263	0.77806356772124
C	1.73597189952134	-10.19969100058455	-0.14156557231063
C	0.61136225006721	-11.00409940353064	1.96038517048522
H	-2.36928948677420	-9.32854032081642	-0.98418163442385
H	-1.04377143471133	-11.25504018322238	-0.20538728209966
H	-0.24923211270220	-10.86487389007750	2.61754235256841
H	1.52361448768958	-10.81768941048284	2.53095197084142
H	0.62293003036798	-12.03962282885049	1.61185875357927
C	2.08187805245466	1.53744832214383	-5.86161832138919
C	3.04810783133681	1.08152662900875	-3.58900644219764
H	1.18004355914288	1.47292246219896	-6.47418217261460
H	2.86454591844651	0.93673707777469	-6.33100544205564
H	2.41192373499154	2.57939648846354	-5.82824505983606
H	2.84570978633991	0.63575066209702	-2.61236778039653
H	3.36077692328296	2.11816453772157	-3.43417182366935
H	3.86726515354538	0.53199694429408	-4.06213583305234
H	1.69184561318746	-9.45988804235472	-0.94416811377539
H	1.74665950634370	-11.19439094091902	-0.59447390118056
H	2.66272244537974	-10.05901350491888	0.42035649590575
C	-4.49837774874726	-6.16578930408367	-2.72696788632645
H	-4.19437733259567	-5.77685701830807	-0.65277092375333
C	-5.85646517107646	-5.88433127761722	-2.65735798563077
C	-3.97261413441042	-6.71313723691762	-3.89330505122007
C	-6.68235875444464	-6.13632060114640	-3.74385333587217
C	-6.15487038048088	-6.68247076353863	-4.90397203470145
C	-4.79858666420216	-6.97327875610825	-4.97468200660622
H	-4.38226094321126	-7.40364609586536	-5.87813206140404
H	-2.91562817548731	-6.94370355934094	-3.94811047881259
H	-6.27398894526952	-5.46668729411816	-1.74715429617269
H	-7.74070121918869	-5.91199332885873	-3.67980799179154
H	-6.79905978806702	-6.88441294820875	-5.75177647381743

Cartesian nuclear coordinates of $(S_1)_{pyr\text{-}CC}$ ($\boldsymbol{\omega}$B97X-D4/cc-pVDZ + C-PCM CH_2Cl_2)

C	-3.06967827955964	4.32271770970507	2.12687525656967
C	-1.87801605407156	4.39745701910581	1.39550252894918
C	-1.27566528830318	3.21467794662423	1.00173043100843
C	-3.64310598550015	3.09361384301790	2.49571436845057
C	-3.05451674674069	1.93986571112732	2.07546963125011
C	-1.87031978435236	1.95662207278277	1.29678503298270
H	-4.55170644674747	3.10450407924168	3.08164975428505
H	-3.46211481256962	0.95834390280800	2.27636424113605
O	-0.13168292263832	3.31771952834030	0.33643256352203
C	0.69013965484932	2.14288993216937	0.27694164941293
C	-1.35821844832744	0.77265049495659	0.74866624279858
C	-0.14929235548789	0.95034629339333	-0.12448971250300
H	-0.50159891418972	1.07550948213007	-1.15786828708099
H	0.45355232385590	0.04283731609381	-0.10379138002593
C	-1.93947033763864	-0.52420471741178	0.96313599709261
O	-3.67943458521119	5.41455117073007	2.54401045197737
C	-1.28784460329271	5.71009791121400	1.18053452524475
C	-3.41601681286851	6.70812491427745	1.93056197344145
C	-1.98997512800079	6.79872733393793	1.46745285508054
H	-1.57432082106727	7.79213139980048	1.34441273019512
H	-0.27100742102442	5.76148701646484	0.81519403420863
C	1.82557537318493	2.41040831027600	-0.66333476663375
H	1.08095543874120	1.97629366569278	1.28640406171517
C	1.66634518261452	3.22644289424212	-1.77754408420861
C	3.04407988303610	1.77690922841219	-0.44827009460857
C	4.09126036918616	1.94960406861541	-1.34052584754501
C	2.71701872967190	3.40496363123216	-2.66508421096771
C	3.92986771524737	2.76538619234752	-2.45124184461005
H	2.58738430150939	4.04705744463583	-3.52878233981401
H	4.74909929323254	2.90562437288227	-3.14682677414934
H	3.17478487653935	1.14372771800109	0.42309425114599
H	5.03690107580703	1.45065818632634	-1.16385456084454
H	0.72178116781652	3.72910313106239	-1.94276503559956
C	-2.33530123567517	-1.21506129867249	-0.32739297724559
C	-1.06452830568133	-1.34745026216920	1.78599455646413
C	-2.76678268497856	-2.63494059294049	0.00449468328923

H	-1.53748541776777	-1.30563986029543	-1.09190813547272
H	-3.17228260394167	-0.70356820671850	-0.81491971932523
O	-1.68404363462210	-3.34797837546309	0.57032606792299
C	-0.97590827601928	-2.73680695144356	1.56052089247261
C	-0.26482044172282	-0.85243809784894	2.82920714248650
C	-0.16481785805227	-3.56576425770174	2.32001781180521
C	0.63089805848885	-3.01311039131691	3.32907718160525
C	0.57571275498866	-1.66098385924948	3.58498011308584
H	-0.31642435562543	0.20263192455605	3.07320752810579
H	1.17774200391008	-1.25012552635652	4.38599282539745
O	1.40302985002581	-3.81250625010915	4.11443639553801
C	-0.10991669908401	-5.00824954185831	2.13166160230869
C	0.85362333524310	-5.70825522423369	2.72262980500852
C	1.91940976292845	-5.01308115762954	3.53207143956569
C	3.10990878780636	-4.65686009959079	2.64649727103723
C	2.34939893182998	-5.85923400407866	4.71492755531173
H	-0.86813576299879	-5.47839075243694	1.51901096124723
H	0.92358800302631	-6.78547498932059	2.61375586561427
H	1.48722549247018	-6.08892719440528	5.34453509841118
H	3.08393387602490	-5.31773725172352	5.31548434010279
H	2.79709254421762	-6.79656048035838	4.37384991477561
C	-3.71681112251979	7.71373697517849	3.02325324557721
C	-4.37524311006497	6.84199707187172	0.75656126294960
H	-3.03207603387610	7.57987184007211	3.86297698865696
H	-4.74061556181513	7.58729830312750	3.38081796920120
H	-3.60476199988999	8.72969589866996	2.63741246831533
H	-4.18204047555477	6.06277933734316	0.01556213853850
H	-4.24123917602926	7.81244219856925	0.27188030819426
H	-5.41000947491418	6.75852415788508	1.09848466975203
H	2.79399057861350	-3.98738693920929	1.84290094260759
H	3.53587073816917	-5.55677390649561	2.19377615946811
H	3.88396050765900	-4.15696758282629	3.23621295977827
C	-3.22517676607792	-3.39417018359178	-1.20913720283419
H	-3.58020908603292	-2.58682915438797	0.73622808753489
C	-4.52869751523214	-3.23221065782030	-1.66750861853598
C	-2.36235140059437	-4.22611541436327	-1.91351973112903
C	-4.96302693951811	-3.88180285926819	-2.81274207175049
C	-4.09610560314409	-4.70932056866276	-3.51338468682537
C	-2.79666267412758	-4.87977522034034	-3.05820537392972
H	-2.11497130210831	-5.52935941160878	-3.59628088830410
H	-1.35279641891190	-4.36400753960091	-1.54989680043361
H	-5.21257810233376	-2.59398012999304	-1.11704873128951
H	-5.98330423354860	-3.74855724175243	-3.15494325769900
H	-4.43422059063936	-5.22299547566654	-4.40627169923139

Cartesian nuclear coordinates of $(S_1)_{TS}$ (ωB97X-D4/cc-pVDZ + C-PCM CH_2Cl_2)

C	-1.26424391867501	3.36320231109524	-2.15817413673288
C	-0.63929269432472	3.86810513786742	-0.99304898844623
C	-0.38340095756419	2.98883612851348	0.03622774764530
C	-1.63546417807706	2.01975707587963	-2.28086275612334
C	-1.36055034523177	1.14910598789326	-1.26555044541075
C	-0.73586205298591	1.60562987749177	-0.05688428510261
H	-2.10224731929767	1.70032036926823	-3.20262604112852
H	-1.59520361306603	0.09798211851261	-1.33366615763710
O	0.21959820855592	3.47906475552163	1.11899836521623
C	0.11502477895277	2.71749275469278	2.32410736374604
C	-0.30579392606988	0.70471112368769	0.89990257394873
C	0.45518107246401	1.26419956002566	2.05877022678370
H	1.52699417767810	1.14481900596268	1.85028865500796
H	0.25508892242818	0.67851927763203	2.95910495288207
C	-0.47994204539278	-0.72172808455794	0.76924761939812
O	-1.55463077220215	4.16593741204426	-3.16713643347599
C	-0.38306837262758	5.29212678159178	-0.91824242535091
C	-0.88935368509234	5.44819824574014	-3.33119219194834
C	-0.53558744270217	6.04879459694527	-2.00173678609934
H	-0.37053311181380	7.11975046250153	-1.97497911039909
H	-0.08967879119091	5.71129685150523	0.03513128265443
C	1.00800953474557	3.34747274014441	3.35007844359051
H	-0.92797567015321	2.78065053463228	2.65505876518735
C	2.23305826618418	3.89838332830675	2.98871231934246

C	0.63827367246938	3.32738159550892	4.68921654211309
C	1.48543449115629	3.84135718542152	5.66021296337193
C	3.07612785145476	4.41885680606731	3.95824317401698
C	2.70674288750144	4.38888338865214	5.29618255350903
H	4.02691710384770	4.85074256058294	3.66757067714056
H	3.36779398840469	4.79545266413627	6.05266192092492
H	-0.32083090995695	2.90913312637539	4.97675286441182
H	1.18674242727152	3.82057985517977	6.70186150999935
H	2.51830195374456	3.92702731517712	1.94459328851691
C	-1.33443410145063	-1.47302872835226	1.74267285765413
C	0.27341568830690	-1.42752390125594	-0.18436229110785
C	-0.77403825315292	-2.87897945363992	1.93192464993120
H	-2.38745219624044	-1.57048666402646	1.43405648943803
H	-1.35939066141385	-0.98369909646303	2.72606418797401
O	-0.61398893210344	-3.50412029990398	0.67040717089442
C	0.18047836965752	-2.84968577594924	-0.22173257380828
C	1.11385606794531	-0.84373333474724	-1.16796412434571
C	0.84227105197233	-3.61491311353149	-1.15559522158083
C	1.64627191244453	-2.98125975352231	-2.12111476790346
C	1.78204437815422	-1.61064477841836	-2.11310536083103
H	1.25056667054587	0.22806073121200	-1.18249408404735
H	2.42044579315930	-1.14469103713560	-2.85365698011325
O	2.35923284515271	-3.72072454898604	-3.01180984725476
C	0.76878083130123	-5.06775499215260	-1.18623504092203
C	1.24878349026424	-5.72436419290238	-2.23813446876756
C	1.81984808143651	-4.98198997646338	-3.41956245581801
C	0.73505207442086	-4.73488425317508	-4.46409764025766
C	2.99861783226494	-5.72735654774684	-4.01623914517849
H	0.32475476032581	-5.58001269957942	-0.34256785878739
H	1.21171255675096	-6.80719848676723	-2.29590269219598
H	3.76777647896021	-5.88299154417132	-3.25670800541367
H	3.43157135493881	-5.14865511503175	-4.83552218744825
H	2.68228784377011	-6.69992655203252	-4.40276691004822
C	-1.89371771417885	6.30084025439575	-4.08057362092891
C	0.36211658340204	5.18961983771295	-4.15910088598571
H	-2.79221091115771	6.44683228155520	-3.47755506293484
H	-2.17452242824186	5.81751487335533	-5.01872402077875
H	-1.46068541950053	7.27826958504786	-4.30679203728544
H	1.03629707583119	4.51488311984864	-3.62656493605697
H	0.89307797211563	6.12737955348610	-4.34190879880076
H	0.09763604250604	4.74100016841274	-5.12019612113152
H	-0.06740381726689	-4.12861089023618	-4.03725072248503
H	0.30480461947300	-5.68096883114043	-4.80468311719968
H	1.15378939140069	-4.20643939839018	-5.32544985087498
C	-1.66808626314961	-3.74911987245574	2.76518106347208
H	0.20988478012731	-2.80162062897885	2.41046107775521
C	-1.54268118008992	-3.74596502808643	4.14985404389744
C	-2.66123479086357	-4.52627497997062	2.17851075839642
C	-2.39750986305001	-4.50013683557949	4.93973975128557
C	-3.38774100055901	-5.27254709892446	4.34912624945117
C	-3.51401843896235	-5.28454106286734	2.96704327882459
H	-4.28133365479220	-5.89080971340292	2.49847822207854
H	-2.74895722149887	-4.54062670966707	1.09995536780708
H	-0.76234018112093	-3.15165892773093	4.61456404981336
H	-2.28505806286983	-4.49113095524425	6.01811368257073
H	-4.05420198539976	-5.86736347639653	4.96338887749603

Cartesian nuclear coordinates of $(S_1)_{TS\text{-}CC}$ (**ω**B97X-D4/cc-pVDZ + C-PCM CH_2Cl_2)

C	-2.12322478872877	-1.78602072512178	4.16187636685025
C	-2.22081497873750	-0.74072804574271	3.22598353786127
C	-2.30177220779430	-1.04371939756653	1.88434145448548
C	-2.09295244030255	-3.09884916032109	3.73681710819478
C	-2.15577134024352	-3.39823858843387	2.38582172921407
C	-2.26230556831273	-2.38383284243431	1.39526273436975
H	-2.01749055534083	-3.88521023002871	4.47744285882909
H	-2.17542332376850	-4.43427387009381	2.08421811645320
O	-2.44079137847328	0.00006303702994	1.02639942349331
C	-1.95061426139585	-0.19310948293822	-0.29058516760971
C	-2.34101855908294	-2.60701731890023	0.00914943519938
C	-2.58216337733604	-1.43817427890367	-0.89626033269242

H	-3.65088845850334	-1.22302454069000	-1.06626702095404
H	-2.14235158566060	-1.59910831682104	-1.88698672305743
C	-2.44870114050610	-3.94322311284456	-0.51242385901121
O	-1.98719027486393	-1.51544821088193	5.48159794021971
C	-2.20445684441162	0.62319319025325	3.73185376061194
C	-2.56690127923562	-0.30655244942259	5.99072813163699
C	-2.33899520234713	0.83772860643333	5.03690463264632
H	-2.32748307505521	1.84022974984231	5.45132388095701
H	-2.07539976702445	1.43349722961707	3.02624012372072
C	-2.24978658882528	1.04899000675670	-1.07797765458105
H	-0.86559573766458	-0.34121238982018	-0.24055040278270
C	-3.38275443472252	1.81054575325741	-0.81276093851341
C	-1.41389606678497	1.42108600420049	-2.12448703324099
C	-1.70594408295291	2.53220708772956	-2.90137488101580
C	-3.67334631096023	2.92462932096629	-1.58616830838235
C	-2.83860892896146	3.28774416112465	-2.63378525904346
H	-4.55649664434417	3.51470233439880	-1.36776303230381
H	-3.06689259204237	4.15970018969108	-3.23614461854656
H	-0.52217914362372	0.83758601418073	-2.33015542964623
H	-1.04364312670542	2.81290208741205	-3.71254425890861
H	-4.02510275907176	1.53032928337442	0.01210318974823
C	-3.65791004068680	-4.34852646838096	-1.29877816123526
C	-1.44796091451232	-4.88518968698505	-0.36009623873001
C	-3.90944343031798	-5.84249570851190	-1.21120884023152
H	-3.52010557113754	-4.05756991687901	-2.34994043998395
H	-4.53791336938054	-3.80874538057029	-0.94296129254412
O	-2.72014174466299	-6.56582980529046	-1.52576295739059
C	-1.59582972856664	-6.19636163227742	-0.90249462663757
C	-0.20630887420036	-4.55645100668157	0.28540794066255
C	-0.57302502300110	-7.12058332580484	-0.85484626855960
C	0.63485972110208	-6.73910147403516	-0.22335361586771
C	0.81057460206440	-5.47277530537938	0.33638542190465
H	-0.09070112940249	-3.56236003396405	0.68428534464316
H	1.76046109997125	-5.24810867114566	0.80271618679284
O	1.66412668554416	-7.57355450095572	-0.17225778377634
C	-0.64175964208185	-8.43255865768471	-1.46199966090450
C	0.34170302511046	-9.30831748532973	-1.26539933592011
C	1.49785854285410	-9.00244477866662	-0.35861413202053
C	2.81413507274868	-9.46050947808815	-0.95667836095155
C	1.27075571695354	-9.59993092485410	1.02410381641650
H	-1.49846007143161	-8.66944869293627	-2.07865900575748
H	0.32182889969206	-10.29424963453511	-1.71540863069738
H	0.35500230752890	-9.19752777293979	1.46348587383222
H	2.11166159081546	-9.36627228215411	1.68254008879047
H	1.16784046685538	-10.68607987756713	0.95504271469980
C	-1.85844143654718	-0.06983096236430	7.31044274054658
C	-4.06068439924556	-0.53737876414801	6.19537127984897
H	-0.78913181690027	0.07447748301390	7.14205980622493
H	-1.99413805343082	-0.93126567361494	7.96826245142217
H	-2.26048259114520	0.81724128033199	7.80696946248388
H	-4.53730233537371	-0.78668832870990	5.24441102728821
H	-4.53796613977401	0.36340396833638	6.59133491244989
H	-4.22343719168852	-1.36112796057093	6.89625643489447
H	3.64315987655491	-9.17941974351020	-0.30357192486288
H	2.96585323023051	-8.99973125056887	-1.93471693339880
H	2.81589607791089	-10.54678386529059	-1.07616892287264
C	-4.99021777509301	-6.31143753592783	-2.13946809829348
H	-4.18048722965388	-6.09154404416776	-0.17890472536691
C	-4.70293585013767	-6.64608082269915	-3.45860185908967
C	-6.30540887367853	-6.36432504681240	-1.69454752034048
C	-5.72091872268775	-7.02763446675367	-4.31894064787949
C	-7.03438540687458	-7.07266908180331	-3.87196786787490
C	-7.32504628117627	-6.73919206638472	-2.55721380335905
H	-8.34713023057073	-6.77875037892014	-2.19875653135385
H	-6.53479399044042	-6.11523259286231	-0.66359907736630
H	-3.67715819468864	-6.61875612649819	-3.80397876364993
H	-5.48748084554297	-7.29285837931060	-5.34382345661789
H	-7.82893318810809	-7.37168423443037	-4.54575552357095

Cartesian nuclear coordinates of $(S_1)_{(P)\text{-}(Z)\text{-}\mathbf{2}}$ ($\boldsymbol{\omega}$B97X-D4/cc-pVDZ + C-PCM CH_2Cl_2)

C	-7.01290742599046	0.37983808278298	-0.03369764110846
C	-5.66016503983276	0.79104822315798	-0.20012919673454
C	-4.66960232114964	-0.15377541518386	-0.05628815572778
C	-7.33957455575842	-0.90050851367251	0.32678604801973
C	-6.34413033234815	-1.89778760402406	0.42107663600760
C	-4.95409802394517	-1.51715826924395	0.18894713821280
H	-8.37517132801167	-1.13495712429810	0.53628459750186
H	-6.51349873907710	-2.70754895088389	1.12203433417125
O	-3.38919451457233	0.24585088126263	-0.21922112982030
C	-3.93945381314183	-2.47306880872698	0.04876120155306
C	-4.24439436653632	-3.83693051763009	-0.11599783711378
C	-3.24274922142292	-4.90545748403243	0.21457425296322
C	-5.50861945169918	-4.24739004989722	-0.55916907400638
C	-3.69719501068904	-6.25855846290049	-0.30447347946535
H	-3.09375581621634	-4.96976285610017	1.30001402356818
H	-2.26258187005411	-4.68348745109808	-0.21807322022790
O	-5.03968400618054	-6.50171371127844	0.09178156898243
C	-2.84262486293994	-7.38448558794363	0.19911372378792
H	-3.66956079715355	-6.24238916793598	-1.40059430917829
C	-5.92749156035806	-5.58109144310256	-0.34386270608038
C	-6.48090832630448	-3.30072012060549	-1.09745297085701
C	-7.23574682903502	-5.97766595590972	-0.50517733331271
C	-8.17905136865748	-5.01561118896861	-0.96403255470463
C	-7.80466379184534	-3.74409960718567	-1.30923604204412
H	-6.09667066613982	-2.54107341431133	-1.76915044817530
H	-8.54242465139733	-3.08491733609684	-1.74800083307902
C	-3.17667034519924	-8.08327922468473	1.35358326953402
C	-2.34268747018541	-9.08515802152851	1.82858369943990
C	-1.16712946930048	-9.39499943930838	1.15947399414113
C	-0.83081129535656	-8.70073471169083	0.00598661605875
C	-1.66777558547645	-7.70392905908659	-0.47201806023294
H	-4.09727830215195	-7.84424410746166	1.86998000799266
H	-2.61416246584086	-9.62851430682228	2.72665094356852
H	-1.40735515625570	-7.17134654608827	-1.38105708421933
H	-0.51781924203830	-10.17877198074063	1.53225770665491
H	0.08130715037292	-8.94132976222843	-0.52805632907416
C	-5.40018543093432	2.19086613961773	-0.45833350253956
O	-7.99509775655418	1.29539188422673	-0.10466776101207
C	-7.80678885712949	2.47384320429747	-0.91046089546210
C	-6.40733744098378	3.00442480566304	-0.76580832287046
H	-6.24869027971637	4.05957632316684	-0.95827434400364
H	-4.37992809039821	2.54483671249167	-0.38512200213518
C	-8.83001130145623	3.46047654723508	-0.38294914305053
H	-8.62255403008990	3.69500283854198	0.66308272744744
H	-9.83347353649928	3.03484135139094	-0.45287216077509
H	-8.80036819027781	4.38571183460504	-0.96409007677490
C	-8.09081827119026	2.09940141404229	-2.36115070951231
H	-7.38542370199279	1.33688056501712	-2.70023987596279
H	-7.98476764958718	2.97313168585567	-3.00972859661528
H	-9.10701703372795	1.70722863875173	-2.45892201458099
C	-7.69404965873490	-7.33340305615784	-0.29101380715797
C	-8.99788712409078	-7.59890112679071	-0.29534904745572
C	-10.00679805926227	-6.49557200726544	-0.45509129200398
O	-9.44964920265346	-5.39082470627390	-1.19201076138715
C	-10.44948811687525	-5.97338417349866	0.90746666143645
H	-9.59015122878533	-5.59047286845335	1.46324927340738
H	-10.90483026337424	-6.77531024776036	1.49490159071827
H	-11.17777540973426	-5.16673066565929	0.78578887691469
C	-11.19155422212460	-6.94589910468843	-1.28735739643088
H	-10.85377502994327	-7.29411790172771	-2.26563065578400
H	-11.88617773221237	-6.11559360678700	-1.43248387750129
H	-11.71958840300626	-7.76130036616044	-0.78627250784074
C	-2.51940510305732	-1.98512826123610	0.03543206360014
C	-2.43329624942261	-0.53619641845630	0.48377399138252
H	-2.09169065026578	-2.07480486239433	-0.97120161524122
H	-1.88378349660762	-2.58526422790930	0.69385733195486
C	-1.07610413030811	0.05892278669905	0.24791237470660
H	-2.67601637282435	-0.48213654602737	1.55171728760127
C	-0.78250018490456	0.72379596035736	-0.93739186646182
C	-0.07982226972307	-0.09529385816181	1.20487782621939

C	1.19644292496052	0.39893603696446	0.97990604513226
C	0.49196467526621	1.22384773977537	-1.16003607973572
C	1.48529059848715	1.06132840119214	-0.20456674534161
H	-1.56157426874068	0.85610721443041	-1.67690193800950
H	0.70988265965529	1.74577888299359	-2.08496292052651
H	2.48021163782346	1.45400425971160	-0.38019090129185
H	1.96408632912836	0.27374550639584	1.73498636632567
H	-0.30494357738320	-0.60164612374688	2.13800306547284
H	-6.95293968151275	-8.10790336351081	-0.14052639330534
H	-9.37453594734590	-8.60459822728950	-0.14614962854502

Cartesian nuclear coordinates of $(S_1)_{(M)\text{-}(Z)\text{-}\mathbf{2}}$ (**ω**B97X-D4/cc-pVDZ + C-PCM CH_2Cl_2)

C	-1.17773012037443	2.49992310272819	2.64198619765991
C	-0.76383069328389	3.33224403832601	1.56485237857747
C	-0.46418241625341	2.74360771950559	0.35517683445599
C	-1.31843455267203	1.14496488384946	2.49589577827682
C	-0.98251511684979	0.51887353710561	1.27773262686130
C	-0.51933800265338	1.34395203753205	0.16673104675537
H	-1.70789629191021	0.56706922043032	3.32377008661185
H	-1.53824375991799	-0.37343685416720	1.01433808386325
O	-0.07986829292307	3.56844354686415	-0.64507041919434
C	0.72805779119710	3.01837398261289	-1.67560716758369
C	-0.05538178354479	0.77455162689752	-1.02761400460634
C	0.13113326344783	1.71268950974330	-2.18263460098609
H	-0.82182771899159	1.93834516280127	-2.67709144587953
H	0.79840247560542	1.29809697032610	-2.93961480437324
C	0.23047826572650	-0.59775184700196	-1.11372273777092
O	-1.42249969856341	3.04490976456781	3.84635205127970
C	-0.62350434086460	4.75075184669256	1.81671913087563
C	-1.84613604747534	4.41882889471866	3.93203273668985
C	-1.10033086016851	5.27216268226961	2.94436035534511
H	-1.00444866229882	6.32895417669133	3.16739395698420
H	-0.12166053312624	5.35687854803696	1.07363365502161
C	0.85756247558058	4.04895645629674	-2.75961935545791
H	1.71823774183199	2.80781684139210	-1.25377494768340
C	-0.16020499008707	4.96319047865998	-3.00728625425639
C	1.98887381187282	4.05668890071139	-3.56773084063389
C	2.10071453402682	4.95806339661480	-4.61563956912664
C	-0.04662702160588	5.86833665124534	-4.05236570828370
C	1.08123028490125	5.86719827265664	-4.86092576761460
H	-0.84361035788313	6.58032114671146	-4.23493866485610
H	1.16850929192668	6.57621802556774	-5.67621245622512
H	2.79265092623042	3.35389148109829	-3.37273331677306
H	2.98920843018108	4.95569306023794	-5.23666420156580
H	-1.03437738395934	4.96771289991219	-2.36929751548401
C	0.28554100502458	-1.33239412208154	-2.42000308204686
C	0.47420547159632	-1.35993316979549	0.03723766318117
C	-0.36881592480268	-2.69772632351709	-2.25891880727920
H	1.31998363425192	-1.48299794000107	-2.75299222675391
H	-0.23428530965778	-0.79303398388950	-3.21327565875154
O	0.23183677066626	-3.41762375150853	-1.19134047884936
C	0.41085864750500	-2.77064617117068	-0.01794720601075
C	0.71149206301619	-0.73233607923548	1.33325949756228
C	0.49490019865166	-3.55197579618443	1.11439521880304
C	0.68900971039086	-2.91122743775141	2.36966058128895
C	0.82939988960672	-1.55223051757110	2.47437713837121
H	1.29115530376240	0.18321243228266	1.32405124392730
H	1.05060641483646	-1.12229105084072	3.44248199660431
O	0.71903105822725	-3.64723444579210	3.49396473494248
C	0.33575333258977	-4.99061029047078	1.10209036725546
C	0.60184667129519	-5.69484432669720	2.19922669023712
C	1.13506515383500	-5.02480616224312	3.43460260153537
C	2.65948918527139	-5.05224828969334	3.44264647529955
C	0.56184527478797	-5.64796595915404	4.69260463684777
H	-0.00704655239521	-5.46111994993788	0.18973079395620
H	0.48478085448197	-6.77252815672449	2.22560294279353
H	-0.52883309382777	-5.59783267682044	4.67437630555298
H	0.92119946126426	-5.11356347545063	5.57474682988693
H	0.86498214574060	-6.69531357318799	4.76938844069634
C	-1.53408323559296	4.82312079474475	5.35981397693816

C	-3.34479194841921	4.46352346674209	3.65505521840128
H	-0.45915204744793	4.76281054825408	5.54241748121387
H	-2.04473699975437	4.15753903385768	6.05927655025941
H	-1.86591613160126	5.84789953351384	5.54536752954334
H	-3.55372983382007	4.10066858422116	2.64585109600922
H	-3.71830741037637	5.48816248881339	3.73275162238341
H	-3.88094123460733	3.83640032781129	4.37282744597346
H	3.05100988618942	-4.53267342292601	2.56485171413782
H	3.02418854362774	-6.08275016554770	3.41936893967906
H	3.04240491640338	-4.56198881361441	4.34200710702461
C	-0.25227966125177	-3.53605637940024	-3.49905518225490
H	-1.42788886645376	-2.55107648261802	-2.01571042117235
C	-1.22926479680402	-3.45248172946620	-4.48394725191678
C	0.85247499002422	-4.35483654453873	-3.70622678725543
C	-1.10233638976548	-4.16805911674796	-5.66543202292455
C	0.00327513187332	-4.98109057603220	-5.86917138306903
C	0.97778228896701	-5.07399269479136	-4.88521881315601
H	1.84051903215300	-5.71318928314429	-5.03547681011729
H	1.60534330610195	-4.43382599826090	-2.93251420528068
H	-2.10003100829224	-2.82465255902511	-4.32448202160062
H	-1.87229203024732	-4.09575429575085	-6.42514627941052
H	0.10233948585416	-5.54494266029274	-6.78960534335917

Cartesian nuclear coordinates of $(S_0)_{(M)\text{-}(E)}$à$_{(P)\text{-}(E)\text{-TS1}}$ (ωB97X-D4/cc-pVDZ + C-PCM CH_2Cl_2)

C	-2.89601736063573	3.32996643300009	2.09883669569968
C	-1.59240302912601	3.70166441378566	1.74590690264654
C	-0.68101734814396	2.70074190272908	1.37747593107736
C	-3.25237076900675	1.98689372620747	2.15517693182929
C	-2.33753833625005	1.02320587905374	1.77195543537587
C	-1.03790278429588	1.32689059177576	1.30769072023959
H	-4.24480290127637	1.71201382766240	2.49586875140568
H	-2.65575892958980	-0.00397358419732	1.84336932412865
O	0.57544292879019	3.16524946439093	1.12476513854272
C	1.62012885835625	2.18046321765212	1.18478504508411
C	-0.05972876466782	0.34832945091300	0.81324473937357
C	1.21284301922423	1.00574751092597	0.31645490942142
H	1.01148746740751	1.41164088179109	-0.68880673429611
H	2.04361262923265	0.31329867952149	0.20699206173487
C	-0.25228148654591	-0.99248442844024	0.61827731570668
O	-3.80231709081198	4.25247740781113	2.50771741284359
C	-1.22414543698023	5.09634050608739	1.89929187760073
C	-3.63395123561207	5.62475157297785	2.02672268977129
C	-2.17947970701686	6.01503071455708	2.07700600402568
H	-1.94237686332942	7.06449554553056	2.23045590127531
H	-0.17245378849575	5.36174766756908	1.89663118704455
C	2.90049496862496	2.82095994369346	0.72570784260536
H	1.72228832289091	1.85147153541203	2.23311982640913
C	4.11368465034228	2.37458460832942	1.25037518859379
C	2.90508842766787	3.80223970209510	-0.26562205978667
C	4.10952615603177	4.32881109542490	-0.72173793826697
C	5.31712328315271	2.89550359524397	0.78820125774709
C	5.31751931401344	3.87660578385523	-0.19928725619976
H	6.25533093437856	2.54189613286686	1.20594771654126
H	6.25611350115926	4.29002265011527	-0.55675023248726
H	1.96236833432859	4.15832057835997	-0.66922504932882
H	4.10382270969545	5.09752278685562	-1.48933838038542
H	4.11516690586152	1.61466922903827	2.02916633854542
C	-1.61955295843483	-1.64719178446685	0.55173084710021
C	0.85168877152424	-1.89682627073576	0.26167720871340
C	-1.58818912016502	-3.01044360457176	-0.15742297208388
H	-2.31821465029373	-0.98888566052319	0.02040747038741
H	-2.04480625548713	-1.83434285898862	1.54839391125584
O	-0.63242386915312	-3.00945842183229	-1.24448692311375
C	0.63228902476681	-2.85137056640162	-0.74227290213634
C	2.11411389394040	-1.89848893941843	0.87433005470146
C	1.64583496980107	-3.67848116165365	-1.23212035535892
C	2.92172409203791	-3.56447714467311	-0.65300374920481
C	3.14358733619783	-2.71344400279128	0.42746778272279
H	2.28408427265694	-1.24113773652881	1.72281854928337
H	4.11793971369332	-2.70710249587146	0.90452541890225

O	3.93622383783652	-4.37741578382882	-1.04719838195935
C	1.43348459870616	-4.71835204485240	-2.21903502444995
C	2.48942747264288	-5.33684646023103	-2.75765659781148
C	3.88999219386862	-4.89391473313752	-2.41591839804378
C	4.34508624819446	-3.77872766271635	-3.35993121710260
C	4.86596831674198	-6.06118350957231	-2.41882763491412
H	0.41430687699142	-4.98650457321428	-2.47849856354695
H	2.37589563423928	-6.14233690712158	-3.47843801065344
H	4.52909859445498	-6.84185711149176	-1.73100238880527
H	5.85995389168431	-5.72361162820673	-2.11141887414615
H	4.93578661623133	-6.48428950409809	-3.42565091811507
C	-4.47719870910066	6.46935996053061	2.97049363814370
C	-4.16247221316582	5.69172522541904	0.59193616475171
H	-4.09390885210224	6.39812698094141	3.99229333149754
H	-5.51623378744921	6.12761478007875	2.95779525908730
H	-4.44965772597200	7.51715154470059	2.65580957451251
H	-3.58196141050392	5.02892289299503	-0.05710666056233
H	-4.07166126299084	6.71293962424321	0.20773491821361
H	-5.21564558887594	5.39260455093459	0.56034757173978
H	3.66554312521658	-2.92387963558774	-3.28883750917565
H	4.33740478325301	-4.13712035449740	-4.39440741653196
H	5.35893127128780	-3.45302830746161	-3.10364124195595
C	-2.93953613713661	-3.37393069312632	-0.70601839305674
H	-1.26150796537085	-3.78327866320916	0.55603806620285
C	-3.35903855597198	-2.89347405768207	-1.94748108742685
C	-3.80945436408653	-4.15277826819209	0.05540299679491
C	-4.63207338941321	-3.19426852476078	-2.41834849262391
C	-5.50113537542257	-3.96611116822821	-1.65114482388428
C	-5.08786174246769	-4.44353868279338	-0.41167808535353
H	-5.75714718904968	-5.05240007000925	0.18921773467843
H	-3.48302153723403	-4.53971183472454	1.01826477193758
H	-2.67945299887357	-2.29335917830647	-2.54504922423718
H	-4.94806144588399	-2.82386722534349	-3.38942932634712
H	-6.49564501073626	-4.19810735158740	-2.02103359254351

Cartesian nuclear coordinates of $(S_0)_{(M)\text{-}(E)}$à$_{(P)\text{-}(E)\text{-}I}$ (ωB97X-D4/cc-pVDZ + C-PCM CH_2Cl_2)

C	-2.58839288496921	3.19705998629153	2.39506878141133
C	-1.43573021810065	3.50416916384266	1.65441343028562
C	-0.50771354723295	2.48464515664390	1.42637142867053
C	-2.77246173525674	1.92177080490569	2.93284815610313
C	-1.83018575056124	0.92745747772776	2.67931799327348
C	-0.69636612826197	1.17342624813867	1.89146126047443
H	-3.65530543202512	1.72422728132476	3.54366234502226
H	-1.98033436570149	-0.06228448402687	3.11153841538779
O	0.63813653175948	2.79758627257565	0.74260003225911
C	1.78638502983881	2.22382392630938	1.40083648411952
C	0.30724068459586	0.16105211789353	1.47763696600333
C	1.72939676564614	0.69125770660392	1.36556801257012
H	2.18038359560245	0.32554899517436	0.43035969386822
H	2.36676628980523	0.33262927392872	2.18837619171652
C	-0.04000089625911	-1.07981023892986	1.05689392303347
O	-3.50441640486488	4.15985059656966	2.68513601411142
C	-1.23574605339434	4.89427107837759	1.23378506086048
C	-3.63128725958536	5.26990178395269	1.74905619885345
C	-2.26196364501745	5.74865314816309	1.31174528905860
H	-2.16033814846103	6.79948708803490	1.02687154284909
H	-0.24735434210651	5.19991608641745	0.88605781016095
C	3.02903449892737	2.75368789085064	0.72700476430057
H	1.76528176691273	2.56811783479008	2.44908826922728
C	4.17845567494880	3.00581128118877	1.48102515618595
C	3.05890336042033	2.95047699595477	-0.65839982461125
C	4.22529868442504	3.39926230877078	-1.27892418784257
C	5.34944608794458	3.44605349421974	0.85913646336040
C	5.37475397294758	3.64567961663618	-0.52250001142910
H	6.24268608252388	3.64123620562086	1.45837452635095
H	6.28809404579316	3.99633222440173	-1.01011042295557
H	2.15975374561650	2.75845511923559	-1.24802213922221
H	4.23811473912186	3.55726439892636	-2.36070303069220
H	4.15638015494909	2.86012082826219	2.56503019988354
C	-1.49192705882193	-1.50426643379043	0.88800429537476

C	0.95179793144655	-2.05483271414944	0.53828226142204
C	-1.64369080642894	-2.71131741753389	-0.04570679001209
H	-2.07399450059163	-0.65660528721108	0.49471705657750
H	-1.95149340692767	-1.78840524683473	1.84695604379114
O	-0.67480300775759	-2.66118815400632	-1.11323876090592
C	0.59425221633436	-2.78042662382098	-0.60949343207387
C	2.22438867760929	-2.29192969651291	1.07798246254565
C	1.47743532415797	-3.65019682120181	-1.25463184471726
C	2.76358854487312	-3.80962450394430	-0.71421297128480
C	3.12804303424593	-3.15698902459415	0.46497878299324
H	2.51737082193346	-1.79438619951808	2.00285285957626
H	4.11867976471336	-3.33498905972223	0.88742708052383
O	3.64974318854577	-4.67626497440454	-1.27439728492221
C	1.11098011982247	-4.46652419000734	-2.41592584010178
C	2.07428738895633	-5.10739322555321	-3.08716510023520
C	3.52937411757686	-4.92796316496749	-2.70511130118125
C	4.13504945731128	-3.73913681966922	-3.45506083825076
C	4.32672990910829	-6.20502679677326	-2.93741151144540
H	0.05751898249880	-4.54517210565826	-2.69022550482756
H	1.85192227520498	-5.74224159814486	-3.94928513364162
H	3.88115812434748	-7.04032319082282	-2.37636477611710
H	5.36722390700792	-6.06753725279545	-2.60674724458431
H	4.32705340748700	-6.45797247045382	-4.00839244136758
C	-4.37339541687264	6.34669828928113	2.53051005345221
C	-4.43996110496926	4.79030311859958	0.54134909163490
H	-3.78072535002544	6.66206027146462	3.40245922562767
H	-5.34355962086294	5.96129290774779	2.87851272280135
H	-4.55090805044523	7.22197902585855	1.88759319761808
H	-3.91811245122299	3.96608463415911	0.03064499285347
H	-4.56905881728193	5.61474453689427	-0.17707002579594
H	-5.43364431698614	4.44059990931965	0.86326594540685
H	3.58500150533918	-2.81483185533321	-3.21953859023528
H	4.07512858238199	-3.90930691934216	-4.54128440692506
H	5.19183777899858	-3.60925434864100	-3.17294969956537
C	-3.02071034342676	-2.77377035791201	-0.66097508528489
H	-1.45550958126325	-3.63968735743530	0.52040103745768
C	-3.31724095228922	-2.04546581259817	-1.81882792733956
C	-4.02614609743344	-3.52482622726905	-0.04604599673956
C	-4.60480662225396	-2.07399054576056	-2.35574273049142
C	-5.60958743547609	-2.82205584834753	-1.73522773163125
C	-5.31793471606143	-3.54626184013897	-0.57766635163951
H	-6.09699153311842	-4.13757329593671	-0.08932787135484
H	-3.79561169258222	-4.10109136439955	0.85483562247633
H	-2.53089296313895	-1.46198075015736	-2.30284619812566
H	-4.82657725466321	-1.50770511270787	-3.26431776828032
H	-6.61819685898047	-2.84238975403153	-2.15618636570420

Cartesian nuclear coordinates of (S_0)$_{(M)\text{-}(E)}$à$_{(P)\text{-}(E)}$ -TS2 (**ω**B97X-D4/cc-pVDZ + C-PCM CH_2Cl_2)

C	-2.92557914760475	3.24137781013652	2.16984458676799
C	-1.72639142583519	3.52166993117764	1.50853647657588
C	-0.90327282288149	2.45290693130960	1.11080305250567
C	-3.29704770825245	1.92594321561085	2.42585674099713
C	-2.47574850181483	0.89617089374955	2.00756006883153
C	-1.26778392982868	1.09364878781072	1.30791554265106
H	-4.22160778973172	1.72580938461166	2.95655740319719
H	-2.79696958622660	-0.10292191290074	2.25107833847439
O	0.26396950015642	2.86143251545151	0.53760169676263
C	1.28163459477346	1.86766764273975	0.35304952708534
C	-0.37970563368005	0.03796365942100	0.78682794651490
C	0.63261078826053	0.60611080776150	-0.18214462529212
H	0.09146474201051	0.88464570525397	-1.10186731501348
H	1.39122108022488	-0.11705860635098	-0.47340845774612
C	-0.41944190412403	-1.31241000990012	0.97024693817283
O	-3.78010671918713	4.23307965774010	2.53073415380489
C	-1.42313757833303	4.90992510272722	1.21480738635050
C	-3.21061666058346	5.55402695001920	2.79818339565156
C	-2.14720384965005	5.88051270114211	1.78211343659243
H	-1.96602336185883	6.92963413348207	1.56327549244056
H	-0.62442360282451	5.13602581308445	0.51694251624892
C	2.32344869180569	2.41296604734477	-0.58384055282210

H	1.73890091313203	1.66084188588296	1.33595709271660
C	1.99083631593415	3.29862774457971	-1.60834521033004
C	3.63676203562819	1.95525059812794	-0.47628873822086
C	4.60622685197678	2.37149418123816	-1.38145190563782
C	2.96393116783699	3.72292953712633	-2.50821076452901
C	4.27198326390031	3.26036579978154	-2.39954635652143
H	2.69813994289634	4.41863831360956	-3.29914848111285
H	5.02911332018693	3.59307047915107	-3.10358959402712
H	3.89978400675723	1.26483832354145	0.32241317570443
H	5.62508024678353	2.00694598961714	-1.28749333371509
H	0.97005457670404	3.65821067832638	-1.69273557906314
C	-1.57905333626896	-2.17804466655438	1.39126171889799
C	0.67062107711567	-2.20901066282978	0.54267784732320
C	-1.99463994185635	-2.96311035786092	0.12958654971370
H	-2.46089899604556	-1.65277885326776	1.73873626997713
H	-1.27984683421479	-2.89338905120285	2.16870083765641
O	-0.93199060872336	-3.81181227396360	-0.33405831397861
C	0.35129851180326	-3.43683318190158	-0.06190786600386
C	2.02013052972027	-1.98048045933297	0.85201828012685
C	1.34101688035927	-4.36061027617600	-0.43311130935638
C	2.68073252847409	-4.04350606023469	-0.16377537050964
C	3.02298912169080	-2.86941337994172	0.50290223517832
H	2.27485840140551	-1.07611008581026	1.39835032322994
H	4.06249538271892	-2.67945674696713	0.74738692915834
O	3.67473967542000	-4.92421504430549	-0.45392012913089
C	1.05141119527982	-5.66184284261919	-1.00294126978589
C	2.04761267309782	-6.39984237606606	-1.50443789095729
C	3.45095796348012	-5.85301702798405	-1.56095553817896
C	3.68214764018595	-5.09562591755401	-2.87093589574045
C	4.48956489353974	-6.94693905600852	-1.36098643845744
H	0.02824530323288	-6.02197733954830	-0.98888656638726
H	1.87462873498257	-7.39174756480340	-1.91375464369587
H	4.31345009486664	-7.47370080720746	-0.41883297714362
H	5.49401560632255	-6.51424553321569	-1.34178657681914
H	4.43419808075930	-7.66713353141989	-2.18318142049427
C	-2.61525540480149	5.53444788575820	4.20790536897370
C	-4.39206820309007	6.50867745534830	2.70514072722621
H	-1.81012133885562	4.79542756819535	4.26698844192243
H	-3.38722654670371	5.28387028578227	4.94348307263043
H	-2.19692254030854	6.51640365082261	4.45216041850738
H	-4.81247612223561	6.49805367689878	1.69559349184871
H	-4.06713697402569	7.52672605793703	2.94114301153239
H	-5.17070564713500	6.21621059916587	3.41562802457673
H	2.97692798081133	-4.26286599929853	-2.95698396578364
H	3.52770568848866	-5.76473064297478	-3.72370588934368
H	4.70397107769841	-4.70288439389640	-2.90730831642900
C	-2.46257161643978	-2.02315928999701	-0.96170043237919
H	-2.80652980259755	-3.65586228756642	0.38992550076074
C	-1.69233947741609	-1.76903949934541	-2.09424117173995
C	-3.66562288034281	-1.33720317034303	-0.78932632105483
C	-2.11375166495219	-0.82978963147336	-3.03158252267463
C	-3.30672240528139	-0.13860871653830	-2.84765930246915
C	-4.08595274195188	-0.39878404137581	-1.72376618010110
H	-5.02073767853148	0.13295254056999	-1.57263094734657
H	-4.27810279783156	-1.53142048216716	0.08873305320747
H	-0.75965797593189	-2.30425671810484	-2.24139947887350
H	-1.50274501664754	-0.63679140062002	-3.90895722130005
H	-3.63074130581576	0.59712695759370	-3.57782420032848

Cartesian nuclear coordinates of $(S_0)_{(M)\text{-}(Z)}$à$_{(P)\text{-}(Z)\text{-TS}}$ (ωB97X-D4/cc-pVDZ + C-PCM CH_2Cl_2)

H	1.71039277913376	-8.28961119899800	-0.69357510429847
H	1.55681387631513	-7.97461578793132	1.06346580780447
H	4.28239011253612	-7.90504585285325	-0.39576356629288
H	4.02929386756063	-7.52077790353513	1.33278452092505
C	1.56883740910999	-7.50578926305008	0.06675954401069
C	4.05026018595818	-7.09904034879134	0.31660075152735
H	0.59641195197989	-7.02153938234089	-0.11329870789174
C	2.70050810923340	-6.47913692785276	-0.02189773502334
H	4.84370886858017	-6.33850453852103	0.26179988639632
H	3.29876254596377	-6.31926011381864	-2.17583163853985

C	2.71677998595929	-5.82973719781729	-1.39007809717078
O	2.48836473263453	-5.47460585348542	1.01223007409687
C	1.54168683023576	-4.52480841093994	0.77864219332353
H	-6.46946190698470	5.08394559507179	-1.66345742915222
C	1.99873945762147	-4.72644233651480	-1.62877660328751
H	1.08671632379538	-4.34218622051402	2.87670968274223
C	0.88165952978302	-3.96292965287444	1.87431532258029
C	1.25603211480244	-4.10503198997418	-0.52728554336266
C	-5.60795207882611	4.44839905883082	-1.44247106892175
H	-5.17817980462593	5.41462426530553	0.44357130704763
H	1.96505486078742	-4.26159261254199	-2.61542681842145
C	-4.88354432006934	4.63317708829816	-0.26208836420727
H	-5.78130104298685	3.30265596544230	-3.26910571620162
C	-0.03985933478841	-2.94422232890457	1.65530938182772
C	-5.22292514257967	3.45036726963223	-2.34090726318054
C	0.30348205424491	-3.09074020099031	-0.71372992699317
H	-0.60474649404185	-2.54784694165865	2.50131807668354
C	-0.30703166542971	-2.44119642941919	0.37313142211521
O	0.02141753020346	-2.77881400024158	-2.00839247673503
H	-2.52706960014687	-2.96133364840563	-0.94813430984972
C	-3.78210698401350	3.82309616318997	0.02148873391879
C	-4.11778559784834	2.64502913098543	-2.05944511117302
H	-1.62779126969520	-2.50020128842893	-3.14988477938101
H	-3.21336600403527	3.97003160303177	0.94225167546389
C	-2.14056748313141	-1.94489636253280	-1.12470524460643
C	-1.14774031491184	-1.98729309743302	-2.30334935799185
C	-1.35666787387269	-1.43894137308990	0.06764217633117
C	-3.39289832429969	2.82391614624858	-0.87653649442925
H	-3.81121997373543	1.86906780380804	-2.76648220719234
H	-2.97849411331695	-1.30882076138019	-1.42964675107818
C	-1.48388572247853	-0.18583495063496	0.56480656111613
H	0.46535225694945	-0.90327324008436	2.51889794365989
C	-0.71685457074336	-0.60355266680845	-2.74679205608890
C	0.47578555810460	0.14230520328491	2.23331185730713
C	-0.46586956650382	0.61441816927209	1.30338448833001
H	0.96813690685346	-0.47056850643896	-1.39568420996871
C	-2.24158299818397	1.89461082867228	-0.56690410940997
C	1.42512261492507	0.96149494078715	2.83213187310687
H	-3.13671546277825	1.02990183197708	1.17909404403429
C	-2.68152688614482	0.67856237191729	0.23650882935794
H	-2.30416177355435	-0.43669529964545	-4.20161440240440
H	2.13765606831638	0.56306712937297	3.55642411026382
C	-0.41405666447963	1.99889763706266	1.01514417771475
C	0.37983950003392	0.03918760843203	-2.16093199884740
O	-1.25566659625169	2.64246983809114	0.15739949387328
C	-1.45162446243159	0.06369471550589	-3.73201779920500
C	1.46162181957342	2.31976207030657	2.50951333285990
C	0.54210248890103	2.84859826243975	1.59974862171809
H	-1.78717845252296	1.55920534824411	-1.50883057100121
O	2.40950258115015	3.07336144808439	3.11888833119712
H	1.36943017050185	5.01360603236542	4.60661150687266
C	0.62418904619335	4.27995629550427	1.29114192298430
H	-0.08296875369787	4.70353574701732	0.57749432523087
H	-3.46059747976498	0.14804280488133	-0.32026930194923
C	0.72306044500160	1.33703101180510	-2.54280716708044
C	-1.10718609068540	1.36129108611185	-4.11880973192342
C	1.55579589672041	5.03725376978271	1.87677179289628
C	2.38677367476541	5.18668293140333	4.22392583030347
C	2.56168253515619	4.50325628210451	2.86702282519953
H	3.11396181634184	4.78392609658822	4.94604766648965
H	1.57360803101569	1.83331430000916	-2.06817156357147
C	-0.01992321551226	2.00293157875972	-3.52109236758743
H	1.63554812274901	6.10613137053273	1.65876939272611
H	-1.69033347679682	1.87177808623329	-4.88973719294333
H	2.54832332146175	6.27170915754485	4.12816491726324
H	0.24895537598280	3.01987566646423	-3.81823098199822
C	3.98248366162537	4.68384556620976	2.33024609641165
H	4.09596501346130	4.15294330463462	1.37281237029925
H	4.71187180832297	4.28203004287304	3.05065220359864
H	4.19675965732436	5.75189806733129	2.16922869775159

Cartesian nuclear coordinates of $(S_1/S_0)_{(P)}$ (ωB97X-D4/cc-pVDZ + C-PCM CH_2Cl_2)

C	2.64504735066876	2.51511813667098	0.68610768299349
C	1.46692884315449	3.22799524544118	1.07627623186191
C	0.28672229907496	2.72755813486907	0.58729633530252
C	2.65159673033731	1.35994676746586	-0.07831998468201
C	1.44368169877941	0.72770599947739	-0.43562199636378
C	0.19714302022822	1.51004824707957	-0.15166618569303
H	3.60969357208053	0.94507984148507	-0.35871414020859
H	1.41697609302660	0.19484783327563	-1.38585016235733
O	-0.82713183100399	3.41799449113235	0.84947870516311
C	-1.92778149138299	3.18031431392247	-0.01862704983211
C	-1.01448794776142	0.88053160843080	-0.28368348508881
C	-2.24325240074846	1.69908293323171	-0.05578178861962
H	-2.69921818947184	1.37597995474613	0.88785286371415
H	-2.98422636315981	1.50974796882879	-0.83519690667154
C	-1.11129257893516	-0.51352912083618	-0.54636937366105
O	3.82720604836608	3.02072709216856	0.97330909497958
C	1.62629175734943	4.53325183570046	1.67094307559605
C	4.00620297091867	4.04335784005392	2.00012798810186
C	2.81971786427285	4.95594348926961	2.07838492200597
H	2.97303912089703	5.94081828857388	2.50203556126411
H	0.75106155770865	5.16755271255903	1.74479311462354
C	-3.08905144135270	4.00447746652631	0.45543519945848
H	-1.63253662309469	3.50296324646925	-1.02299613431423
C	-3.23233756469691	4.34245982906373	1.79627711362818
C	-4.06607909540488	4.39452733863726	-0.45312082835217
C	-5.17878812407624	5.10510079311659	-0.02883367996287
C	-4.34215601528327	5.05903091920689	2.21945099062452
C	-5.31909399076743	5.43957805454099	1.31057873085357
H	-4.44262105281448	5.32346931536283	3.26601018397569
H	-6.18466491972825	6.00025781706303	1.64383412394645
H	-3.95365955199730	4.14604732862589	-1.50372299208012
H	-5.93288257727838	5.40563567773559	-0.74696433127676
H	-2.46531225318634	4.05209884247799	2.50225457660321
C	-2.31537023762744	-1.14603760919118	-1.18805388271315
C	-0.06127831459606	-1.33844778174567	-0.13591179996149
C	-2.37918176319209	-2.62635735436618	-0.84059716799551
H	-2.31207878625161	-1.05989235541109	-2.28337901942051
H	-3.25213489489029	-0.69183590527087	-0.84807563177511
O	-1.13687080667716	-3.24781091203974	-1.14414319066923
C	-0.05830695949481	-2.71355577415235	-0.51504607999783
C	1.08715181730778	-0.87629666524466	0.60301133786463
C	1.04624986912485	-3.50297250160696	-0.34105160540585
C	2.21835894431767	-2.99016189358737	0.28907983178772
C	2.20689658974550	-1.73493716488829	0.79652463759094
H	0.88224976185360	-0.19475415683921	1.42030161584310
H	3.06967372537895	-1.37010636751789	1.34163107772437
O	3.29463244720427	-3.80760636470320	0.46512926485620
C	1.09326506747811	-4.87178641441790	-0.83187500168750
C	2.27031372748050	-5.48254225858891	-0.92607887060425
C	3.55046446014000	-4.78835624101861	-0.53976524889190
C	4.17118022575633	-4.11426822827115	-1.75909847653398
C	4.51705846958993	-5.76165720165235	0.10806002063694
H	0.16865174642460	-5.34404548028655	-1.13465531984132
H	2.34694380875123	-6.49086331986708	-1.31991297880899
H	4.05480549306860	-6.22479251029569	0.98227572475250
H	5.41544178198705	-5.23166413839828	0.43093819186257
H	4.80594597774751	-6.54491172630326	-0.59742656086636
C	5.26178460114915	4.77765433869646	1.57383922937544
C	4.21544144399652	3.28965386517939	3.30753302425436
H	5.10275113083134	5.26770945637141	0.61122618802162
H	6.10035897742643	4.08335415783584	1.48227630936871
H	5.51770974348836	5.53942546461009	2.31480697768699
H	3.31374273006713	2.72957099638137	3.56618881544233
H	4.42018304082463	3.99544265171361	4.11671729113170
H	5.05514885538690	2.59353665529850	3.22385102620710
H	3.49586878873277	-3.35401216243104	-2.15874827700233
H	4.36873843219385	-4.84406611424931	-2.54889259456292
H	5.11274512407760	-3.63391572072850	-1.48019913020614

C	-3.45650020370230	-3.34978362684525	-1.59384245694492
H	-2.55749265259875	-2.72853593875742	0.23697585464715
C	-4.74914607966275	-3.38119540236329	-1.08320916332560
C	-3.19994433508149	-3.94435932629301	-2.82427267705600
C	-5.77437018363691	-3.98978382137282	-1.79160844318595
C	-5.51340292768583	-4.58127731903787	-3.01948023053029
C	-4.22364234771375	-4.55836025526381	-3.53095600013449
H	-4.01242871109790	-5.02245302671710	-4.48787545886354
H	-2.19221041515192	-3.92954233873014	-3.21857359186282
H	-4.95522058804017	-2.92829150814332	-0.11874247699934
H	-6.77737578678319	-4.00870600788804	-1.38047725620631
H	-6.31153470236519	-5.06212093397399	-3.57339728253309

Cartesian nuclear coordinates of $(S_1/S_0)_{(M)}$ (***ω***B97X-D4/cc-pVDZ + C-PCM CH_2Cl_2)

C	-1.12503020526114	2.47908643340987	2.65622204926772
C	-0.79096046968049	3.28639727736389	1.52942849003548
C	-0.46851032847947	2.71806519779982	0.32571363046915
C	-1.16399648075567	1.13194332244294	2.52262150720471
C	-0.77882489459202	0.50638417540224	1.30354505248150
C	-0.36589560959747	1.30321781496574	0.17355174514165
H	-1.46112164664691	0.51756170026034	3.36476997261200
H	-1.33098306853440	-0.39636407587133	1.06410675957980
O	-0.21141154363738	3.54048065641087	-0.72442155603711
C	0.78618845463261	3.08529272749343	-1.63481116240867
C	0.17398302172023	0.77508049882099	-1.00254954768433
C	0.41499221085214	1.70549067781907	-2.15773289146678
H	-0.45994128330814	1.82229813557509	-2.81308794898057
H	1.23412726772319	1.35590033978077	-2.79259607792259
C	0.35317881509296	-0.63096405014337	-1.09352930349827
O	-1.33245577754539	3.06799613173068	3.86727051604275
C	-0.79957846276158	4.72892323494297	1.71492367506115
C	-1.93056826760366	4.36278858786339	3.89032630731825
C	-1.33172374880342	5.23861804449911	2.82194644977382
H	-1.38313534705955	6.31208492035056	2.97287067327219
H	-0.39542242490962	5.35144821305221	0.92825449055028
C	0.90534560709675	4.11058637366856	-2.72386301980078
H	1.73740599740000	3.01219116181707	-1.09451041250075
C	-0.19612175057191	4.85657003221298	-3.12901727736228
C	2.11993364965286	4.28726717575429	-3.37676519408872
C	2.23357942545573	5.18850983942195	-4.42495744868131
C	-0.08175304260043	5.76240398733696	-4.17347014574035
C	1.13121189137492	5.92922066932053	-4.82611323794946
H	-0.94522198400024	6.34286078110678	-4.47857742070688
H	1.21842518827554	6.63805797385684	-5.64143462844889
H	2.98699096198856	3.71684525203820	-3.05902385874637
H	3.18736597320371	5.31790354639415	-4.92408947446123
H	-1.13865824281782	4.73029158668372	-2.61176374701755
C	0.33781141965482	-1.35327198232354	-2.40493581551348
C	0.51012770627399	-1.39941156678071	0.02567154110428
C	-0.41201958079494	-2.66630211847255	-2.24521347440456
H	1.36612154847450	-1.55405927392977	-2.72445715456996
H	-0.13955314601414	-0.76351902893703	-3.18828121145558
O	0.10655018080221	-3.43260389310892	-1.16688255782738
C	0.38024106600890	-2.81429789494966	-0.00955616527858
C	0.74623042516441	-0.69833546331140	1.32851576973225
C	0.49616156210129	-3.59999111606616	1.10431657250597
C	0.74820961130233	-2.90568867471000	2.33124803110948
C	0.85989426405166	-1.53066040149574	2.45901768517891
H	1.44988500442321	0.13243092694759	1.24852434651447
H	1.16063038645830	-1.13797923727791	3.42025972175258
O	0.90087305835384	-3.59817323819371	3.43871253116605
C	0.24537006813815	-5.01907826968486	1.16177930603165
C	0.51317910395569	-5.70708492958177	2.26821338171183
C	1.19139617831137	-5.02752676530578	3.42079028743290
C	2.70664536496287	-5.14993236440300	3.32193165013315
C	0.67809933171525	-5.52386057150857	4.75705728863359
H	-0.19915286062347	-5.49460006687251	0.29533292047155
H	0.29367570891313	-6.76320673191173	2.36541272082019
H	-0.40229060103574	-5.38118149221107	4.82052070409777
H	1.15512128229321	-4.98301469127958	5.57783717079288

H	0.89705276354255	-6.58906901606397	4.86669277323756
C	-1.61516839457708	4.90726743653102	5.27098208184332
C	-3.43454058444234	4.22713845313729	3.67606684889558
H	-0.53466994304061	4.99179944806266	5.40452976776635
H	-2.00663822305207	4.23115109994867	6.03393133637897
H	-2.06775516671323	5.89222931928530	5.40929189857368
H	-3.64224451433728	3.75545383881922	2.71239589238771
H	-3.91914473768338	5.20732116196248	3.68737940484769
H	-3.86971086921671	3.61206252820569	4.46782871954260
H	3.05596055508947	-4.73589826580921	2.37298071734306
H	2.99978473563677	-6.20216495991618	3.36329527028561
H	3.19164516648029	-4.61472796468427	4.14363274450814
C	-0.35584623041020	-3.51156479951519	-3.48622577699543
H	-1.45967960887103	-2.43858200440909	-2.01132479863332
C	-1.23252737515251	-3.24566665859612	-4.53210482281845
C	0.58729869778526	-4.52202722023924	-3.62983919661315
C	-1.16293040602901	-3.97015438051100	-5.71207394097092
C	-0.21720906514103	-4.97607554635175	-5.85325183835690
C	0.65343311467779	-5.25093407119903	-4.80888924063533
H	1.38904863477340	-6.04084054287879	-4.90993329892222
H	1.25906020563142	-4.74055590520768	-2.81048004577486
H	-1.98172874315685	-2.46803065793592	-4.42244806826336
H	-1.85352088240077	-3.75389989262720	-6.51876318959919
H	-0.16459109758617	-5.54732089822067	-6.77272745347464

Cartesian nuclear coordinates of $(S_1)_{R,R}$-**2'** (***ω***B97X-D4/cc-pVDZ + C-PCM CH_2Cl_2)

C	0.98859356336857	-0.28052207419867	0.11179420098853
C	-0.25888651746556	0.25385658042239	0.52937678423768
C	-1.41484967445160	-0.55990011515117	0.34192558470068
C	1.09069441589845	-1.49071389700687	-0.49226126071302
C	-0.08428171601225	-2.35490898888560	-0.77401571598600
C	-1.36398738276058	-1.82392871212138	-0.19638190468272
H	2.06726101310106	-1.79726971606328	-0.84290314568996
H	-0.20942126431018	-2.43045418973708	-1.86619063585569
O	-2.58026116118039	-0.01775466386795	0.76423513879577
C	-3.74096957818001	-0.51848148968625	0.11665550075332
C	-2.51323508533305	-2.66014759043620	-0.18437930668718
C	-3.80389157604935	-2.03181679257720	0.25226364414007
H	-4.01776005295621	-2.28079949288148	1.29997182841246
H	-4.65173301696084	-2.39620824893873	-0.33321631081594
C	-2.41056335262605	-4.00282312591803	-0.52048217026623
O	2.10136798013020	0.48053188420468	0.22217462894005
C	-0.29870849773629	1.56828575992863	1.06266741728648
C	2.16344828545074	1.47080406236681	1.26201037403655
C	0.85167664521435	2.19406534060691	1.38180605765387
H	0.85889673696456	3.19956978897255	1.78583343981365
H	-1.26131956911601	2.04120602381553	1.20890626031288
C	-4.93904661454962	0.16380009755392	0.70874743767447
H	-3.66293398958493	-0.26539763645460	-0.94785303784472
C	-4.94323170945713	0.57892477403617	2.03573201561242
C	-6.08222428281619	0.34284624001699	-0.06163391437781
C	-7.21807763118582	0.92053248267647	0.48552471019296
C	-6.07698837685655	1.16222569700286	2.58176918006620
C	-7.21795192276522	1.33278493231739	1.81047274115168
H	-6.06805080354344	1.48777169541873	3.61594149396235
H	-8.10257894297619	1.79012074677046	2.23857006574521
H	-6.08310495291110	0.03254502878020	-1.10169064321061
H	-8.10223900161304	1.05649864010647	-0.12683917230703
H	-4.04898295838610	0.45266597371996	2.63233215631051
C	-3.61011443853118	-4.86911514900474	-0.76882134248362
C	-1.13578563110694	-4.60825757764097	-0.69072835446068
C	-3.24316563753298	-6.34062644211952	-0.65965923792074
H	-4.01776099407308	-4.68420421171283	-1.77131127497634
H	-4.41642355965608	-4.65976128545203	-0.06145829783494
O	-2.12017254267829	-6.62524500153475	-1.48066873217500
C	-1.02777992620533	-5.86751261306558	-1.23174694429807
C	0.09269900174624	-3.83856603940235	-0.30262575866083
C	0.21914251058975	-6.45259323652918	-1.60151209800410
C	1.39212004430564	-5.68785663361453	-1.36668024033014
C	1.35403249451929	-4.47144410060932	-0.76774339936844

H	0.11887292757681	-3.77199348623657	0.79696926408682
H	2.29693787014936	-3.98201357280380	-0.56310050178605
O	2.59857831490576	-6.22919232960027	-1.65272451256197
C	0.34698677164939	-7.75685104383327	-2.14627221850431
C	1.53275309780897	-8.15642516277742	-2.64740784605840
C	2.68680948489450	-7.19608125190545	-2.71249153066607
C	2.68630156993451	-6.45082250170044	-4.04397986388825
C	4.00862682164544	-7.90009566186334	-2.47500994125501
H	-0.51998916198306	-8.40501669666750	-2.15461593260170
H	1.66862859528947	-9.14613269980242	-3.06735629589357
H	3.99698363743772	-8.40587497937905	-1.50733417737161
H	4.82599710472768	-7.17555472745012	-2.47994182183995
H	4.19213753483984	-8.64051108128610	-3.25790851373052
C	3.27585146681761	2.40607474000592	0.82901567668775
C	2.50697000134560	0.76859210301504	2.57207756463240
H	3.02151938393178	2.87836024790301	-0.12203531516903
H	4.20703177093917	1.84909356703620	0.70342088055859
H	3.43217618544962	3.18545044213419	1.57926498158272
H	1.72447470864209	0.05343990062428	2.83717957859294
H	2.59452361543124	1.49451955123037	3.38493057987594
H	3.45458281015285	0.23123859305976	2.47405305135895
H	1.75165104390005	-5.89920066101842	-4.17112227292612
H	2.78325724054615	-7.15284064921788	-4.87647680236444
H	3.51919761368439	-5.74282766980191	-4.07977315086353
C	-4.36576203279113	-7.24855187800664	-1.06800920914402
H	-2.95859196146821	-6.55428786455288	0.37806080241086
C	-5.33049609536173	-7.61162868939752	-0.13530505844089
C	-4.48455441902376	-7.69862399543647	-2.37810988702712
C	-6.40491440634558	-8.40683470969264	-0.50467255061783
C	-6.51967355216554	-8.85364531727944	-1.81348155497786
C	-5.55592810808386	-8.49906901405776	-2.74674573787672
H	-5.63676707576403	-8.85024143988482	-3.76929344322181
H	-3.72662006587840	-7.42769252561590	-3.10163472721801
H	-5.23750549146317	-7.27421764275834	0.89201329381373
H	-7.14875503388323	-8.68524197276040	0.23303496536284
H	-7.35491249720941	-9.48063664432844	-2.10357553479661

Cartesian nuclear coordinates of $(S_1)_{S,S\text{-}\mathbf{2'}}$ ($\boldsymbol{\omega}$B97X-D4/cc-pVDZ + C-PCM CH_2Cl_2)

C	0.88166809156837	-0.25381315266504	-0.20789728619084
C	-0.31553940626923	0.25883799554401	0.35865803024673
C	-1.43214551729502	-0.62387058625795	0.44854056417004
C	0.99724571875387	-1.54283855239220	-0.61482419636099
C	-0.09898575224852	-2.52834528191738	-0.46791933955177
C	-1.37808628529159	-1.93797738406259	0.04566411207184
H	1.94895006852213	-1.86666807495655	-1.01537880787214
H	0.22917027483540	-3.30353102273907	0.24232231859281
O	-2.56689178454062	-0.06345200476729	0.92780009481535
C	-3.78670830741109	-0.69741673758672	0.56944385634737
C	-2.53185143359831	-2.76608164197900	0.09837920248139
C	-3.70734204270273	-2.19699757654654	0.83020561155143
H	-3.61538841431356	-2.35311662475099	1.91260887723210
H	-4.64766642243145	-2.65349478623171	0.51733673720843
C	-2.52656113022736	-4.01209771092785	-0.51340865006486
O	1.92455920336119	0.58266669822142	-0.41564975317606
C	-0.37131721881395	1.63296560482486	0.71459348050618
C	2.08587659344746	1.71905603773177	0.44939124936736
C	0.75843338111865	2.36686924720402	0.72435424678777
H	0.74960189422353	3.41920909257366	0.98344000954429
H	-1.32935811415847	2.06375468914410	0.97544171436790
C	-4.89645812209011	-0.03810789308623	1.33412258582708
H	-3.94163316180638	-0.54062305613018	-0.50502035363541
C	-4.68424078880566	0.47768188218365	2.60785857440733
C	-6.17258090796234	0.01268636886281	0.78533104285050
C	-7.22648561695881	0.56311420094063	1.49906487466021
C	-5.73676417013501	1.03355406725454	3.32004861306013
C	-7.01035248258431	1.07566494366654	2.77036804818425
H	-5.56059481388711	1.43826202166697	4.31031981304588
H	-7.83093280044128	1.51157512885647	3.32855173953936
H	-6.34254875943474	-0.37760346169167	-0.21303033602299
H	-8.21622897449129	0.59880044187163	1.05834845637764

H	-3.68806415210241	0.45248969742152	3.03048348458216
C	-3.60705849287213	-5.03437664401671	-0.34024919095842
C	-1.45253163302543	-4.38743239556317	-1.36506171565752
C	-2.98804762117291	-6.42678475122222	-0.31197526468370
H	-4.32673039028576	-4.99554185569478	-1.16803887502319
H	-4.16948668910774	-4.88575656817654	0.58286513989640
O	-2.20838180380152	-6.65039834414678	-1.47778843070617
C	-1.32374712583365	-5.68228554291612	-1.81072889828785
C	-0.45977660605961	-3.33807948600670	-1.76714666046341
C	-0.23616726170155	-6.11475467222669	-2.62658836903000
C	0.76958884087253	-5.16135489152705	-2.93631568929220
C	0.69378166572995	-3.86890523750322	-2.53051979455461
H	-0.97902250341323	-2.60014823660140	-2.39892126403102
H	1.48629465758946	-3.19476410939498	-2.82810888231195
O	1.87715841370991	-5.56440865995647	-3.60078407565004
C	-0.06627484175423	-7.45567697013246	-3.06352907997174
C	0.91327512681415	-7.75469535072127	-3.93919478932333
C	1.77215670584312	-6.66581658464400	-4.51796399388437
C	1.17270974400107	-6.14819599090170	-5.82193125846915
C	3.20172358360008	-7.13199889859561	-4.71581681644272
H	-0.73771031139587	-8.21480970946184	-2.68356379398424
H	1.07161716760188	-8.76801917816680	-4.28938033302339
H	3.61991656972956	-7.47581699048008	-3.76760914399847
H	3.81576171538369	-6.30969190989806	-5.08996087614953
H	3.23883491414667	-7.95305278750221	-5.43652246590265
C	3.01013356769231	2.64867312247975	-0.31319769373501
C	2.72703696678360	1.24458584114242	1.74991984907166
H	2.54339454122345	2.95791002305545	-1.25064978478280
H	3.94882202600661	2.13939214809284	-0.54232323373591
H	3.23063518980499	3.53889676839405	0.28161391819982
H	2.07626964484902	0.52720428552723	2.25562183278737
H	2.89352098385288	2.08831176448666	2.42507397774129
H	3.68676075883229	0.76264089299899	1.54288939638112
H	0.16677446117480	-5.75779714409467	-5.64978225577442
H	1.10435734655169	-6.95304259147195	-6.55886980062520
H	1.79479038430673	-5.34666984821747	-6.23038547726309
C	-4.01941718969199	-7.51381462490004	-0.23035900850030
H	-2.31930735862255	-6.49138322069077	0.55495633679152
C	-4.48461359109400	-7.93012916035158	1.01158699102379
C	-4.55798552063781	-8.07885672545322	-1.38114132466480
C	-5.48003834839726	-8.89116405203698	1.10577684924940
C	-6.01557538497628	-9.45118144476627	-0.04531262415443
C	-5.54980278675639	-9.04390337407997	-1.28752349186818
H	-5.95937184154263	-9.48232033998665	-2.19061026730242
H	-4.18550496126301	-7.76693044474092	-2.34839457152114
H	-4.06050522704566	-7.50240434373071	1.91445871783754
H	-5.83222253688979	-9.20913258414974	2.08047714908999
H	-6.78960359458855	-10.20668175132882	0.02599042270709

Cartesian nuclear coordinates of $(S_0)_{\mathbf{3}\text{-ax}}$ ($\boldsymbol{\omega}$B97X-D4/cc-pVDZ + C-PCM CH_2Cl_2)

C	0.28297006123282	-1.19826408238405	2.19599103913680
C	-0.93144803445319	-0.49210156914119	2.36459566785067
C	-2.09114541331202	-1.05174134838849	1.85019036768936
C	0.32059289637188	-2.43067613951637	1.57453984450324
C	-0.86380806034911	-3.00350939560004	1.06116717762603
C	-2.08545665976771	-2.30084007900581	1.17731550471617
H	1.28342822216941	-2.93324641949185	1.49540712301906
O	-3.24712286639131	-0.34090435816475	2.02067508801401
C	-4.44005599803005	-1.14995802962529	2.05744224769008
C	-3.31326613622052	-2.81997772150705	0.61517962144330
C	-4.53351682758925	-1.94451531379188	0.76219089932521
H	-5.46683881408323	-2.51973851070398	0.73834472653181
H	-4.57931005444086	-1.22151737653985	-0.06948108000968
C	-3.31798763189470	-4.02448682995571	-0.01603890898845
O	1.43763822046598	-0.70639899045420	2.72553042534834
C	-0.90242640878404	0.75484320414050	3.13385442998819
C	1.55721528478870	0.73970729691749	2.84578604187195
C	0.27690096956371	1.32705254158588	3.40099061243196
H	0.35177612041163	2.24934402636598	3.98343893219568
H	-1.84494958811213	1.17924518118758	3.48359514600201

C	-4.45454871919333	-1.98832404071194	3.33067583996934
H	-5.25271268769475	-0.41126541369771	2.10106539045637
C	-3.96670109812872	-1.42382052256115	4.51712156100537
C	-4.95874710382888	-3.29220791474870	3.36294633582852
C	-4.97822159720202	-4.01638692376233	4.55858665741037
C	-3.98156245687095	-2.14588155189430	5.70971180734929
C	-4.48932930410287	-3.44782687141750	5.73475962757020
H	-3.59403078561158	-1.68958712216564	6.62458987494090
H	-4.50204666853237	-4.01682334133607	6.66801439864544
H	-5.33947417502366	-3.76365944417660	2.45518766161441
H	-5.37390462636171	-5.03544538632967	4.56517840688352
H	-3.56773753532814	-0.40726600461881	4.50318302118602
C	-4.55580175733773	-4.64989989091297	-0.61364119688089
C	-2.09097633356421	-4.78309899536735	-0.13202402088343
C	-4.17048281636814	-5.54693476430439	-1.78147920410247
H	-5.28434567129027	-3.89785236450801	-0.94114531716174
H	-5.06015554438011	-5.27956440952702	0.13839581414132
O	-3.25775449837201	-6.55693764339975	-1.30540298406605
C	-2.10243302815169	-6.04503818354373	-0.77982996910624
C	-0.87062150134970	-4.30253900153760	0.39527516007056
C	-0.95710862397994	-6.81439721391277	-0.90696741972454
C	0.25066775808931	-6.30627010774411	-0.37370839226594
C	0.30061880611304	-5.08149651134255	0.26045071215635
H	1.26096774890631	-4.74720428876536	0.65020151345068
O	1.39047066913190	-7.05149845662415	-0.44283716684014
C	-0.93134203929817	-8.13472332221086	-1.54294626795173
C	0.24967156763572	-8.68519299455245	-1.84656762491440
C	1.53707313700396	-7.92830236711356	-1.59581757276793
C	1.89271630536594	-7.07334623574620	-2.81504476824774
C	2.67176069962351	-8.87062209579509	-1.21592889764418
H	-1.87597601852704	-8.64158710893290	-1.74597275827057
H	0.32268903283910	-9.66850531533086	-2.31935632589852
H	2.39660179665152	-9.45939980743797	-0.32785788136101
H	3.58305154287392	-8.29537931611287	-0.99370417976276
H	2.88276159519973	-9.56084631480965	-2.04646990316523
C	1.84722625206388	1.31845443485432	1.45872911415295
C	2.72631203700865	0.94816989905661	3.79955487276554
H	1.01721080416071	1.09806323231088	0.76953018997933
H	2.77520592679543	0.88585188265761	1.05253655960803
H	1.96123901335866	2.41170344358479	1.52197257922862
H	2.49889211690355	0.50846632338817	4.78238372017720
H	2.91909631388676	2.02385692463256	3.92851899153053
H	3.63304038039176	0.47253199801612	3.39662207646561
H	1.08416073382173	-6.35827865694698	-3.03403439325315
H	2.03278235796731	-7.71613131518467	-3.69792399453536
H	2.82445070299825	-6.51582439966412	-2.62994817794548
C	-3.55175121018691	-4.82728651367091	-2.97438765265704
H	-5.04571964954073	-6.12099903132629	-2.11642289908304
C	-3.69436209755879	-3.45199841310634	-3.17978734268089
C	-2.81855766028984	-5.57775225551626	-3.90425083071987
C	-3.11694386120637	-2.83732536380742	-4.29498992469674
C	-2.38698231029506	-3.59017735430460	-5.21446910422907
C	-2.23985600964378	-4.96603452091398	-5.01522884660754
H	-1.67073935817127	-5.56630561561833	-5.73009836223878
H	-2.69444752408665	-6.65194567653039	-3.74801886751851
H	-4.25136230277821	-2.83943683496008	-2.46852244375602
H	-3.23552250382338	-1.76021718730983	-4.43896610294611
H	-1.93233750228743	-3.10785983861466	-6.08371599908919

Cartesian nuclear coordinates of $(S_0)_{3\text{-eq}}$ (ωB97X-D4/cc-pVDZ + C-PCM CH_2Cl_2)

C	0.51715754159323	-1.33279596683896	2.37993390766610
C	-0.68575277821419	-0.59338942836544	2.46475449368004
C	-1.82050256926429	-1.11811048493736	1.86279449094649
C	0.57930741163374	-2.53390624113615	1.70221166073281
C	-0.57944024879609	-3.07057615123671	1.09854096984356
C	-1.80180047653527	-2.36379758125525	1.18377888684903
H	1.53649652614881	-3.05187989991679	1.66751063817984
O	-2.96952187868346	-0.39006687457232	1.97765457460794
C	-3.93332969957643	-0.59782110669978	0.93382351145771
C	-3.02638512427745	-2.90079101967821	0.63316143695490

C	-4.27799785680196	-2.07993180758218	0.82854869607521
H	-4.79532850735057	-2.38306798637750	1.75577298052239
H	-4.98422631102199	-2.21698225167023	-0.00187561226732
C	-3.01637264756403	-4.10605808340762	0.00067521261748
O	1.63805373234861	-0.89478468022544	3.01974339636751
C	-0.67144213037988	0.66587128931836	3.21455674666596
C	1.80977993855617	0.54032281313181	3.18977029233945
C	0.50159271278841	1.19175803655227	3.58446293982970
H	0.55828624706511	2.12942108750317	4.14430771890558
H	-1.62034542177540	1.14532620717981	3.45926514183669
C	-5.14565693129556	0.24752691703681	1.24754849611096
H	-3.48406541148152	-0.26129243705839	-0.01780176868544
C	-5.47462716619639	0.58396225786170	2.56418885720726
C	-5.98397667040601	0.65868882498047	0.20536938729316
C	-7.13889575619691	1.39449270097318	0.47446818073978
C	-6.62638513795463	1.32703961642540	2.83318725432577
C	-7.46259847229550	1.73286356919836	1.79100205787643
H	-6.87170642913022	1.58961580977343	3.86580080875867
H	-8.36352733610033	2.31443013955066	2.00316878452433
H	-5.72853391135894	0.40305271433886	-0.82746407287953
H	-7.78574362280133	1.71036998634109	-0.34821450195243
H	-4.82132532008700	0.26906714569417	3.37986473102614
C	-4.24822207172549	-4.73238178189642	-0.60742813945153
C	-1.77897925168261	-4.84243036330393	-0.13197983039853
C	-4.11628543087692	-6.25156528383436	-0.63593063703401
H	-4.38861546500632	-4.37547458591687	-1.64283435409526
H	-5.15489635527256	-4.47179655292141	-0.04412974731060
O	-2.91268795231713	-6.62458007636600	-1.32743399738327
C	-1.76721264162299	-6.08374177746463	-0.81881026224761
C	-0.56096228552953	-4.34353518076606	0.38583278241449
C	-0.59728019160778	-6.80178840261708	-1.01636158669685
C	0.60988224922901	-6.26309379735623	-0.51248262400696
C	0.63287531355558	-5.07112126327632	0.18389825885917
H	1.59181237849480	-4.71588401985126	0.55821608975469
O	1.77311843679634	-6.96001814226811	-0.64130424033879
C	-0.54978318322976	-8.11458644142518	-1.66577324542320
C	0.63696038120822	-8.62471232657432	-2.01358115408391
C	1.90865025553289	-7.83112383535947	-1.80011004813238
C	2.20782400420149	-6.96333446151641	-3.02483566027345
C	3.07969246944001	-8.74278388448724	-1.45623923869097
H	-1.48508438710330	-8.64954691878998	-1.83822367090119
H	0.72520125246869	-9.60387469323659	-2.49236373679686
H	2.84665152200062	-9.34249816496525	-0.56348459024510
H	3.98065088251133	-8.14305196886432	-1.25782923892028
H	3.28556458050707	-9.42342239853513	-2.29600672530612
C	2.31750584272330	1.12307065886544	1.86852767654673
C	2.84780800452987	0.67408633623610	4.29742251728843
H	1.58248090435414	0.95342132867944	1.06656113777967
H	3.27073306737635	0.64849114816415	1.58696881830361
H	2.47585513896272	2.20807742378462	1.96875650010337
H	2.46708265316977	0.23670634824017	5.23264184536059
H	3.07657397168562	1.73651151331263	4.47060721609137
H	3.77516687987551	0.15518074222642	4.01180376472105
H	1.37871899083481	-6.26344336128028	-3.21267625837022
H	2.33280452129970	-7.59820775267728	-3.91568343069099
H	3.13351199752752	-6.38813123861927	-2.86541848422474
C	-5.27569357683257	-6.91203794329185	-1.34193768965925
H	-4.04689687048269	-6.63168941160588	0.39869904829228
C	-6.38209314791896	-7.34204693379476	-0.60278739995843
C	-5.28240595657745	-7.05277403301527	-2.73374888184975
C	-7.48748017090509	-7.90253254161855	-1.24592937970215
C	-7.49039294814994	-8.04338732527949	-2.63560694251878
C	-6.38409873310098	-7.61987690497494	-3.37670564858520
H	-6.37889336794902	-7.73142758625565	-4.46428575354029
H	-4.41596897105614	-6.72512736154141	-3.31229360768586
H	-6.37648467277799	-7.24148726893909	0.48659219800503
H	-8.34651375365251	-8.23721244867584	-0.65845478099883
H	-8.35262060749589	-8.48669418124722	-3.14054716615536

REFERENCES

[55] S. W. Pelletier, H. P. Chokshi, H. K. Desai, "Separation of Diterpenoid Alkaloid Mixtures Using Vacuum Liquid Chromatography" *Journal of Natural Products* **49**, (1986): 892–900.
[56] S. Bruker, *APEX4, SAINT and SADABS.*, Bruker AXS Inc., Madison (WI, United States of America), 2021.
[57] G. M. Sheldrick, "*SHELXT* – Integrated Space-Group and Crystal-Structure Determination" *Acta Crystallographica Section A: Foundations and Advances* **71**, (2015): 3–8.
[58] G. M. Sheldrick, "A Short History of *SHELX*" *Acta Crystallographica Section A: Foundations and Advances* **64**, (2008): 112–122.
[59] O. V. Dolomanov, L. J. Bourhis, R. J. Gildea, J. A. K. Howard, H. Puschmann, "*OLEX2*: A Complete Structure Solution, Refinement and Analysis Program" *Journal of Applied Crystallography* **42**, (2009): 339–341.
[60] L. Krause, R. Herbst-Irmer, G. M. Sheldrick, D. Stalke, "Comparison of Silver and Molybdenum Microfocus X-ray Sources for Single-Crystal Structure Determination" *Journal of Applied Crystallography* **48**, (2015): 3–10.
[61] A. L. Spek, "*PLATON SQUEEZE*: A Tool for the Calculation of the Disordered Solvent Contribution to the Calculated Structure Factors" *Acta Crystallographica Section C: Structural Chemistry* **71**, (2015): 9–18.
[62] M. D. Hanwell, D. E. Curtis, D. C. Lonie, T. Vandermeersch, E. Zurek, G. R. Hutchison, "Avogadro: An Advanced Semantic Chemical Editor, Visualization, and Analysis Platform" *Journal of Cheminformatics* **4**, (2012): 17.
[63] A. Najibi, L. Goerigk, "DFT‐D_4 Counterparts of Leading meta‐Generalized‐Gradient Approximation and Hybrid Density Functionals for Energetics and Geometries" *Journal of Computational Chemistry* **41**, (2020): 2562‐2572.
[64] D. E. Woon, T. H. Dunning, "Gaussian Basis Sets for Use in Correlated Molecular Calculations. V. Core-Valence Basis Sets for Boron Through Neon" *Journal of Chemical Physics* **103**, (1995): 4572–4585.
[65] E. Caldeweyher, S. Ehlert, A. Hansen, H. Neugebauer, S. Spicher, C. Bannwarth, S. Grimme, "A Generally Applicable Atomic-Charge Dependent London Dispersion Correction" *Journal of Chemical Physics* **150**, (2019): 154122.
[66] V. Barone, M. Cossi, "Quantum Calculation of Molecular Energies and Energy Gradients in Solution by a Conductor Solvent Model" *Journal of Physical Chemistry A* **102**, (1998): 1995–2001.
[67] V. Ásgeirsson, B. O. Birgisson, R. Bjornsson, U. Becker, F. Neese, C. Riplinger, H. Jónsson, "Nudged Elastic Band Method for Molecular Reactions Using Energy-Weighted Springs Combined with Eigenvector Following" *Journal of Chemical Theory and Computation* **17**, (2021): 4929–4945.
[68] Y. Shao, M. Head-Gordon, A. I. Krylov, "The Spin–Flip Approach within Time-Dependent Density Functional Theory: Theory and Applications to Diradicals" *Journal of Chemical Physics* **118**, (2003): 4807–4818.
[69] J. Baker, "An Algorithm for the Location of Transition States" *Journal of Computational Chemistry* **7**, (1986): 385–395.
[70] S. Maeda, K. Ohno, K. Morokuma, "Updated Branching Plane for Finding Conical Intersections without Coupling Derivative Vectors" *Journal of Chemical Theory and Computation* **6**, (2010): 1538–1545.
[71] F. Neese, "Software update: The ORCA Program System—Version 5.0" *WIREs Computational Molecular Science* **12**, (2022): e1606.
[72] F. Neese, F. Wennmohs, U. Becker, C. Riplinger, "The ORCA Quantum Chemistry Program Package" *Journal of Chemical Physics* **152**, (2020): 224108.
[73] F. Neese, F. Wennmohs, A. Hansen, U. Becker, "Efficient, Approximate and Parallel Hartree–Fock and Hybrid DFT Calculations. A 'Chain-of-spheres' Algorithm for the Hartree–Fock Exchange" *Chemical Physics* **356**, (2009): 98–109.
[74] R. L. Martin, "Natural Transition Orbitals" Journal of Chemical Physics 118, (2003): 4775–4777.
[75] T. Lu, F. Chen, "Multiwfn: A Multifunctional Wavefunction Analyzer" *Journal of Computational Chemistry* **33**, (2012): 580–592.
[76] C. Bannwarth, S. Grimme, "A Simplified Time-Dependent Density Functional Theory Approach for Electronic Ultraviolet and Circular Dichroism Spectra of Very Large Molecules" *Computational and Theoretical Chemistry* **1040–1041**, (2014): 45–53.
[77] C. Bannwarth, S. Grimme, "Electronic Circular Dichroism of Highly Conjugated π-Systems: Breakdown of theTamm–Dancoff/Configuration Interaction Singles Approximation" *Journal of Physical Chemistry A* **119**, (2015): 3653–3662.
[78] C. Adamo, V. Barone, "Toward Reliable Density Functional Methods without Adjustable Parameters: The PBE0 Model" *Journal of Chemical Physics* **110**, (1999): 6158–6170.
